# Enhanced plant-derived vesicles for nucleotide delivery for cancer therapy

**DOI:** 10.1038/s41698-024-00556-3

**Published:** 2024-04-06

**Authors:** Sara Corvigno, Yuan Liu, Emine Bayraktar, Elaine Stur, Nazende Nur Bayram, Adrian Lankenau Ahumada, Supriya Nagaraju, Cristian Rodriguez-Aguayo, Hu Chen, Thanh Chung Vu, Yunfei Wen, Han Liang, Li Zhao, Sanghoon Lee, Gabriel Lopez-Berestein, Anil K. Sood

**Affiliations:** 1 Department of Gynecologic Oncology and Reproductive Medicine, The University of Texas MD Anderson Cancer Center, Houston, TX 77030 USA; 2 The University of Texas MD Anderson Cancer Center UTHealth Houston Graduate School of Biomedical Sciences, Houston, TX 77030 USA; 3 Department of Urology, The University of Texas MD Anderson Cancer Center, Houston, TX 77030 USA; 4 Department of Experimental Therapeutics, The University of Texas MD Anderson Cancer Center, Houston, TX 77030 USA; 5 Department of Bioinformatics and Computational Biology, The University of Texas MD Anderson Cancer Center, Houston, TX 77030 USA; 6 Department of Systems Biology, The University of Texas MD Anderson Cancer Center, Houston, TX 77030 USA; 7 Department of Genomic Medicine, The University of Texas MD Anderson Cancer Center, Houston, TX 77030 USA

**Keywords:** Oncology, Cancer

## Abstract

Small RNAs (microRNAs [miRNAs] or small interfering RNAs [siRNAs]) are effective tools for cancer therapy, but many of the existing carriers for their delivery are limited by low bioavailability, insufficient loading, impaired transport across biological barriers, and low delivery into the tumor microenvironment. Extracellular vesicle (EV)–based communication in mammalian and plant systems is important for many physiological and pathological processes, and EVs show promise as carriers for RNA interference molecules. However, some fundamental issues limit their use, such as insufficient cargo loading and low potential for scaling production. Plant-derived vesicles (PDVs) are membrane-coated vesicles released in the apoplastic fluid of plants that contain biomolecules that play a role in several biological mechanisms. Here, we developed an alternative approach to deliver miRNA for cancer therapy using PDVs. We isolated vesicles from watermelon and formulated a hybrid, exosomal, polymeric system in which PDVs were combined with a dendrimer bound to miRNA146 mimic. Third generation PAMAM was chosen due to its high branching structure and versatility for loading molecules of interest. We performed several in vivo experiments to demonstrate the therapeutic efficacy of our compound and explored in vitro biological mechanisms underlying the anti-tumor effects of miRNA146, which are mostly related to its anti-angiogenic activity.

## Introduction

The use of nucleic acids such as small RNAs (microRNAs [miRNAs] or small interfering RNAs [siRNAs]) for biomedical applications is expanding, including as biomedical therapies^1–4^. While nanocarriers have been used successfully for RNA delivery as therapeutics for neurological and metabolic disorders^5–9^, their use in cancer therapy has been more challenging. Some of the limitations of RNAs for therapy include difficulty in targeting the tumor microenvironment (TME), high clearance rates from the bloodstream, and low biocompatibility^10^. Extracellular vesicles (EVs) are membrane-coated vesicles secreted by most cells^11^; mammalian-derived EVs have been considered a potential drug delivery system^12^. However, concerns related to their potential immunogenicity, low loading efficiency^13^, low yield, and high cost^14^ pose major challenges to their use^15^. To overcome these limitations, we considered plant-derived vesicles (PDVs) for efficient delivery of small RNAs to the TME.

Vesicles with similarities to mammalian EVs can be generated from plants. These vesicles, which range from 50 to 1000 nanometers in size, are biocompatible, can be taken up by mammalian cells, and remain stable in the human body^16,17^. They can be isolated using several techniques; one of the most utilized is represented by physical tissue disruption and juice extraction, followed by differential ultracentrifugation. The potential clinical applications of PDVs stem from their intrinsic therapeutic properties (e.g., anti-inflammatory and antioxidant)^18^.

In this study, we developed an alternative drug-delivery system using dendrimers complexed with PDVs to enable highly efficient loading of therapeutic RNAs. Using a systematic strategy, we selected watermelon-derived PDVs (among others) for further development as the delivery vehicles and used a generation 3 dendrimer (polyamidoamine (PAMAM)) to load a therapeutic miRNA mimic (hsa-miR146a-5p) into the PDVs (HEXPO). We demonstrated that the HEXPO system is highly effective for the delivery of miR146a-5p to the TME and results in robust inhibition of tumor growth in three in vivo cancer models. Moreover, we identified a previously undiscovered anti-angiogenic effect of miR146a using both in vitro and in vivo models.

## Results

### Selection of PDV vehicle

**Fig. 1:**
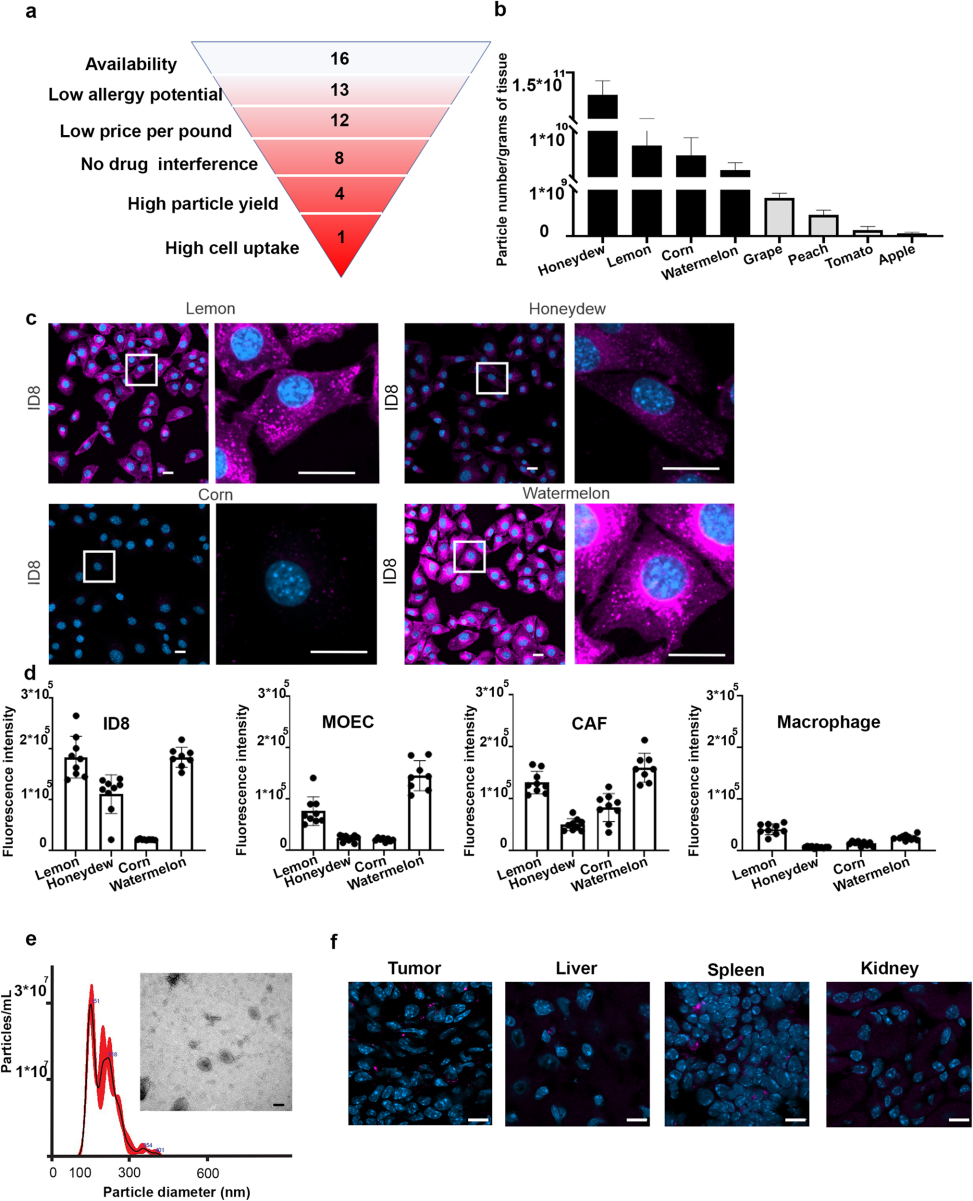
Selection and characterization of plant-derived vesicles (PDVs). **a** Scheme of the systematic approach adopted to select watermelon PDVs. **b** Particle yield from selected species. **c** Cell uptake of PDVs from selected species. The left panels show 20× magnification of random fields, and the right panels show 63× magnification of the same areas. Scale bar = 20 µm. **d** Quantification of cell uptake. Bars represent the means and standard deviations of the ratios of positive cells to fluorescent control microRNA (miRNA) on total cells counted on the basis of the 4′,6-diamidino-2-phenylindole (DAPI) signal. **e** Characterization of PDVs according to Nanotracking analysis and transmission electron microscopy (TEM) imaging results (Scale bar = 100 nm). **f** Fluorescence-based imaging of the tumor, liver, spleen, and kidney from an ID8 model after intraperitoneal administration of fluorescent watermelon PDVs. Scale bar = 10 µm. In the bar graphs, the whiskers represent the standard deviation from the mean. MOECs mouse ovarian endothelial cells, CAFs cancer-associated fibroblasts, mL milliliter, nm nanometer, µm micrometer.

To select a plant from which PDVs could be isolated for therapeutic development, we first considered a list of edible plants (Table 1). This list was further filtered down according to specific criteria, including low allergenic potential (www.mayoclinic.org), low cost per pound at retail (USDA), absence of known drug interactions according to published research^19,20^, high particle yield, and high cell uptake (Fig. 1a and Table 1). We calculated the PDV yield per gram of tissue of nine plants (Fig. 1b). On the basis of these results, we selected four plants (lemon, honeydew, corn, and watermelon) with the highest PDV yield per gram of tissue (>1 × 10^9^). Next, we tested PDVs from these four species for uptake by several cell types found in the ovarian cancer microenvironment, such as mouse ovarian cancer cells (ID8), mouse ovarian endothelial cells (MOECs), mouse-derived primary cultures of cancer-associated fibroblasts (CAFs), and a human monocytic cell line (THP-1). Cell uptake was measured as the mean fluorescence normalized per the total number of cells and divided by single PDV fluorescence intensity, as measured *via* flow cytometry (Fig. 1c). Watermelon PDVs had consistently higher uptake among all four cell types compared to the other three PDVs and thus were selected for further development (Fig. 1d).

**Table 1:** Selection of plant source

Available fruits and nuts					
Tomato					
Apple					
Corn	**Low allergy potential**				
Grape	Tomato	**Low price (lb)**			
Honeydew	Apple	Tomato			
Lemon	Corn	Apple			
Orange	Grape	Corn			
Peach	Honeydew	Grape	**No drug interference**		
*Watermelon*	Lemon	Honeydew	Tomato		
Cranberry	Orange	Lemon	Apple		
Grapefruit	Peach	Orange	Corn		
Kale	*Watermelon*	Peach	Grape	**High particle yield**	
Spinach	Cranberry	*Watermelon*	Honeydew	Honeydew	
Peanut	Grapefruit	Grapefruit	Lemon	Lemon	
Soybean	Kale	Kale	*Watermelon*	*Watermelon*	**High cell uptake**
Walnut	Spinach	Spinach	Peach	Corn	*Watermelon*

List of plants for each selection step.

After characterizing watermelon PDVs by nanotrack analysis and transmission electron microscopy (TEM) (Fig. 1e), we tested whether they could be delivered to the TME following systemic injection. Two weeks after injecting ID8 cells into the peritoneal cavities of C57BL/6 immunocompetent mice, we gave the mice one intraperitoneal injection of CellMask Deep Red-labeled watermelon PDVs (1 × 10^12^ particles). Necropsies were performed 12 h following injection and the delivery of watermelon PDVs to the mouse tumor, muscle, brain, and major visceral organs was examined. We found high uptake of watermelon PDVs in the tumor areas (uptake in an average of 87% of cells counted in each 20× magnification field of tumor sections; Fig. 1f). In addition, we determined whether sections from abdominal tumor nodules showed co-expression of cytokeratin AE1/AE3, an epithelial cancer cell marker, and fluorescent PDVs. As shown in Supplementary Fig. 1A, we identified that the fluorescent signals from both cytokeratin and CellMask Deep Red were visible in the same cells. On the basis of these findings, we selected watermelon-derived PDVs for further studies.

### HEXPO formulation

**Fig. 2:**
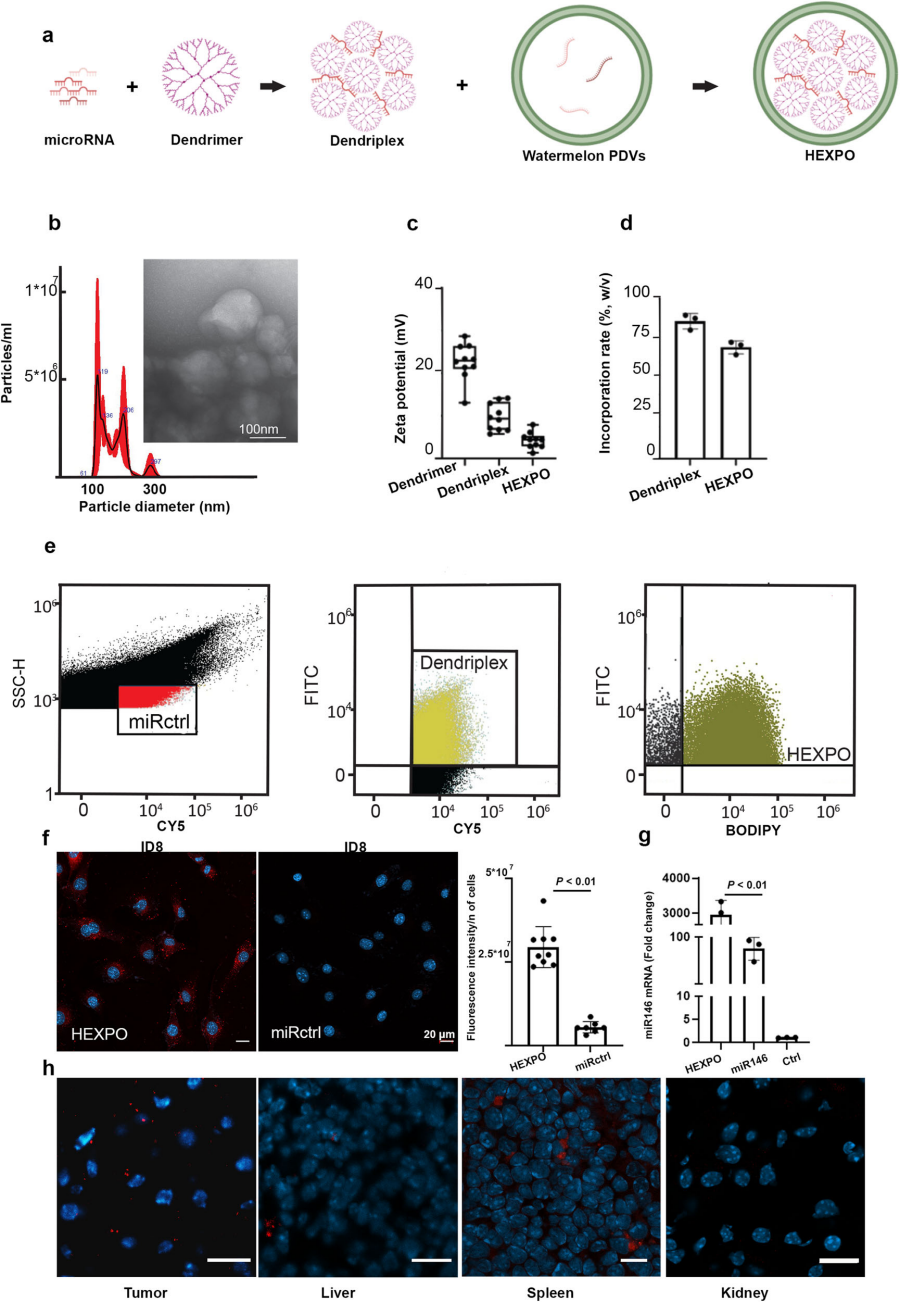
Hybrid exosomal polymeric (HEXPO) nanoparticle formulation and characterization. **a** Schematic representation of HEXPO formulation. **b** Characterization of HEXPO with nanotracking analysis and transmission electron microscopy (TEM) imaging. **c** Measurement of HEXPO charge. **d** Quantification of dendriplex incorporation into HEXPO, measured through spectrophotometry. **e** Quantification of the loading efficiency of control microRNA (miRctrl) plus dendrimers into HEXPO, measured via small-particle flow cytometry. Gating for the population of interest was based on negative controls (unstained compensation beads, unstained PDVs, and 1:1000 diluted BODIPY), single color controls (BODIPY-stained PDVs, Cy5-labeled polystyrene beads, and FITC-labeled compensation beads bound to PAMAM dendrimer), and fluorescence minus one (FMO) controls (cy5-labeled polystyrene beads loaded in BODIPY-stained PDVs, FITC-conjugated PAMAM dendrimers loaded in BODIPY-stained PDVs, and cy5-labeled-miRNA control loaded in FITC-conjugated PAMAM dendrimers bound to compensation beads). **f** Uptake of fluorescent miRctrl after HEXPO treatment of ID8 cells (left) and free miRctrl treatment (right), with quantification. Scale bar = 20 µm. **g** Quantitative polymerase chain reaction of miR146a after HEXPO and miR146 treatment (versus untreated control) of ID8 cells. **h** Fluorescence-based imaging of the tumor, liver, spleen, and kidneys from an ID8 model after intraperitoneal administration of HEXPO with fluorescent miRctrl. In the histograms, the whiskers represent standard deviation from the mean. Scale bar = 10 µm. CY5 cyanine 5, Bodipy TR Bodipy tracker, Fc fold change, mV millivolt, w/v weight per volume, nm nanometer, miRctrl miRNA-negative control, n of cells number of cells, miR146 miRNA 146a, Ctrl control cells, µm micrometer, *P* *p* value.

The delivery of RNA interference molecules (e.g., miRNA) *via* a systemic route remains a challenge because these molecules have negatively charged backbones and are unstable in physiological fluids. Therefore, cationic molecules, such as PAMAM dendrimers, are frequently used to deliver miRNA to cells. PAMAM is also attractive because of its endosomolytic properties (pH-dependent conformational change and pH-dependent membrane perturbation). To further enhance the loading efficiency of nucleotides into watermelon PDVs, we used a positively charged polymeric core to increase the affinity between the nucleic acids and the PDVs. This polymeric core was assembled using generation 3 PAMAM dendrimers complexed with a control miRNA (miRctrl) sequence. These constructs were added to the PDV suspension in a 1:1 ratio to produce hybrid, exosomal, polymeric (HEXPO) nanoparticles (Fig. 2a).

We characterized the HEXPO nanoparticles by nanotracking analysis and TEM and determined their size to be 100–300 nm in diameter (Fig. 2b), with a near-neutral charge (Fig. 2c). The incorporation of the three components (i.e., the miRNA, the dendrimer, and the PDVs) was tested using an electrophoresis retardation assay (Supplementary Fig. 1b), which measures the complexation of the small RNA, the cationic polymer carrier PAMAM, and the slightly negatively charged PDVs. Due to the decreased negative charge density of the miRNA complex, migration of the miRNA/PAMAM cationic polymer carrier and the HEXPO particles inside the electrophoresis gel is not expected to occur. Since we did not observe retardation of free forms of miRNA in the loading wells of the complexed forms, we concluded that there was near complete complexation of the miRNA/PAMAM cationic polymer carrier and HEXPO particles. We then tested the loading efficiency of the three components with quantitative techniques, including spectrophotometry (Fig. 2d) and small-particle flow cytometry (Fig. 2e). Flow cytometry showed that between 45% and 71% of total small RNA particles were loaded in dendrimer particles, and between 96% and 98% of dendriplex particles were incorporated into the PDVs to form HEXPO particles.

We compared cancer cells’ (ID8) uptake of free miRNA mimic labeled with Cy5 and miRNA mimic-loaded HEXPO (Fig. 2f, g) and detected a 5.5-fold higher uptake of Cy5-positive miRNA mimic when ID8 cells were treated with HEXPO. To assess the biodistribution of the HEXPO nanoparticles, we used the ID8 orthotopic tumor mouse model and the fluorescently labeled miRctrl sequence as the cargo. We found substantial delivery of the HEXPO nanoparticles into the TME; 86% of tumor cells showed miRctrl fluorescence (Fig. 2h). Delivery of the nanoparticles was also noted in the spleen. In addition, we examined whether sections from abdominal tumor nodules showed co-expression of cytokeratin AE1/AE3, an epithelial cancer cell marker, as well as fluorescent Cy5 and miRNA mimic loaded HEXPO. As shown in Supplementary Fig. 1a, we found that fluorescent signal from both cytokeratin and CellMask Deep Red was visible in the same cells.

### Selection of miR146a as the therapeutic candidate

**Fig. 3:**
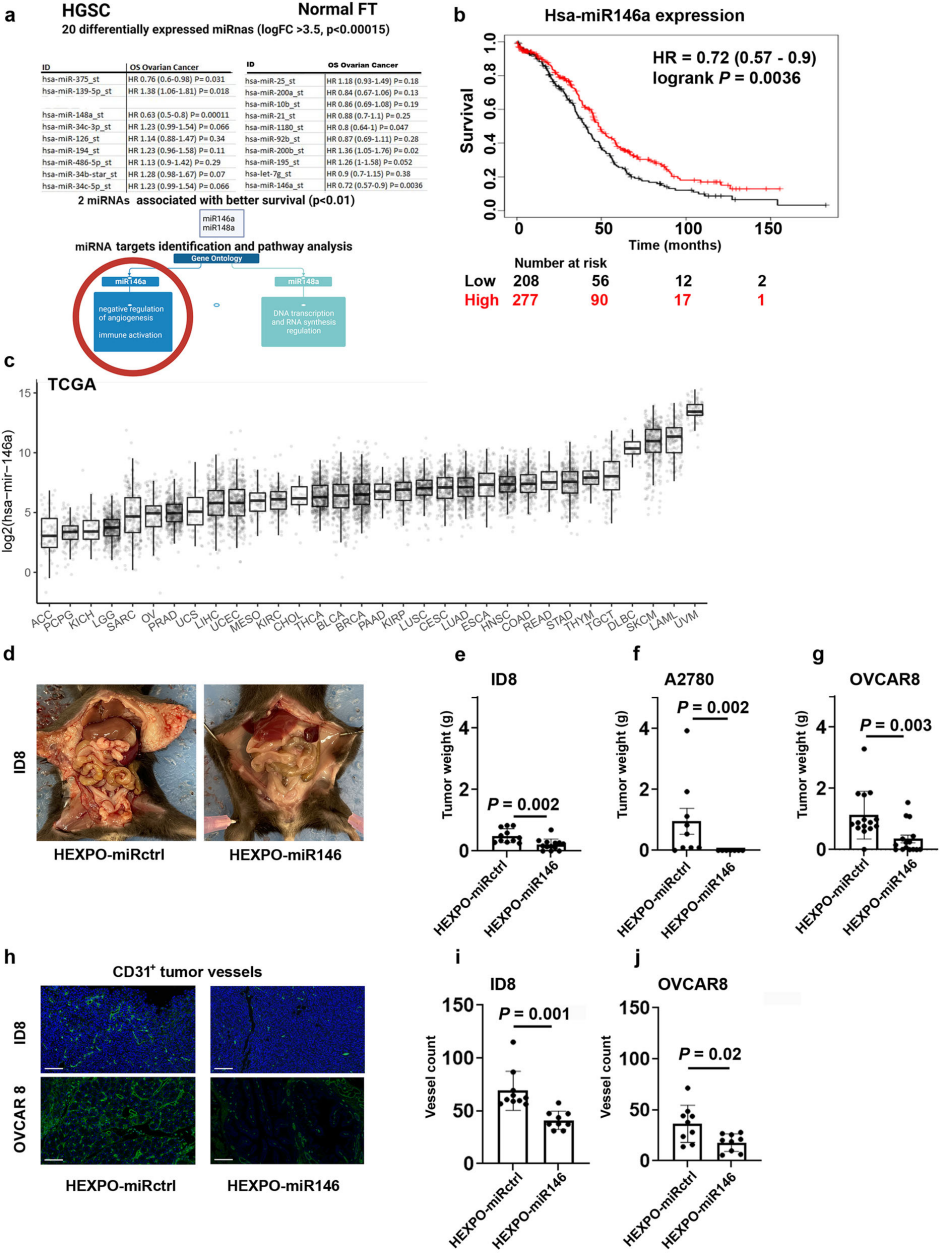
Selection of miR146a. **a** Scheme of the systematic approach adopted to select miR146a. **b** Kaplan–Meier curve representing the survival of patients with ovarian cancer with high or low expression of miR146a using data from a publicly available database (kmplot.com). **c** MiR146a expression from The Cancer Genome Atlas (TCGA) RNA sequencing data of different tumors (https://ropensci.org/blog/2021/11/16/how-to-cite-r-and-r-packages/). **d** ID8 in vivo model treated with hybrid exosomal polymeric control microRNA (HEXPO-miRctrl) and hybrid exosomal polymeric miR146a (HEXPO-miR146). Quantification of tumor weight in **e** ID8, **f** A2780, and **g** OVCAR8 models (two treatment groups per model). **h** Representative images of CD31 vessel-specific staining of formalin-fixed, paraffin-embedded (FFPE) sections from ID8 and OVCAR8 models. Scale bar = 100 µm. Quantification of vessel numbers in sections from FFPE tumor samples from the **i** ID8 model and **j** OVCAR8 model. In the bar graphs, dots represent each sample, bars represent means, and whiskers represent the standard deviations from the mean (standard error of the mean for panel **f**). FT fallopian tube, OS overall survival, HR hazard ratio, *P* *p*-value, logFC logarithmic fold change of expression, TCGA The Cancer Genome Atlas Program, miRctrl miRNA negative control, g grams.

To select a therapeutic candidate for use with the HEXPO delivery system, we used miRNA expression data from high-grade serous ovarian carcinoma tumor tissues and normal fallopian tube samples in the Gene Expression Omnibus (GSE131790), a public database (Fig. 3a). We identified differentially expressed miRNAs in the tumor tissues compared with the normal tissues and selected 20 miRNAs with >3.5-fold increase in expression in the fallopian tube tissues (*P* < 0.00015). These miRNAs were further analyzed for their associations with survival outcomes in patients with ovarian cancer using a Kaplan–Meier plotter (kmplot.com). Among these, high expression of hsa-miR-148a-5p (miR148a) and hsa-miR-146a-5p (miR146a) were associated with improved overall survival (*P* < 0.01; Fig. 3b and Supplementary Fig. 1c). Using *miRPathDB*, a miRNA reference database, we found that miR148a was related to the regulation of RNA synthesis and DNA transcription (Gene Ontology Biologic Processes)^21^ pathways (*P* < 0.01). For miR146a, pathways related to both angiogenesis and inflammation/immunity were enriched, particularly those associated with negative regulation of blood vessel formation (Supplementary Tables 1A, B). We found that ovarian cancer has among the lowest miR146a expression levels among all human cancers with miRNA expression data from the Cancer Genome Atlas (TCGA) database (Fig. 3c), which made it attractive for therapeutic supplementation. Therefore, we selected miR146a for further development using the HEXPO delivery system.

### In vivo therapeutic efficacy of HEXPO

We tested the therapeutic efficacy of HEXPO loaded with miR146a (HEXPO-miR146) in multiple in vivo mouse models of ovarian cancer. Seven days after intraperitoneal injection of A2780 cancer cells, mice were randomized into HEXPO-miRctrl and HEXPO-miR146 groups (10 mice per group). Each mouse was given 10 μg of miRNA (approximately 0.4 mg/kg) per treatment, and treatments were administered twice a week. Compared to treatment with HEXPO-miRctrl, treatment with HEXPO-miR146 resulted in a 67% reduction in tumor weight (*P* = 0.04, Supplementary Fig. 1d and *P* = 0.002 after excluding outliers, Fig. 3f). Similar results were noted with the ID8 (55% reduction; *P* = 0.002; Fig. 3d, e and Supplementary Fig. 1e, f) and OVCAR8 (69% reduction; *P* = 0.003; Fig. 3g) models. Average mouse body weight is shown in Supplementary Fig. 1g–i. The higher mouse body weight in the control group is likely reflective of the presence of ascites. Consistent with the predicted role of miR146a, there was a significant reduction in angiogenesis parameters, as assessed by microvessel density, in the TME (by 51% and 33% in the OVCAR8 and ID8 models, respectively; Fig. 3h–j). Potential treatment toxicity was assessed in the OVCAR8 model *via* blood tests: measurement of circulating markers for hepatic and renal function did not differ significantly between HEXPO-miRctrl- and HEXPO-miR146-treated mice (Supplementary Fig. 2a–h). Expression of miR146 in ID8 tumors was assessed *via* RT-qPCR and found to be higher in the HEXPO-miR146 treated tumors (Supplementary Fig. 3a). We measured the distribution of CD8^+^ lymphocytes *via* immunohistochemistry in tissue sections from ID8 tumors of mice treated with HEXPO miR-146 *versus* HEXPO miRctrl. Tumors from mice treated with miR146 had a significantly higher abundance of CD8^+^ cells in the TME (*P* = 0.04, Supplementary Fig. 4a, b).

### In vitro validation of the dual anti-angiogenic effect of miR146a

**Fig. 4:**
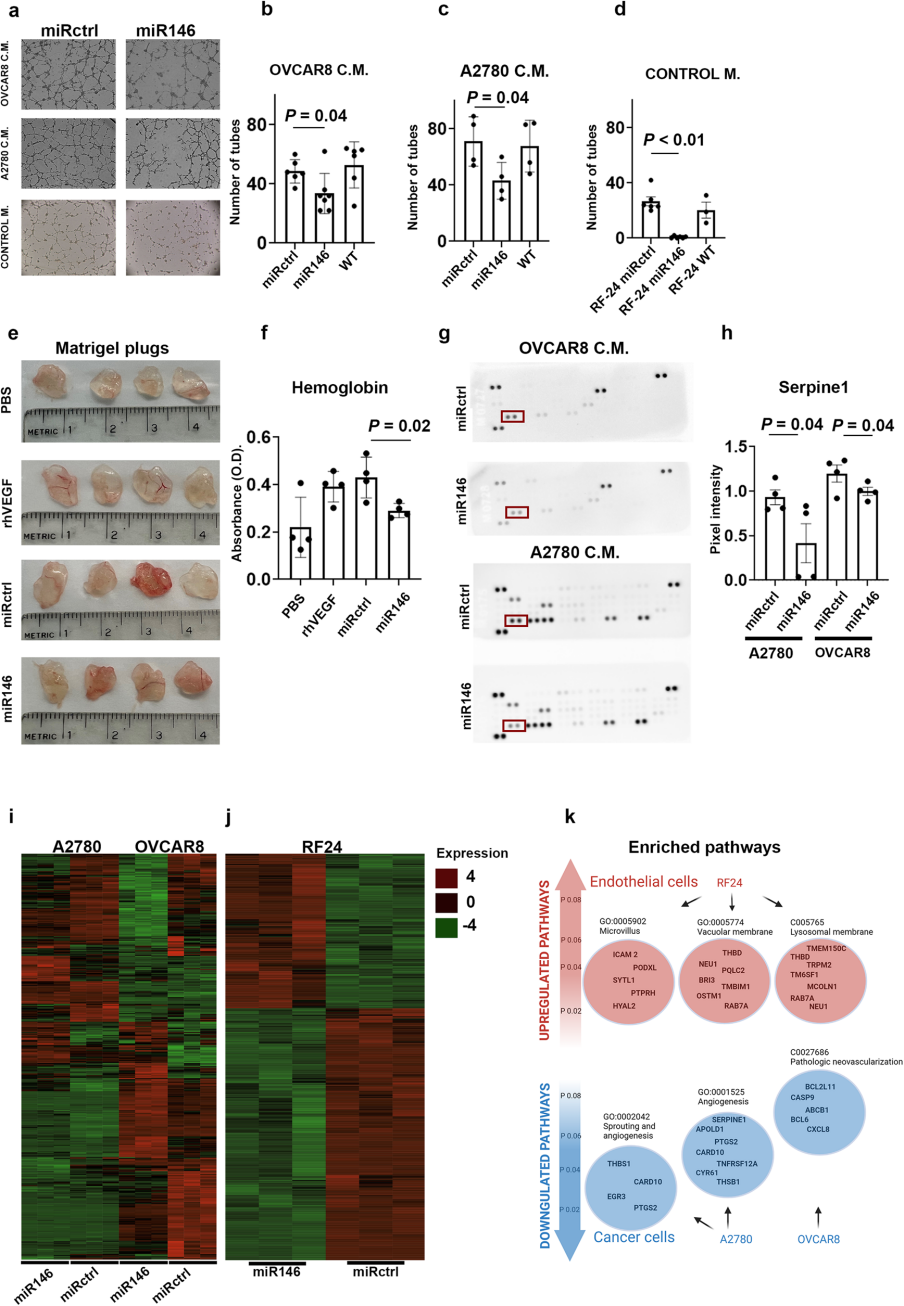
Dual anti-angiogenic effect of miR146a. **a** Representative 5X images of wells from the tube-formation assay of control miRNA (miRctrl)–transfected RF24 cells treated with conditioned medium from miRctrl- and miR146a-transfected OVCAR8 and A2780 cells and miR146a-transfected RF24 cells. **b**–**d** Quantification of tube counts from the tube formation assays in **a. e** Representative images from the Matrigel plug assay performed in nude mice, with quantification in **f**. **g** Angiogenesis array performed on conditioned media from miRctrl- and miR146a-transfected OVCAR8 and A2780 cells (in red SERPINE1 dots), with quantification in **h**. Heatmaps of the gene expression profiles of miRctrl- and miR146a-transfected **i** A2780 and OVCAR8 cells and **j** RF24 cells. **k** Representative image of Gene Ontology pathways enriched in upregulated and downregulated genes in miR146a-transfected RF24, A2780, and OVCAR8 cells. In the histograms, dots represent each sample, bars represent the mean, and whiskers represent the standard deviations from the mean. CM conditioned medium, M medium, *p* *p* value, miRctrl miRNA-negative control, PBS phosphate-buffered saline, rhVEGF recombinant human vascular endothelial growth factor, OD optical densities, ICAM2 intercellular adhesion molecule 2, PODXL podocalyxin-like, SYTL1 synaptotagmin-like protein 1, PTPRH protein tyrosine phosphatase receptor type H, HYAL2 hyaluronidase 2, THBD thrombomodulin, NEU1 neuraminidase 1, PQLC2 PQ loop repeat‐containing, BRI3 brain protein I3, TMBIM1 transmembrane BAX inhibitor motif containing 1, OSTM1 osteopetrosis-associated transmembrane protein 1, RAB7A member RAS oncogene family, TMEM150C transmembrane protein 150C, TRPM2 transient receptor potential melastatin 2, TM6SF1 transmembrane 6 superfamily member 1, MCOLN1 mucolipin transient receptor potential cation channel 1, THBS1 thrombospondin 1, CARD10 caspase recruitment domain family member 10, EGR3 early growth response protein 3, PTGS2 prostaglandin-endoperoxide synthase 2, SERPINE1 serine proteinase inhibitor, Clade E1, APOLD1 apolipoprotein L domain containing 1, TNFRSF12A tumor necrosis factor receptor superfamily, member 12a, CYR61 cysteine-rich angiogenic inducer 61, BCL2L11 Bcl-2-like protein 11, CASP9 caspase 9, ABCB1 adenosine triphosphate (ATP)-binding cassette transporter 1, BCL6 B-cell lymphoma 6, CXCL8 C-X-C motif chemokine ligand 8, *p* value.

To further characterize the anti-angiogenic effects of miR146a, we tested its direct effects on the angiogenic properties of endothelial cells. RF24 endothelial cells transfected with miR146a formed 54% fewer tube structures on average than did RF24 cells transfected with miRctrl (experiments performed in triplicate; Fig. 4a, lower panel and d; *P* < 0.01). We also examined the indirect effects of miR146a on angiogenesis by exposing RF24 endothelial cells to conditioned medium from A2780 or OVCAR8 cancer cells transfected with either miR146a or miRctrl. The number of tubes formed by RF24 cells was significantly lower in cells exposed to conditioned medium from A2780 and OVCAR8 cells transfected with miR146a than in cells exposed to conditioned medium from A2780 and OVCAR8 cells transfected with miRctrl (by 40% for A2780, *P* = 0.04; and 21% for OVCAR8, *P* = 0.04; Fig. 4a top and middle panel, b and c) as compared to control medium (Fig. 4a bottom panel and d). Moreover, we performed an in vivo matrigel plug assay in nude mice using conditioned medium from A2780 cells transfected with miR146a, conditioned medium from A2780 cells transfected with miRctrl, fresh medium supplemented with human recombinant vascular endothelial growth factor (VEGF), and phosphate-buffered saline (PBS). After 1 week, the plugs were removed, and hemoglobin was measured. Representative pictures of the matrigel plugs in each group are shown in Fig. 4e. The hemoglobin levels were 32% lower in the plugs injected with A2780-miR146a conditioned medium than in the plugs treated with A2780-miRctrl conditioned medium (*P* = 0.02; Fig. 4f). We then tested for secreted factors in the supernatant from A2780 and OVCAR8 cancer cells transfected with miR146a or miRctrl for the expression of angiogenesis-related factors.

After transfection with miR146a, SERPINE1 (serine proteinase inhibitor, Clade E1) levels were reduced by 55% in the A2780 medium and 17% in the OVCAR8 medium (*P* = 0.04 for both; Fig. 4g, h). To determine the mechanisms by which miR146a exerts its anti-angiogenic effects, we performed RNA sequencing of RF24, A2780, and OVCAR8 cells following transfection with either miR146a or miRctrl (Supplementary Fig. 3b). Among cancer cells, miR146a transfection resulted in the increased expression of 94–95 genes and the decreased expression of 73–192 genes (Fig. 4i and Supplementary Fig. 3c). In the RF24 cells, 348 genes were upregulated and 568 were downregulated after transfection with miR146a (Fig. 4j and Supplementary Fig. 3d). The pathway analysis for cancer cells indicated that the downregulated genes affected angiogenesis-related processes (e.g., downregulation of the GO pathway GO:0002042 “Cell migration involved in sprouting angiogenesis”; *P* adj = 0.04; Fig. 4k). A pathway analysis of RF24 cells indicated that the upregulated genes were associated with vacuolization effects (GO:0005774) and late endosome formation (CO05765), processes that are involved in transcytosis and vascular permeability of endothelial cells and, thus, loss of cell adhesion and impaired tube formation^22,23^ (Fig. 4k). The GO pathway (GO:0002042) that was enriched among the downregulated genes in A2780 cells transfected with miR146a (Fig. 4k) involves genes that are particularly related to angiogenesis, such as *CARD10*^24,25^ and *EGR3*^26^. Interestingly, *SERPINE1* was the most downregulated gene in these A2780 cells (log_2_ fold change = −3.07; *P* adj = 4.95*10^−12^; Supplementary Table 2).

We measured the downregulation of interleukin-1 receptor-associated kinase 1 (IRAK1) and SERPINE1 in A2780 and OVCAR8 with quantitative real-time PCR and found reductions of 2.2- (IRAK1) and 1.5-fold (SERPINE1) in OVCAR8 (*P* = 0.004 and 0.05) and 3.8- (IRAK1) and 6.7-fold (SERPINE1) (*P* = 0.07 and 0.05) in A2780 cells (Supplementary Fig. 4c, d). We also confirmed the downregulation of IRAK1 protein expression by Western blot analysis in A2780 (1.5-fold, *P* = 0.04, paired t-test) and OVCAR8 (1.3-fold, *P* = 0.14, paired t-test) cells (Supplementary Fig. 4e–g. Original blots for IRAK1 and VINCULIN are displayed in Supplementary Fig. 5). Finally, we verified the decreased expression of SERPINE1 in the supernatant from A2780 cells treated with miR146 (by 1.7-fold, *P* < 0.0001) and in the supernatant from OVCAR8 cells (by two-fold, *P* = 0.0002) (Supplementary Fig. 4h, i). Taken together, these findings demonstrate an impact of miR146a expression on the angiogenic processes. This effect is both direct for endothelial cells (i.e., miR146a decreases expression of genes related to vessel formation and vessel integrity and causes reduced tube formation activity), and indirect for cancer cells (i.e., miR146a decreases the expression of secreted proteins, such as SERPINE1, that stimulate vessel formation).

Using an algorithm from the DIANA microT web server (http://www.microrna.gr/webServer), we performed an in-silico target-prediction analysis to determine whether SERPINE1 could be directly regulated by miR146a. The analysis did not reveal complementarity between the sequences of hsa-miR-146a-5p and SERPINE1 (Supplementary Table 3). Therefore, we considered an alternate possibility that SERPINE1 is regulated indirectly *via* an upstream transcription factor. It has been shown that nuclear factor-κB and several interleukins are involved in the regulation of SERPINE1 expression^27^. IRAK-1 is an important protein that plays a role in the activation of nuclear factor-KB’s transcriptional activity^28,29^. Interestingly, we found that IRAK-1 was downregulated in the A2780 and OVCAR8 cancer cell lines after miR146a transfection (Supplementary Table 2). There was complementarity between the sequences of hsa-miR-146a-5p and IRAK-1 in our in-silico analysis (Supplementary Table 4).

## Discussion

Here, we formulated an alternative delivery system for the therapeutic delivery of small RNAs, with low costs and high biocompatibility, and verified its robust therapeutic efficacy in vivo. In addition, we reported a previously undiscovered role for miR146a in ovarian cancer biology. Nanoparticle-based therapeutics using hybrid organic/inorganic systems have demonstrated flexibility and can be explored for different clinical applications^30–32^. Hybrid nanoparticles allow for efficient drug loading, facilitate targeted delivery, and possess intrinsic therapeutic properties, which are considered attractive for cancer therapy applications.

Exosomal delivery systems for therapeutic molecules have recently been considered as an alternative drug carrier system^33^. However, the translation of this system into the clinic is limited by major challenges, including insufficient loading efficiency^34,35^, poor delivery to the TME, low scalability, high production costs, and absence of a standardized isolation technique. To overcome these hurdles, plant-derived vesicles have been considered for therapeutic applications^36^. Isolation of PDVs has mainly been achieved using differential ultracentrifugation, which allows for high yield with minimal technical effort. Some studies have proposed the use of PDVs (e.g., from ginger^37^ and grapefruit^38^) as drug vehicles. Here, we propose an innovative delivery system, called HEXPO, that was composed of a generation 3 dendrimer complexed with watermelon-derived PDVs. This formulation allows for high loading efficiency of therapeutic small RNAs.

Our strategy of incorporating PAMAM into the PDVs protects the target cells from membrane disruption: our in vivo data showed a favorable safety profile of HEXPO-miR146. While the high efficiency of nucleic acids binding demonstrated by the PAMAM generation 3 dendrimer is mostly explained by their highly branched and positively charged three-dimensional structure, such structures have contributed to inflammatory side effects^39^, and limited clinical development^40^. The direct interaction of the cationic branches with the negatively charged cell membranes is thought to lead to formation of nanopores in the cell membrane, which can cause leakage of cellular content and eventually cell death^39^.

Besides showing robust therapeutic efficacy of HEXPO in several cancer models, we identified a previously unknown anti-angiogenetic role of miR146a. We also considered the possible role of immune cell activation in exerting the anti-tumor activity of miR146 and found that tumor tissues from HEXPO-miR146–treated ID8 mice had a higher density of CD8^+^ cells than did tumors from HEXPO-miRctrl treated mice. MiR146 is known to regulate inflammatory pathways^41^, but its role in anti-tumor immunity is not well understood. It is known that many pro-angiogenic molecules (e.g., VEGF) can interfere with T-cell trafficking and maturation. In addition, the vessel-stabilizing effect of many anti-angiogenetic molecules allows for more effective immune cell penetration in the TME^42^. Given the observed anti-angiogenic effects of miR146, a combination approach with immune-targeted drugs could be considered in additional work.

## Methods

### PDV source selection

The allergy potential of fruits and nuts was assessed using the Mayo Clinic list of most common food allergens (https://www.mayoclinic.org/diseases-conditions/food-allergy/symptoms-causes/syc-20355095); the retail price of each fruit as of January 2022 was retrieved from the USDA (https://www.marketnews.usda.gov/mnp/hm-home). Drug interactions were assessed by a literature search, which retrieved results only for spinach, kale, and grapefruit.

### PDV isolation

We used 10% bleach and antibacterial soap, followed by a thorough rinse with tap water, to perform the initial disinfection of the plants. The same disinfecting and cleaning processes were applied to all blending vessels and centrifuge tubes. Plants were blended with a Ninja blender (BL610) for approximately 2 minutes, with PBS added during blending. The tissues underwent additional blending with a Vitamix 7500 for about 3 minutes and the solutions were then filtered with a mesh nylon filter bag (pore size of 8 × 12 μm). The collected juices were centrifuged sequentially at 700 × *g* for 10 min, 2000 × *g* for 20 min, and 10,000 × *g* for 30 min at 4 °C, to remove tissue fragments and large cellular debris. The remaining supernatant was centrifuged at 100,000 × *g* for 2 h to obtain a pellet of the remaining vesicles. The pellet was resuspended in PBS. Each 4 ml of the resuspended pellet was loaded in 24 ml of 1 M sucrose, followed by 100,000 × *g* centrifugation for 12 h. The initial and final 1 ml of solution derived from sucrose gradient centrifugation were discarded together with the remaining pellet. The resulting 26 ml of solution was collected. Next, 170 ml of PBS was added to the solution and centrifugation was performed at 100,000 × *g* for 2 h. The resulting pellets were resuspended in PBS as PDVs.

### PDV uptake by different cancer microenvironment cell lines

Tomato, apple, corn, grape, honeydew, lemon, orange, peach, and watermelon PDVs were isolated from extracted juice using gradient centrifugation, followed by a sucrose gradient. The resultant PDV pellets were hydrated in PBS, and the PDV concentrations were quantified using NanoSight NS300 (Malvern Panalytical, Malvern, United Kingdom) according to the manufacturer’s protocol. Samples were diluted in PBS to obtain 10^11^ particles per mL solution. CellMask Deep Red (cat# C10046, Thermo Fisher Scientific) was added at 1:1000 dilution according to the manufacturer’s instructions. PDVs were incubated at 37 °C for 30 min and then washed twice with PBS, followed by 2 h of centrifugation at 100,000 × *g* at 4 °C. We then incubated PDVs with different cell lines (10^9^ PDVs with 20,000 cells per well). Mouse ovarian cancer (ID8) cells, mouse ovarian endothelial cells (MOECs), mouse cancer-associated fibroblasts (CAFs), and activated THP-1 cells were tested. Cells were seeded in an 8-well chamber (cat# 80826, Ibidi, Gräfelfing, Germany) and treated with PDVs for 3 h. Cells were then washed three times with PBS and fixed with 4% paraformaldehyde (Electron Microscopy Science) for 15 min, followed by three PBS washes. 4′,6-diamidino-2-phenylindole (DAPI) (PerkinElmer) working solution was used for nuclei staining followed by three PBS washes. Images were acquired with a Zeiss LSM 800 microscope. Fluorescent intensity statistics were performed with Imaris software. Mean intensity per cell after uptake was normalized on PDVs by single-particle fluorescent intensity.

### HEXPO formulation

To synthesize the HEXPO nanoparticles, miRNA-containing polymer cores were formulated using generation polyamidoamine (G3 PAMAM) dendrimers (Sigma) in a 1:25 nitrogen-to-phosphate ratio. The solution containing miR-PAMAM polymer cores dendrimers was dissolved in 5% dextrose. The miRNA cores were subsequently incorporated into EVs drop-wise at a 1:1 ratio.

### HEXPO complexing of PAMAM and miRNA into EVs

miR146a was complexed with PAMAM (cat# 153891-46-4, Sigma) dissolved in 5% dextrose at a 1:25 nitrogen-to-phosphate ratio. The N/P (nitrogen-to-phosphate) ratio is defined as the ratio of positively charged homopolymer amine (N = nitrogen) groups to negatively charged nucleic acid phosphate (P) groups. A gel retardation assay (corresponding to 1.5% agarose gel electrophoresis) was used to assess the complexing ratio of PAMAM/miRNA (w/w) in the watermelon PDVs.

### Encapsulation efficiency of the miR146a oligonucleotide into the HEXPO-miR146a structure

The percent entrapment of the miR146a oligonucleotide into the HEXPO structure was determined after the reconstitution of HEXPO-miR146a-lyophilized vials. The vials were reconstituted and shaken until a milky-white suspension formed. Right after reconstitution, the suspension was centrifuged at 400 × *g* for 15 min at ambient temperature. Next, the supernatant was put aside to determine the presence of unencapsulated miRNA. The concentration of miRNA was then measured using a Nanodrop 2000 Spectrophotometer (Thermo Fisher Scientific) at a 260-nm wavelength. The encapsulation efficiency of the miR146a oligonucleotide in the HEXPO structure was determined using the following formula:

encapsulation efficiency % = (amount of miRNA in HEXPO)/(initial amount of miRNA for drug loading) × 100.

### MiRNA control and dendriplex loading efficiency via flow cytometry and RT-qPCR

The incorporation efficiency of HEXPO particles was assessed via flow cytometry analysis using the Cytek Aurora Flow Cytometer and Cy-5-labeled control miRNA, Cy-5-labeled polystyrene beads (cat# PS200-AMS5-1), FITC (cat# 3326-32-7, Sigma)-labeled PAMAM dendrimers, compensation beads (cat# 004222 Thermo Fisher), and Bodipy-TR ceramide (cat# D754, Invitrogen)–stained watermelon PDVs. BODIPY was diluted 1:1000 to stain PDVs according to the manufacturer’s instructions. To prove single vesicle detection according to the ISEV guidelines for the flow cytometry-EV reporting framework^43^, the following controls and samples were prepared: negative controls: unstained compensation beads, unstained PDVs, and 1:1000 diluted BODIPY; single color controls: BODIPY-stained PDVs, Cy5-labeled polystyrene beads, and FITC-labeled compensation beads bound to PAMAM dendrimer; and fluorescence minus one (FMO) controls: cy5-labeled polystyrene beads loaded in BODIPY-stained PDVs, FITC-conjugated PAMAM dendrimers loaded in BODIPY-stained PDVs, and cy5-labeled-miRNA control loaded in FITC-conjugated PAMAM dendrimers bound to compensation beads.

A fully stained sample with Cy5-labeled polystyrene beads bound with miRNA control + FITC-labeled compensation beads bound with PAMAM dendrimers was loaded in BODIPY-stained PDVs. Lysed full samples and dilutions from fully stained samples (1:5, 1:25, and 1:125) were prepared to confirm that single particles were analyzed (diluted and lysed samples showed a decreased signal, as expected). Single particle fluorescence intensity was assessed with the Cytek Aurora system under low-flow speed and high sensitivity; 10,000 events were collected per sample. Positive particles were selected on the basis of single-color controls and FMO. Negative selection was based on unstained HEXPO particles. This experiment was repeated twice.

Quantitative real-time PCR was performed to assess in vitro cell uptake of HEXPO-miR146a particles. We seeded 5 × 10^4^ OVCAR8 cells in each well of a 24-well plate. The day after, 2 μg HEXPO-miR146a and 2 μg of free miR146a in serum-free medium were added to treatment and control wells and incubated for 3 h. Cells were then washed with PBS three times, trypsinized, and collected. Stem-loop RT-PCR using Hsa-miR-146a MicroRNA Assays (TaqMan®) were performed to measure miR146a levels. RNU44 was used as endogenous control. Three technical replicates and three biological replicates were performed.

### In vivo biodistribution of watermelon-PDVs and HEXPO

To assess the biodistribution of the watermelon-PDVs and HEXPO, we performed an in vivo biodistribution experiment. Cy3-labeled small RNA control (cat# CP-004500-01-20, Horizon Discovery) was used to prepare miR-loaded HEXPO structures, and watermelon PDVs stained with CellMask™ Deep Red Plasma Membrane Stain (cat#C10046, Thermo Fisher) were prepared. ID8 cells were injected intraperitoneally into six C57BL/6 immunocompetent mice (1 million cells per mouse in 200 μL of Hank balanced salt solution (Life Technologies)). At tumor establishment, mice that showed a significant amount of ascites were excluded from the analysis. The remaining mice were assigned to receive (1) HEXPO-miRctrl; (2) PBS; or (3) fluorescent watermelon PDVs. Two doses of HEXPO (15 μg of miRNA per dose given 16 h apart) and one dose of 10^12^ watermelon PDVs were injected intraperitoneally. Twelve hours after treatment completion, mice were sacrificed via asphyxiation and cervical dislocation. Tissues from tumor nodules and organs, including the kidneys, spleen, liver, lungs, and heart, were embedded in optimal cutting temperature compound (Miles, Inc.) and frozen. Sections were then cut and counterstained with DAPI for nuclei identification. Cells that were positive for the miRctrl and CellMask™ Deep Red fluorescent signal were counted in four randomly chosen fields at 20× magnification and normalized per the total number of cells.

### Immunofluorescence

Sections were cut from frozen samples embedded in optimal cutting temperature compound of peritoneal tumor nodules, livers, and spleens from the ID8 model after PDV and HEXPO injection. Slides were fixed in cold acetone and then washed in TBS-T and blocked with 4% fish gelatin. Slides were then incubated with a primary antibody against cytokeratin AE1/AE3 conjugated with Alexa Fluor 488 at the concentration of 1 µg/mL (cat#53-9003-82, Invitrogen) overnight at 4 °C. Sections were washed in PBS, counterstained with Hoechst 1:5000 for 10 min, and then washed again and imaged with a Zeiss LSM 800 microscope.

### In vivo experiments and animals

To test the therapeutic efficacy of HEXPO, we performed experiments using ID8, A2780, and OVCAR8 cells. ID8 and OVCAR8 cells isolated from tumors formed after intraperitoneal injection in mice (one in vivo passage for ID8 and two passages for OVCAR8, *ip1*, or *ip2*) were used for in vivo experiments. Six- to twelve-week-old female athymic nude mice were obtained from Taconic Biosciences (Rensselaer) and used for injection of A2780 (20 mice total, 10 mice per group) and OVCAR8 (30 mice total, 15 per group). C57BL/6 mice were also purchased from Taconic Biosciences and used for the injection of ID8 cells (20 mice total, 10 mice per group). The study protocol was approved and supervised by the Institutional Animal Care and Use Committee (IACUC) at The University of Texas MD Anderson Cancer Center, and all of the in vivo studies were performed following the American Association for Laboratory Animal Care institutional guidelines. Five animals were kept in each cage and maintained under specific pathogen-free conditions. For all the models, cells (1 × 10^6^ ID8 cells, 1 × 10^6^ A2780 cells, or 4 × 10^6^ OVCAR8ip2 cells) in 200 μL of Hank balanced salt solution were injected intraperitoneally. After tumors had been establishment (7 days for ID8 and A2780 cells and 14 days for OVCAR8ip2 cells), the mice were divided into 2 groups: 1) HEXPO-miRctrl (mice treated twice a week with 100 μL of HEXPO solution, loaded with 5 µg of negative miR mimic (cat# 4464061, Thermo Fisher Scientific) or 2) HEXPO-miR146 (mice treated twice for 1 week with 100 μL of HEXPO solution, loaded with 5 µg of miR146a mimic [cat# 4464070, Thermo Fisher Scientific]).

When the control animals showed significant morbidity (at 6 weeks from cell injection for the ID8 model, at 4 weeks for the A2780 model, and at 8 weeks for the OVCAR8 model), they were euthanized using cervical dislocation. Mouse weight, number of tumor nodules, and tumor weight were recorded during necropsies. Tissue specimens were either fixed with 10% buffered formalin, frozen in optimal cutting temperature compound, or snap-frozen in liquid nitrogen. For the A2780 model, we performed a Mann–Whitney test, and additionally a sensitivity test with the identification and exclusion of outliers according to the Robust Regression and Outlier Removal method (ROUT)^44^ (three values from the HEXPO-miR146 group and one from the HEXPO-miRctrl group). GraphPad 9.0 (GraphPad Software) was used for statistical analysis and graph production. An unpaired *t*-test was used for the ID8ip1 and OVCAR8ip2 models.

### Vessel density measurement in mouse tumor samples

Tumor tissues from the ID8 mouse model used for the therapeutic efficacy experiments were harvested and immediately fixed in formalin and embedded in paraffin. Sections were cut and subjected to immunohistochemistry staining with tyramide signal amplification technology using Opal technology (Akoya Biosciences), according to the manufacturer’s instructions. Briefly, sections were deparaffinized, subjected to antigen retrieval, and quenched with horseradish peroxidase. After 10 min of blocking, the sections were incubated with antiCD31 antibody (cat# 77699, Cell Signaling Technology) at a 1:20 dilution in Opal antibody diluent/block (cat# ARD1001EA, Akoya Biosciences) overnight at 4 °C. After being washed in tris-buffered saline and polysorbate 20, slides were incubated with secondary antibody solution Opal Anti-MS + RB HRP (cat# SKU ARH1001EA, Akoya Biosciences) for 10 min at room temperature and washed again in tris-buffered saline and polysorbate 20. Finally, Opal 520 reagent (cat# SKU FP1487001KT, Akoya Biosciences) was added at a 1:50 concentration in amplification diluent (cat# FP1135, Akoya Biosciences). After the sections were washed in PBS, DAPI was added to counterstain them. The sections were mounted and imaged on a LEICA DM4000 B LED microscope. Pictures from three or four random, high-power fields (20×) per mouse were taken using LASX software (LAX 5.1.0), and vessels were counted. GraphPad 9.0 was used for the statistical analysis (independent *t*-test) and graph production. Slides were also scanned at a Vectra Polaris Automated Quantitative Pathology Imaging System; representative images were acquired using Visiopharm and are displayed in Fig. 3H.

### T-lymphocyte immune staining in ID8 tumors

Tumor tissues from the ID8 mouse model used for the therapeutic efficacy experiments were harvested and immediately fixed in formalin and embedded in paraffin. Sections from four mice were cut and subjected to immunohistochemical staining with anti-CD8 antibody (98941 s CST, at 1:25 dilution), plus counter-staining with hematoxylin. Sections were mounted and CD8-positive cells from four random fields at ×20 were counted. The statistical analysis was performed using GraphPad; p-values were calculated using a t-test considering each value as an independent observation within each mouse. Results are displayed in Supplementary Fig. 4.

### Cell lines and cultures

We used the human ovarian cancer cell lines A2780 and OVCAR8, the human monocyte cell line THP1, the mouse ovarian cancer cell line ID8 and MOEC (obtained from the MD Anderson Characterized Cell Line Core Facility), and the human endothelial cell line RF24. All cell lines were routinely tested for Mycoplasma by using a Universal Mycoplasma Detection Kit (ATCC) and fingerprinted by short tandem repeat (STR) analysis by the Cell Line Core at the university of Texas, MD Anderson. A CAF cell line was derived from cancer-associated fibroblasts isolated from tumors established in C57BL/6 mice after intraperitoneal injection of ID8 cells. Tumors were dissociated and both positive and negative selections for CAFs were performed during sorting. CAFs were also tested for Mycoplasma. The A2780 and OVCAR8 cells were maintained in Roswell Park Memorial Institute medium (cat# SH30027.01, Cytiva), 10% fetal bovine serum, and 0.1% gentamicin. The ID8 cells were grown in Dulbecco-modified Eagle medium (cat# 11995-065, Gibco) 5% fetal bovine serum, 1% insulin-transferrin-selenium supplement, and 0.1% gentamicin. The murine ovarian endothelial cells (MOECs) were cultured in Dulbecco modified essential medium supplemented with 10% FBS and 0.1% gentamicin sulfate. Human monocytic THP-1 (RRID: CVCL_0006) cells were obtained from the American Type Culture Collection (ATCC) and the University of Texas MD Anderson Cancer Center Characterized Cell Line Core Facility and were differentiated into macrophages by adding 30 ng/ml PMA for 24 h. The RF24 cells were a kind gift from Dr. Lee M. Ellis (from MD Anderson’s Department of Surgical Oncology) and were grown in modified Eagle medium (cat# 10-010-CV, Corning) supplemented with 10% fetal bovine serum and 1× sodium pyruvate, 1× non-essential amino acids, 1× modified Eagle medium vitamins, and 1× penicillin/streptomycin. The cell cultures were maintained at 37 °C in a 5% CO_2_ incubator with 95% humidity. For the in vivo injections, the cells were trypsinized and centrifuged at 1200 rotations per minute for 5 min at 4 °C and then washed and reconstituted in serum-free Hank balanced salt solution.

### miR146a transfection in cell lines

The cells were transfected with 100 nM specified miRNAs using Lipofectamine RNAiMAX Transfection Reagent (cat# 13778500, Thermo Fisher Scientific) at a ratio of 3 μL of reagent to 2 μg of miRNA. The cells were treated with miRctrl or miR146a mimics for 4 h in serum-free media before being incubated in fresh complete media for 48 h. The miRNAs were purchased from Thermo Fisher Scientific (cat# 446470 for hsa-miR-146a-5p mimics) and Thermo Fisher Scientific (cat# 4464061 for *mir*Vana miRNA Mimic, negative control #1).

### Stem-Loop RT-PCR for miR146a measurement

Total miRNA isolated from transfected cells (A2780, OVCAR8, and RF24) was isolated using the PureLink^TM^ miRNA Isolation kit (cat# K157001 Thermofisher). For ID8 tumors, instead, after homogenization, total RNA was isolated using the Direct-zol RNA miniprep kit (cat# R2050 Zymo Research, Irvine, CA), according to the manufacturer’s instructions. Complementary DNA was synthesized from 10 ng of the total RNA using the TaqMan MicroRNA reverse transcription kit according to the manufacturer’s instructions (cat#4366597, Applied Biosystems, Waltham, MA) and the specific primers for reverse transcription (has-miR-146a RT cat#000468 for miR146a, RNU44 RT cat# 001094 as the housekeeping gene for human samples, and snoRNA135 RT cat# 001230 as the housekeeping gene for mouse tissue). A quantitative PCR analysis was performed in triplicate with Taqman Universal PCR Master Mix (cat#4304437 Thermo Scientific), according to the manufacturer’s instructions, using the primers described above. The relative quantification was expressed as the fold change of the target gene versus the housekeeping gene.

### RNA isolation and quantitative PCR

Total RNA was isolated from OVCAR8 and A2780 cells 48 h after transfection (performed as described above) using Direct-zol RNA miniprep kit (cat# R2050 Zymo Research, Irvine, CA) according to the manufacturer’s instructions. Complementary DNA was synthesized from 200 ng of total RNA using Verso cDNA Synthesis Kit (cat# AB1453A, ThermoFisher, Waltham, MA). The resulting cDNA was then used to amplify IRAK1 and SERPINE1; quantitative PCR was performed in triplicate using Power Syber Green (cat# 4367659, ThermoFisher) and specific primers for IRAK1 (forward: TCAGCTTTGGGGTGGTAGTG; reverse TAGATCTGCATGGCGATGGG), for SERPINE (forward: CCGGAACAGCCTGAAGAAGTG; reverse: GTGTTTCAGCAGGTGGCGC) and for 18S (forward: CGCCGCTAGAGGTGAAATTC; reverse: TTGGCAAATGCTTTCGCTC) (Sigma-Aldrich, St. Louis, MO). Expression levels are shown as log2 fold change of IRAK1 and SERPINE1 expression after normalization to the housekeeping gene 18S and to cells transfected with control miRNA. Statistical analyses were performed using GraphPad Prism 9.0.0 and t-test.

### Protein isolation and Western blot analysis

The cell lysates from OVCAR8 and A2780 cells was collected 48 h after miRNA transfection. Western blot analysis was performed according to the manufacturer’s guidelines (https://www.sigmaaldrich.com/US/en/technical-documents/protocol/protein-biology/western-blotting/western-blotting) using anti-human antibody for IRAK1 (D21G7, CST, 1:1000 for A2780, and 1:500 for OVCAR8). The experiments were run in triplicate. Optical densities were measured using Image J (Java 1.8.0_172). Statistical analyses were performed using GraphPad Prism 9.0.0 and paired t-test.

### ELISA for SERPINE1

Supernatant from OVCAR8 and A2780 cells transfected with miR146, as described above, was collected 48 h after transfection and centrifuged at high speed for 15 min. The pellet was discarded and ELISA for SERPINE1 using the Human PAI1 ELISA Kit (ab269373) was performed according to the manufacturer’s instructions. Experiments were run in triplicate. Graphs from one representative experiment are shown. Statistical analyses were performed using GraphPad Prism 9.0.0 and t-test.

### Tube formation assay

Matrigel (12.5 mg/mL) was thawed at 4 °C. Then, 10 µL of thawed Matrigel was quickly added to each well of a µ-slide angiogenesis plate (cat# 81506 Ibidi GmbH) and allowed to solidify for 10 minutes at 37 °C. Untreated and RF24 cells transfected with miR146a or miRctrl were then added to the wells (20,000 cells per 50 µL per well) and incubated for 12 h at 37 °C with fresh or conditioned medium from OVCAR8 and A2780 cells transfected with miR-146a or miRctrl. The experiments were performed in triplicate. Using a Leica DM4000 B LED microscope, we obtained one image per well at 5× magnification and counted the number of tubes per image. GraphPad 9.0 was used for the statistical analysis (an unpaired t-test) and graph production. The experiments were repeated three times and representative pictures and graphs from one experiment are showed.

### Matrigel plug assay

A Matrigel plug assay and hemoglobin measurements were performed to assess vessel formation in mice after their exposure to conditioned medium from transfected ovarian cancer cells. After transfecting A2780 cells with the miR-146a mimic, the cells were exposed to serum-free media for 48 h. The supernatant was then collected and centrifuged at 18,000 RCF for 15 min to remove the cells. The conditioned medium was then mixed with phenol-red-free Matrigel in a 2:3 ratio (total volume, 500 μL).

Nude mice were divided into four groups (four mice per group) and given subcutaneous injections of PBS, recombinant human vascular endothelial growth factor (rhVEGF 200 ng/mL), conditioned medium from A2780 cells transfected with miR-146a, or conditioned medium from A2780 cells transfected with miRctrl. After 7 days, the mice were sacrificed via cervical dislocation and the Matrigel plugs were harvested and examined for their hemoglobin content using the QuantiChrom hemoglobin assay kit (cat# DIHB-250, Bioassay Systems) per the manufacturer’s protocol. GraphPad 9.0 was used for the statistical analysis (an unpaired t-test) and graph production.

### Angiogenesis array

Ovarian cancer cells (OVCAR8 and A2780) were seeded in six-well plates and transfected with miR146a and miRctrl, as described above. After 48 h, conditioned medium from the two treatment groups was collected and centrifuged at 18,000 RCF for 15 min. The supernatant was collected and added to the array membranes (cat# ARY007, R&D Systems) according to the manufacturer’s instructions. After overnight incubation, the membranes were washed, and images of the protein blots were made using an Azure biosystem CCD camera 400. Image J 1.53 s (https://imagej.nih.gov/ij/download.html) was used to measure the optical densities of each dot relative to each protein. GraphPad 9.0 was used for the statistical analysis (a paired t-test) and graph production.

### RNA sequencing and data analysis

OVCAR8 and A2780 ovarian cancer cells and RF24 endothelial cells were transfected with miR146a and miRctrl, as described above. Whole RNA was isolated and miR146a expression was measured via RT-PCR, as described above. RNA samples were subjected to whole transcriptome sequencing by Novogene. Samples were sequenced in triplicate. After quality control, an RNA library was constructed and quantified. The quantified libraries were pooled and sequenced on Illumina platforms. Raw reads were first processed through in-house Perl scripts. The clean data proceeded to the downstream analysis step. Reference-genome and gene-model annotations were downloaded, and an index of the reference genome was built using Hisat2 v2.0.5 (https://benlangmead.github.io/aws-indexes/). Paired-end clean reads were then aligned to the reference genome. To count the read numbers mapped to each gene, featureCounts v1.5.0-p3 (https://subread.sourceforge.net/subread-package/) was used. The fragments per kilobase of transcript per million mapped fragments value for each gene was calculated. A differential expression analysis of the genes was performed using the DESeq2 R package (1.20.0) (https://bioconductor.org/packages/release/bioc/html/DESeq2.html), which uses the Benjamini and Hochberg approaches to control the false-discovery rate. Genes with an adjusted *P* value ≤ 0.05 were categorized as differentially expressed. ClusterProfiler software (https://bioconductor.org/packages/release/bioc/html/clusterProfiler.html) was used to test the statistical enrichment of the differentially expressed genes in the GO enrichment analysis, the KEGG pathways, the reactome pathway, the disease ontology pathway, and the DisGeNET pathway. Pathways with *P* values < 0.05 were considered to be significantly enriched by differentially expressed genes.

### Statistical analyses

For the in vitro experiments, the statistical analyses were performed using a two-tailed t-test; for in vivo experiments, the statistical analyses were performed using a two-tailed t-test and Mann–Whitney U test (for A2780 cells in vivo). Statistical significance was attributed if the *P* value was less than or equal to .05.

### Reporting summary

Further information on research design is available in the Nature Research Reporting Summary linked to this article.

### Supplementary information

supp files

REPORTING SUMMARY

## Data Availability

The authors confirm that the data supporting the findings of this study are available within the article and/or its supplementary materials. Additional supporting data are available upon request. Sequencing data are deposited in GEO, accession number PRJNA956523. All data needed to evaluate the conclusions in the paper are present in the paper or the Supplementary Materials; sequencing data are available on NCBI (BioProject PRJNA956523).

## Code availability

Graphical visualization for Fig. 2C was implemented using the R packages dplyr, stringr, and ggplot2. Codes are available upon request. A differential expression analysis of the genes was performed using the DESeq2 R package (1.20.0) (https://bioconductor.org/packages/release/bioc/html/DESeq2.html), which uses the Benjamini and Hochberg approaches to control the false-discovery rate. Genes with an adjusted *P* value ≤ 0.05 were categorized as differentially expressed. ClusterProfiler software (https://bioconductor.org/packages/release/bioc/html/clusterProfiler.html) was used to test the statistical enrichment of the differentially expressed genes in the GO enrichment analysis, the KEGG pathways, the reactome pathway, the disease ontology pathway, and the DisGeNET pathway. Pathways with *P* values < 0.05 were considered to be significantly enriched by differentially expressed genes. Sequencing data are deposited in GEO, accession n PRJNA956523.

## References

[1] Carthew RW, Sontheimer EJ (2009). Origins and Mechanisms of miRNAs and siRNAs. Cell.

[2] Mainini F, Eccles MR (2020). Lipid and Polymer-Based Nanoparticle siRNA Delivery Systems for Cancer Therapy. Molecules.

[3] Yadav S, Shekhawat M, Jahagirdar D, Kumar Sharma N (2017). Natural and artificial small RNAs: a promising avenue of nucleic acid therapeutics for cancer. Cancer Biol. Med..

[4] Zuckerman JE, Davis ME (2015). Clinical experiences with systemically administered siRNA-based therapeutics in cancer. Nat. Rev. Drug Discov..

[5] Blanco E, Shen H, Ferrari M (2015). Principles of nanoparticle design for overcoming biological barriers to drug delivery. Nat. Biotechnol..

[6] Howard KA, Kjems J (2007). Polycation-based nanoparticle delivery for improved RNA interference therapeutics. Expert Opin. Biol. Th.

[7] Wang Y, Li Z, Han Y, Liang LH, Ji A (2010). Nanoparticle-based delivery system for application of siRNA in vivo. Curr. Drug Metab..

[8] Raal FJ (2020). Inclisiran for the Treatment of Heterozygous Familial Hypercholesterolemia. N. Engl. J. Med.

[9] Coelho T (2013). Safety and Efficacy of RNAi Therapy for Transthyretin Amyloidosis. N. Engl. J. Med.

[10] Sercombe, L. et al. Advances and Challenges of Liposome Assisted Drug Delivery. *Front. Pharmacol*. **6**, 286 10.3389/fphar.2015.00286 (2015). 10.3389/fphar.2015.00286 PMC4664963 26648870

[11] Mathieu M, Martin-Jaular L, Lavieu G, Thery C (2019). Specificities of secretion and uptake of exosomes and other extracellular vesicles for cell-to-cell communication. Nat. Cell Biol..

[12] Babaker, M. A. et al. The Therapeutic Potential of Milk Extracellular Vesicles on Colorectal Cancer. *Int. J. Mol. Sci*. **23**, 10.3390/ijms23126812 (2022). 10.3390/ijms23126812 PMC9224713 35743255

[13] Zhang, Y. et al. Recent advances in exosome-mediated nucleic acid delivery for cancer therapy. *J. Nanobiotechnol.* **20**, 279 10.1186/s12951-022-01472-z (2022). 10.1186/s12951-022-01472-z PMC9194774 35701788

[14] Chen, H. Z. et al. Exosomes, a New Star for Targeted Delivery. *Front. Cell Dev. Biol*. **9**, 751079 10.3389/fcell.2021.751079 (2021). 10.3389/fcell.2021.751079 PMC8531489 34692704

[15] Yang, L., Huang, S. Q., Zhang, Z. R., Liu, Z. M. & Zhang, L. Roles and Applications of Red Blood Cell-Derived Extracellular Vesicles in Health and Diseases. *Int. J. Mol. Sci.* **23**, 5927 10.3390/ijms23115927 (2022). 10.3390/ijms23115927 PMC9180222 35682606

[16] Wang BM (2014). Targeted Drug Delivery to Intestinal Macrophages by Bioactive Nanovesicles Released from Grapefruit. Mol. Ther..

[17] Liu Y (2023). Enhancing oral delivery of plant-derived vesicles for colitis. J. Control Rel..

[18] Lian, M. Q. et al. Plant-derived extracellular vesicles: Recent advancements and current challenges on their use for biomedical applications. *J. Extracell Vesicles* **11**, e12283 10.1002/jev2.12283 (2022). 10.1002/jev2.12283 PMC9753580 36519808

[19] Yamasaki I (2012). Inhibitory effects of kale ingestion on metabolism by cytochrome P450 enzymes in rats. Biomed. Res. Tokyo.

[20] Schmidt LE, Dalhoff K (2002). Food-drug interactions. Drugs.

[21] Ashburner M (2000). Gene Ontology: tool for the unification of biology. Nat. Genet.

[22] Pettersson A (2000). Heterogeneity of the angiogenic response induced in different normal adult tissues by vascular permeability factor/vascular endothelial growth factor. Lab. Invest..

[23] Feng D, Nagy JA, Hipp J, Dvorak HF, Dvorak AM (1996). Vesiculo-vacuolar organelles and the regulation of venule permeability to macromolecules by vascular permeability factor, histamine, and serotonin. J. Exp. Med..

[24] Zhao TT (2013). CARMA3 overexpression accelerates cell proliferation and inhibits paclitaxel-induced apoptosis through NF-kappa B regulation in breast cancer cells. Tumor. Biol..

[25] Israel, L. et al. CARD10 cleavage by MALT1 restricts lung carcinoma growth in vivo. *Oncogenesis* **10**, 32 10.1038/s41389-021-00321-2 (2021). 10.1038/s41389-021-00321-2 PMC8024357 33824280

[26] Liu D, Evans I, Britton G, Zachary I (2008). The zinc-finger transcription factor, early growth response 3, mediates VEGF-induced angiogenesis. Oncogene.

[27] Rahman, F. A. & Krause, M. P. PAI-1, the Plasminogen System, and Skeletal Muscle. *Int. J. Mol. Sci.* **21**, 7066 10.3390/ijms21197066 (2020). 10.3390/ijms21197066 PMC7582753 32993026

[28] Yamin TT, Miller DK (1997). The interleukin-1 receptor-associated kinase is degraded by proteasomes following its phosphorylation. J. Biol. Chem..

[29] Swantek JL, Tsen MF, Cobb MH, Thomas JA (2000). IL-1 receptor-associated kinase modulates host responsiveness to endotoxin. J. Immunol..

[30] Seaberg J (2021). Hybrid Nanosystems for Biomedical Applications. Acs Nano.

[31] Krishnan, N., Fang, R. N. H. & Zhang, L. F. Engineering of stimuli-responsive self-assembled biomimetic nanoparticles. *Adv. Drug Deliver. Rev.* **179**, 114006 10.1016/j.addr.2021.114006 (2021). 10.1016/j.addr.2021.114006 34655662

[32] Hossen MN (2020). Switching the intracellular pathway and enhancing the therapeutic efficacy of small interfering RNA by auroliposome. Sci. Adv..

[33] Herrmann IK, Wood MJA, Fuhrmann G (2021). Extracellular vesicles as a next-generation drug delivery platform. Nat. Nanotechnol..

[34] Sutaria DS, Badawi M, Phelps MA, Schmittgen TD (2017). Achieving the Promise of Therapeutic Extracellular Vesicles: The Devil is in Details of Therapeutic Loading. Pharm. Res. Dordr..

[35] Johnsen KB (2018). On the use of liposome controls in studies investigating the clinical potential of extracellular vesicle-based drug delivery systems - A commentary. J. Control Rel..

[36] Tan, Z. L., Li, J. F., Luo, H. M., Liu, Y. Y. & Jin, Y. Plant extracellular vesicles: A novel bioactive nanoparticle for tumor therapy. *Front. Pharmacol*. **13**, 1006299 10.3389/fphar.2022.1006299 (2022). 10.3389/fphar.2022.1006299 PMC9559701 36249740

[37] Teng Y (2021). Plant-derived exosomal microRNAs inhibit lung inflammation induced by exosomes SARS-CoV-2 Nsp12. Mol. Ther..

[38] Tang Z (2020). Aptamer-conjugated and doxorubicin-loaded grapefruit-derived nanovectors for targeted therapy against HER2 breast cancer. J. Drug Target.

[39] Malik N (2000). Dendrimers: Relationship between structure and biocompatibility in vitro, and preliminary studies on the biodistribution of ^125^I-labelled polyamidoamine dendrimers in vivo. J. Control Rel..

[40] Caminade, A. M. Dendrimers, an Emerging Opportunity in Personalized Medicine? *J. Pers. Med.* **12**, 1334 10.3390/jpm12081334 (2022). 10.3390/jpm12081334 PMC9409959 36013283

[41] Wang HH (2019). Multiple roles of microRNA-146a in immune responses and hepatocellular carcinoma. Oncol. Lett..

[42] Lee WS, Yang H, Chon HJ, Kim C (2020). Combination of anti-angiogenic therapy and immune checkpoint blockade normalizes vascular-immune crosstalk to potentiate cancer immunity. Exp. Mol. Med..

[43] Welsh, J. A. et al. MIFlowCyt-EV: a framework for standardized reporting of extracellular vesicle flow cytometry experiments. *J. Extracell Vesicles* **9**, 1713526 10.1080/20013078.2020.1713526 (2020). 10.1080/20013078.2020.1713526 PMC7034442 32128070

[44] Motulsky, H. J. & Brown, R. E. Detecting outliers when fitting data with nonlinear regression - a new method based on robust nonlinear regression and the false discovery rate. *Bmc Bioinformatics* **7**, 123 10.1186/1471-2105-7-123 (2006). 10.1186/1471-2105-7-123 PMC1472692 16526949

