# Supplementary material for: Enhanced plant-derived vesicles for nucleotide delivery for cancer therapy
Source: NPJ Precis Oncol. 2024 Apr 6;8:86. doi: 10.1038/s41698-024-00556-3 (PMC10998889; doi:10.1038/s41698-024-00556-3)
Supplement: Supplementary file 1 — supp files [file 41698_2024_556_MOESM1_ESM.pdf]

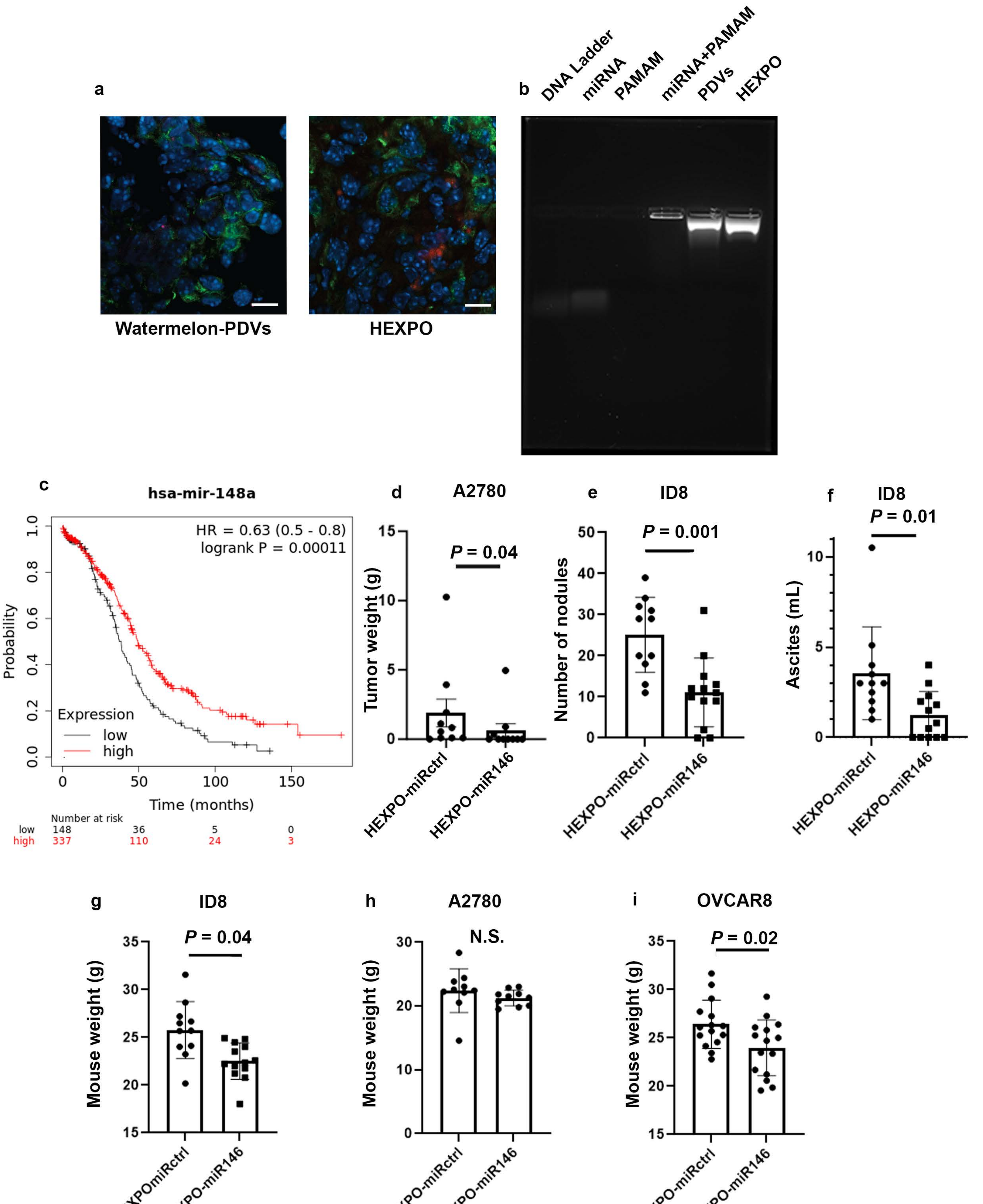

Supplementary Figure 1

Supplementary Figure 1: A) Watermelon-PDVs (fuchsia) and HEXPO (red) uptake from cytokeratin positive (green) tumor cells in ID8 tumor nodules. Scale bar= 10 µm. B) Electrophoresis retardation assay; C) Kaplan Meier curve representing survival in ovarian cancer patients with high and low tumor expression of miR148a (kmplotter.com); D) Quantification of tumor weight from the A2780 *in vivo* model treated with hybrid exosomal polymeric control microRNA (HEXPO-miRctrl) and hybrid exosomal polymeric miR146a (HEXPO-miR-146), without outliers exclusion (dots represent each sample, bars represent means, and whiskers the standard error of the mean) E) Quantification of tumor nodules from the ID8 *in vivo* model treated with hybrid exosomal polymeric control microRNA (HEXPO-miRctrl) and hybrid exosomal polymeric miR146a (HEXPO-miR-146); F) Quantification of ascites from the ID8 in vivo model; G) Quantification of body weight from the ID8 in vivo model; H) Quantification of body weight from the A2780 in vivo model in mice treated with HEXPO-ctrl *versus* HEXPO-miR146; I) Quantification of body weight from the OVCAR8 in vivo model in mice treated with HEXPO-ctrl *versus* HEXPO-miR146.

ABBREVIATIONS: miRctrl = mirna negative control; miR146 = mirna 146a.

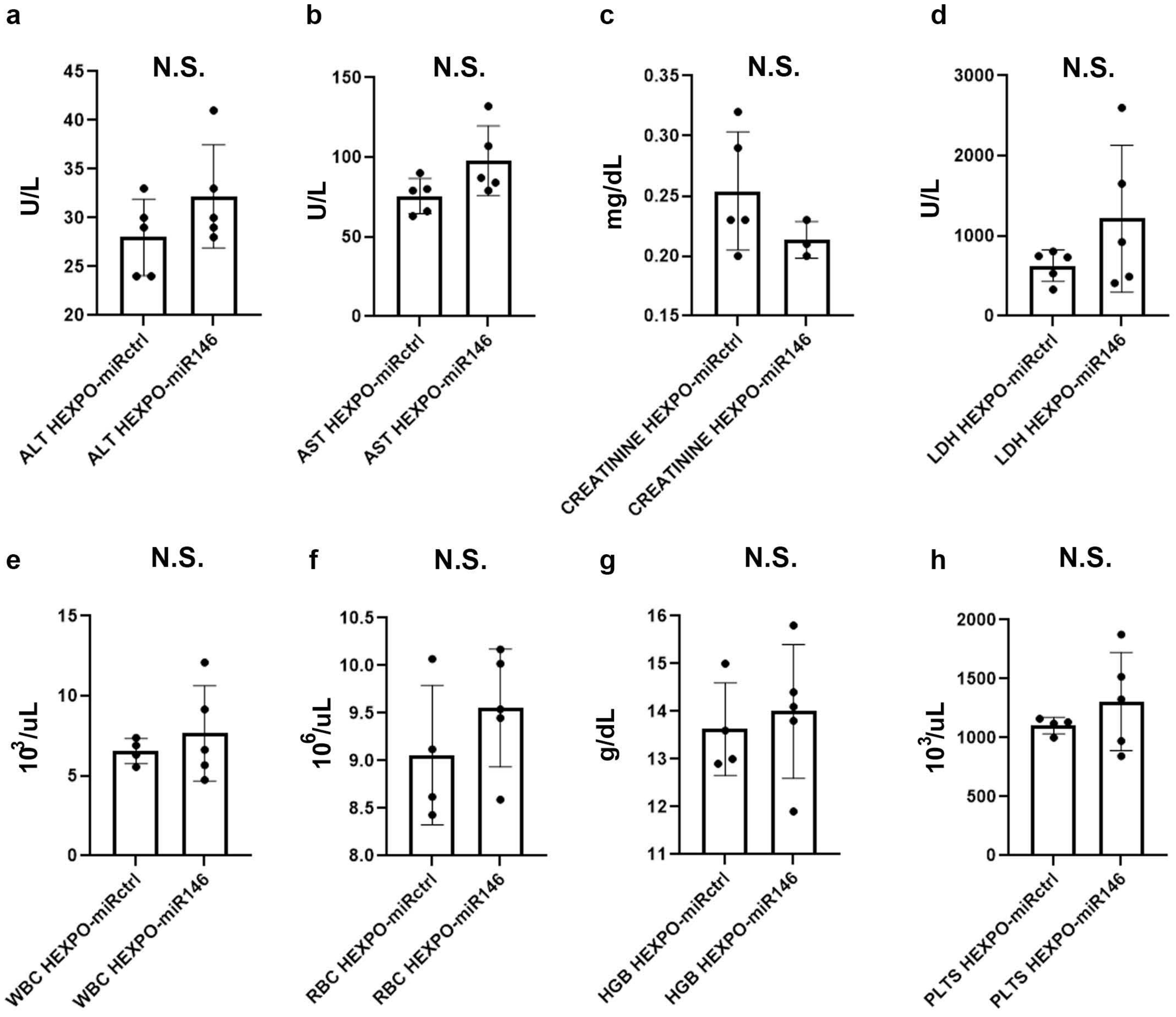

Supplementary Figure 2

Supplementary Figure 2: Serum levels of ALT (A), AST (B), creatinine (C), LDH (D), WBC (E), RBC (F), HBG (G), PLTS (H) in mouse treated with HEXPO-ctrl *versus* HEXPO-miR146 in the OVCAR8 in vivo model.

ABBREVIATIONS: miRctrl = mirna negative control; miR146 = mirna 146a; ALT = serum alanine transaminase; AST = Serum aspartate transaminase; LDH = lactate dehydrogenase; WBC = white blood cells; RBC = red blood cells; HBG = hemoglobin; PLTS = platelets.

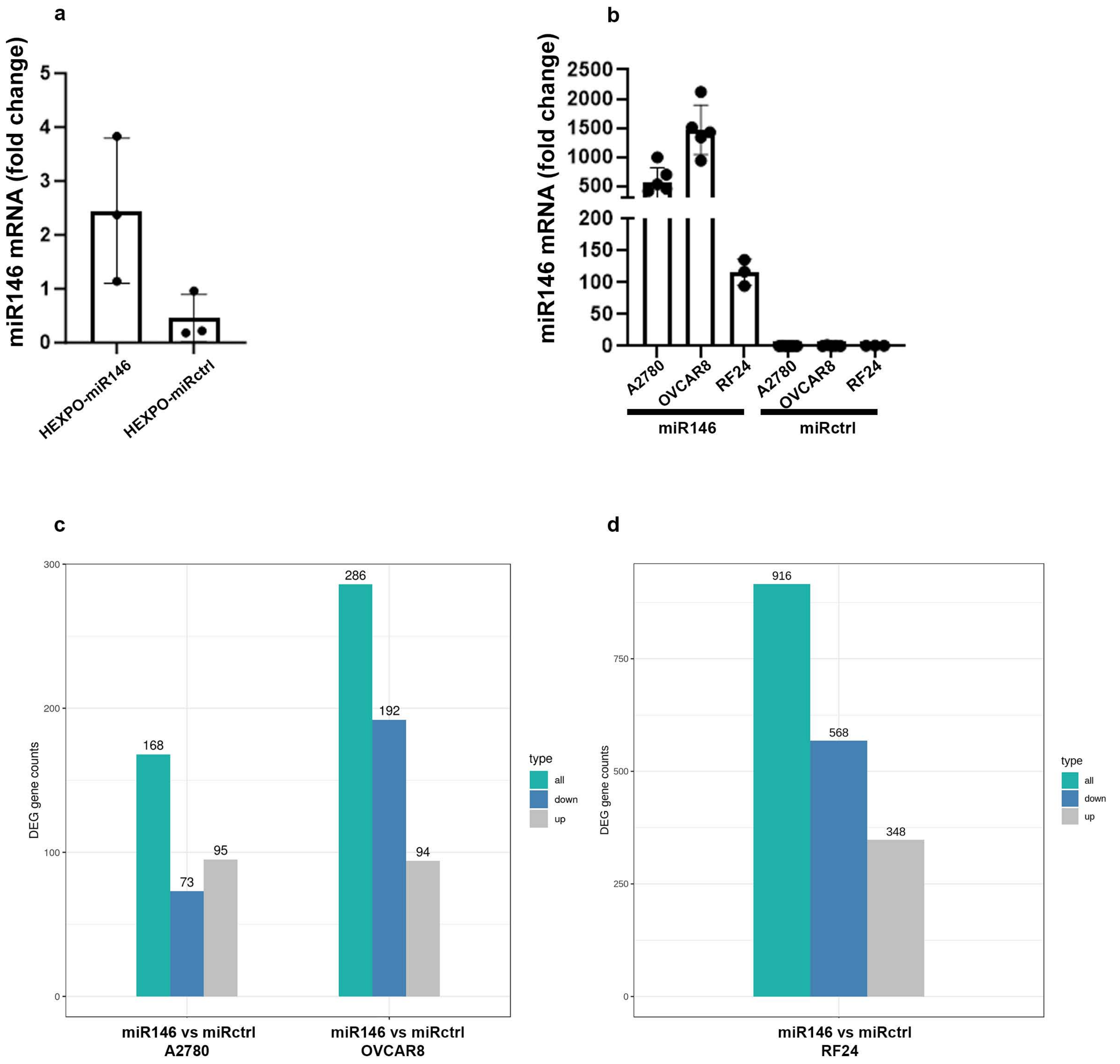

Supplementary figure 3

Supplementary Figure 3: A) miR146 expression in tumors from ID8 mice treated with HEXPO-miRctrl or HEXPO-miR146; B) miR146 expression in A2780, OVCAR8 and RF24 cells transfected with miR146 or miRctrl; C) DEG counts of all, downregulated and upregulated genes in A2780 and OVCAR8 cells transfected with miR146 versus A2780 and OVCAR8 cells transfected with miRctrl; D) DEG counts of all, downregulated and upregulated genes in RF24 cells transfected with miR146 versus RF24 cells transfected with miRctrl. MiR146 expression is calculated as fold change over housekeeping gene. Bars in histograms represent mean values and whiskers represent standard deviation from the mean.

ABBREVIATIONS: miRctrl = mirna negative control; miR146 = mirna 146a, DEG= differentially expressed genes.

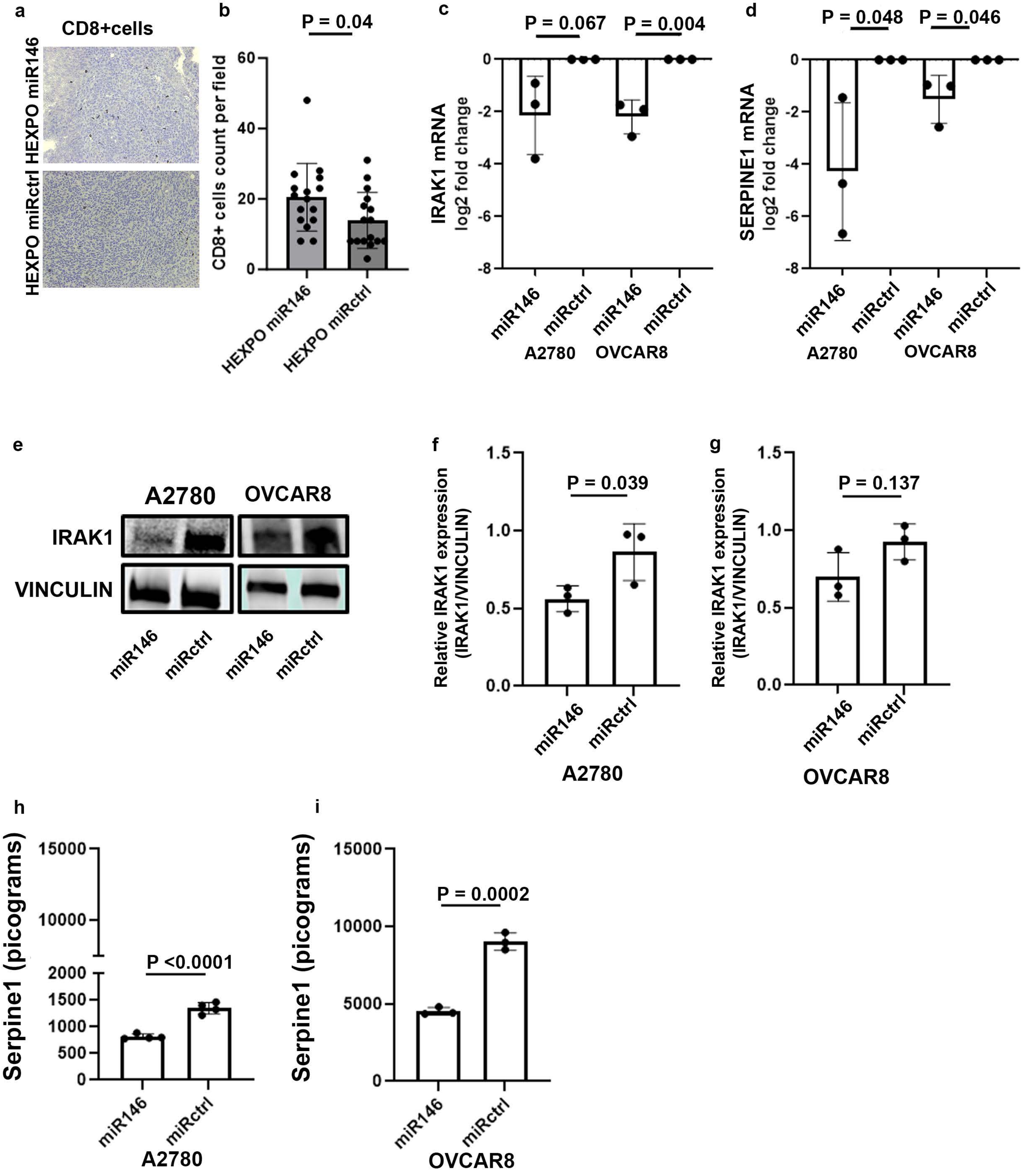

Supplementary Figure 4

Supplementary Figure 4: A) representative pictures from 20X magnification quadrants of tumor sections from the ID8 mouse model treated with HEXPO-ctrl and HEXPO-miR146 stained with anti-CD8 antibody, and quantification of CD8-positive cells in B); C) IRAK1 mRNA differential expression in $\log_2$ fold change between miR146 and miRctrl treated A2780 and OVCAR8 cells; D) SERPINE1 mRNA differential expression in log2 fold change between miR146 and miRctrl treated A2780 and OVCAR8 cells; E, F, and G) Western blot with quantification showing IRAK1 protein expression in miR146 and miRctrl treated A2780 and OVCAR8 cells normalized to vimentin (the pale blue background is an artifact from the imaging software); H and I) Quantification of ELISA assay showing SERPINE1 protein expression in the supernatant from miR146 and miRctrl treated A2780 and OVCAR8 cells.

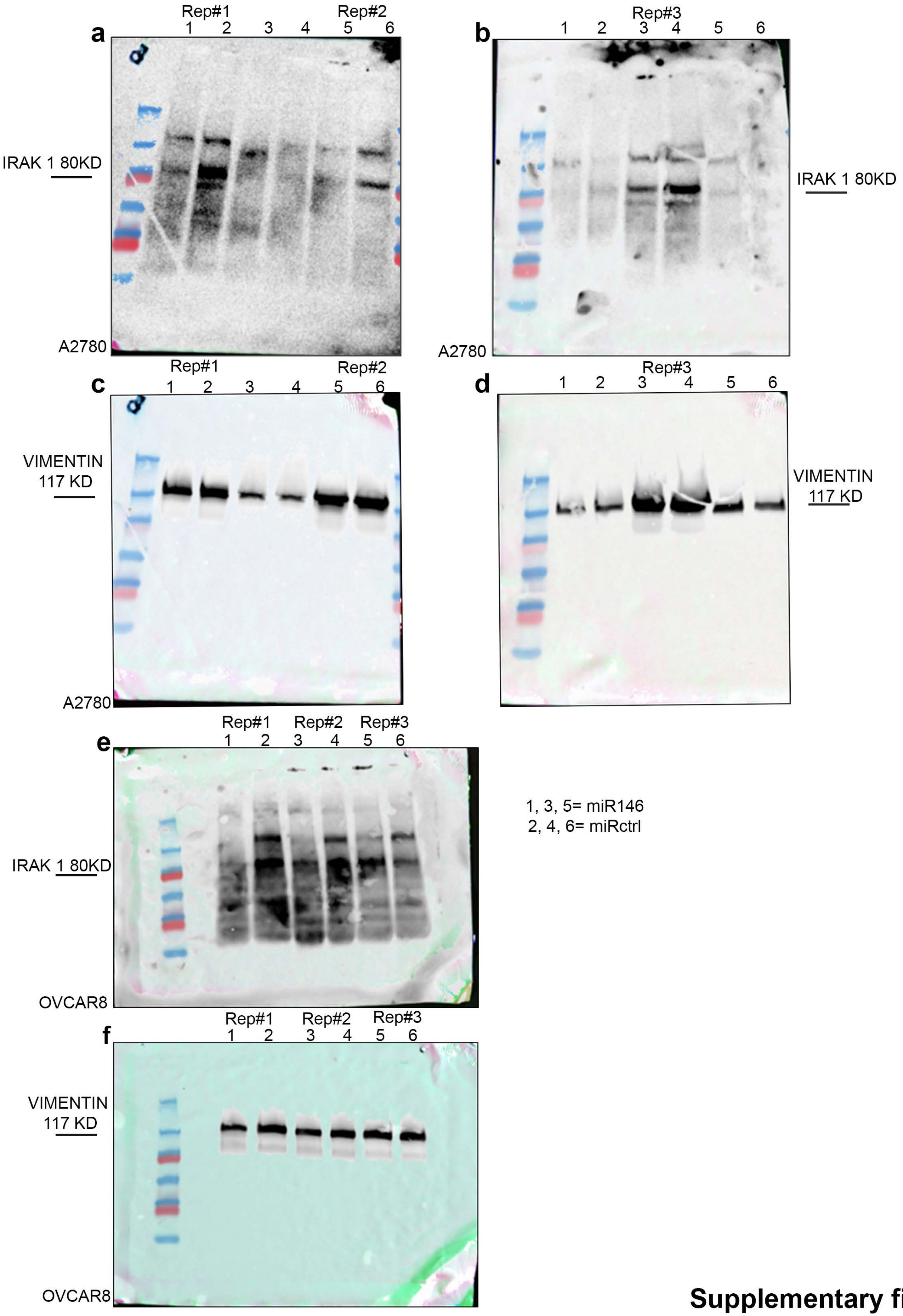

Supplementary figure 5

Supplementary Figure 5: Original acquired blots from Western Blot analysis (three experimental triplicates) of IRAK1 protein expression in A2780 cells (A and B), and OVCAR8 cells (E), and VINCULIN as housekeeping protein (C, D and F). Rep= replicate

Database	Pathway	Evidence	Hits	Expected hits	P value
Gene Ontology - Biological Process	RNA metabolic process	predicted (intersection)	963	814.2	3.90e-8
Gene Ontology - Biological Process	RNA metabolic process	predicted (intersection)	810	670.2	3.90e-8
Gene Ontology - Biological Process	regulation of gene expression	predicted (intersection)	936	785.7	3.90e-8
Gene Ontology - Biological Process	transcription, DNA-templated	predicted (intersection)	788	650.5	3.90e-8
Gene Ontology - Biological Process	transcription by RNA polymerase II	predicted (intersection)	627	505.4	9.25e-8
Gene Ontology - Biological Process	RNA biosynthetic process	predicted (intersection)	798	663.9	1.02e-7
Gene Ontology - Biological Process	transcription, DNA-templated	predicted (intersection)	745	616.3	1.50e-7
Gene Ontology - Biological Process	RNA biosynthetic process	predicted (intersection)	758	632.1	3.54e-7
Gene Ontology - Biological Process	nucleic acid-templated transcription	predicted (intersection)	757	630.6	3.54e-7
Gene Ontology - Biological Process	transcription by RNA polymerase II	predicted (intersection)	589	476.3	3.54e-7
Gene Ontology - Biological Process	regulation of cellular metabolic	predicted (intersection)	679	562	9.12e-7
Gene Ontology - Biological Process	positive regulation of metabolic process	predicted (intersection)	732	615.3	2.96e-6
Gene Ontology - Biological Process	positive regulation of transcription, DNA-templated	predicted (intersection)	337	260.4	1.93e-5
Gene Ontology - Biological Process	anatomical structure morphogenesis	predicted (union)	989	867.6	2.30e-5

Gene Ontology - Biological Process	positive regulation of RNA biosynthetic process	predicted (union)	627	530.2	2.30e-5
Gene Ontology - Biological Process	positive regulation of nucleic acid-templated transcription	predicted (union)	627	529.8	2.30e-5
Gene Ontology - Biological Process	positive regulation of transcription, DNA-templated	predicted (union)	589	493.6	2.30e-5
Gene Ontology - Biological Process	nervous system development	predicted (union)	897	784.5	2.35e-5
Gene Ontology - Biological Process	positive regulation of gene expression	predicted (union)	745	641	2.35e-5
Gene Ontology - Biological Process	positive regulation of macromolecule biosynthetic process	predicted (union)	719	617.6	2.35e-5
Gene Ontology - Biological Process	positive regulation of transcription by RNA polymerase II	predicted (union)	454	372.8	2.35e-5
Gene Ontology - Biological Process	positive regulation of gene expression	predicted (intersection)	423	338.1	2.52e-5
Gene Ontology - Biological Process	negative regulation of transcription by RNA polymerase II	predicted (union)	336	267.5	2.52e-5
Gene Ontology - Biological Process	regulation of cell differentiation	predicted (union)	706	606.5	2.54e-5

Gene Ontology - Biological Process	negative regulation of cellular metabolic process	predicted (intersection)	524	430.8	2.78e-5
Gene Ontology - Biological Process	positive regulation of RNA biosynthetic process	predicted (intersection)	357	279.7	2.78e-5
Gene Ontology - Biological Process	positive regulation of nucleic acid-templated transcription	predicted (intersection)	357	279.4	2.78e-5
Gene Ontology - Biological Process	positive regulation of transcription by RNA polymerase II	predicted (intersection)	262	196.6	3.63e-5
Gene Ontology - Biological Process	positive regulation of cellular biosynthetic process	predicted (union)	747	646.2	3.66e-5
Gene Ontology - Biological Process	positive regulation of macromolecule biosynthetic process	predicted (intersection)	407	325.8	4.01e-5
Gene Ontology - Biological Process	anatomical structure morphogenesis	predicted (intersection)	551	457.6	4.25e-5
Gene Ontology - Biological Process	positive regulation of cellular biosynthetic process	predicted (intersection)	421	340.9	8.64e-5
Gene Ontology - Biological Process	cellular protein modification process	predicted (intersection)	824	717.2	9.49e-5
Gene Ontology - Biological Process	nervous system development	predicted (intersection)	500	413.8	9.49e-5

Gene Ontology - Biological Process	protein modification process	predicted (intersection)	824	717.2	9.49e-5
Gene Ontology - Biological Process	urogenital system development	predicted (intersection)	99	62.26	9.49e-5
Gene Ontology - Biological Process	tube development	predicted (union)	432	358.1	1.08e-4
Gene Ontology - Biological Process	negative regulation of cellular metabolic process	predicted (union)	920	816.7	2.49e-4
Gene Ontology - Biological Process	tube morphogenesis	predicted (union)	352	289.3	4.82e-4
Gene Ontology - Biological Process	intracellular signal transduction	predicted (union)	985	882.7	5.97e-4
Gene Ontology - Biological Process	enzyme linked receptor protein signaling pathway	predicted (intersection)	233	178.2	5.98e-4
Gene Ontology - Biological Process	regulation of cell differentiation	predicted (intersection)	388	319.9	0.001
Gene Ontology - Biological Process	tube development	predicted (intersection)	243	188.9	0.001
Gene Ontology - Biological Process	enzyme linked receptor protein signaling pathway	predicted (union)	402	337.8	0.001
Gene Ontology - Biological Process	heart development	predicted (union)	240	191.2	0.001
Gene Ontology - Biological Process	negative regulation of nucleic acid-templated transcription	predicted (union)	463	394.6	0.001

Gene Ontology - Biological Process	negative regulation of transcription, DNA-templated	predicted (union)	441	373.6	0.001
Gene Ontology - Biological Process	cellular protein metabolic process	predicted (intersection)	988	888.4	0.002
Gene Ontology - Biological Process	response to growth factor	predicted (intersection)	169	125.2	0.002
Gene Ontology - Biological Process	muscle structure development	predicted (union)	266	216.6	0.002
Gene Ontology - Biological Process	negative regulation of RNA biosynthetic process	predicted (union)	463	395.4	0.002
Gene Ontology - Biological Process	negative regulation of biosynthetic process	predicted (union)	571	496	0.002
Gene Ontology - Biological Process	protein phosphorylation	predicted (union)	732	649.4	0.002
Gene Ontology - Biological Process	regulation of cellular component organization	predicted (union)	893	802.4	0.002
Gene Ontology - Biological Process	regulation of neurogenesis	predicted (union)	322	267.1	0.002
Gene Ontology - Biological Process	head development	predicted (intersection)	184	139.6	0.003
Gene Ontology - Biological Process	regulation of cellular component organization	predicted (intersection)	497	423.3	0.003
Gene Ontology - Biological Process	renal system development	predicted (intersection)	85	55.76	0.003
Gene Ontology - Biological Process	cell development	predicted (union)	770	687.1	0.003

Gene Ontology - Biological Process	brain development	predicted (intersection)	174	131.7	0.004
Gene Ontology - Biological Process	cell development	predicted (intersection)	429	362.5	0.004
Gene Ontology - Biological Process	cell migration	predicted (intersection)	296	240.7	0.004
Gene Ontology - Biological Process	kidney development	predicted (intersection)	81	53.04	0.004
Gene Ontology - Biological Process	negative regulation of transcription by RNA polymerase II	predicted (intersection)	185	141.1	0.004
Gene Ontology - Biological Process	tube morphogenesis	predicted (intersection)	198	152.6	0.004
Gene Ontology - Biological Process	negative regulation of gene expression	predicted (union)	668	591.4	0.004
Gene Ontology - Biological Process	negative regulation of gene expression	predicted (intersection)	373	311.9	0.005
Gene Ontology - Biological Process	phosphate-containing compound metabolic process	predicted (intersection)	650	571.5	0.005
Gene Ontology - Biological Process	dephosphorylation	predicted (union)	205	163.7	0.005
Gene Ontology - Biological Process	cellular response to growth factor stimulus	predicted (intersection)	159	119.7	0.006
Gene Ontology - Biological Process	neurogenesis	predicted (union)	596	525.4	0.006
Gene Ontology - Biological Process	negative regulation of biosynthetic process	predicted (intersection)	317	261.6	0.007

Gene Ontology - Biological Process	generation of neurons	predicted (union)	561	493.6	0.007
Gene Ontology - Biological Process	phosphorylation	predicted (union)	875	791.7	0.007
Gene Ontology - Biological Process	positive regulation of autophagy	predicted (union)	62	41.33	0.007
Gene Ontology - Biological Process	positive regulation of cell differentiation	predicted (union)	385	328.7	0.007
Gene Ontology - Biological Process	protein modification by small protein conjugation	predicted (union)	363	308.8	0.007
Gene Ontology - Biological Process	stem cell population maintenance	predicted (union)	82	57.63	0.007
Gene Ontology - Biological Process	regulation of phosphorus metabolic process	predicted (union)	645	573.9	0.008
Gene+2:81 Ontology - Biological Process	central nervous system development	predicted (intersection)	222	176.7	0.010
Gene Ontology - Biological Process	circadian rhythm	predicted (intersection)	60	37.94	0.011
Gene Ontology - Biological Process	muscle structure development	predicted (intersection)	151	114.3	0.011
Gene Ontology - Biological Process	immune system development	predicted (union)	379	325.5	0.011
Gene Ontology - Biological Process	regulation of chromatin organization	predicted (union)	84	60.01	0.011
Gene Ontology - Biological Process	regulation of muscle organ development	predicted (union)	68	46.9	0.011
Gene Ontology - Biological Process	vasculature development	predicted (union)	292	245.2	0.011

Gene Ontology - Biological Process	cell migration	predicted (union)	518	456.2	0.012
Gene Ontology - Biological Process	regulation of cellular protein metabolic process	predicted (union)	927	845.7	0.012
Gene Ontology - Biological Process	response to growth factor	predicted (union)	283	237.3	0.012
Gene Ontology - Biological Process	protein phosphorylation	predicted (intersection)	402	342.5	0.013
Gene Ontology - Biological Process	cellular response to growth factor stimulus	predicted (union)	271	226.9	0.014
Gene Ontology - Biological Process	organophosphate metabolic process	predicted (union)	394	340.6	0.014
Gene Ontology - Biological Process	protein K48-linked ubiquitination	predicted (union)	34	20.27	0.014
Gene Ontology - Biological Process	cell surface receptor signaling pathway	predicted (intersection)	586	516.1	0.015
Gene Ontology - Biological Process	blood vessel development	predicted (union)	280	235.7	0.015
Gene Ontology - Biological Process	cardiovascular system development	predicted (union)	294	248.4	0.015
Gene Ontology - Biological Process	urogenital system development	predicted (union)	150	118	0.015
Gene Ontology - Biological Process	blood vessel morphogenesis	predicted (union)	248	206.7	0.016
Gene Ontology - Biological Process	cell projection morphogenesis	predicted (union)	242	201.1	0.016
Gene Ontology - Biological Process	lipid phosphorylation	predicted (union)	57	38.55	0.016

Gene Ontology - Biological Process	locomotion	predicted (union)	650	583.4	0.016
Gene Ontology - Biological Process	regulation of cellular response to stress	predicted (union)	293	248	0.016
Gene Ontology - Biological Process	regulation of intracellular signal transduction	predicted (union)	675	607.3	0.016
Gene Ontology - Biological Process	positive regulation of neuron apoptotic process	predicted (intersection)	23	10.9	0.017
Gene Ontology - Biological Process	regulation of neurogenesis	predicted (intersection)	180	140.9	0.017
Gene Ontology - Biological Process	coenzyme biosynthetic process	predicted (union)	66	46.1	0.018
Gene Ontology - Biological Process	anatomical structure formation involved in morphogenesis	predicted (intersection)	242	197.1	0.019
Gene Ontology - Biological Process	negative regulation of transcription, DNA-templated	predicted (intersection)	242	197.1	0.019
Gene Ontology - Biological Process	regulation of chromatin organization	predicted (intersection)	51	31.66	0.019
Gene Ontology - Biological Process	cell adhesion	predicted (union)	531	471.3	0.019
Gene Ontology - Biological Process	proteoglycan metabolic process	predicted (intersection)	30	15.93	0.020
Gene Ontology - Biological Process	regulation of cell migration	predicted (intersection)	187	147.8	0.020

Gene Ontology - Biological Process	transmembrane receptor protein tyrosine kinase signaling pathway	predicted (intersection)	160	123.9	0.020
Gene Ontology - Biological Process	locomotion	predicted (intersection)	362	307.7	0.021
Gene Ontology - Biological Process	postsynapse organization	predicted (intersection)	48	29.56	0.021
Gene Ontology - Biological Process	synapse assembly	predicted (intersection)	51	31.86	0.021
Gene Ontology - Biological Process	regulation of DNA-templated transcription, initiation	predicted (union)	21	11.13	0.021
Gene Ontology - Biological Process	regulation of histone modification	predicted (union)	70	49.68	0.021
Gene Ontology - Biological Process	cell part morphogenesis	predicted (union)	247	207.1	0.022
Gene Ontology - Biological Process	protein ubiquitination	predicted (union)	325	279	0.022
Gene Ontology - Biological Process	regulation of lipid metabolic process	predicted (union)	169	136.3	0.022
Gene Ontology - Biological Process	regulation of osteoclast differentiation	predicted (union)	37	23.05	0.022
Gene Ontology - Biological Process	cellular response to organonitrogen compound	predicted (intersection)	135	102.5	0.023
Gene Ontology - Biological Process	hair follicle development	predicted (intersection)	30	16.14	0.023

Gene Ontology - Biological Process	anatomical structure formation involved in morphogenesis	predicted (union)	426	373.6	0.023
Gene Ontology - Biological Process	transmembrane receptor protein tyrosine kinase signaling pathway	predicted (union)	277	234.9	0.023
Gene Ontology - Biological Process	positive regulation of protein modification process	predicted (union)	447	393.8	0.026
Gene Ontology - Biological Process	cell motility	predicted (intersection)	316	266.7	0.027
Gene Ontology - Biological Process	regulation of cell motility	predicted (intersection)	198	158.9	0.027
Gene Ontology - Biological Process	regulation of cellular protein metabolic process	predicted (intersection)	508	446.1	0.027
Gene Ontology - Biological Process	vasculature development	predicted (intersection)	165	129.3	0.027
Gene Ontology - Biological Process	blood vessel development	predicted (intersection)	159	124.3	0.028
Gene Ontology - Biological Process	generation of neurons	predicted (intersection)	309	260.4	0.028
Gene Ontology - Biological Process	negative regulation of nucleic acid-templated transcription	predicted (intersection)	252	208.2	0.028

Gene Ontology - Biological Process	negative regulation of transcription from RNA polymerase II promoter in response to stress	predicted (intersection)	8	2.306	0.028
Gene Ontology - Biological Process	neurogenesis	predicted (intersection)	327	277.1	0.028
Gene Ontology - Biological Process	regulation of cellular component movement	predicted (intersection)	214	173.6	0.028
Gene Ontology - Biological Process	regulation of protein metabolic process	predicted (union)	978	901.8	0.028
Gene Ontology - Biological Process	heart development	predicted (intersection)	132	100.8	0.029
Gene Ontology - Biological Process	negative regulation of RNA biosynthetic process	predicted (intersection)	252	208.6	0.029
Gene Ontology - Biological Process	regulation of organelle organization	predicted (intersection)	260	215.9	0.029
Gene Ontology - Biological Process	retina vasculature development in camera-type eye	predicted (intersection)	10	3.354	0.029
Gene Ontology - Biological Process	rhythmic process	predicted (intersection)	79	55.34	0.029
Gene Ontology - Biological Process	striated muscle tissue development	predicted (union)	158	127.2	0.029
Gene Ontology - Biological Process	cardiovascular system development	predicted (intersection)	166	131	0.030
Gene Ontology - Biological Process	hair cycle process	predicted (intersection)	30	16.56	0.030

Gene Ontology - Biological Process	regulation of glycoprotein biosynthetic process	predicted (intersection)	16	6.918	0.030
Gene Ontology - Biological Process	skin epidermis development	predicted (intersection)	30	16.56	0.030
Gene Ontology - Biological Process	regulation of striated muscle tissue development	predicted (union)	64	45.31	0.030
Gene Ontology - Biological Process	cell motility	predicted (union)	564	505.5	0.031
Gene Ontology - Biological Process	muscle cell differentiation	predicted (union)	144	114.9	0.031
Gene Ontology - Biological Process	regulation of locomotion	predicted (union)	374	326.3	0.031
Gene Ontology - Biological Process	regulation of neuron apoptotic process	predicted (intersection)	59	39.2	0.032
Gene Ontology - Biological Process	covalent chromatin modification	predicted (union)	188	154.6	0.032
Gene Ontology - Biological Process	positive regulation of signal transduction	predicted (union)	614	553.6	0.033
Gene Ontology - Biological Process	muscle tissue development	predicted (union)	163	132.3	0.034
Gene Ontology - Biological Process	positive regulation of protein kinase B signaling	predicted (union)	81	60.01	0.034
Gene Ontology - Biological Process	regulation of JNK cascade	predicted (union)	82	60.81	0.034
Gene Ontology - Biological Process	regulation of phosphorylation	predicted (union)	564	506.3	0.034

Gene Ontology - Biological Process	tissue morphogenesis	predicted (union)	264	224.5	0.034
Gene Ontology - Biological Process	phosphorylation	predicted (intersection)	475	417.6	0.035
Gene Ontology - Biological Process	positive regulation of cellular protein metabolic process	predicted (intersection)	317	269.6	0.035
Gene Ontology - Biological Process	positive regulation of glycoprotein biosynthetic process	predicted (intersection)	11	3.983	0.035
Gene Ontology - Biological Process	positive regulation of protein modification process	predicted (intersection)	250	207.8	0.035
Gene Ontology - Biological Process	regulation of locomotion	predicted (intersection)	211	172.1	0.035
Gene Ontology - Biological Process	neuron projection morphogenesis	predicted (union)	231	194.3	0.035
Gene Ontology - Biological Process	glycoprotein metabolic process	predicted (intersection)	95	69.6	0.036
Gene Ontology - Biological Process	JNK cascade	predicted (union)	95	72.33	0.036
Gene Ontology - Biological Process	cell-cell signaling	predicted (union)	593	534.5	0.036
Gene Ontology - Biological Process	macroautophagy	predicted (union)	124	97.77	0.036
Gene Ontology - Biological Process	morphogenesis of an epithelium	predicted (union)	223	187.2	0.036
Gene Ontology - Biological Process	phosphatidylinositol metabolic process	predicted (union)	95	72.33	0.036

Gene Ontology - Biological Process	regulation of neuron differentiation	predicted (union)	249	211	0.036
Gene Ontology - Biological Process	regulation of organelle organization	predicted (union)	461	409.3	0.036
Gene Ontology - Biological Process	lung alveolus development	predicted (intersection)	18	8.386	0.037
Gene Ontology - Biological Process	neuron apoptotic process	predicted (intersection)	65	44.44	0.037
Gene Ontology - Biological Process	animal organ morphogenesis	predicted (union)	400	352.1	0.037
Gene Ontology - Biological Process	cell morphogenesis	predicted (union)	353	308	0.037
Gene Ontology - Biological Process	cellular lipid metabolic process	predicted (union)	390	343	0.038
Gene Ontology - Biological Process	regulation of cell migration	predicted (union)	323	280.2	0.038
Gene Ontology - Biological Process	regulation of dendritic spine development	predicted (union)	36	23.05	0.038
Gene Ontology - Biological Process	cell morphogenesis involved in differentiation	predicted (union)	262	223.8	0.040
Gene Ontology - Biological Process	gland development	predicted (union)	185	153	0.040
Gene Ontology - Biological Process	hippo signaling	predicted (union)	24	13.91	0.040
Gene Ontology - Biological Process	regulation of cellular component movement	predicted (union)	375	329.1	0.040
Gene Ontology - Biological Process	muscle cell proliferation	predicted (intersection)	55	36.48	0.042

Gene Ontology - Biological Process	circadian rhythm	predicted (union)	94	71.93	0.042
Gene Ontology - Biological Process	regulation of cell motility	predicted (union)	345	301.2	0.042
Gene Ontology - Biological Process	gene silencing	predicted (intersection)	57	38.15	0.043
Gene Ontology - Biological Process	neuron development	predicted (union)	399	352.1	0.043
Gene Ontology - Biological Process	polysaccharide metabolic process	predicted (union)	51	35.37	0.043
Gene Ontology - Biological Process	regulation of transcription initiation from RNA polymerase II promoter	predicted (union)	15	7.551	0.043
Gene Ontology - Biological Process	actin filament-based process	predicted (intersection)	154	121.8	0.044
Gene Ontology - Biological Process	cell-cell signaling	predicted (intersection)	329	282	0.045
Gene Ontology - Biological Process	cell projection organization	predicted (union)	550	496	0.045
Gene Ontology - Biological Process	dendritic spine development	predicted (union)	46	31.4	0.045
Gene Ontology - Biological Process	embryonic morphogenesis	predicted (union)	238	202.3	0.045
Gene Ontology - Biological Process	glycerolipid metabolic process	predicted (union)	180	149	0.045
Gene Ontology - Biological Process	hemopoiesis	predicted (union)	334	291.7	0.045
Gene Ontology - Biological Process	kidney development	predicted (union)	126	100.5	0.045
Gene Ontology - Biological Process	neuron differentiation	predicted (union)	491	439.9	0.045

Gene Ontology - Biological Process	neuron projection development	predicted (union)	352	308.4	0.045
Gene Ontology - Biological Process	phosphatidylinositol phosphorylation	predicted (union)	47	32.19	0.045
Gene Ontology - Biological Process	regulation of circadian rhythm	predicted (union)	57	40.54	0.045
Gene Ontology - Biological Process	regulation of neuron projection development	predicted (union)	190	158.2	0.045
Gene Ontology - Biological Process	renal system development	predicted (union)	132	105.7	0.045
Gene Ontology - Biological Process	trigeminal nerve development	predicted (union)	10	4.372	0.045
Gene Ontology - Biological Process	RNA splicing	predicted (union)	173	143.1	0.046
Gene Ontology - Biological Process	cellular response to organic substance	predicted (union)	950	881.1	0.046
Gene Ontology - Biological Process	histone modification	predicted (union)	180	149.4	0.046
Gene Ontology - Biological Process	positive regulation of dendrite development	predicted (union)	36	23.45	0.046
Gene Ontology - Biological Process	regulation of actin filament-based process	predicted (union)	144	116.8	0.046
Gene Ontology - Biological Process	regulation of developmental growth	predicted (union)	132	106.1	0.046
Gene Ontology - Biological Process	regulation of kinase activity	predicted (union)	327	285.4	0.046
Gene Ontology - Biological Process	cell projection morphogenesis	predicted (intersection)	136	106.1	0.047

Gene Ontology - Biological Process	glomerulus development	predicted (intersection)	22	11.32	0.047
Gene Ontology - Biological Process	organelle organization	predicted (intersection)	691	625.8	0.047
Gene Ontology - Biological Process	positive regulation of smooth muscle cell proliferation	predicted (intersection)	29	16.35	0.047
Gene Ontology - Biological Process	regulation of protein metabolic process	predicted (intersection)	534	475.7	0.047
Gene Ontology - Biological Process	sensory system development	predicted (union)	154	126	0.048
Gene Ontology - Biological Process	glycoprotein biosynthetic process	predicted (intersection)	79	56.81	0.049
Gene Ontology - Biological Process	response to radiation	predicted (intersection)	109	82.6	0.049
Gene Ontology - Biological Process	sig	predicted (intersection)	139	109	0.050
Gene Ontology - Biological Process	carbohydrate derivative metabolic process	predicted (intersection)	235	195.8	0.050
Gene Ontology - Biological Process	homeostatic process	predicted (union)	650	592.6	0.050
Gene Ontology - Biological Process	peptidyl-serine phosphorylation	predicted (union)	105	82.27	0.050
Gene Ontology - Cellular Component	nucleoplasm	predicted (intersection)	766	637.3	2.01e-7
Gene Ontology - Cellular Component	chromosome	predicted (union)	950	820.3	2.36e-7
Gene Ontology - Cellular Component	nuclear chromatin	predicted (union)	738	624	2.36e-7

Gene Ontology - Cellular Component	chromatin	predicted (union)	787	671.3	3.01e-7
Gene Ontology - Cellular Component	nuclear chromosome	predicted (union)	792	678	4.60e-7
Gene Ontology - Cellular Component	chromatin	predicted (intersection)	427	354.1	7.56e-4
Gene Ontology - Cellular Component	early endosome	predicted (intersection)	94	61.63	7.56e-4
Gene Ontology - Cellular Component	nuclear chromatin	predicted (intersection)	397	329.1	7.56e-4
Gene Ontology - Cellular Component	postsynapse	predicted (intersection)	154	111.1	7.56e-4
Gene Ontology - Cellular Component	postsynaptic membrane	predicted (intersection)	88	57.23	7.56e-4
Gene Ontology - Cellular Component	synapse	predicted (intersection)	270	213.6	7.56e-4
Gene Ontology - Cellular Component	synaptic membrane	predicted (intersection)	112	75.89	7.56e-4
Gene Ontology - Cellular Component	chromosome	predicted (intersection)	505	432.7	0.002
Gene Ontology - Cellular Component	postsynaptic specialization	predicted (intersection)	88	59.12	0.002
Gene Ontology - Cellular Component	Golgi membrane	predicted (union)	301	249.2	0.002
Gene Ontology - Cellular Component	integral component of synaptic membrane	predicted (intersection)	49	28.72	0.003
Gene Ontology - Cellular Component	nuclear chromosome	predicted (intersection)	420	357.6	0.004
Gene Ontology - Cellular Component	Golgi apparatus	predicted (intersection)	326	271.7	0.005

Gene Ontology - Cellular Component	guanyl-nucleotide exchange factor complex	predicted (intersection)	8	2.096	0.005
Gene Ontology - Cellular Component	postsynaptic density	predicted (intersection)	80	54.09	0.005
Gene Ontology - Cellular Component	asymmetric synapse	predicted (intersection)	80	54.92	0.007
Gene Ontology - Cellular Component	cis-Golgi network	predicted (intersection)	18	7.757	0.007
Gene Ontology - Cellular Component	dendritic tree	predicted (intersection)	127	95.18	0.008
Gene Ontology - Cellular Component	nuclear body	predicted (union)	314	265.5	0.008
Gene Ontology - Cellular Component	nuclear envelope	predicted (union)	184	147.4	0.008
Gene Ontology - Cellular Component	dendrite	predicted (intersection)	126	94.76	0.009
Gene Ontology - Cellular Component	glutamatergic synapse	predicted (intersection)	92	66.46	0.013
Gene Ontology - Cellular Component	intrinsic component of membrane	predicted (intersection)	938	860.8	0.013
Gene Ontology - Cellular Component	integral component of postsynaptic membrane	predicted (intersection)	37	22.01	0.016
Gene Ontology - Cellular Component	endomembrane system	predicted (intersection)	858	785.5	0.017
Gene Ontology - Cellular Component	integral component of membrane	predicted (intersection)	907	832.7	0.017
Gene Ontology - Cellular Component	Golgi apparatus	predicted (union)	577	515.1	0.017

Gene Ontology - Cellular Component	Golgi membrane	predicted (intersection)	165	131.4	0.018
Gene Ontology - Cellular Component	synapse	predicted (union)	459	405	0.022
Gene Ontology - Cellular Component	dendrite	predicted (union)	215	179.6	0.024
Gene Ontology - Cellular Component	dendritic tree	predicted (union)	216	180.4	0.024
Gene Ontology - Cellular Component	postsynapse	predicted (union)	249	210.6	0.024
Gene Ontology - Cellular Component	synaptic membrane	predicted (union)	176	143.9	0.024
Gene Ontology - Cellular Component	transcriptional repressor complex	predicted (union)	44	29.01	0.024
Gene Ontology - Cellular Component	cell junction	predicted (union)	485	432	0.025
Gene Ontology - Cellular Component	presynaptic membrane	predicted (intersection)	42	26.62	0.027
Gene Ontology - Cellular Component	cell junction	predicted (intersection)	269	227.9	0.028
Gene Ontology - Cellular Component	postsynaptic density	predicted (union)	129	102.5	0.028
Gene Ontology - Cellular Component	postsynaptic specialization	predicted (union)	139	112.1	0.035
Gene Ontology - Cellular Component	endoplasmic reticulum-Golgi intermediate compartment	predicted (intersection)	35	21.8	0.043
Gene Ontology - Cellular Component	host cell	predicted (intersection)	24	13.42	0.043

Gene Ontology - Cellular Component	nucleotide-activated protein kinase complex	predicted (intersection)	5	1.258	0.047
Gene Ontology - Cellular Component	mitotic spindle	predicted (intersection)	29	17.4	0.049
Gene Ontology - Cellular Component	nuclear envelope	predicted (intersection)	101	77.78	0.049
Gene Ontology - Molecular Function	DNA-binding transcription factor activity	predicted (union)	849	707.8	7.15e-10
Gene Ontology - Molecular Function	DNA-binding transcription factor activity, RNA polymerase II-specific	predicted (union)	681	566.3	5.96e-8
Gene Ontology - Molecular Function	purine nucleotide binding	predicted (union)	748	639.5	2.09e-6
Gene Ontology - Molecular Function	purine ribonucleotide binding	predicted (union)	745	637.1	2.09e-6
Gene Ontology - Molecular Function	nucleotide binding	predicted (union)	824	712.6	2.97e-6
Gene Ontology - Molecular Function	purine ribonucleoside triphosphate binding	predicted (union)	715	611.6	3.19e-6
Gene Ontology - Molecular Function	DNA binding	predicted (union)	889	776.6	5.16e-6
Gene Ontology - Molecular Function	cation binding	predicted (intersection)	881	763.3	2.98e-5
Gene Ontology - Molecular Function	metal ion binding	predicted (intersection)	872	752.4	2.98e-5
Gene Ontology - Molecular Function	kinase activity	predicted (union)	546	463.8	4.95e-5

Gene Ontology - Molecular Function	ATP binding	predicted (union)	595	511.1	7.00e-5
Gene Ontology - Molecular Function	adenyl nucleotide binding	predicted (union)	618	532.2	7.00e-5
Gene Ontology - Molecular Function	carbohydrate derivative binding	predicted (union)	842	743.2	7.00e-5
Gene Ontology - Molecular Function	DNA-binding transcription factor activity	predicted (intersection)	454	373.4	2.59e-4
Gene Ontology - Molecular Function	enzyme binding	predicted (union)	773	684	2.91e-4
Gene Ontology - Molecular Function	phosphotransferase activity, alcohol group as acceptor	predicted (union)	499	426.8	2.91e-4
Gene Ontology - Molecular Function	protein serine/threonine kinase activity	predicted (union)	328	270.2	3.21e-4
Gene Ontology - Molecular Function	enzyme binding	predicted (intersection)	438	360.8	3.80e-4
Gene Ontology - Molecular Function	protein kinase activity	predicted (union)	458	390.7	4.17e-4
Gene Ontology - Molecular Function	small molecule binding	predicted (union)	923	828.6	4.17e-4
Gene Ontology - Molecular Function	DNA-binding transcription factor activity, RNA polymerase II-specific	predicted (intersection)	365	298.7	0.001
Gene Ontology - Molecular Function	kinase activity	predicted (intersection)	306	244.6	0.001
Gene Ontology - Molecular Function	double-stranded DNA binding	predicted (union)	319	265.5	0.001

Gene Ontology - Molecular Function	drug binding	predicted (union)	658	583	0.001
Gene Ontology - Molecular Function	phosphoric ester hydrolase activity	predicted (union)	226	182.4	0.001
Gene Ontology - Molecular Function	sequence-specific DNA binding	predicted (union)	384	326.3	0.001
Gene Ontology - Molecular Function	carbohydrate derivative binding	predicted (intersection)	462	392	0.002
Gene Ontology - Molecular Function	nucleotide binding	predicted (intersection)	445	375.9	0.002
Gene Ontology - Molecular Function	protein kinase activity	predicted (intersection)	261	206.1	0.002
Gene Ontology - Molecular Function	purine nucleotide binding	predicted (intersection)	403	337.3	0.002
Gene Ontology - Molecular Function	purine ribonucleotide binding	predicted (intersection)	402	336	0.002
Gene Ontology - Molecular Function	transferase activity	predicted (intersection)	559	483.2	0.002
Gene Ontology - Molecular Function	RNA polymerase II proximal promoter sequence-specific DNA binding	predicted (union)	193	153.4	0.002
Gene Ontology - Molecular Function	sequence-specific double-stranded DNA binding	predicted (union)	290	241.2	0.002
Gene Ontology - Molecular Function	transcription regulatory region sequence-specific DNA binding	predicted (union)	276	228.9	0.002

Gene Ontology - Molecular Function	nucleic acid binding	predicted (intersection)	759	674	0.003
Gene Ontology - Molecular Function	phosphoric ester hydrolase activity	predicted (intersection)	132	96.22	0.003
Gene Ontology - Molecular Function	phosphotransferase activity, alcohol group as acceptor	predicted (intersection)	278	225.2	0.003
Gene Ontology - Molecular Function	RNA polymerase II regulatory region DNA binding	predicted (union)	257	212.6	0.003
Gene Ontology - Molecular Function	RNA polymerase II regulatory region sequence-specific DNA binding	predicted (union)	257	212.2	0.003
Gene Ontology - Molecular Function	transcription regulatory region DNA binding	predicted (union)	318	268.7	0.003
Gene Ontology - Molecular Function	DNA binding	predicted (intersection)	477	409.6	0.004
Gene Ontology - Molecular Function	purine ribonucleoside triphosphate binding	predicted (intersection)	383	322.6	0.004
Gene Ontology - Molecular Function	ubiquitin-like protein transferase activity	predicted (union)	162	128.4	0.005
Gene Ontology - Molecular Function	RNA polymerase II proximal promoter sequence-specific DNA binding	predicted (intersection)	112	80.92	0.006

Gene Ontology - Molecular Function	proximal promoter sequence-specific DNA binding	predicted (union)	196	159.4	0.006
Gene Ontology - Molecular Function	histone acetyltransferase binding	predicted (intersection)	14	5.241	0.008
Gene Ontology - Molecular Function	transcription coregulator activity	predicted (intersection)	129	96.22	0.008
Gene Ontology - Molecular Function	proximal promoter sequence-specific DNA binding	predicted (intersection)	114	84.06	0.011
Gene Ontology - Molecular Function	protein serine/threonine kinase activity	predicted (intersection)	180	142.6	0.014
Gene Ontology - Molecular Function	transcription regulatory region DNA binding	predicted (intersection)	179	141.7	0.014
Gene Ontology - Molecular Function	protein phosphatase 1 binding	predicted (union)	13	5.961	0.014
Gene Ontology - Molecular Function	phosphatase activity	predicted (union)	169	137.1	0.015
Gene Ontology - Molecular Function	histone binding	predicted (intersection)	51	32.49	0.016
Gene Ontology - Molecular Function	adenyl nucleotide binding	predicted (intersection)	330	280.7	0.017
Gene Ontology - Molecular Function	kinase binding	predicted (intersection)	156	122.2	0.017
Gene Ontology - Molecular Function	DNA-binding transcription activator activity, RNA polymerase II-specific	predicted (union)	173	141.1	0.017

Gene Ontology - Molecular Function	ubiquitin-protein transferase activity	predicted (union)	148	118.8	0.018
Gene Ontology - Molecular Function	hydrolase activity, acting on ester bonds	predicted (union)	354	308.8	0.021
Gene Ontology - Molecular Function	small molecule binding	predicted (intersection)	495	437.1	0.024
Gene Ontology - Molecular Function	RNA polymerase II regulatory region DNA binding	predicted (intersection)	143	112.2	0.025
Gene Ontology - Molecular Function	RNA polymerase II regulatory region sequence-specific DNA binding	predicted (intersection)	143	111.9	0.025
Gene Ontology - Molecular Function	SMAD binding	predicted (intersection)	25	13.42	0.025
Gene Ontology - Molecular Function	phosphoric diester hydrolase activity	predicted (intersection)	40	24.53	0.025
Gene Ontology - Molecular Function	protein serine/threonine/tyrosine kinase activity	predicted (intersection)	32	18.45	0.025
Gene Ontology - Molecular Function	transcription coactivator activity	predicted (intersection)	79	56.6	0.025
Gene Ontology - Molecular Function	transcription coregulator activity	predicted (union)	217	182.4	0.025
Gene Ontology - Molecular Function	ATP binding	predicted (intersection)	315	269.6	0.028
Gene Ontology - Molecular Function	ubiquitin conjugating enzyme binding	predicted (union)	10	4.372	0.031

Gene Ontology - Molecular Function	protein domain specific binding	predicted (union)	274	236.1	0.033
Gene Ontology - Molecular Function	DNA binding, bending	predicted (union)	13	6.359	0.038
Gene Ontology - Molecular Function	transcription regulatory region sequence-specific DNA binding	predicted (intersection)	151	120.8	0.041
Gene Ontology - Molecular Function	ubiquitin protein ligase activity	predicted (union)	74	55.64	0.046
Gene Ontology - Molecular Function	enhancer binding	predicted (union)	52	36.96	0.047
KEGG	Endometrial cancer	experimental (any)	4	0.284	0.012
KEGG	Chronic myeloid leukemia	experimental (strong)	2	0.139	0.021
KEGG	ErbB signaling pathway	experimental (strong)	2	0.139	0.021
KEGG	HIF-1 signaling pathway	experimental (strong)	2	0.157	0.021
KEGG	Measles	experimental (strong)	2	0.175	0.021
KEGG	Prostate cancer	experimental (strong)	2	0.162	0.021
KEGG	Small cell lung cancer	experimental (strong)	2	0.139	0.021
KEGG	Epstein-Barr virus infection	experimental (strong)	2	0.201	0.022
KEGG	FoxO signaling pathway	experimental (strong)	2	0.208	0.022
KEGG	Circadian rhythm	predicted (union)	20	10.73	0.024

KEGG	FoxO signaling pathway	predicted (union)	66	46.5	0.024
KEGG	Proteoglycans in cancer	predicted (union)	104	79.88	0.024
KEGG	Renal cell carcinoma	predicted (union)	36	22.65	0.024
KEGG	Hepatitis B	experimental (strong)	2	0.237	0.025
KEGG	Colorectal cancer	predicted (union)	35	22.26	0.026
KEGG	Ubiquitin mediated proteolysis	predicted (union)	69	50.87	0.036
KEGG	MAPK signaling pathway	predicted (union)	115	91.41	0.038
KEGG	mTOR signaling pathway	predicted (union)	33	21.46	0.041
KEGG	Transcriptional misregulation in cancer	predicted (union)	82	63.19	0.048
Reactome	Gene expression (Transcription)	predicted (union)	639	517	1.12e-9
Reactome	RNA Polymerase II Transcription	predicted (union)	571	465.8	5.91e-8
Reactome	Gene expression (Transcription)	predicted (intersection)	358	272.7	4.01e-6
Reactome	Generic Transcription Pathway	predicted (union)	508	420.5	6.29e-6
Reactome	RNA Polymerase II Transcription	predicted (intersection)	323	245.7	1.09e-5
Reactome	Generic Transcription Pathway	predicted (intersection)	289	221.8	1.06e-4

Reactome	Diseases of signal transduction	predicted (union)	185	138.7	1.24e-4
Reactome	Intracellular signaling by second messengers	predicted (union)	147	109.7	0.001
Reactome	AKT phosphorylates targets in the cytosol	experimental (strong)	2	0.020	0.004
Reactome	Constitutive Signaling by AKT1 E17K in Cancer	experimental (strong)	2	0.040	0.006
Reactome	Estrogen-dependent nuclear events downstream of ESR-membrane signaling	experimental (strong)	2	0.042	0.006
Reactome	PIP3 activates AKT signaling	predicted (union)	126	94.98	0.009
Reactome	Cell Cycle, Mitotic	experimental (strong)	3	0.294	0.009
Reactome	Cyclin A:Cdk2-associated events at S phase entry	experimental (strong)	2	0.064	0.009
Reactome	Cyclin E associated events during G1/S transition	experimental (strong)	2	0.062	0.009
Reactome	Neuronal System	predicted (intersection)	106	73.79	0.010
Reactome	Cell Cycle	experimental (strong)	3	0.343	0.011

Reactome	Extra-nuclear estrogen signaling	experimental (strong)	2	0.102	0.011
Reactome	FOXO-mediated transcription	experimental (strong)	2	0.104	0.011
Reactome	G1/S Transition	experimental (strong)	2	0.106	0.011
Reactome	S Phase	experimental (strong)	2	0.108	0.011
Reactome	Cell Cycle Checkpoints	experimental (strong)	2	0.137	0.016
Reactome	Mitotic G1-G1/S phases	experimental (strong)	2	0.142	0.016
Reactome	PI3K/AKT Signaling in Cancer	experimental (strong)	2	0.150	0.016
Reactome	Signal Transduction	experimental (strong)	5	1.708	0.016
Reactome	Activation of AKT2	experimental (any)	2	0.023	0.020
Reactome	Constitutive Signaling by AKT1 E17K in Cancer	experimental (any)	3	0.148	0.020
Reactome	VEGFR2 mediated vascular permeability	experimental (any)	3	0.153	0.020
Reactome	SUMOylation	predicted (union)	87	63.19	0.022
Reactome	RHO GTPase Effectors	experimental (strong)	2	0.192	0.024
Reactome	Signaling by BRAF and RAF fusions	predicted (union)	39	24.24	0.025
Reactome	ESR-mediated signaling	experimental (strong)	2	0.208	0.026

Reactome	Ligand-receptor interactions	experimental (any)	2	0.045	0.028
Reactome	Signaling by Nuclear Receptors	experimental (strong)	2	0.232	0.030
Reactome	Signaling by Rho GTPases	experimental (strong)	2	0.248	0.032
Reactome	Negative regulation of the PI3K/AKT network	predicted (union)	59	40.93	0.038
Reactome	PI5P, PP2A and IER3 Regulate PI3K/AKT Signaling	predicted (union)	56	38.55	0.038
Reactome	Regulation of cholesterol biosynthesis by SREBP (SREBF)	predicted (union)	32	19.47	0.038
Reactome	SUMO E3 ligases SUMOylate target proteins	predicted (union)	83	61.2	0.038
Reactome	Disease	experimental (strong)	3	0.739	0.038
Reactome	PIP3 activates AKT signaling	experimental (strong)	2	0.294	0.040
Reactome	Constitutive Signaling by Aberrant PI3K in Cancer	predicted (union)	43	28.22	0.041
Reactome	Transcriptional Regulation by TP53	experimental (strong)	2	0.303	0.041
Reactome	Generic Transcription Pathway	experimental (strong)	3	0.838	0.044

Reactome	Intracellular signaling by second messengers	experimental (strong)	2	0.325	0.044
Reactome	RNA Polymerase II Transcription	experimental (strong)	3	0.858	0.045
WikiPathways	MECP2 and Associated Rett Syndrome	predicted (intersection)	28	11.74	5.86e-4
WikiPathways	Pathways Regulating Hippo Signaling	predicted (union)	54	34.97	0.016
WikiPathways	Brain-Derived Neurotrophic Factor (BDNF) signaling pathway	predicted (union)	74	52.06	0.017
WikiPathways	Hippo-Merlin Signaling Dysregulation	predicted (union)	61	41.73	0.017
WikiPathways	Endometrial cancer	experimental (any)	4	0.346	0.020
WikiPathways	Aryl Hydrocarbon Receptor	experimental (any)	3	0.250	0.021
WikiPathways	Integrated Cancer Pathway	experimental (any)	3	0.244	0.021
WikiPathways	Integrin-mediated Cell Adhesion	experimental (any)	4	0.511	0.021
WikiPathways	Vitamin D Receptor Pathway	experimental (any)	5	0.874	0.021
WikiPathways	Energy Metabolism	predicted (union)	29	17.49	0.024

WikiPathways	Epithelial to mesenchymal transition in colorectal cancer	predicted (union)	79	57.63	0.024
WikiPathways	Factors and pathways affecting insulin-like growth factor (IGF1)-Akt signaling	predicted (union)	20	10.73	0.024
WikiPathways	Sterol Regulatory Element-Binding Proteins (SREBP) signalling	predicted (union)	39	25.04	0.024
WikiPathways	Synaptic signaling pathways associated with autism spectrum disorder	predicted (union)	29	17.49	0.024
WikiPathways	ErbB Signaling Pathway	experimental (strong)	2	0.150	0.024
WikiPathways	Signaling Pathways in Glioblastoma	experimental (strong)	2	0.159	0.024
WikiPathways	MECP2 and Associated Rett Syndrome	predicted (union)	35	22.26	0.025
WikiPathways	DNA Damage Response (only ATM dependent)	experimental (strong)	2	0.204	0.026
WikiPathways	Human Thyroid Stimulating Hormone (TSH) signaling pathway	predicted (intersection)	25	12.37	0.029

WikiPathways	Pathways Regulating Hippo Signaling	predicted (intersection)	33	18.45	0.029
WikiPathways	TGF-beta Signaling Pathway	predicted (intersection)	41	24.32	0.029
WikiPathways	Human Thyroid Stimulating Hormone (TSH) signaling pathway	predicted (union)	36	23.45	0.031
WikiPathways	Leptin signaling pathway	predicted (union)	40	26.63	0.031
WikiPathways	Prolactin Signaling Pathway	predicted (union)	41	27.42	0.031
WikiPathways	Chromosomal and microsatellite instability in colorectal cancer	predicted (union)	40	27.02	0.032
WikiPathways	Endoderm Differentiation	predicted (union)	67	49.68	0.032
WikiPathways	Endometrial cancer	predicted (union)	34	22.26	0.032
WikiPathways	IL-1 signaling pathway	predicted (union)	30	19.08	0.032
WikiPathways	Interleukin-11 Signaling Pathway	predicted (union)	26	15.9	0.032
WikiPathways	TGF-beta Signaling Pathway	predicted (union)	63	46.1	0.032
WikiPathways	Association Between Physico-Chemical Features and Toxicity Associated Pathways	experimental (any)	3	0.352	0.035

WikiPathways	Human Thyroid Stimulating Hormone (TSH) signaling pathway	experimental (any)	3	0.369	0.035
WikiPathways	Imatinib and Chronic Myeloid Leukemia	experimental (any)	2	0.114	0.035
WikiPathways	miRNAs involved in DNA damage response	experimental (any)	2	0.096	0.035
WikiPathways	Prion disease pathway	predicted (union)	21	12.32	0.038
WikiPathways	Chromosomal and microsatellite instability in colorectal cancer	experimental (any)	3	0.409	0.038
WikiPathways	IL-7 Signaling Pathway	experimental (any)	2	0.136	0.038
WikiPathways	ErbB Signaling Pathway	predicted (union)	45	31.79	0.042
WikiPathways	MAPK Signaling Pathway	predicted (union)	109	87.43	0.042
WikiPathways	Oncostatin M Signaling Pathway	predicted (union)	36	24.24	0.042
WikiPathways	Phosphodiesterases in neuronal function	predicted (union)	29	18.68	0.042
WikiPathways	Signaling Pathways in Glioblastoma	predicted (union)	41	28.61	0.046
WikiPathways	Signaling Pathways in Glioblastoma	experimental (any)	3	0.454	0.046

WikiPathways	Sleep regulation	experimental (any)	2	0.165	0.047
WikiPathways	ERK Pathway in Huntington's Disease	predicted (intersection)	9	2.935	0.048

Supplementary Table 1a

Database	Pathway	Evidence	Hits	Expected hits	P-value
Ontology - Biological	response to	experimental (any)	51	12	6.63e-16
Ontology - Biological	mediated signaling	experimental (any)	42	8.304	1.67e-15
Ontology - Biological	to cytokine	experimental (any)	51	12.88	4.51e-15
Ontology - Biological	defense response	experimental (any)	50	16.09	8.70e-11
Ontology - Biological	to external biotic	experimental (any)	47	14.39	8.70e-11
Ontology - Biological	to other organism	experimental (any)	47	14.38	8.70e-11
Ontology - Biological	response to organic	experimental (any)	69	28.82	1.88e-10
Ontology - Biological	immune response	experimental (any)	57	20.93	1.88e-10
Ontology - Biological	system process	experimental (any)	72	31.03	1.88e-10
Ontology - Biological	immune response	experimental (any)	35	9.221	1.65e-9
Ontology - Biological	response to type I	experimental (any)	13	0.983	2.31e-9
Ontology - Biological	to organic substance	experimental (any)	75	35.2	2.31e-9
Ontology - Biological	interferon signaling	experimental (any)	13	0.983	2.31e-9
Ontology - Biological	of gene expressio	(intersection)	821	670.6	3.32e-9
Ontology - Biological	to type I interferon	experimental (any)	13	1.023	3.64e-9
Ontology - Biological	apoptotic process	experimental (any)	55	21.68	5.11e-9
Gene Ontology - Biological Process	cell surface receptor signaling pathway	experimental (any)	69	31.41	5.11e-9
Gene Ontology - Biological Process	positive regulation of intracellular signal transduction	experimental (any)	37	10.95	6.62e-9

Gene Ontology - Biological Process	positive regulation of cellular metabolic process	experimental (any)	73	34.83	7.28e-9
Gene Ontology - Biological Process	positive regulation of signal transduction	experimental (any)	49	18.12	7.28e-9
Gene Ontology - Biological Process	response to virus	experimental (any)	21	3.561	7.28e-9
Gene Ontology - Biological Process	cell death	experimental (any)	58	24.22	9.49e-9
Gene Ontology - Biological Process	cellular response to lipopolysaccharide	experimental (any)	16	2.006	1.61e-8
Gene Ontology - Biological Process	programmed cell death	experimental (any)	55	22.71	2.07e-8
Gene Ontology - Biological Process	cellular response to molecule of bacterial origin	experimental (any)	16	2.099	2.91e-8
Gene Ontology - Biological Process	gene expression	predicted (intersection)	947	798.2	3.88e-8

Gene Ontology - Biological Process	positive regulation of cellular protein metabolic process	experimental (any)	45	16.69	4.69e-8
Gene Ontology - Biological Process	regulation of intracellular signal transduction	experimental (any)	50	19.96	4.98e-8
Gene Ontology - Biological Process	regulation of RNA metabolic process	predicted (intersection)	703	572	5.32e-8
Gene Ontology - Biological Process	positive regulation of protein modification process	experimental (any)	38	12.69	7.11e-8
Gene Ontology - Biological Process	regulation of cell death	experimental (any)	48	18.92	7.46e-8
Gene Ontology - Biological Process	response to lipopolysaccharide	experimental (any)	19	3.348	8.07e-8
Gene Ontology - Biological Process	cellular macromolecule biosynthetic process	predicted (intersection)	873	733.6	1.05e-7
Gene Ontology - Biological Process	positive regulation of metabolic process	experimental (any)	74	38.04	1.14e-7

Gene Ontology - Biological Process	regulation of cell adhesion	experimental (any)	28	7.534	1.35e-7
Gene Ontology - Biological Process	positive regulation of phosphorylation	experimental (any)	34	10.76	1.38e-7
Gene Ontology - Biological Process	positive regulation of protein phosphorylation	experimental (any)	33	10.22	1.39e-7
Gene Ontology - Biological Process	response to molecule of bacterial origin	experimental (any)	19	3.494	1.41e-7
Gene Ontology - Biological Process	intracellular signal transduction	experimental (any)	61	28.59	1.66e-7
Gene Ontology - Biological Process	leukocyte adhesion to vascular endothelial cell	experimental (any)	8	0.385	1.71e-7
Gene Ontology - Biological Process	positive regulation of protein metabolic process	experimental (any)	45	17.66	1.89e-7
Gene Ontology - Biological Process	regulation of cell population proliferation	experimental (any)	45	17.67	1.89e-7

Gene Ontology - Biological Process	positive regulation of leukocyte cell-cell adhesion	experimental (any)	16	2.471	1.99e-7
Gene Ontology - Biological Process	positive regulation of NF-kappaB transcription factor activity	experimental (any)	14	1.82	2.06e-7
Gene Ontology - Biological Process	positive regulation of cell adhesion	experimental (any)	21	4.531	2.84e-7
Gene Ontology - Biological Process	regulation of immune system process	experimental (any)	42	16.1	3.23e-7
Gene Ontology - Biological Process	positive regulation of cell-cell adhesion	experimental (any)	17	2.963	3.62e-7
Gene Ontology - Biological Process	positive regulation of leukocyte adhesion to vascular endothelial cell	experimental (any)	6	0.173	3.97e-7
Gene Ontology - Biological Process	lipopolysaccharide-mediated signaling pathway	experimental (any)	9	0.638	5.53e-7
Gene Ontology - Biological Process	RNA biosynthetic process	predicted (intersection)	686	566.7	6.21e-7

Gene Ontology - Biological Process	nucleobase-containing compound metabolic process	predicted (intersection)	986	849.2	6.21e-7
Gene Ontology - Biological Process	regulation of transcription by RNA polymerase II	predicted (intersection)	512	406.5	6.21e-7
Gene Ontology - Biological Process	transcription by RNA polymerase II	predicted (intersection)	540	431.4	6.21e-7
Gene Ontology - Biological Process	transcription, DNA-templated	predicted (intersection)	675	555.2	6.21e-7
Gene Ontology - Biological Process	regulation of viral genome replication	experimental (any)	11	1.116	6.45e-7
Gene Ontology - Biological Process	positive regulation of cellular biosynthetic process	experimental (any)	49	21.33	7.55e-7
Gene Ontology - Biological Process	negative regulation of viral genome replication	experimental (any)	9	0.664	7.56e-7
Gene Ontology - Biological Process	regulation of protein phosphorylation	experimental (any)	39	14.81	8.41e-7

Gene Ontology - Biological Process	regulation of cytokine production	experimental (any)	26	7.334	8.81e-7
Gene Ontology - Biological Process	regulation of programmed cell death	experimental (any)	43	17.47	9.36e-7
Gene Ontology - Biological Process	response to bacterium	experimental (any)	23	5.886	9.91e-7
Gene Ontology - Biological Process	secretion	experimental (any)	43	17.53	9.91e-7
Gene Ontology - Biological Process	leukocyte cell-cell adhesion	experimental (any)	18	3.627	9.94e-7
Gene Ontology - Biological Process	negative regulation of apoptotic process	experimental (any)	31	10.19	1.08e-6
Gene Ontology - Biological Process	response to lipid	experimental (any)	31	10.19	1.08e-6
Gene Ontology - Biological Process	cytokine production	experimental (any)	27	8.025	1.20e-6
Gene Ontology - Biological Process	defense response to virus	experimental (any)	15	2.525	1.33e-6
Gene Ontology - Biological Process	RNA metabolic process	predicted (intersection)	819	695	1.48e-6
Gene Ontology - Biological Process	regulation of RNA biosynthetic process	predicted (intersection)	653	539.5	1.48e-6

Gene Ontology - Biological Process	regulation of transcription, DNA-templated	predicted (intersection)	639	526.1	1.48e-6
Gene Ontology - Biological Process	negative regulation of programmed cell death	experimental (any)	31	10.39	1.56e-6
Gene Ontology - Biological Process	regulation of apoptotic process	experimental (any)	42	17.22	1.56e-6
Gene Ontology - Biological Process	cell population proliferation	experimental (any)	48	21.3	1.57e-6
Gene Ontology - Biological Process	I-kappaB kinase/NF-kappaB signaling	experimental (any)	16	2.963	1.66e-6
Gene Ontology - Biological Process	signal transduction	experimental (any)	94	58.34	2.08e-6
Gene Ontology - Biological Process	regulation of nucleic acid-templated transcription	predicted (intersection)	650	538.2	2.16e-6
Gene Ontology - Biological Process	response to mechanical stimulus	experimental (any)	14	2.285	2.39e-6
Gene Ontology - Biological Process	hemopoiesis	experimental (any)	29	9.62	3.34e-6

Gene Ontology - Biological Process	regulation of phosphorylation	experimental (any)	40	16.45	3.47e-6
Gene Ontology - Biological Process	negative regulation of cell death	experimental (any)	32	11.43	3.56e-6
Gene Ontology - Biological Process	negative regulation of viral process	experimental (any)	10	1.09	4.04e-6
Gene Ontology - Biological Process	leukocyte activation	experimental (any)	35	13.38	4.26e-6
Gene Ontology - Biological Process	cellular response to lipopolysaccharide	experimental (strong)	14	2.271	5.07e-6
Gene Ontology - Biological Process	intracellular signal transduction	experimental (strong)	45	21.89	5.07e-6
Gene Ontology - Biological Process	positive regulation of cellular protein metabolic process	experimental (strong)	37	14.84	5.07e-6
Gene Ontology - Biological Process	positive regulation of phosphorylation	experimental (strong)	30	10.66	5.07e-6
Gene Ontology - Biological Process	positive regulation of protein metabolic process	experimental (strong)	37	15.74	5.07e-6

Gene Ontology - Biological Process	positive regulation of protein modification process	experimental (strong)	32	12.13	5.07e-6
Gene Ontology - Biological Process	positive regulation of protein phosphorylation	experimental (strong)	30	10.32	5.07e-6
Gene Ontology - Biological Process	regulation of intracellular signal transduction	experimental (strong)	38	16.29	5.07e-6
Gene Ontology - Biological Process	viral genome replication	experimental (any)	11	1.422	5.42e-6
Gene Ontology - Biological Process	protein phosphorylation	experimental (any)	46	20.95	5.99e-6
Gene Ontology - Biological Process	regulation of cellular protein metabolic process	experimental (any)	55	27.49	5.99e-6
Gene Ontology - Biological Process	regulation of immune response	experimental (any)	30	10.55	6.20e-6
Gene Ontology - Biological Process	positive regulation of macromolecule biosynthetic process	experimental (any)	45	20.32	6.33e-6

Gene Ontology - Biological Process	viral process	experimental (any)	29	9.992	6.45e-6
Gene Ontology - Biological Process	leukocyte differentiation	experimental (any)	21	5.647	6.70e-6
Gene Ontology - Biological Process	regulation of signal transduction	experimental (any)	66	36.17	6.80e-6
Gene Ontology - Biological Process	positive regulation of immune system process	experimental (any)	31	11.24	6.96e-6
Gene Ontology - Biological Process	cell activation	experimental (any)	37	15.04	7.21e-6
Gene Ontology - Biological Process	positive regulation of transcription, DNA-templated	experimental (any)	39	16.38	7.41e-6
Gene Ontology - Biological Process	cellular response to lipid	experimental (any)	23	6.776	8.25e-6
Gene Ontology - Biological Process	inflammatory response	experimental (any)	23	6.79	8.44e-6
Gene Ontology - Biological Process	cellular response to molecule of bacterial origin	experimental (strong)	14	2.4	8.85e-6

Gene Ontology - Biological Process	leukocyte adhesion to vascular endothelial cell	experimental (strong)	7	0.413	8.85e-6
Gene Ontology - Biological Process	positive regulation of intracellular signal transduction	experimental (strong)	29	10.51	8.85e-6
Gene Ontology - Biological Process	positive regulation of NIK/NF-kappaB signaling	experimental (any)	9	0.930	9.02e-6
Gene Ontology - Biological Process	positive regulation of cytokine production	experimental (any)	19	4.797	9.02e-6
Gene Ontology - Biological Process	positive regulation of signal transduction	experimental (strong)	36	15.62	9.35e-6
Gene Ontology - Biological Process	lipopolysaccharide-mediated signaling pathway	experimental (strong)	9	0.852	9.68e-6
Gene Ontology - Biological Process	positive regulation of cellular metabolic process	experimental (strong)	50	28.06	9.68e-6
Gene Ontology - Biological Process	cell adhesion	experimental (any)	36	14.62	9.72e-6

Gene Ontology - Biological Process	regulation of phosphorus metabolic process	experimental (any)	42	18.66	9.91e-6
Gene Ontology - Biological Process	cellular protein modification process	experimental (any)	75	44.15	1.08e-5
Gene Ontology - Biological Process	positive regulation of cell death	experimental (any)	25	8.012	1.08e-5
Gene Ontology - Biological Process	positive regulation of cell differentiation	experimental (any)	30	10.92	1.08e-5
Gene Ontology - Biological Process	protein modification process	experimental (any)	75	44.15	1.08e-5
Gene Ontology - Biological Process	cellular response to interleukin-1	experimental (any)	12	1.913	1.15e-5
Gene Ontology - Biological Process	positive regulation of DNA-binding transcription factor activity	experimental (any)	15	3.096	1.21e-5
Gene Ontology - Biological Process	regulation of protein metabolic process	experimental (any)	56	29.17	1.36e-5
Gene Ontology - Biological Process	regulation of cell-cell adhesion	experimental (any)	18	4.491	1.40e-5

Gene Ontology - Biological Process	positive regulation of gene expression	experimental (any)	45	21.23	1.72e-5
Gene Ontology - Biological Process	regulation of MAPK cascade	experimental (any)	24	7.706	1.79e-5
Gene Ontology - Biological Process	regulation of cell differentiation	experimental (any)	43	19.9	1.86e-5
Gene Ontology - Biological Process	regulation of viral life cycle	experimental (any)	11	1.661	1.86e-5
Gene Ontology - Biological Process	regulation of viral process	experimental (any)	13	2.392	1.86e-5
Gene Ontology - Biological Process	immune system development	experimental (any)	29	10.72	2.07e-5
Gene Ontology - Biological Process	cell communication	experimental (any)	96	63.53	2.21e-5
Gene Ontology - Biological Process	regulation of hemopoiesis	experimental (any)	19	5.169	2.30e-5
Gene Ontology - Biological Process	leukocyte activation involved in immune response	experimental (any)	24	7.866	2.40e-5
Gene Ontology - Biological Process	cell activation involved in immune response	experimental (any)	24	7.919	2.67e-5

Gene Ontology - Biological Process	positive regulation of nucleic acid-templated transcription	experimental (any)	39	17.49	2.93e-5
Gene Ontology - Biological Process	positive regulation of RNA biosynthetic process	experimental (any)	39	17.5	2.95e-5
Gene Ontology - Biological Process	regulation of cell migration	experimental (any)	26	9.168	3.07e-5
Gene Ontology - Biological Process	regulation of cell motility	experimental (any)	27	9.766	3.07e-5
Gene Ontology - Biological Process	secretion by cell	experimental (any)	37	16.18	3.07e-5
Gene Ontology - Biological Process	viral life cycle	experimental (any)	16	3.827	3.07e-5
Gene Ontology - Biological Process	signaling	experimental (any)	95	63.18	3.13e-5
Gene Ontology - Biological Process	MAPK cascade	experimental (any)	27	9.872	3.70e-5
Gene Ontology - Biological Process	response to interleukin-1	experimental (any)	12	2.179	3.72e-5

Gene Ontology - Biological Process	regulation of cellular protein metabolic process	experimental (strong)	42	21.6	3.84e-5
Gene Ontology - Biological Process	negative regulation of cell population proliferation	experimental (any)	23	7.56	3.87e-5
Gene Ontology - Biological Process	signal transduction by protein phosphorylation	experimental (any)	27	9.912	3.89e-5
Gene Ontology - Biological Process	protein phosphorylation	experimental (strong)	38	18.3	3.97e-5
Gene Ontology - Biological Process	response to lipopolysaccharide	experimental (strong)	16	3.717	3.97e-5
Gene Ontology - Biological Process	regulation of I-kappaB kinase/NF-kappaB signaling	experimental (any)	13	2.631	4.50e-5
Gene Ontology - Biological Process	regulation of myeloid cell differentiation	experimental (any)	13	2.631	4.50e-5
Gene Ontology - Biological Process	protein metabolic process	experimental (any)	91	60.11	4.55e-5

Gene Ontology - Biological Process	regulation of secretion	experimental (any)	24	8.238	4.65e-5
Gene Ontology - Biological Process	leukocyte activation involved in immune response	experimental (strong)	19	5.343	4.73e-5
Gene Ontology - Biological Process	positive regulation of NIK/NF-kappaB signaling	experimental (strong)	9	1.058	4.73e-5
Gene Ontology - Biological Process	positive regulation of leukocyte adhesion to vascular endothelial cell	experimental (strong)	5	0.206	4.73e-5
Gene Ontology - Biological Process	regulation of leukocyte migration	experimental (strong)	11	1.704	4.73e-5
Gene Ontology - Biological Process	cellular response to chemokine	experimental (any)	7	0.624	4.74e-5
Gene Ontology - Biological Process	regulation of signaling	experimental (any)	68	40.27	4.82e-5
Gene Ontology - Biological Process	response to stimulus	experimental (any)	121	88.74	4.82e-5
Gene Ontology - Biological Process	leukocyte migration	experimental (any)	17	4.491	4.86e-5

Gene Ontology - Biological Process	cell activation involved in immune response	experimental (strong)	19	5.395	4.87e-5
Gene Ontology - Biological Process	response to interferon-gamma	experimental (any)	11	1.873	4.91e-5
Gene Ontology - Biological Process	cellular response to cytokine stimulus	experimental (strong)	28	11.07	5.07e-5
Gene Ontology - Biological Process	regulation of protein phosphorylation	experimental (strong)	32	13.96	5.07e-5
Gene Ontology - Biological Process	response to molecule of bacterial origin	experimental (strong)	16	3.872	5.07e-5
Gene Ontology - Biological Process	lymphocyte activation	experimental (any)	22	7.162	5.12e-5
Gene Ontology - Biological Process	MyD88-dependent toll-like receptor signaling pathway	experimental (any)	6	0.425	5.93e-5
Gene Ontology - Biological Process	positive regulation of cell activation	experimental (any)	15	3.614	6.08e-5
Gene Ontology - Biological Process	positive regulation of T cell activation	experimental (any)	12	2.312	6.09e-5

Gene Ontology - Biological Process	regulation of RNA metabolic process	experimental (any)	69	41.46	6.18e-5
Gene Ontology - Biological Process	regulation of leukocyte migration	experimental (any)	11	1.927	6.18e-5
Gene Ontology - Biological Process	cellular macromolecule biosynthetic process	experimental (any)	83	53.51	6.24e-5
Gene Ontology - Biological Process	myeloid leukocyte activation	experimental (any)	22	7.308	6.71e-5
Gene Ontology - Biological Process	positive regulation of MAPK cascade	experimental (any)	19	5.674	6.94e-5
Gene Ontology - Biological Process	regulation of cellular component organization	predicted (intersection)	446	361.3	6.99e-5
Gene Ontology - Biological Process	cellular protein metabolic process	experimental (any)	84	54.6	7.16e-5
Gene Ontology - Biological Process	cell migration	experimental (any)	34	14.99	8.14e-5
Gene Ontology - Biological Process	leukocyte mediated immunity	experimental (any)	24	8.597	8.14e-5
Gene Ontology - Biological Process	regulation of NIK/NF-kappaB signaling	experimental (any)	9	1.276	8.14e-5

Gene Ontology - Biological Process	regulation of extrinsic apoptotic signaling pathway via death domain receptors	experimental (any)	7	0.691	8.14e-5
Gene Ontology - Biological Process	regulation of nitrogen compound metabolic process	experimental (any)	92	61.97	8.14e-5
Gene Ontology - Biological Process	response to glucocorticoid	experimental (any)	10	1.621	8.14e-5
Gene Ontology - Biological Process	response to organic cyclic compound	experimental (any)	27	10.42	8.14e-5
Gene Ontology - Biological Process	cellular response to mechanical stimulus	experimental (any)	8	0.970	8.22e-5
Gene Ontology - Biological Process	regulation of chemotaxis	experimental (any)	11	2.006	8.27e-5
Gene Ontology - Biological Process	bone remodeling	experimental (any)	8	0.983	8.98e-5
Gene Ontology - Biological Process	positive regulation of leukocyte migration	experimental (any)	9	1.302	9.08e-5

Gene Ontology - Biological Process	phosphorylation	experimental (strong)	39	20	9.21e-5
Gene Ontology - Biological Process	positive regulation of leukocyte migration	experimental (strong)	9	1.187	9.71e-5
Gene Ontology - Biological Process	positive regulation of metabolic process	experimental (strong)	50	30.43	9.71e-5
Gene Ontology - Biological Process	regulation of protein metabolic process	experimental (strong)	42	22.74	9.71e-5
Gene Ontology - Biological Process	positive regulation of I-kappaB kinase/NF-kappaB signaling	experimental (any)	11	2.046	9.79e-5
Gene Ontology - Biological Process	reactive oxygen species metabolic process	experimental (any)	13	2.897	1.02e-4
Gene Ontology - Biological Process	regulation of locomotion	experimental (any)	27	10.58	1.02e-4
Gene Ontology - Biological Process	regulation of cellular component movement	experimental (any)	27	10.6	1.05e-4

Gene Ontology - Biological Process	toll-like receptor signaling pathway	experimental (any)	10	1.687	1.07e-4
Gene Ontology - Biological Process	protein secretion	experimental (any)	19	5.926	1.13e-4
Gene Ontology - Biological Process	regulation of RNA biosynthetic process	experimental (any)	65	38.98	1.13e-4
Gene Ontology - Biological Process	myeloid leukocyte activation	experimental (strong)	17	4.723	1.20e-4
Gene Ontology - Biological Process	RNA biosynthetic process	experimental (any)	67	40.96	1.45e-4
Gene Ontology - Biological Process	immune response	experimental (strong)	31	13.99	1.49e-4
Gene Ontology - Biological Process	transcription, DNA-templated	experimental (any)	66	40.18	1.51e-4
Gene Ontology - Biological Process	phosphorylation	experimental (any)	48	25.76	1.57e-4
Gene Ontology - Biological Process	regulation of DNA-binding transcription factor activity	experimental (any)	17	5.009	1.63e-4
Gene Ontology - Biological Process	regulation of cell population proliferation	experimental (strong)	35	17.24	1.69e-4

Gene Ontology - Biological Process	regulation of signal transduction	experimental (strong)	45	26.07	1.69e-4
Gene Ontology - Biological Process	response to cytokine	experimental (strong)	28	11.9	1.69e-4
Gene Ontology - Biological Process	regulation of phosphorylation	experimental (strong)	32	14.94	1.78e-4
Gene Ontology - Biological Process	chemotaxis	experimental (any)	19	6.139	1.80e-4
Gene Ontology - Biological Process	nervous system development	predicted (union)	883	770.8	1.81e-4
Gene Ontology - Biological Process	T cell activation	experimental (any)	17	5.062	1.84e-4
Gene Ontology - Biological Process	myeloid cell differentiation	experimental (any)	16	4.544	1.85e-4
Gene Ontology - Biological Process	regulation of lymphocyte activation	experimental (any)	16	4.557	1.90e-4
Gene Ontology - Biological Process	regulation of nucleic acid-templated transcription	experimental (any)	64	38.86	1.94e-4
Gene Ontology - Biological Process	response to nitrogen compound	experimental (any)	28	11.67	1.94e-4

Gene Ontology - Biological Process	pattern recognition receptor signaling pathway	experimental (any)	11	2.232	2.01e-4
Gene Ontology - Biological Process	regulation of transcription, DNA-templated	experimental (any)	63	38.09	2.02e-4
Gene Ontology - Biological Process	response to corticosteroid	experimental (any)	10	1.834	2.02e-4
Gene Ontology - Biological Process	peptide secretion	experimental (any)	19	6.218	2.04e-4
Gene Ontology - Biological Process	cell death	experimental (strong)	39	20.86	2.10e-4
Gene Ontology - Biological Process	cellular protein metabolic process	experimental (strong)	51	32.37	2.10e-4
Gene Ontology - Biological Process	protein metabolic process	experimental (strong)	53	34.51	2.10e-4
Gene Ontology - Biological Process	response to hypoxia	experimental (any)	15	4.106	2.14e-4
Gene Ontology - Biological Process	anatomical structure morphogenesis	predicted (union)	965	852.4	2.16e-4
Gene Ontology - Biological Process	dephosphorylation	predicted (union)	213	160.9	2.16e-4

Gene Ontology - Biological Process	negative regulation of gene expression	predicted (union)	675	581	2.16e-4
Gene Ontology - Biological Process	positive regulation of apoptotic process	experimental (any)	21	7.401	2.16e-4
Gene Ontology - Biological Process	positive regulation of programmed cell death	experimental (any)	21	7.454	2.39e-4
Gene Ontology - Biological Process	leukocyte migration	experimental (strong)	15	4.001	2.51e-4
Gene Ontology - Biological Process	positive regulation of leukocyte cell-cell adhesion	experimental (strong)	12	2.581	2.53e-4
Gene Ontology - Biological Process	regulation of cell adhesion	experimental (strong)	21	7.485	2.53e-4
Gene Ontology - Biological Process	regulation of phosphorus metabolic process	experimental (strong)	33	16.11	2.53e-4
Gene Ontology - Biological Process	response to mechanical stimulus	experimental (strong)	12	2.581	2.53e-4
Gene Ontology - Biological Process	cell chemotaxis	experimental (any)	12	2.777	2.95e-4

Gene Ontology - Biological Process	apoptotic process	experimental (strong)	37	19.56	3.00e-4
Gene Ontology - Biological Process	regulation of NIK/NF-kappaB signaling	experimental (strong)	9	1.42	3.00e-4
Gene Ontology - Biological Process	positive regulation of cell adhesion	experimental (strong)	16	4.672	3.22e-4
Gene Ontology - Biological Process	positive regulation of cell population proliferation	experimental (any)	25	10.1	3.28e-4
Gene Ontology - Biological Process	positive regulation of immune response	experimental (any)	22	8.225	3.28e-4
Gene Ontology - Biological Process	cell motility	experimental (any)	34	16.2	3.38e-4
Gene Ontology - Biological Process	cytokine biosynthetic process	experimental (any)	8	1.209	3.46e-4
Gene Ontology - Biological Process	nitric oxide metabolic process	experimental (any)	7	0.890	3.59e-4
Gene Ontology - Biological Process	tube morphogenesis	experimental (any)	24	9.54	3.64e-4
Gene Ontology - Biological Process	cytokine metabolic process	experimental (any)	8	1.222	3.68e-4

Gene Ontology - Biological Process	response to organonitrogen compound	experimental (any)	26	10.87	3.87e-4
Gene Ontology - Biological Process	neuron differentiation	predicted (union)	512	432.2	3.91e-4
Gene Ontology - Biological Process	cytokine-mediated signaling pathway	experimental (strong)	21	7.743	3.92e-4
Gene Ontology - Biological Process	positive regulation of protein serine/threonine kinase activity	experimental (any)	14	3.84	3.93e-4
Gene Ontology - Biological Process	lymphocyte differentiation	experimental (any)	14	3.853	4.06e-4
Gene Ontology - Biological Process	regulation of protein secretion	experimental (any)	16	4.903	4.11e-4
Gene Ontology - Biological Process	heart valve morphogenesis	experimental (any)	6	0.624	4.12e-4
Gene Ontology - Biological Process	interleukin-1-mediated signaling pathway	experimental (any)	8	1.249	4.16e-4
Gene Ontology - Biological Process	programmed cell death	experimental (strong)	37	19.9	4.32e-4
Gene Ontology - Biological Process	leukocyte chemotaxis	experimental (any)	10	2.033	4.43e-4

Gene Ontology - Biological Process	leukocyte mediated immunity	experimental (strong)	17	5.395	4.55e-4
Gene Ontology - Biological Process	positive regulation of cell-cell adhesion	experimental (strong)	13	3.252	4.55e-4
Gene Ontology - Biological Process	cell-cell adhesion	experimental (any)	22	8.451	4.57e-4
Gene Ontology - Biological Process	response to steroid hormone	experimental (any)	15	4.425	4.58e-4
Gene Ontology - Biological Process	leukocyte cell-cell adhesion	experimental (strong)	14	3.768	4.65e-4
Gene Ontology - Biological Process	cell population proliferation	experimental (strong)	37	20.03	4.69e-4
Gene Ontology - Biological Process	reactive oxygen species biosynthetic process	experimental (any)	8	1.276	4.74e-4
Gene Ontology - Biological Process	regulation of interleukin-12 biosynthetic process	experimental (any)	3	0.080	5.11e-4
Gene Ontology - Biological Process	regulation of reactive oxygen species metabolic process	experimental (any)	10	2.073	5.11e-4

Gene Ontology - Biological Process	neurogenesis	predicted (union)	601	516.2	5.33e-4
Gene Ontology - Biological Process	immune response-regulating signaling pathway	experimental (any)	18	6.165	5.46e-4
Gene Ontology - Biological Process	activation of NF-kappaB-inducing kinase activity	experimental (any)	4	0.213	5.48e-4
Gene Ontology - Biological Process	cellular response to interferon-gamma	experimental (any)	9	1.687	5.55e-4
Gene Ontology - Biological Process	cellular response to interleukin-6	experimental (any)	5	0.412	5.64e-4
Gene Ontology - Biological Process	positive regulation of cell migration	experimental (any)	17	5.62	5.64e-4
Gene Ontology - Biological Process	regulation of T cell activation	experimental (any)	13	3.494	5.64e-4
Gene Ontology - Biological Process	response to bacterium	experimental (strong)	16	4.956	6.01e-4
Gene Ontology - Biological Process	generation of neurons	predicted (union)	566	484.9	6.10e-4
Gene Ontology - Biological Process	regulation of cellular component organization	predicted (union)	889	788.3	6.10e-4

Gene Ontology - Biological Process	response to growth factor	experimental (any)	21	8.039	6.21e-4
Gene Ontology - Biological Process	response to hormone	experimental (any)	25	10.59	6.23e-4
Gene Ontology - Biological Process	regulation of protein localization	experimental (any)	26	11.27	6.36e-4
Gene Ontology - Biological Process	regulation of cell death	experimental (strong)	34	17.78	6.57e-4
Gene Ontology - Biological Process	cellular response to mechanical stimulus	experimental (strong)	8	1.239	7.17e-4
Gene Ontology - Biological Process	neutrophil activation	experimental (any)	17	5.767	7.48e-4
Gene Ontology - Biological Process	positive regulation of protein kinase activity	experimental (any)	17	5.767	7.48e-4
Gene Ontology - Biological Process	response to interleukin-6	experimental (any)	5	0.438	7.48e-4
Gene Ontology - Biological Process	cell fate commitment	experimental (any)	11	2.644	7.58e-4
Gene Ontology - Biological Process	extrinsic apoptotic signaling pathway via death domain receptors	experimental (any)	7	1.023	7.58e-4

Gene Ontology - Biological Process	negative regulation of signal transduction	experimental (any)	30	14.15	7.58e-4
Gene Ontology - Biological Process	regulation of reactive oxygen species biosynthetic process	experimental (any)	7	1.023	7.58e-4
Gene Ontology - Biological Process	MAPK cascade	experimental (strong)	23	9.524	7.70e-4
Gene Ontology - Biological Process	positive regulation of cell motility	experimental (any)	17	5.793	7.72e-4
Gene Ontology - Biological Process	cell projection morphogenesis	predicted (union)	250	197.6	8.06e-4
Gene Ontology - Biological Process	regulation of interleukin-23 production	experimental (any)	3	0.093	8.09e-4
Gene Ontology - Biological Process	positive regulation of cellular biosynthetic process	experimental (strong)	34	17.99	8.12e-4
Gene Ontology - Biological Process	signal transduction by protein phosphorylation	experimental (strong)	23	9.576	8.13e-4

Gene Ontology - Biological Process	nucleotide-binding oligomerization domain containing signaling pathway	experimental (any)	5	0.452	8.26e-4
Gene Ontology - Biological Process	regulation of establishment of protein localization	experimental (any)	21	8.238	8.26e-4
Gene Ontology - Biological Process	tissue development	experimental (any)	39	20.81	8.27e-4
Gene Ontology - Biological Process	response to drug	experimental (any)	25	10.83	8.32e-4
Gene Ontology - Biological Process	regulation of protein transport	experimental (any)	20	7.64	8.46e-4
Gene Ontology - Biological Process	regulation of cytokine-mediated signaling pathway	experimental (any)	9	1.807	8.52e-4
Gene Ontology - Biological Process	negative regulation of biosynthetic process	predicted (union)	566	487.3	8.54e-4
Gene Ontology - Biological Process	positive regulation of RNA biosynthetic process	predicted (union)	602	520.9	8.54e-4

Gene Ontology - Biological Process	regulation of cell differentiation	predicted (union)	682	595.8	8.54e-4
Gene Ontology - Biological Process	negative regulation of cellular metabolic process	predicted (union)	900	802.4	8.59e-4
Gene Ontology - Biological Process	positive regulation of nucleic acid-templated transcription	predicted (union)	601	520.5	8.59e-4
Gene Ontology - Biological Process	positive regulation of immune system process	experimental (strong)	22	8.931	8.60e-4
Gene Ontology - Biological Process	cytoplasmic pattern recognition receptor signaling pathway	experimental (any)	6	0.731	8.64e-4
Gene Ontology - Biological Process	regulation of peptidyl-tyrosine phosphorylation	experimental (any)	11	2.697	8.64e-4
Gene Ontology - Biological Process	regulation of chemotaxis	experimental (strong)	10	2.091	9.01e-4

Gene Ontology - Biological Process	nucleotide-binding domain, leucine rich repeat containing receptor signaling pathway	experimental (any)	5	0.465	9.05e-4
Gene Ontology - Biological Process	positive regulation of myeloid cell differentiation	experimental (any)	7	1.063	9.05e-4
Gene Ontology - Biological Process	regulation of cell activation	experimental (any)	17	5.899	9.05e-4
Gene Ontology - Biological Process	response to vitamin D	experimental (any)	5	0.465	9.05e-4
Gene Ontology - Biological Process	semi-lunar valve development	experimental (any)	5	0.465	9.05e-4
Gene Ontology - Biological Process	intracellular transport	predicted (union)	621	539.6	9.07e-4
Gene Ontology - Biological Process	locomotion	experimental (any)	36	18.71	9.10e-4
Gene Ontology - Biological Process	positive regulation of transcription by RNA polymerase II	experimental (any)	27	12.28	9.10e-4

Gene Ontology - Biological Process	response to alcohol	experimental (any)	11	2.724	9.10e-4
Gene Ontology - Biological Process	positive regulation of cellular component movement	experimental (any)	17	5.913	9.11e-4
Gene Ontology - Biological Process	heart valve development	experimental (any)	6	0.744	9.13e-4
Gene Ontology - Biological Process	positive regulation of reactive oxygen species metabolic process	experimental (any)	7	1.076	9.46e-4
Gene Ontology - Biological Process	positive regulation of smooth muscle cell proliferation	experimental (any)	7	1.076	9.46e-4
Gene Ontology - Biological Process	response to interferon-alpha	experimental (any)	4	0.252	9.47e-4
Gene Ontology - Biological Process	monocyte chemotaxis	experimental (any)	5	0.478	9.99e-4
Gene Ontology - Biological Process	anatomical structure morphogenesis	predicted (intersection)	467	390.6	0.001

Gene Ontology - Biological Process	negative regulation of gene expression	predicted (intersection)	332	266.2	0.001
Gene Ontology - Biological Process	axonogenesis	predicted (union)	179	137.1	0.001
Gene Ontology - Biological Process	cell part morphogenesis	predicted (union)	254	203.4	0.001
Gene Ontology - Biological Process	neuron projection morphogenesis	predicted (union)	240	190.9	0.001
Gene Ontology - Biological Process	positive regulation of gene expression	predicted (union)	716	629.8	0.001
Gene Ontology - Biological Process	positive regulation of transcription, DNA-templated	predicted (union)	561	484.9	0.001
Gene Ontology - Biological Process	NIK/NF-kappaB signaling	experimental (strong)	9	1.729	0.001
Gene Ontology - Biological Process	cell activation	experimental (strong)	26	11.95	0.001
Gene Ontology - Biological Process	cellular protein modification process	experimental (strong)	45	28.44	0.001
Gene Ontology - Biological Process	leukocyte activation	experimental (strong)	24	10.51	0.001

Gene Ontology - Biological Process	neutrophil activation	experimental (strong)	12	3.149	0.001
Gene Ontology - Biological Process	positive regulation of MAPK cascade	experimental (strong)	17	6.014	0.001
Gene Ontology - Biological Process	protein modification process	experimental (strong)	45	28.44	0.001
Gene Ontology - Biological Process	regulation of immune response	experimental (strong)	20	7.795	0.001
Gene Ontology - Biological Process	regulation of signaling	experimental (strong)	45	28.44	0.001
Gene Ontology - Biological Process	response to lipid	experimental (strong)	23	9.912	0.001
Gene Ontology - Biological Process	NIK/NF-kappaB signaling	experimental (any)	9	1.98	0.001
Gene Ontology - Biological Process	T cell differentiation	experimental (any)	11	2.777	0.001
Gene Ontology - Biological Process	anatomical structure formation involved in morphogenesis	experimental (any)	26	11.95	0.001
Gene Ontology - Biological Process	blood vessel morphogenesis	experimental (any)	18	6.644	0.001

Gene Ontology - Biological Process	cellular response to hormone stimulus	experimental (any)	19	7.454	0.001
Gene Ontology - Biological Process	chemokine-mediated signaling pathway	experimental (any)	5	0.531	0.001
Gene Ontology - Biological Process	cytokine production involved in immune response	experimental (any)	7	1.183	0.001
Gene Ontology - Biological Process	entry into host cell	experimental (any)	8	1.528	0.001
Gene Ontology - Biological Process	forebrain development	experimental (any)	14	4.292	0.001
Gene Ontology - Biological Process	innate immune response-activating signal transduction	experimental (any)	12	3.362	0.001
Gene Ontology - Biological Process	mononuclear cell migration	experimental (any)	6	0.771	0.001
Gene Ontology - Biological Process	negative regulation of cell migration	experimental (any)	11	2.817	0.001
Gene Ontology - Biological Process	negative regulation of interleukin-23 production	experimental (any)	2	0.027	0.001

Gene Ontology - Biological Process	negative regulation of leukocyte apoptotic process	experimental (any)	5	0.531	0.001
Gene Ontology - Biological Process	nitric oxide biosynthetic process	experimental (any)	6	0.824	0.001
Gene Ontology - Biological Process	osteoclast differentiation	experimental (any)	7	1.143	0.001
Gene Ontology - Biological Process	positive regulation of T cell migration	experimental (any)	4	0.292	0.001
Gene Ontology - Biological Process	positive regulation of kinase activity	experimental (any)	17	6.205	0.001
Gene Ontology - Biological Process	positive regulation of locomotion	experimental (any)	17	6.152	0.001
Gene Ontology - Biological Process	positive regulation of secretion	experimental (any)	14	4.478	0.001
Gene Ontology - Biological Process	regulation of MAP kinase activity	experimental (any)	13	3.8	0.001
Gene Ontology - Biological Process	regulation of angiogenesis	experimental (any)	12	3.282	0.001
Gene Ontology - Biological Process	regulation of cellular process	experimental (any)	134	108.2	0.001

Gene Ontology - Biological Process	regulation of epithelial cell proliferation	experimental (any)	13	3.827	0.001
Gene Ontology - Biological Process	regulation of innate immune response	experimental (any)	15	4.929	0.001
Gene Ontology - Biological Process	regulation of leukocyte proliferation	experimental (any)	10	2.352	0.001
Gene Ontology - Biological Process	regulation of osteoclast differentiation	experimental (any)	6	0.784	0.001
Gene Ontology - Biological Process	regulation of protein serine/threonine kinase activity	experimental (any)	16	5.581	0.001
Gene Ontology - Biological Process	regulation of type I interferon production	experimental (any)	8	1.475	0.001
Gene Ontology - Biological Process	response to estradiol	experimental (any)	8	1.501	0.001
Gene Ontology - Biological Process	response to hydroxyisoflavone	experimental (any)	2	0.027	0.001
Gene Ontology - Biological Process	response to ultrasound	experimental (any)	2	0.027	0.001

Gene Ontology - Biological Process	response to vitamin	experimental (any)	7	1.156	0.001
Gene Ontology - Biological Process	stem cell population maintenance	experimental (any)	9	1.98	0.001
Gene Ontology - Biological Process	system development	experimental (any)	73	49.77	0.001
Gene Ontology - Biological Process	telencephalon development	experimental (any)	11	2.883	0.001
Gene Ontology - Biological Process	tissue remodeling	experimental (any)	9	1.873	0.001
Gene Ontology - Biological Process	toll-like receptor 9 signaling pathway	experimental (any)	4	0.292	0.001
Gene Ontology - Biological Process	type I interferon biosynthetic process	experimental (any)	3	0.120	0.001
Gene Ontology - Biological Process	type I interferon production	experimental (any)	8	1.501	0.001
Gene Ontology - Biological Process	nervous system development	predicted (intersection)	426	353.2	0.002
Gene Ontology - Biological Process	central nervous system neuron differentiation	predicted (union)	91	62.86	0.002

Gene Ontology - Biological Process	regulation of organelle organization	predicted (union)	470	402.2	0.002
Gene Ontology - Biological Process	cellular response to lipid	experimental (strong)	18	6.84	0.002
Gene Ontology - Biological Process	positive regulation of cell population proliferation	experimental (strong)	23	10.43	0.002
Gene Ontology - Biological Process	positive regulation of cytokine production	experimental (strong)	14	4.465	0.002
Gene Ontology - Biological Process	reactive oxygen species metabolic process	experimental (strong)	11	2.865	0.002
Gene Ontology - Biological Process	regulation of MAPK cascade	experimental (strong)	20	8.027	0.002
Gene Ontology - Biological Process	regulation of cellular extravasation	experimental (strong)	3	0.103	0.002
Gene Ontology - Biological Process	secretion	experimental (strong)	25	11.9	0.002
Gene Ontology - Biological Process	RNA metabolic process	experimental (any)	73	50.57	0.002
Gene Ontology - Biological Process	T cell migration	experimental (any)	5	0.611	0.002

Gene Ontology - Biological Process	activation of immune response	experimental (any)	17	6.497	0.002
Gene Ontology - Biological Process	activation of innate immune response	experimental (any)	12	3.627	0.002
Gene Ontology - Biological Process	biosynthetic process	experimental (any)	92	67.59	0.002
Gene Ontology - Biological Process	cardiac septum morphogenesis	experimental (any)	6	0.943	0.002
Gene Ontology - Biological Process	cellular response to drug	experimental (any)	13	4.119	0.002
Gene Ontology - Biological Process	cellular response to growth factor stimulus	experimental (any)	19	7.706	0.002
Gene Ontology - Biological Process	central nervous system development	experimental (any)	24	10.84	0.002
Gene Ontology - Biological Process	endoderm development	experimental (any)	6	0.917	0.002
Gene Ontology - Biological Process	extrinsic apoptotic signaling pathway	experimental (any)	10	2.657	0.002
Gene Ontology - Biological Process	glial cell differentiation	experimental (any)	10	2.538	0.002
Gene Ontology - Biological Process	immune response-activating signal transduction	experimental (any)	16	5.727	0.002

Gene Ontology - Biological Process	interferon-alpha production	experimental (any)	4	0.332	0.002
Gene Ontology - Biological Process	negative regulation of cell motility	experimental (any)	11	2.976	0.002
Gene Ontology - Biological Process	negative regulation of signaling	experimental (any)	30	15.31	0.002
Gene Ontology - Biological Process	negative regulation of transport	experimental (any)	15	5.155	0.002
Gene Ontology - Biological Process	neutrophil mediated immunity	experimental (any)	16	5.767	0.002
Gene Ontology - Biological Process	positive regulation of MAP kinase activity	experimental (any)	11	2.976	0.002
Gene Ontology - Biological Process	positive regulation of epithelial to mesenchymal transition	experimental (any)	5	0.611	0.002
Gene Ontology - Biological Process	positive regulation of gliogenesis	experimental (any)	6	0.850	0.002
Gene Ontology - Biological Process	positive regulation of innate immune response	experimental (any)	13	4.199	0.002

Gene Ontology - Biological Process	positive regulation of interleukin-6 production	experimental (any)	6	0.890	0.002
Gene Ontology - Biological Process	positive regulation of protein complex assembly	experimental (any)	10	2.618	0.002
Gene Ontology - Biological Process	positive regulation of reactive oxygen species biosynthetic process	experimental (any)	5	0.585	0.002
Gene Ontology - Biological Process	positive regulation of type I interferon production	experimental (any)	6	0.890	0.002
Gene Ontology - Biological Process	positive regulation of vascular endothelial growth factor production	experimental (any)	4	0.332	0.002
Gene Ontology - Biological Process	positive regulation of vasoconstriction	experimental (any)	4	0.319	0.002
Gene Ontology - Biological Process	proteolysis	experimental (any)	33	17.54	0.002

Gene Ontology - Biological Process	regulation of ERK1 and ERK2 cascade	experimental (any)	11	2.976	0.002
Gene Ontology - Biological Process	regulation of T cell proliferation	experimental (any)	8	1.674	0.002
Gene Ontology - Biological Process	regulation of actin cytoskeleton organization	experimental (any)	12	3.468	0.002
Gene Ontology - Biological Process	regulation of calcium ion transport	experimental (any)	10	2.618	0.002
Gene Ontology - Biological Process	regulation of cytokine production involved in immune response	experimental (any)	6	0.943	0.002
Gene Ontology - Biological Process	regulation of gene expression	experimental (any)	71	48.79	0.002
Gene Ontology - Biological Process	regulation of metabolic process	experimental (any)	95	70.99	0.002
Gene Ontology - Biological Process	regulation of protein kinase activity	experimental (any)	20	8.344	0.002

Gene Ontology - Biological Process	regulation of tissue remodeling	experimental (any)	6	0.877	0.002
Gene Ontology - Biological Process	tube development	experimental (any)	25	11.64	0.002
Gene Ontology - Biological Process	negative regulation of transcription, DNA-templated	predicted (union)	430	367	0.003
Gene Ontology - Biological Process	MyD88-dependent toll-like receptor signaling pathway	experimental (strong)	5	0.516	0.003
Gene Ontology - Biological Process	activation of NF-kappaB-inducing kinase activity	experimental (strong)	4	0.310	0.003
Gene Ontology - Biological Process	cell adhesion	experimental (strong)	24	11.56	0.003
Gene Ontology - Biological Process	cellular macromolecule biosynthetic process	experimental (strong)	45	29.79	0.003
Gene Ontology - Biological Process	cellular response to interleukin-6	experimental (strong)	5	0.568	0.003
Gene Ontology - Biological Process	cellular response to organic substance	experimental (strong)	40	24.65	0.003

Gene Ontology - Biological Process	macromolecule metabolic process	experimental (strong)	62	48.86	0.003
Gene Ontology - Biological Process	negative regulation of apoptotic process	experimental (strong)	23	10.61	0.003
Gene Ontology - Biological Process	negative regulation of cell death	experimental (strong)	24	11.46	0.003
Gene Ontology - Biological Process	negative regulation of programmed cell death	experimental (strong)	23	10.69	0.003
Gene Ontology - Biological Process	phosphate-containing compound metabolic process	experimental (strong)	39	23.64	0.003
Gene Ontology - Biological Process	positive regulation of NF-kappaB transcription factor activity	experimental (strong)	9	2.039	0.003
Gene Ontology - Biological Process	positive regulation of T cell migration	experimental (strong)	4	0.284	0.003
Gene Ontology - Biological Process	positive regulation of gliogenesis	experimental (strong)	6	0.878	0.003

Gene Ontology - Biological Process	regulation of calcium ion transport	experimental (strong)	9	1.962	0.003
Gene Ontology - Biological Process	regulation of cell motility	experimental (strong)	22	9.757	0.003
Gene Ontology - Biological Process	regulation of cell-cell adhesion	experimental (strong)	14	4.646	0.003
Gene Ontology - Biological Process	regulation of immune system process	experimental (strong)	26	12.83	0.003
Gene Ontology - Biological Process	regulation of interleukin-12 biosynthetic process	experimental (strong)	3	0.129	0.003
Gene Ontology - Biological Process	regulation of nitrogen compound metabolic process	experimental (strong)	54	38.77	0.003
Gene Ontology - Biological Process	regulation of programmed cell death	experimental (strong)	31	16.73	0.003
Gene Ontology - Biological Process	ERK1 and ERK2 cascade	experimental (any)	11	3.215	0.003
Gene Ontology - Biological Process	MyD88-independent toll-like receptor signaling pathway	experimental (any)	4	0.385	0.003

Gene Ontology - Biological Process	Ras protein signal transduction	experimental (any)	12	3.84	0.003
Gene Ontology - Biological Process	adaptive immune response based on somatic recombination of immune receptors built from immunoglobulin superfamily domains	experimental (any)	10	2.764	0.003
Gene Ontology - Biological Process	aortic valve morphogenesis	experimental (any)	4	0.359	0.003
Gene Ontology - Biological Process	biomineral tissue development	experimental (any)	8	1.807	0.003
Gene Ontology - Biological Process	body fluid secretion	experimental (any)	6	1.023	0.003
Gene Ontology - Biological Process	cell cycle arrest	experimental (any)	10	2.737	0.003
Gene Ontology - Biological Process	cell migration involved in sprouting angiogenesis	experimental (any)	5	0.651	0.003
Gene Ontology - Biological Process	cellular extravasation	experimental (any)	5	0.624	0.003

Gene Ontology - Biological Process	chemokine (C-X-C motif) ligand 12 signaling pathway	experimental (any)	2	0.040	0.003
Gene Ontology - Biological Process	endothelium development	experimental (any)	7	1.342	0.003
Gene Ontology - Biological Process	epithelial cell proliferation	experimental (any)	13	4.478	0.003
Gene Ontology - Biological Process	fibroblast proliferation	experimental (any)	6	1.023	0.003
Gene Ontology - Biological Process	gliogenesis	experimental (any)	11	3.348	0.003
Gene Ontology - Biological Process	interferon-gamma-mediated signaling pathway	experimental (any)	6	1.023	0.003
Gene Ontology - Biological Process	interleukin-12 production	experimental (any)	5	0.651	0.003
Gene Ontology - Biological Process	leukocyte homeostasis	experimental (any)	6	0.983	0.003
Gene Ontology - Biological Process	lymphocyte homeostasis	experimental (any)	5	0.678	0.003
Gene Ontology - Biological Process	lymphocyte proliferation	experimental (any)	10	2.764	0.003
Gene Ontology - Biological Process	macrophage activation	experimental (any)	6	0.997	0.003

Gene Ontology - Biological Process	maintenance of permeability of blood-brain barrier	experimental (any)	2	0.040	0.003
Gene Ontology - Biological Process	mononuclear cell proliferation	experimental (any)	10	2.79	0.003
Gene Ontology - Biological Process	muscle cell proliferation	experimental (any)	9	2.299	0.003
Gene Ontology - Biological Process	negative regulation of blood vessel endothelial cell migration	experimental (any)	4	0.372	0.003
Gene Ontology - Biological Process	negative regulation of cellular component movement	experimental (any)	11	3.202	0.003
Gene Ontology - Biological Process	negative regulation of growth rate	experimental (any)	2	0.040	0.003
Gene Ontology - Biological Process	negative regulation of locomotion	experimental (any)	11	3.335	0.003
Gene Ontology - Biological Process	negative regulation of serotonin uptake	experimental (any)	2	0.040	0.003

Gene Ontology - Biological Process	neutrophil activation involved in immune response	experimental (any)	15	5.634	0.003
Gene Ontology - Biological Process	neutrophil degranulation	experimental (any)	15	5.607	0.003
Gene Ontology - Biological Process	nucleobase-containing compound metabolic process	experimental (any)	84	61.56	0.003
Gene Ontology - Biological Process	ossification	experimental (any)	13	4.478	0.003
Gene Ontology - Biological Process	outflow tract septum morphogenesis	experimental (any)	4	0.359	0.003
Gene Ontology - Biological Process	positive regulation of cellular response to macrophage colony-stimulating factor stimulus	experimental (any)	2	0.040	0.003

Gene Ontology - Biological Process	positive regulation of cytokine production involved in immune response	experimental (any)	5	0.624	0.003
Gene Ontology - Biological Process	positive regulation of extrinsic apoptotic signaling pathway via death domain receptors	experimental (any)	3	0.159	0.003
Gene Ontology - Biological Process	positive regulation of histone acetylation	experimental (any)	4	0.372	0.003
Gene Ontology - Biological Process	positive regulation of lymphocyte migration	experimental (any)	4	0.359	0.003
Gene Ontology - Biological Process	positive regulation of lymphocyte proliferation	experimental (any)	7	1.369	0.003
Gene Ontology - Biological Process	positive regulation of mononuclear cell proliferation	experimental (any)	7	1.382	0.003

Gene Ontology - Biological Process	positive regulation of nitric-oxide synthase biosynthetic process	experimental (any)	3	0.159	0.003
Gene Ontology - Biological Process	positive regulation of nucleotide-binding oligomerization domain containing 2 signaling pathway	experimental (any)	2	0.040	0.003
Gene Ontology - Biological Process	positive regulation of response to cytokine stimulus	experimental (any)	5	0.638	0.003
Gene Ontology - Biological Process	regulation of extrinsic apoptotic signaling pathway	experimental (any)	8	1.847	0.003
Gene Ontology - Biological Process	regulation of fibroblast proliferation	experimental (any)	6	1.01	0.003
Gene Ontology - Biological Process	regulation of interleukin-12 production	experimental (any)	5	0.624	0.003

Gene Ontology - Biological Process	regulation of vascular endothelial growth factor production	experimental (any)	4	0.385	0.003
Gene Ontology - Biological Process	response to phenylpropanoid	experimental (any)	2	0.040	0.003
Gene Ontology - Biological Process	stress-activated protein kinase signaling cascade	experimental (any)	11	3.255	0.003
Gene Ontology - Biological Process	telencephalon cell migration	experimental (any)	5	0.664	0.003
Gene Ontology - Biological Process	viral entry into host cell	experimental (any)	7	1.382	0.003
Gene Ontology - Biological Process	axon development	predicted (union)	193	152.3	0.004
Gene Ontology - Biological Process	cell morphogenesis	predicted (union)	359	302.6	0.004
Gene Ontology - Biological Process	cell morphogenesis involved in neuron differentiation	predicted (union)	215	171.8	0.004

Gene Ontology - Biological Process	negative regulation of nucleic acid-templated transcription	predicted (union)	451	387.7	0.004
Gene Ontology - Biological Process	neuron projection development	predicted (union)	359	303	0.004
Gene Ontology - Biological Process	cell cycle arrest	experimental (strong)	10	2.607	0.004
Gene Ontology - Biological Process	cell surface receptor signaling pathway	experimental (strong)	39	24.31	0.004
Gene Ontology - Biological Process	cellular response to interleukin-1	experimental (strong)	8	1.704	0.004
Gene Ontology - Biological Process	immune system process	experimental (strong)	36	21.68	0.004
Gene Ontology - Biological Process	neutrophil mediated immunity	experimental (strong)	11	3.149	0.004
Gene Ontology - Biological Process	positive regulation of immune response	experimental (strong)	16	6.195	0.004
Gene Ontology - Biological Process	positive regulation of macromolecule biosynthetic process	experimental (strong)	31	17.37	0.004

Gene Ontology - Biological Process	positive regulation of vasoconstriction	experimental (strong)	4	0.336	0.004
Gene Ontology - Biological Process	reactive oxygen species biosynthetic process	experimental (strong)	7	1.265	0.004
Gene Ontology - Biological Process	regulation of apoptotic process	experimental (strong)	30	16.6	0.004
Gene Ontology - Biological Process	regulation of cell migration	experimental (strong)	21	9.395	0.004
Gene Ontology - Biological Process	response to glucocorticoid	experimental (strong)	8	1.678	0.004
Gene Ontology - Biological Process	response to hypoxia	experimental (strong)	13	4.31	0.004
Gene Ontology - Biological Process	response to organonitrogen compound	experimental (strong)	21	9.602	0.004
Gene Ontology - Biological Process	viral process	experimental (strong)	17	6.788	0.004
Gene Ontology - Biological Process	SMAD protein complex assembly	experimental (any)	3	0.173	0.004
Gene Ontology - Biological Process	aortic valve development	experimental (any)	4	0.412	0.004

Gene Ontology - Biological Process	apoptotic signaling pathway	experimental (any)	17	7.029	0.004
Gene Ontology - Biological Process	blood vessel development	experimental (any)	18	7.613	0.004
Gene Ontology - Biological Process	bone resorption	experimental (any)	5	0.691	0.004
Gene Ontology - Biological Process	cellular response to prostaglandin E stimulus	experimental (any)	3	0.186	0.004
Gene Ontology - Biological Process	forebrain cell migration	experimental (any)	5	0.704	0.004
Gene Ontology - Biological Process	myeloid cell apoptotic process	experimental (any)	4	0.412	0.004
Gene Ontology - Biological Process	negative regulation of cell migration involved in sprouting angiogenesis	experimental (any)	3	0.173	0.004
Gene Ontology - Biological Process	negative regulation of chemotaxis	experimental (any)	4	0.412	0.004
Gene Ontology - Biological Process	negative regulation of cytokine production	experimental (any)	10	2.95	0.004

Gene Ontology - Biological Process	negative regulation of epithelial cell proliferation	experimental (any)	7	1.462	0.004
Gene Ontology - Biological Process	neuroinflammatory response	experimental (any)	5	0.704	0.004
Gene Ontology - Biological Process	neurotransmitter biosynthetic process	experimental (any)	6	1.036	0.004
Gene Ontology - Biological Process	positive regulation of cellular component biogenesis	experimental (any)	15	5.713	0.004
Gene Ontology - Biological Process	positive regulation of chemotaxis	experimental (any)	7	1.488	0.004
Gene Ontology - Biological Process	positive regulation of interleukin-12 production	experimental (any)	4	0.412	0.004
Gene Ontology - Biological Process	positive regulation of leukocyte proliferation	experimental (any)	7	1.435	0.004
Gene Ontology - Biological Process	positive regulation of peptidyl-lysine acetylation	experimental (any)	4	0.412	0.004

Gene Ontology - Biological Process	positive regulation of peptidyl-serine phosphorylation	experimental (any)	6	1.05	0.004
Gene Ontology - Biological Process	positive regulation of small GTPase mediated signal transduction	experimental (any)	5	0.718	0.004
Gene Ontology - Biological Process	regulation of actin filament-based process	experimental (any)	12	3.946	0.004
Gene Ontology - Biological Process	regulation of cell growth	experimental (any)	13	4.544	0.004
Gene Ontology - Biological Process	regulation of epithelial to mesenchymal transition	experimental (any)	6	1.036	0.004
Gene Ontology - Biological Process	regulation of nitric oxide biosynthetic process	experimental (any)	5	0.691	0.004
Gene Ontology - Biological Process	response to toxic substance	experimental (any)	15	5.727	0.004
Gene Ontology - Biological Process	tissue morphogenesis	experimental (any)	18	7.6	0.004

Gene Ontology - Biological Process	vascular endothelial growth factor production	experimental (any)	4	0.399	0.004
Gene Ontology - Biological Process	axon guidance	predicted (union)	101	73.02	0.005
Gene Ontology - Biological Process	negative regulation of RNA biosynthetic process	predicted (union)	451	388.5	0.005
Gene Ontology - Biological Process	I-kappaB kinase/NF-kappaB signaling	experimental (strong)	10	2.762	0.005
Gene Ontology - Biological Process	T cell migration	experimental (strong)	5	0.619	0.005
Gene Ontology - Biological Process	cellular response to chemokine	experimental (strong)	5	0.645	0.005
Gene Ontology - Biological Process	leukocyte chemotaxis	experimental (strong)	8	1.781	0.005
Gene Ontology - Biological Process	positive regulation of cell differentiation	experimental (strong)	22	10.4	0.005
Gene Ontology - Biological Process	regulation of actin cytoskeleton organization	experimental (strong)	10	2.762	0.005
Gene Ontology - Biological Process	regulation of catalytic activity	experimental (strong)	31	17.68	0.005

Gene Ontology - Biological Process	regulation of cellular component movement	experimental (strong)	22	10.4	0.005
Gene Ontology - Biological Process	regulation of interleukin-23 production	experimental (strong)	3	0.155	0.005
Gene Ontology - Biological Process	regulation of locomotion	experimental (strong)	22	10.4	0.005
Gene Ontology - Biological Process	regulation of reactive oxygen species metabolic process	experimental (strong)	9	2.271	0.005
Gene Ontology - Biological Process	response to interleukin-6	experimental (strong)	5	0.619	0.005
Gene Ontology - Biological Process	T cell proliferation	experimental (any)	8	1.993	0.005
Gene Ontology - Biological Process	cardiac chamber development	experimental (any)	8	2.006	0.005
Gene Ontology - Biological Process	cell differentiation	experimental (any)	60	41.22	0.005
Gene Ontology - Biological Process	cell proliferation involved in heart valve development	experimental (any)	2	0.053	0.005

Gene Ontology - Biological Process	cellular response to organic cyclic compound	experimental (any)	16	6.484	0.005
Gene Ontology - Biological Process	developmental process	experimental (any)	85	63.82	0.005
Gene Ontology - Biological Process	endothelial cell differentiation	experimental (any)	6	1.129	0.005
Gene Ontology - Biological Process	leukocyte proliferation	experimental (any)	10	3.029	0.005
Gene Ontology - Biological Process	membrane protein intracellular domain proteolysis	experimental (any)	3	0.199	0.005
Gene Ontology - Biological Process	negative regulation of mammary gland epithelial cell proliferation	experimental (any)	2	0.053	0.005
Gene Ontology - Biological Process	neurotransmitter metabolic process	experimental (any)	7	1.555	0.005
Gene Ontology - Biological Process	positive regulation of adaptive immune response	experimental (any)	6	1.116	0.005

Gene Ontology - Biological Process	positive regulation of inflammatory response	experimental (any)	7	1.555	0.005
Gene Ontology - Biological Process	positive regulation of interleukin-12 biosynthetic process	experimental (any)	2	0.053	0.005
Gene Ontology - Biological Process	positive regulation of superoxide anion generation	experimental (any)	3	0.199	0.005
Gene Ontology - Biological Process	regulation of T cell migration	experimental (any)	4	0.438	0.005
Gene Ontology - Biological Process	regulation of cytokine biosynthetic process	experimental (any)	6	1.103	0.005
Gene Ontology - Biological Process	regulation of focal adhesion assembly	experimental (any)	5	0.757	0.005
Gene Ontology - Biological Process	regulation of kinase activity	experimental (any)	20	9.141	0.005
Gene Ontology - Biological Process	regulation of mammary gland epithelial cell proliferation	experimental (any)	3	0.199	0.005

Gene Ontology - Biological Process	negative regulation of translation	predicted (union)	60	39.44	0.006
Gene Ontology - Biological Process	neuron development	predicted (union)	404	345.9	0.006
Gene Ontology - Biological Process	chemotaxis	experimental (strong)	15	5.833	0.006
Gene Ontology - Biological Process	nitric oxide metabolic process	experimental (strong)	6	1.032	0.006
Gene Ontology - Biological Process	positive regulation of T cell activation	experimental (strong)	9	2.349	0.006
Gene Ontology - Biological Process	positive regulation of cell migration	experimental (strong)	16	6.479	0.006
Gene Ontology - Biological Process	positive regulation of cell motility	experimental (strong)	16	6.504	0.006
Gene Ontology - Biological Process	positive regulation of leukocyte proliferation	experimental (strong)	7	1.445	0.006
Gene Ontology - Biological Process	positive regulation of lymphocyte migration	experimental (strong)	4	0.387	0.006

Gene Ontology - Biological Process	positive regulation of lymphocyte proliferation	experimental (strong)	7	1.42	0.006
Gene Ontology - Biological Process	positive regulation of mononuclear cell proliferation	experimental (strong)	7	1.445	0.006
Gene Ontology - Biological Process	regulation of T cell migration	experimental (strong)	4	0.387	0.006
Gene Ontology - Biological Process	response to corticosteroid	experimental (strong)	8	1.884	0.006
Gene Ontology - Biological Process	cardiovascular system development	experimental (any)	18	8.052	0.006
Gene Ontology - Biological Process	cell-substrate junction assembly	experimental (any)	6	1.196	0.006
Gene Ontology - Biological Process	embryonic morphogenesis	experimental (any)	16	6.644	0.006
Gene Ontology - Biological Process	heterocycle metabolic process	experimental (any)	84	63.37	0.006
Gene Ontology - Biological Process	leukocyte apoptotic process	experimental (any)	6	1.183	0.006
Gene Ontology - Biological Process	leukocyte degranulation	experimental (any)	15	6.085	0.006

Gene Ontology - Biological Process	negative regulation of biosynthetic process	experimental (any)	30	16.61	0.006
Gene Ontology - Biological Process	negative regulation of extrinsic apoptotic signaling pathway via death domain receptors	experimental (any)	4	0.465	0.006
Gene Ontology - Biological Process	negative regulation of myeloid cell apoptotic process	experimental (any)	3	0.213	0.006
Gene Ontology - Biological Process	pericardium development	experimental (any)	3	0.213	0.006
Gene Ontology - Biological Process	positive regulation of ERK1 and ERK2 cascade	experimental (any)	8	2.059	0.006
Gene Ontology - Biological Process	positive regulation of T cell mediated immunity	experimental (any)	4	0.478	0.006
Gene Ontology - Biological Process	positive regulation of interleukin-8 production	experimental (any)	4	0.465	0.006

Gene Ontology - Biological Process	pulmonary valve morphogenesis	experimental (any)	3	0.213	0.006
Gene Ontology - Biological Process	regulation of catalytic activity	experimental (any)	39	23.66	0.006
Gene Ontology - Biological Process	regulation of cell migration involved in sprouting angiogenesis	experimental (any)	4	0.465	0.006
Gene Ontology - Biological Process	regulation of immunoglobulin secretion	experimental (any)	3	0.213	0.006
Gene Ontology - Biological Process	regulation of neurogenesis	experimental (any)	19	8.743	0.006
Gene Ontology - Biological Process	regulation of smooth muscle cell proliferation	experimental (any)	7	1.634	0.006
Gene Ontology - Biological Process	vasculature development	experimental (any)	18	7.946	0.006
Gene Ontology - Biological Process	negative regulation of biosynthetic process	predicted (intersection)	278	223.3	0.007
Gene Ontology - Biological Process	regulation of cell differentiation	predicted (intersection)	333	273	0.007

Gene Ontology - Biological Process	cell development	predicted (union)	753	675.1	0.007
Gene Ontology - Biological Process	cell morphogenesis involved in differentiation	predicted (union)	266	219.8	0.007
Gene Ontology - Biological Process	neuron projection guidance	predicted (union)	101	73.8	0.007
Gene Ontology - Biological Process	positive regulation of macromolecule biosynthetic process	predicted (union)	680	606.8	0.007
Gene Ontology - Biological Process	regulation of cellular protein localization	predicted (union)	228	185.5	0.007
Gene Ontology - Biological Process	tube morphogenesis	predicted (union)	336	284.3	0.007
Gene Ontology - Biological Process	SMAD protein complex assembly	experimental (strong)	3	0.181	0.007
Gene Ontology - Biological Process	chemokine (C-X-C motif) ligand 12 signaling pathway	experimental (strong)	2	0.052	0.007
Gene Ontology - Biological Process	defense response	experimental (strong)	23	11.69	0.007

Gene Ontology - Biological Process	inflammatory response	experimental (strong)	15	5.937	0.007
Gene Ontology - Biological Process	maintenance of permeability of blood-brain barrier	experimental (strong)	2	0.052	0.007
Gene Ontology - Biological Process	negative regulation of calcium ion transport	experimental (strong)	4	0.413	0.007
Gene Ontology - Biological Process	negative regulation of extracellular matrix organization	experimental (strong)	2	0.052	0.007
Gene Ontology - Biological Process	negative regulation of growth rate	experimental (strong)	2	0.052	0.007
Gene Ontology - Biological Process	negative regulation of interleukin-23 production	experimental (strong)	2	0.052	0.007
Gene Ontology - Biological Process	negative regulation of signal transduction	experimental (strong)	23	11.67	0.007
Gene Ontology - Biological Process	positive regulation of cell death	experimental (strong)	18	8.053	0.007

Gene Ontology - Biological Process	positive regulation of cellular component movement	experimental (strong)	16	6.556	0.007
Gene Ontology - Biological Process	positive regulation of cellular extravasation	experimental (strong)	2	0.052	0.007
Gene Ontology - Biological Process	positive regulation of chemotaxis	experimental (strong)	7	1.497	0.007
Gene Ontology - Biological Process	positive regulation of locomotion	experimental (strong)	16	6.659	0.007
Gene Ontology - Biological Process	prostaglandin biosynthetic process	experimental (strong)	3	0.181	0.007
Gene Ontology - Biological Process	regulation of T cell proliferation	experimental (strong)	7	1.523	0.007
Gene Ontology - Biological Process	regulation of cell differentiation	experimental (strong)	30	17.42	0.007
Gene Ontology - Biological Process	regulation of growth rate	experimental (strong)	2	0.052	0.007
Gene Ontology - Biological Process	regulation of leukocyte proliferation	experimental (strong)	9	2.4	0.007

Gene Ontology - Biological Process	regulation of reactive oxygen species biosynthetic process	experimental (strong)	6	1.058	0.007
Gene Ontology - Biological Process	response to alkaloid	experimental (strong)	6	1.084	0.007
Gene Ontology - Biological Process	response to nitrogen compound	experimental (strong)	21	10.04	0.007
Gene Ontology - Biological Process	response to ultrasound	experimental (strong)	2	0.052	0.007
Gene Ontology - Biological Process	secretion by cell	experimental (strong)	22	10.97	0.007
Gene Ontology - Biological Process	signal transduction	experimental (strong)	51	37.5	0.007
Gene Ontology - Biological Process	ameboidal-type cell migration	experimental (any)	12	4.332	0.007
Gene Ontology - Biological Process	anatomical structure morphogenesis	experimental (any)	44	28.05	0.007
Gene Ontology - Biological Process	angiogenesis	experimental (any)	14	5.567	0.007
Gene Ontology - Biological Process	epithelium development	experimental (any)	25	13.17	0.007

Gene Ontology - Biological Process	gland development	experimental (any)	13	4.956	0.007
Gene Ontology - Biological Process	mammary gland development	experimental (any)	7	1.661	0.007
Gene Ontology - Biological Process	multicellular organism development	experimental (any)	75	55.29	0.007
Gene Ontology - Biological Process	muscle tissue development	experimental (any)	12	4.305	0.007
Gene Ontology - Biological Process	positive regulation of nitric oxide biosynthetic process	experimental (any)	4	0.492	0.007
Gene Ontology - Biological Process	positive regulation of protein acetylation	experimental (any)	4	0.492	0.007
Gene Ontology - Biological Process	regulation of cytoskeleton organization	experimental (any)	14	5.554	0.007
Gene Ontology - Biological Process	regulation of growth	experimental (any)	17	7.441	0.007
Gene Ontology - Biological Process	response to alkaloid	experimental (any)	6	1.209	0.007
Gene Ontology - Biological Process	smooth muscle cell proliferation	experimental (any)	7	1.648	0.007

Gene Ontology - Biological Process	stress-activated MAPK cascade	experimental (any)	10	3.162	0.007
Gene Ontology - Biological Process	establishment of localization in cell	predicted (union)	746	670	0.008
Gene Ontology - Biological Process	bone remodeling	experimental (strong)	6	1.136	0.008
Gene Ontology - Biological Process	neutrophil activation involved in immune response	experimental (strong)	10	3.046	0.008
Gene Ontology - Biological Process	neutrophil degranulation	experimental (strong)	10	3.046	0.008
Gene Ontology - Biological Process	nucleobase-containing compound metabolic process	experimental (strong)	46	32.39	0.008
Gene Ontology - Biological Process	positive regulation of cell activation	experimental (strong)	11	3.639	0.008
Gene Ontology - Biological Process	positive regulation of protein kinase activity	experimental (strong)	15	6.066	0.008

Gene Ontology - Biological Process	positive regulation of protein serine/threonine kinase activity	experimental (strong)	12	4.181	0.008
Gene Ontology - Biological Process	regulation of cytokine production	experimental (strong)	16	6.788	0.008
Gene Ontology - Biological Process	Fc receptor signaling pathway	experimental (any)	8	2.232	0.008
Gene Ontology - Biological Process	activation of protein kinase activity	experimental (any)	11	3.853	0.008
Gene Ontology - Biological Process	animal organ regeneration	experimental (any)	5	0.877	0.008
Gene Ontology - Biological Process	cellular aromatic compound metabolic process	experimental (any)	84	63.8	0.008
Gene Ontology - Biological Process	cellular response to oxygen levels	experimental (any)	9	2.75	0.008
Gene Ontology - Biological Process	glial cell development	experimental (any)	6	1.289	0.008
Gene Ontology - Biological Process	interleukin-6-mediated signaling pathway	experimental (any)	3	0.239	0.008

Gene Ontology - Biological Process	intracellular receptor signaling pathway	experimental (any)	10	3.269	0.008
Gene Ontology - Biological Process	mammary gland epithelium development	experimental (any)	5	0.850	0.008
Gene Ontology - Biological Process	membrane to membrane docking	experimental (any)	2	0.066	0.008
Gene Ontology - Biological Process	muscle tissue morphogenesis	experimental (any)	5	0.877	0.008
Gene Ontology - Biological Process	necroptotic signaling pathway	experimental (any)	2	0.066	0.008
Gene Ontology - Biological Process	negative regulation of apoptotic signaling pathway	experimental (any)	9	2.737	0.008
Gene Ontology - Biological Process	negative regulation of growth	experimental (any)	9	2.737	0.008
Gene Ontology - Biological Process	phosphate-containing compound metabolic process	experimental (any)	52	35.18	0.008

Gene Ontology - Biological Process	positive regulation of T cell cytokine production	experimental (any)	3	0.239	0.008
Gene Ontology - Biological Process	positive regulation of cell cycle	experimental (any)	12	4.398	0.008
Gene Ontology - Biological Process	positive regulation of interferon-alpha production	experimental (any)	3	0.239	0.008
Gene Ontology - Biological Process	positive regulation of nitric oxide metabolic process	experimental (any)	4	0.505	0.008
Gene Ontology - Biological Process	regulation of adaptive immune response	experimental (any)	7	1.727	0.008
Gene Ontology - Biological Process	regulation of glial cell differentiation	experimental (any)	5	0.877	0.008
Gene Ontology - Biological Process	regulation of growth rate	experimental (any)	2	0.066	0.008
Gene Ontology - Biological Process	regulation of ossification	experimental (any)	8	2.219	0.008
Gene Ontology - Biological Process	regulation of superoxide anion generation	experimental (any)	3	0.239	0.008

Gene Ontology - Biological Process	response to radiation	experimental (any)	13	5.076	0.008
Gene Ontology - Biological Process	response to tumor necrosis factor	experimental (any)	10	3.229	0.008
Gene Ontology - Biological Process	serotonin uptake	experimental (any)	2	0.066	0.008
Gene Ontology - Biological Process	cell morphogenesis	predicted (intersection)	182	138.7	0.009
Gene Ontology - Biological Process	response to platelet-derived growth factor	predicted (intersection)	12	3.579	0.009
Gene Ontology - Biological Process	tricuspid valve development	predicted (intersection)	6	1.074	0.009
Gene Ontology - Biological Process	cellular localization	predicted (union)	977	891.8	0.009
Gene Ontology - Biological Process	cell-cell adhesion	experimental (strong)	16	6.866	0.009
Gene Ontology - Biological Process	mononuclear cell migration	experimental (strong)	5	0.774	0.009
Gene Ontology - Biological Process	positive regulation of smooth muscle cell proliferation	experimental (strong)	7	1.6	0.009

Gene Ontology - Biological Process	regulation of interleukin-12 production	experimental (strong)	5	0.774	0.009
Gene Ontology - Biological Process	response to external biotic stimulus	experimental (strong)	21	10.43	0.009
Gene Ontology - Biological Process	response to interleukin-1	experimental (strong)	8	2.065	0.009
Gene Ontology - Biological Process	response to other organism	experimental (strong)	21	10.43	0.009
Gene Ontology - Biological Process	B cell activation involved in immune response	experimental (any)	5	0.890	0.009
Gene Ontology - Biological Process	positive regulation of catalytic activity	experimental (any)	27	14.92	0.009
Gene Ontology - Biological Process	positive regulation of glial cell differentiation	experimental (any)	4	0.545	0.009
Gene Ontology - Biological Process	positive regulation of protein secretion	experimental (any)	9	2.764	0.009
Gene Ontology - Biological Process	response to prostaglandin E	experimental (any)	3	0.252	0.009
Gene Ontology - Biological Process	vesicle-mediated transport	experimental (any)	35	21.15	0.009

Gene Ontology - Biological Process	adaptive immune response based on somatic recombination of immune receptors built from immunoglobulin superfamily domains	experimental (strong)	8	2.117	0.010
Gene Ontology - Biological Process	glial cell differentiation	experimental (strong)	9	2.633	0.010
Gene Ontology - Biological Process	interleukin-12 production	experimental (strong)	5	0.800	0.010
Gene Ontology - Biological Process	positive regulation of histone acetylation	experimental (strong)	4	0.465	0.010
Gene Ontology - Biological Process	positive regulation of protein complex assembly	experimental (strong)	8	2.091	0.010
Gene Ontology - Biological Process	proteolysis	experimental (strong)	21	10.58	0.010
Gene Ontology - Biological Process	regulation of RNA metabolic process	experimental (strong)	40	26.87	0.010

Gene Ontology - Biological Process	regulation of actin filament-based process	experimental (strong)	10	3.149	0.010
Gene Ontology - Biological Process	regulation of metabolic process	experimental (strong)	55	42.43	0.010
Gene Ontology - Biological Process	toll-like receptor signaling pathway	experimental (strong)	7	1.626	0.010
Gene Ontology - Biological Process	DNA damage response, signal transduction by p53 class mediator	experimental (any)	6	1.355	0.010
Gene Ontology - Biological Process	blood vessel endothelial cell migration	experimental (any)	6	1.342	0.010
Gene Ontology - Biological Process	cellular response to prostaglandin stimulus	experimental (any)	3	0.266	0.010
Gene Ontology - Biological Process	cytokine secretion	experimental (any)	8	2.325	0.010
Gene Ontology - Biological Process	exocytosis	experimental (any)	20	9.899	0.010
Gene Ontology - Biological Process	head development	experimental (any)	18	8.57	0.010

Gene Ontology - Biological Process	homeostasis of number of cells	experimental (any)	9	2.87	0.010
Gene Ontology - Biological Process	immunoglobulin secretion	experimental (any)	3	0.266	0.010
Gene Ontology - Biological Process	inflammatory cell apoptotic process	experimental (any)	3	0.266	0.010
Gene Ontology - Biological Process	lymphocyte migration	experimental (any)	5	0.930	0.010
Gene Ontology - Biological Process	macromolecule metabolic process	experimental (any)	118	97.53	0.010
Gene Ontology - Biological Process	necrotic cell death	experimental (any)	4	0.558	0.010
Gene Ontology - Biological Process	negative regulation of endothelial cell migration	experimental (any)	4	0.558	0.010
Gene Ontology - Biological Process	negative regulation of transmembrane receptor protein serine/threonine kinase signaling pathway	experimental (any)	6	1.329	0.010
Gene Ontology - Biological Process	peptidyl-tyrosine phosphorylation	experimental (any)	11	3.973	0.010

Gene Ontology - Biological Process	positive regulation of leukocyte chemotaxis	experimental (any)	5	0.917	0.010
Gene Ontology - Biological Process	positive regulation of mononuclear cell migration	experimental (any)	3	0.266	0.010
Gene Ontology - Biological Process	prostaglandin biosynthetic process	experimental (any)	3	0.266	0.010
Gene Ontology - Biological Process	protein-containing complex assembly	experimental (any)	32	18.93	0.010
Gene Ontology - Biological Process	pulmonary valve development	experimental (any)	3	0.266	0.010
Gene Ontology - Biological Process	regulation of monocyte chemotaxis	experimental (any)	3	0.266	0.010
Gene Ontology - Biological Process	wound healing	experimental (any)	14	5.82	0.010
Gene Ontology - Biological Process	cell migration	experimental (strong)	25	13.78	0.011
Gene Ontology - Biological Process	cellular extravasation	experimental (strong)	4	0.490	0.011

Gene Ontology - Biological Process	heterocycle metabolic process	experimental (strong)	46	32.91	0.011
Gene Ontology - Biological Process	membrane protein intracellular domain proteolysis	experimental (strong)	3	0.232	0.011
Gene Ontology - Biological Process	positive regulation of glial cell differentiation	experimental (strong)	4	0.490	0.011
Gene Ontology - Biological Process	positive regulation of peptidyl-serine phosphorylation	experimental (strong)	6	1.213	0.011
Gene Ontology - Biological Process	positive regulation of reactive oxygen species metabolic process	experimental (strong)	6	1.239	0.011
Gene Ontology - Biological Process	positive regulation of vascular endothelial growth factor production	experimental (strong)	4	0.490	0.011
Gene Ontology - Biological Process	regulation of glial cell differentiation	experimental (strong)	5	0.826	0.011

Gene Ontology - Biological Process	regulation of immunoglobulin secretion	experimental (strong)	3	0.232	0.011
Gene Ontology - Biological Process	regulation of lymphocyte activation	experimental (strong)	12	4.44	0.011
Gene Ontology - Biological Process	regulation of monocyte chemotaxis	experimental (strong)	3	0.232	0.011
Gene Ontology - Biological Process	response to organic cyclic compound	experimental (strong)	20	9.989	0.011
Gene Ontology - Biological Process	SMAD protein signal transduction	experimental (any)	4	0.585	0.011
Gene Ontology - Biological Process	atrioventricular valve formation	experimental (any)	2	0.080	0.011
Gene Ontology - Biological Process	cardiac septum development	experimental (any)	6	1.395	0.011
Gene Ontology - Biological Process	common-partner SMAD protein phosphorylation	experimental (any)	2	0.080	0.011
Gene Ontology - Biological Process	endocardial cell differentiation	experimental (any)	2	0.080	0.011
Gene Ontology - Biological Process	endothelial cell migration	experimental (any)	8	2.352	0.011

Gene Ontology - Biological Process	gene expression	experimental (any)	77	58.17	0.011
Gene Ontology - Biological Process	granulocyte chemotaxis	experimental (any)	5	0.943	0.011
Gene Ontology - Biological Process	heart morphogenesis	experimental (any)	9	2.883	0.011
Gene Ontology - Biological Process	lactation	experimental (any)	4	0.571	0.011
Gene Ontology - Biological Process	mesenchymal cell differentiation	experimental (any)	8	2.378	0.011
Gene Ontology - Biological Process	muscle organ morphogenesis	experimental (any)	5	0.943	0.011
Gene Ontology - Biological Process	negative regulation of extracellular matrix organization	experimental (any)	2	0.080	0.011
Gene Ontology - Biological Process	negative regulation of macrophage apoptotic process	experimental (any)	2	0.080	0.011
Gene Ontology - Biological Process	positive regulation of T-helper 2 cell cytokine production	experimental (any)	2	0.080	0.011

Gene Ontology - Biological Process	positive regulation of extrinsic apoptotic signaling pathway	experimental (any)	4	0.571	0.011
Gene Ontology - Biological Process	positive regulation of lipopolysaccharide-mediated signaling pathway	experimental (any)	2	0.080	0.011
Gene Ontology - Biological Process	positive regulation of peptide secretion	experimental (any)	9	2.923	0.011
Gene Ontology - Biological Process	positive regulation of protein localization to membrane	experimental (any)	6	1.395	0.011
Gene Ontology - Biological Process	positive regulation of wound healing	experimental (any)	4	0.571	0.011
Gene Ontology - Biological Process	regulation of blood vessel diameter	experimental (any)	6	1.382	0.011
Gene Ontology - Biological Process	regulation of bone remodeling	experimental (any)	4	0.571	0.011
Gene Ontology - Biological Process	regulation of cellular extravasation	experimental (any)	3	0.279	0.011

Gene Ontology - Biological Process	regulation of inflammatory response	experimental (any)	10	3.455	0.011
Gene Ontology - Biological Process	response to UV-A	experimental (any)	2	0.080	0.011
Gene Ontology - Biological Process	trabecula morphogenesis	experimental (any)	4	0.571	0.011
Gene Ontology - Biological Process	ventricular cardiac muscle tissue morphogenesis	experimental (any)	4	0.571	0.011
Gene Ontology - Biological Process	cellular response to hormone stimulus	predicted (intersection)	138	101.3	0.012
Gene Ontology - Biological Process	calcium ion transport	experimental (strong)	9	2.736	0.012
Gene Ontology - Biological Process	cell chemotaxis	experimental (strong)	9	2.736	0.012
Gene Ontology - Biological Process	cellular aromatic compound metabolic process	experimental (strong)	46	33.14	0.012
Gene Ontology - Biological Process	cytokine production involved in immune response	experimental (strong)	6	1.265	0.012

Gene Ontology - Biological Process	negative regulation of blood vessel endothelial cell migration	experimental (strong)	4	0.516	0.012
Gene Ontology - Biological Process	positive regulation of kinase activity	experimental (strong)	15	6.427	0.012
Gene Ontology - Biological Process	protein autoubiquitination	experimental (strong)	4	0.516	0.012
Gene Ontology - Biological Process	regulation of blood vessel diameter	experimental (strong)	6	1.265	0.012
Gene Ontology - Biological Process	regulation of vascular endothelial growth factor production	experimental (strong)	4	0.516	0.012
Gene Ontology - Biological Process	response to organic substance	experimental (strong)	41	28.19	0.012
Gene Ontology - Biological Process	vascular endothelial growth factor production	experimental (strong)	4	0.516	0.012
Gene Ontology - Biological Process	cellular response to hypoxia	experimental (any)	8	2.418	0.012
Gene Ontology - Biological Process	gonad development	experimental (any)	8	2.418	0.012

Gene Ontology - Biological Process	negative regulation of sprouting angiogenesis	experimental (any)	3	0.292	0.012
Gene Ontology - Biological Process	outflow tract morphogenesis	experimental (any)	5	0.983	0.012
Gene Ontology - Biological Process	positive regulation of cell growth	experimental (any)	7	1.913	0.012
Gene Ontology - Biological Process	positive regulation of chemokine production	experimental (any)	4	0.598	0.012
Gene Ontology - Biological Process	positive regulation of growth	experimental (any)	9	2.936	0.012
Gene Ontology - Biological Process	regulation of BMP signaling pathway	experimental (any)	5	0.970	0.012
Gene Ontology - Biological Process	regulation of cysteine-type endopeptidase activity involved in apoptotic process	experimental (any)	8	2.392	0.012
Gene Ontology - Biological Process	regulation of gliogenesis	experimental (any)	6	1.422	0.012

Gene Ontology - Biological Process	regulation of interferon-beta production	experimental (any)	4	0.598	0.012
Gene Ontology - Biological Process	regulation of peptidyl-serine phosphorylation	experimental (any)	6	1.422	0.012
Gene Ontology - Biological Process	regulation of stress fiber assembly	experimental (any)	5	0.970	0.012
Gene Ontology - Biological Process	response to interferon-beta	experimental (any)	3	0.292	0.012
Gene Ontology - Biological Process	striated muscle tissue development	experimental (any)	11	4.132	0.012
Gene Ontology - Biological Process	B cell activation involved in immune response	experimental (strong)	5	0.878	0.013
Gene Ontology - Biological Process	interleukin-1-mediated signaling pathway	experimental (strong)	5	0.878	0.013
Gene Ontology - Biological Process	negative regulation of signaling	experimental (strong)	23	12.47	0.013
Gene Ontology - Biological Process	positive regulation of leukocyte chemotaxis	experimental (strong)	5	0.878	0.013

Gene Ontology - Biological Process	positive regulation of transcription, DNA-templated	experimental (strong)	26	14.87	0.013
Gene Ontology - Biological Process	response to drug	experimental (strong)	19	9.395	0.013
Gene Ontology - Biological Process	Fc-epsilon receptor signaling pathway	experimental (any)	6	1.462	0.013
Gene Ontology - Biological Process	Roundabout signaling pathway	experimental (any)	2	0.093	0.013
Gene Ontology - Biological Process	brain development	experimental (any)	17	8.105	0.013
Gene Ontology - Biological Process	cellular response to tumor necrosis factor	experimental (any)	9	3.003	0.013
Gene Ontology - Biological Process	development of primary sexual characteristics	experimental (any)	8	2.485	0.013
Gene Ontology - Biological Process	endocardial cushion formation	experimental (any)	3	0.306	0.013
Gene Ontology - Biological Process	focal adhesion assembly	experimental (any)	5	0.997	0.013
Gene Ontology - Biological Process	interferon-beta production	experimental (any)	4	0.624	0.013

Gene Ontology - Biological Process	interleukin-10 production	experimental (any)	4	0.611	0.013
Gene Ontology - Biological Process	mesenchyme development	experimental (any)	9	2.99	0.013
Gene Ontology - Biological Process	negative regulation of biomineral tissue development	experimental (any)	3	0.306	0.013
Gene Ontology - Biological Process	negative regulation of cellular metabolic process	experimental (any)	41	26.79	0.013
Gene Ontology - Biological Process	negative regulation of chemokine-mediated signaling pathway	experimental (any)	2	0.093	0.013
Gene Ontology - Biological Process	negative regulation of ion transport	experimental (any)	6	1.462	0.013
Gene Ontology - Biological Process	pericardium morphogenesis	experimental (any)	2	0.093	0.013
Gene Ontology - Biological Process	positive regulation of Ras protein signal transduction	experimental (any)	4	0.624	0.013

Gene Ontology - Biological Process	positive regulation of T cell proliferation	experimental (any)	5	0.997	0.013
Gene Ontology - Biological Process	positive regulation of fibroblast proliferation	experimental (any)	4	0.611	0.013
Gene Ontology - Biological Process	receptor signaling pathway via JAK-STAT	experimental (any)	6	1.448	0.013
Gene Ontology - Biological Process	regulated exocytosis	experimental (any)	18	8.823	0.013
Gene Ontology - Biological Process	regulation of MyD88-independent toll-like receptor signaling pathway	experimental (any)	2	0.093	0.013
Gene Ontology - Biological Process	regulation of blood vessel size	experimental (any)	6	1.435	0.013
Gene Ontology - Biological Process	regulation of interleukin-6 production	experimental (any)	6	1.435	0.013
Gene Ontology - Biological Process	regulation of lymphocyte differentiation	experimental (any)	7	1.927	0.013

Gene Ontology - Biological Process	regulation of neural precursor cell proliferation	experimental (any)	5	1.01	0.013
Gene Ontology - Biological Process	regulation of pro-B cell differentiation	experimental (any)	2	0.093	0.013
Gene Ontology - Biological Process	regulation of tube size	experimental (any)	6	1.448	0.013
Gene Ontology - Biological Process	response to fatty acid	experimental (any)	5	0.997	0.013
Gene Ontology - Biological Process	central nervous system development	predicted (union)	382	329.2	0.014
Gene Ontology - Biological Process	enzyme linked receptor protein signaling pathway	predicted (union)	385	331.9	0.014
Gene Ontology - Biological Process	positive regulation of cellular biosynthetic process	predicted (union)	706	634.9	0.014
Gene Ontology - Biological Process	RNA metabolic process	experimental (strong)	41	28.47	0.014
Gene Ontology - Biological Process	cell motility	experimental (strong)	25	14.22	0.014

Gene Ontology - Biological Process	cytokine production	experimental (strong)	16	7.33	0.014
Gene Ontology - Biological Process	divalent metal ion transport	experimental (strong)	9	2.839	0.014
Gene Ontology - Biological Process	innate immune response	experimental (strong)	15	6.582	0.014
Gene Ontology - Biological Process	positive regulation of apoptotic process	experimental (strong)	16	7.356	0.014
Gene Ontology - Biological Process	positive regulation of cell cycle	experimental (strong)	11	4.001	0.014
Gene Ontology - Biological Process	positive regulation of epithelial to mesenchymal transition	experimental (strong)	5	0.903	0.014
Gene Ontology - Biological Process	positive regulation of mononuclear cell migration	experimental (strong)	3	0.258	0.014
Gene Ontology - Biological Process	positive regulation of peptidyl-lysine acetylation	experimental (strong)	4	0.542	0.014
Gene Ontology - Biological Process	positive regulation of type I interferon production	experimental (strong)	5	0.903	0.014

Gene Ontology - Biological Process	prostaglandin metabolic process	experimental (strong)	3	0.258	0.014
Gene Ontology - Biological Process	regulation of ERK1 and ERK2 cascade	experimental (strong)	10	3.433	0.014
Gene Ontology - Biological Process	regulation of T cell activation	experimental (strong)	10	3.407	0.014
Gene Ontology - Biological Process	regulation of peptidyl-tyrosine phosphorylation	experimental (strong)	9	2.813	0.014
Gene Ontology - Biological Process	response to hormone	experimental (strong)	19	9.55	0.014
Gene Ontology - Biological Process	toll-like receptor 9 signaling pathway	experimental (strong)	3	0.258	0.014
Gene Ontology - Biological Process	ventricular cardiac muscle tissue morphogenesis	experimental (strong)	4	0.542	0.014
Gene Ontology - Biological Process	T cell mediated immunity	experimental (any)	5	1.023	0.014
Gene Ontology - Biological Process	aging	experimental (any)	10	3.641	0.014
Gene Ontology - Biological Process	endoderm formation	experimental (any)	4	0.638	0.014

Gene Ontology - Biological Process	extracellular matrix assembly	experimental (any)	3	0.319	0.014
Gene Ontology - Biological Process	positive regulation of cell cycle phase transition	experimental (any)	5	1.036	0.014
Gene Ontology - Biological Process	positive regulation of focal adhesion assembly	experimental (any)	3	0.319	0.014
Gene Ontology - Biological Process	positive regulation of myeloid leukocyte differentiation	experimental (any)	4	0.638	0.014
Gene Ontology - Biological Process	regulation of intrinsic apoptotic signaling pathway	experimental (any)	7	1.966	0.014
Gene Ontology - Biological Process	regulation of lymphocyte migration	experimental (any)	4	0.638	0.014
Gene Ontology - Biological Process	regulation of supramolecular fiber organization	experimental (any)	10	3.627	0.014
Gene Ontology - Biological Process	regulation of vasoconstriction	experimental (any)	4	0.638	0.014
Gene Ontology - Biological Process	activation of protein kinase activity	experimental (strong)	11	4.104	0.015

Gene Ontology - Biological Process	biosynthetic process	experimental (strong)	48	35.88	0.015
Gene Ontology - Biological Process	cellular nitrogen compound metabolic process	experimental (strong)	47	34.74	0.015
Gene Ontology - Biological Process	cyclooxygenase pathway	experimental (strong)	2	0.077	0.015
Gene Ontology - Biological Process	divalent inorganic cation transport	experimental (strong)	9	2.891	0.015
Gene Ontology - Biological Process	gliogenesis	experimental (strong)	10	3.485	0.015
Gene Ontology - Biological Process	lymphocyte migration	experimental (strong)	5	0.929	0.015
Gene Ontology - Biological Process	lymphocyte proliferation	experimental (strong)	9	2.942	0.015
Gene Ontology - Biological Process	monocyte chemotaxis	experimental (strong)	4	0.568	0.015
Gene Ontology - Biological Process	necrotic cell death	experimental (strong)	4	0.568	0.015
Gene Ontology - Biological Process	neurotransmitter metabolic process	experimental (strong)	6	1.342	0.015

Gene Ontology - Biological Process	nitrogen compound metabolic process	experimental (strong)	61	50.56	0.015
Gene Ontology - Biological Process	positive regulation of DNA-binding transcription factor activity	experimental (strong)	10	3.51	0.015
Gene Ontology - Biological Process	positive regulation of ERK1 and ERK2 cascade	experimental (strong)	8	2.375	0.015
Gene Ontology - Biological Process	positive regulation of T cell proliferation	experimental (strong)	5	0.929	0.015
Gene Ontology - Biological Process	positive regulation of cellular response to macrophage colony-stimulating factor stimulus	experimental (strong)	2	0.077	0.015
Gene Ontology - Biological Process	positive regulation of interleukin-12 production	experimental (strong)	4	0.568	0.015

Gene Ontology - Biological Process	positive regulation of programmed cell death	experimental (strong)	16	7.434	0.015
Gene Ontology - Biological Process	positive regulation of protein acetylation	experimental (strong)	4	0.568	0.015
Gene Ontology - Biological Process	protein-containing complex assembly	experimental (strong)	20	10.45	0.015
Gene Ontology - Biological Process	regulation of DNA replication	experimental (strong)	5	0.929	0.015
Gene Ontology - Biological Process	regulation of blood vessel size	experimental (strong)	6	1.342	0.015
Gene Ontology - Biological Process	regulation of cytoskeleton organization	experimental (strong)	11	4.13	0.015
Gene Ontology - Biological Process	regulation of pro-B cell differentiation	experimental (strong)	2	0.077	0.015
Gene Ontology - Biological Process	regulation of tube size	experimental (strong)	6	1.368	0.015
Gene Ontology - Biological Process	regulation of vasoconstriction	experimental (strong)	4	0.568	0.015

Gene Ontology - Biological Process	response to UV-A	experimental (strong)	2	0.077	0.015
Gene Ontology - Biological Process	telencephalon cell migration	experimental (strong)	4	0.568	0.015
Gene Ontology - Biological Process	wound healing	experimental (strong)	13	5.42	0.015
Gene Ontology - Biological Process	B cell activation	experimental (any)	8	2.551	0.015
Gene Ontology - Biological Process	Notch signaling pathway	experimental (any)	7	2.02	0.015
Gene Ontology - Biological Process	T cell costimulation	experimental (any)	4	0.651	0.015
Gene Ontology - Biological Process	TRIF-dependent toll-like receptor signaling pathway	experimental (any)	3	0.332	0.015
Gene Ontology - Biological Process	cellular nitrogen compound metabolic process	experimental (any)	87	68.65	0.015
Gene Ontology - Biological Process	cellular response to decreased oxygen levels	experimental (any)	8	2.538	0.015
Gene Ontology - Biological Process	digestive tract development	experimental (any)	6	1.515	0.015

Gene Ontology - Biological Process	heart development	experimental (any)	14	6.271	0.015
Gene Ontology - Biological Process	negative regulation of cytokine biosynthetic process	experimental (any)	3	0.332	0.015
Gene Ontology - Biological Process	neutrophil chemotaxis	experimental (any)	4	0.651	0.015
Gene Ontology - Biological Process	positive regulation of T cell differentiation	experimental (any)	5	1.05	0.015
Gene Ontology - Biological Process	positive regulation of establishment of protein localization	experimental (any)	12	4.956	0.015
Gene Ontology - Biological Process	positive regulation of peptidyl-tyrosine phosphorylation	experimental (any)	7	2.02	0.015
Gene Ontology - Biological Process	receptor signaling pathway via STAT	experimental (any)	6	1.501	0.015
Gene Ontology - Biological Process	regulation of blood vessel endothelial cell migration	experimental (any)	5	1.05	0.015

Gene Ontology - Biological Process	regulation of fibroblast migration	experimental (any)	3	0.332	0.015
Gene Ontology - Biological Process	response to angiotensin	experimental (any)	3	0.332	0.015
Gene Ontology - Biological Process	response to morphine	experimental (any)	3	0.332	0.015
Gene Ontology - Biological Process	regulation of cell size	predicted (union)	75	53.1	0.016
Gene Ontology - Biological Process	RNA biosynthetic process	experimental (strong)	38	25.99	0.016
Gene Ontology - Biological Process	drug metabolic process	experimental (strong)	10	3.562	0.016
Gene Ontology - Biological Process	locomotion	experimental (strong)	27	16.13	0.016
Gene Ontology - Biological Process	mononuclear cell proliferation	experimental (strong)	9	2.968	0.016
Gene Ontology - Biological Process	pericardium development	experimental (strong)	3	0.284	0.016
Gene Ontology - Biological Process	regulation of extrinsic apoptotic signaling pathway via death domain receptors	experimental (strong)	5	0.955	0.016
Gene Ontology - Biological Process	T cell extravasation	experimental (any)	2	0.106	0.016

Gene Ontology - Biological Process	defense response to bacterium	experimental (any)	7	2.033	0.016
Gene Ontology - Biological Process	extracellular matrix organization	experimental (any)	10	3.734	0.016
Gene Ontology - Biological Process	gastrulation	experimental (any)	7	2.059	0.016
Gene Ontology - Biological Process	interleukin-6 production	experimental (any)	6	1.555	0.016
Gene Ontology - Biological Process	male gonad development	experimental (any)	6	1.555	0.016
Gene Ontology - Biological Process	marginal zone B cell differentiation	experimental (any)	2	0.106	0.016
Gene Ontology - Biological Process	membrane protein proteolysis	experimental (any)	4	0.664	0.016
Gene Ontology - Biological Process	negative regulation of calcium ion transport	experimental (any)	4	0.664	0.016
Gene Ontology - Biological Process	neural tube closure	experimental (any)	5	1.076	0.016
Gene Ontology - Biological Process	positive regulation of histone H3-K9 acetylation	experimental (any)	2	0.106	0.016

Gene Ontology - Biological Process	positive regulation of ossification	experimental (any)	5	1.076	0.016
Gene Ontology - Biological Process	positive regulation of transport	experimental (any)	20	10.54	0.016
Gene Ontology - Biological Process	regulation of G protein-coupled receptor signaling pathway	experimental (any)	6	1.555	0.016
Gene Ontology - Biological Process	regulation of cellular component organization	experimental (any)	40	26.45	0.016
Gene Ontology - Biological Process	regulation of extracellular matrix assembly	experimental (any)	2	0.106	0.016
Gene Ontology - Biological Process	regulation of histone acetylation	experimental (any)	4	0.678	0.016
Gene Ontology - Biological Process	regulation of macrophage activation	experimental (any)	4	0.678	0.016
Gene Ontology - Biological Process	regulation of protein localization to plasma membrane	experimental (any)	5	1.076	0.016

Gene Ontology - Biological Process	response to nutrient	experimental (any)	8	2.578	0.016
Gene Ontology - Biological Process	transmembrane receptor protein serine/threonine kinase signaling pathway	experimental (any)	10	3.734	0.016
Gene Ontology - Biological Process	ventricular cardiac muscle tissue development	experimental (any)	4	0.664	0.016
Gene Ontology - Biological Process	cellular response to organonitrogen compound	predicted (intersection)	121	87.5	0.017
Gene Ontology - Biological Process	forebrain cell migration	experimental (strong)	4	0.594	0.017
Gene Ontology - Biological Process	leukocyte degranulation	experimental (strong)	10	3.588	0.017
Gene Ontology - Biological Process	nitric oxide biosynthetic process	experimental (strong)	5	0.981	0.017
Gene Ontology - Biological Process	positive regulation of cytokine production involved in immune response	experimental (strong)	4	0.594	0.017

Gene Ontology - Biological Process	positive regulation of gene expression	experimental (strong)	30	18.84	0.017
Gene Ontology - Biological Process	regulation of G protein-coupled receptor signaling pathway	experimental (strong)	5	0.981	0.017
Gene Ontology - Biological Process	regulation of protein kinase activity	experimental (strong)	17	8.311	0.017
Gene Ontology - Biological Process	adherens junction assembly	experimental (any)	5	1.103	0.017
Gene Ontology - Biological Process	development of primary male sexual characteristics	experimental (any)	6	1.568	0.017
Gene Ontology - Biological Process	granulocyte migration	experimental (any)	5	1.103	0.017
Gene Ontology - Biological Process	mammary gland epithelial cell proliferation	experimental (any)	3	0.345	0.017
Gene Ontology - Biological Process	positive regulation of response to wounding	experimental (any)	4	0.691	0.017

Gene Ontology - Biological Process	regulation of cysteine-type endopeptidase activity	experimental (any)	8	2.618	0.017
Gene Ontology - Biological Process	regulation of immunoglobulin production	experimental (any)	4	0.691	0.017
Gene Ontology - Biological Process	response to X-ray	experimental (any)	3	0.345	0.017
Gene Ontology - Biological Process	response to gonadotropin	experimental (any)	3	0.345	0.017
Gene Ontology - Biological Process	heart valve morphogenesis	experimental (strong)	5	1.007	0.018
Gene Ontology - Biological Process	lymphocyte activation	experimental (strong)	15	6.969	0.018
Gene Ontology - Biological Process	membrane protein proteolysis	experimental (strong)	4	0.619	0.018
Gene Ontology - Biological Process	negative regulation of leukocyte apoptotic process	experimental (strong)	4	0.619	0.018

Gene Ontology - Biological Process	nucleotide-binding domain, leucine rich repeat containing receptor signaling pathway	experimental (strong)	4	0.619	0.018
Gene Ontology - Biological Process	nucleotide-binding oligomerization domain containing signaling pathway	experimental (strong)	4	0.619	0.018
Gene Ontology - Biological Process	positive regulation of RNA biosynthetic process	experimental (strong)	26	15.51	0.018
Gene Ontology - Biological Process	positive regulation of nucleic acid-templated transcription	experimental (strong)	26	15.51	0.018
Gene Ontology - Biological Process	protein secretion	experimental (strong)	12	4.878	0.018
Gene Ontology - Biological Process	regulation of lymphocyte migration	experimental (strong)	4	0.619	0.018

Gene Ontology - Biological Process	stress-activated protein kinase signaling cascade	experimental (strong)	10	3.639	0.018
Gene Ontology - Biological Process	animal organ morphogenesis	experimental (any)	21	11.48	0.018
Gene Ontology - Biological Process	cardiocyte differentiation	experimental (any)	6	1.608	0.018
Gene Ontology - Biological Process	cellular response to dexamethasone stimulus	experimental (any)	3	0.359	0.018
Gene Ontology - Biological Process	epithelial tube morphogenesis	experimental (any)	10	3.84	0.018
Gene Ontology - Biological Process	female gonad development	experimental (any)	5	1.129	0.018
Gene Ontology - Biological Process	morphogenesis of an epithelium	experimental (any)	14	6.431	0.018
Gene Ontology - Biological Process	negative regulation of angiogenesis	experimental (any)	5	1.129	0.018
Gene Ontology - Biological Process	positive regulation of apoptotic signaling pathway	experimental (any)	7	2.099	0.018

Gene Ontology - Biological Process	positive regulation of cytokine biosynthetic process	experimental (any)	4	0.704	0.018
Gene Ontology - Biological Process	positive regulation of erythrocyte differentiation	experimental (any)	3	0.359	0.018
Gene Ontology - Biological Process	positive regulation of histone modification	experimental (any)	5	1.116	0.018
Gene Ontology - Biological Process	positive regulation of interferon-beta production	experimental (any)	3	0.359	0.018
Gene Ontology - Biological Process	positive regulation of protein transport	experimental (any)	11	4.478	0.018
Gene Ontology - Biological Process	regulation of actin filament bundle assembly	experimental (any)	5	1.116	0.018
Gene Ontology - Biological Process	regulation of cell junction assembly	experimental (any)	5	1.116	0.018
Gene Ontology - Biological Process	regulation of interleukin-8 production	experimental (any)	4	0.704	0.018

Gene Ontology - Biological Process	response to wounding	experimental (any)	15	7.109	0.018
Gene Ontology - Biological Process	stress fiber assembly	experimental (any)	5	1.116	0.018
Gene Ontology - Biological Process	superoxide anion generation	experimental (any)	3	0.359	0.018
Gene Ontology - Biological Process	ERK1 and ERK2 cascade	experimental (strong)	10	3.665	0.019
Gene Ontology - Biological Process	T cell proliferation	experimental (strong)	7	1.962	0.019
Gene Ontology - Biological Process	icosanoid biosynthetic process	experimental (strong)	3	0.310	0.019
Gene Ontology - Biological Process	immunoglobulin secretion	experimental (strong)	3	0.310	0.019
Gene Ontology - Biological Process	negative regulation of biomineral tissue development	experimental (strong)	3	0.310	0.019
Gene Ontology - Biological Process	negative regulation of cell migration involved in sprouting angiogenesis	experimental (strong)	3	0.310	0.019

Gene Ontology - Biological Process	positive regulation of nitric-oxide synthase biosynthetic process	experimental (strong)	3	0.310	0.019
Gene Ontology - Biological Process	regulation of protein secretion	experimental (strong)	11	4.285	0.019
Gene Ontology - Biological Process	unsaturated fatty acid biosynthetic process	experimental (strong)	3	0.310	0.019
Gene Ontology - Biological Process	adherens junction organization	experimental (any)	6	1.634	0.019
Gene Ontology - Biological Process	cardiac atrium morphogenesis	experimental (any)	3	0.372	0.019
Gene Ontology - Biological Process	cellular response to insulin-like growth factor stimulus	experimental (any)	2	0.120	0.019
Gene Ontology - Biological Process	coronary artery morphogenesis	experimental (any)	2	0.120	0.019
Gene Ontology - Biological Process	detection of molecule of bacterial origin	experimental (any)	2	0.120	0.019
Gene Ontology - Biological Process	digestive system development	experimental (any)	6	1.634	0.019

Gene Ontology - Biological Process	histone monoubiquitination	experimental (any)	3	0.372	0.019
Gene Ontology - Biological Process	homeostatic process	experimental (any)	31	19.32	0.019
Gene Ontology - Biological Process	interleukin-17 production	experimental (any)	3	0.372	0.019
Gene Ontology - Biological Process	lateral ventricle development	experimental (any)	2	0.120	0.019
Gene Ontology - Biological Process	negative regulation of intrinsic apoptotic signaling pathway	experimental (any)	5	1.143	0.019
Gene Ontology - Biological Process	nervous system development	experimental (any)	38	25.1	0.019
Gene Ontology - Biological Process	positive regulation of cell cycle process	experimental (any)	9	3.282	0.019
Gene Ontology - Biological Process	positive regulation of cyclin-dependent protein serine/threonine kinase activity	experimental (any)	3	0.372	0.019
Gene Ontology - Biological Process	positive regulation of neurogenesis	experimental (any)	12	5.182	0.019

Gene Ontology - Biological Process	positive regulation of production of miRNAs involved in gene silencing by miRNA	experimental (any)	2	0.120	0.019
Gene Ontology - Biological Process	regulation of T cell cytokine production	experimental (any)	3	0.372	0.019
Gene Ontology - Biological Process	regulation of actin filament organization	experimental (any)	8	2.697	0.019
Gene Ontology - Biological Process	regulation of myeloid cell apoptotic process	experimental (any)	3	0.372	0.019
Gene Ontology - Biological Process	regulation of nucleotide-binding oligomerization domain containing signaling pathway	experimental (any)	2	0.120	0.019
Gene Ontology - Biological Process	regulation of superoxide metabolic process	experimental (any)	3	0.372	0.019

Gene Ontology - Biological Process	response to temperature stimulus	experimental (any)	8	2.697	0.019
Gene Ontology - Biological Process	regulation of intracellular protein transport	predicted (union)	109	82.39	0.020
Gene Ontology - Biological Process	DNA replication	experimental (strong)	7	2.013	0.020
Gene Ontology - Biological Process	T cell activation	experimental (strong)	12	5.007	0.020
Gene Ontology - Biological Process	actin polymerization or depolymerization	experimental (strong)	5	1.032	0.020
Gene Ontology - Biological Process	icosanoid metabolic process	experimental (strong)	4	0.645	0.020
Gene Ontology - Biological Process	macrophage activation	experimental (strong)	5	1.032	0.020
Gene Ontology - Biological Process	negative regulation of endothelial cell migration	experimental (strong)	4	0.645	0.020
Gene Ontology - Biological Process	pattern recognition receptor signaling pathway	experimental (strong)	7	2.013	0.020
Gene Ontology - Biological Process	positive regulation of protein secretion	experimental (strong)	8	2.555	0.020

Gene Ontology - Biological Process	regulation of actin filament organization	experimental (strong)	7	1.987	0.020
Gene Ontology - Biological Process	regulation of cytokine production involved in immune response	experimental (strong)	5	1.032	0.020
Gene Ontology - Biological Process	transcription, DNA-templated	experimental (strong)	37	25.55	0.020
Gene Ontology - Biological Process	activation of MAPK activity	experimental (any)	7	2.166	0.020
Gene Ontology - Biological Process	cardiac muscle tissue morphogenesis	experimental (any)	4	0.731	0.020
Gene Ontology - Biological Process	cellular component assembly	experimental (any)	44	30.41	0.020
Gene Ontology - Biological Process	cellular response to organonitrogen compound	experimental (any)	14	6.551	0.020
Gene Ontology - Biological Process	epithelial cell migration	experimental (any)	9	3.335	0.020
Gene Ontology - Biological Process	histone modification	experimental (any)	12	5.222	0.020

Gene Ontology - Biological Process	hormone secretion	experimental (any)	9	3.322	0.020
Gene Ontology - Biological Process	liver regeneration	experimental (any)	3	0.385	0.020
Gene Ontology - Biological Process	negative regulation of cellular response to growth factor stimulus	experimental (any)	6	1.648	0.020
Gene Ontology - Biological Process	peptidyl-serine phosphorylation	experimental (any)	8	2.724	0.020
Gene Ontology - Biological Process	positive regulation of BMP signaling pathway	experimental (any)	3	0.385	0.020
Gene Ontology - Biological Process	positive regulation of epithelial cell proliferation	experimental (any)	7	2.179	0.020
Gene Ontology - Biological Process	positive regulation of interleukin-10 production	experimental (any)	3	0.385	0.020
Gene Ontology - Biological Process	positive regulation of lymphocyte differentiation	experimental (any)	5	1.169	0.020

Gene Ontology - Biological Process	positive regulation of synaptic transmission	experimental (any)	6	1.661	0.020
Gene Ontology - Biological Process	regulation of T cell differentiation	experimental (any)	6	1.661	0.020
Gene Ontology - Biological Process	response to transforming growth factor beta	experimental (any)	8	2.737	0.020
Gene Ontology - Biological Process	signal transduction in response to DNA damage	experimental (any)	6	1.648	0.020
Gene Ontology - Biological Process	toll-like receptor 4 signaling pathway	experimental (any)	3	0.385	0.020
Gene Ontology - Biological Process	negative regulation of cellular metabolic process	predicted (intersection)	430	367.7	0.021
Gene Ontology - Biological Process	regulation of cytoskeleton organization	predicted (union)	199	162.4	0.021
Gene Ontology - Biological Process	regulation of dephosphorylation	predicted (union)	93	68.72	0.021

Gene Ontology - Biological Process	positive regulation of adaptive immune response	experimental (strong)	5	1.058	0.021
Gene Ontology - Biological Process	regulation of RNA biosynthetic process	experimental (strong)	37	25.63	0.021
Gene Ontology - Biological Process	regulation of gliogenesis	experimental (strong)	6	1.523	0.021
Gene Ontology - Biological Process	development of primary female sexual characteristics	experimental (any)	5	1.183	0.021
Gene Ontology - Biological Process	epithelial to mesenchymal transition	experimental (any)	6	1.674	0.021
Gene Ontology - Biological Process	epithelium migration	experimental (any)	9	3.362	0.021
Gene Ontology - Biological Process	negative regulation of RNA biosynthetic process	experimental (any)	23	13.25	0.021
Gene Ontology - Biological Process	negative regulation of nucleic acid-templated transcription	experimental (any)	23	13.23	0.021

Gene Ontology - Biological Process	negative regulation of protein metabolic process	experimental (any)	21	11.68	0.021
Gene Ontology - Biological Process	neutrophil migration	experimental (any)	4	0.757	0.021
Gene Ontology - Biological Process	regulation of toll-like receptor signaling pathway	experimental (any)	4	0.757	0.021
Gene Ontology - Biological Process	ureteric bud development	experimental (any)	5	1.196	0.021
Gene Ontology - Biological Process	cellular response to platelet-derived growth factor stimulus	predicted (intersection)	11	3.4	0.022
Gene Ontology - Biological Process	netrin-activated signaling pathway	predicted (intersection)	8	1.968	0.022
Gene Ontology - Biological Process	cell proliferation involved in heart valve development	experimental (strong)	2	0.103	0.022
Gene Ontology - Biological Process	heart valve development	experimental (strong)	5	1.084	0.022
Gene Ontology - Biological Process	interleukin-6-mediated signaling pathway	experimental (strong)	3	0.336	0.022

Gene Ontology - Biological Process	leukocyte homeostasis	experimental (strong)	5	1.084	0.022
Gene Ontology - Biological Process	muscle cell proliferation	experimental (strong)	9	3.201	0.022
Gene Ontology - Biological Process	negative regulation of extrinsic apoptotic signaling pathway via death domain receptors	experimental (strong)	4	0.671	0.022
Gene Ontology - Biological Process	negative regulation of leukocyte migration	experimental (strong)	3	0.336	0.022
Gene Ontology - Biological Process	peptide secretion	experimental (strong)	12	5.085	0.022
Gene Ontology - Biological Process	positive regulation of histone H3-K9 acetylation	experimental (strong)	2	0.103	0.022
Gene Ontology - Biological Process	positive regulation of interleukin-12 biosynthetic process	experimental (strong)	2	0.103	0.022

Gene Ontology - Biological Process	positive regulation of lipopolysaccharide-mediated signaling pathway	experimental (strong)	2	0.103	0.022
Gene Ontology - Biological Process	positive regulation of reactive oxygen species biosynthetic process	experimental (strong)	4	0.671	0.022
Gene Ontology - Biological Process	positive regulation of secretion	experimental (strong)	10	3.794	0.022
Gene Ontology - Biological Process	regulation of fibroblast migration	experimental (strong)	3	0.336	0.022
Gene Ontology - Biological Process	regulation of immunoglobulin production	experimental (strong)	4	0.671	0.022
Gene Ontology - Biological Process	response to estradiol	experimental (strong)	6	1.549	0.022
Gene Ontology - Biological Process	semi-lunar valve development	experimental (strong)	4	0.671	0.022
Gene Ontology - Biological Process	signaling	experimental (strong)	51	39.78	0.022

Gene Ontology - Biological Process	ventricular cardiac muscle tissue developm ent	experimen tal (strong)	4	0.671	0.022
Gene Ontology - Biological Process	cell projection morphoge nesis	experimen tal (any)	14	6.697	0.022
Gene Ontology - Biological Process	cellular response to alkaloid	experimen tal (any)	3	0.399	0.022
Gene Ontology - Biological Process	cellular response to lipoteichoi c acid	experimen tal (any)	2	0.133	0.022
Gene Ontology - Biological Process	cellular response to macropha ge colony-stimulatin g factor stimulus	experimen tal (any)	2	0.133	0.022
Gene Ontology - Biological Process	cyclooxyg enase pathway	experimen tal (any)	2	0.133	0.022
Gene Ontology - Biological Process	detection of mechanic al stimulus involved in sensory perception of pain	experimen tal (any)	2	0.133	0.022
Gene Ontology - Biological Process	dorsal/ven tral axis specificati on	experimen tal (any)	2	0.133	0.022

Gene Ontology - Biological Process	drug metabolic process	experimental (any)	13	6.006	0.022
Gene Ontology - Biological Process	endocardium development	experimental (any)	2	0.133	0.022
Gene Ontology - Biological Process	forebrain generation of neurons	experimental (any)	4	0.771	0.022
Gene Ontology - Biological Process	hormone transport	experimental (any)	9	3.415	0.022
Gene Ontology - Biological Process	intrinsic apoptotic signaling pathway	experimental (any)	9	3.401	0.022
Gene Ontology - Biological Process	kidney epithelium development	experimental (any)	6	1.687	0.022
Gene Ontology - Biological Process	macrophage derived foam cell differentiation	experimental (any)	3	0.399	0.022
Gene Ontology - Biological Process	mesonephric epithelium development	experimental (any)	5	1.209	0.022
Gene Ontology - Biological Process	mesonephric tubule development	experimental (any)	5	1.209	0.022
Gene Ontology - Biological Process	multicellular organismal homeostasis	experimental (any)	12	5.315	0.022

Gene Ontology - Biological Process	negative regulation of immune system process	experimental (any)	11	4.664	0.022
Gene Ontology - Biological Process	olfactory bulb interneuron differentiation	experimental (any)	2	0.133	0.022
Gene Ontology - Biological Process	pattern specification process	experimental (any)	11	4.677	0.022
Gene Ontology - Biological Process	positive regulation of adherens junction organization	experimental (any)	3	0.399	0.022
Gene Ontology - Biological Process	positive regulation of cell development	experimental (any)	13	5.992	0.022
Gene Ontology - Biological Process	regulation of macrophage apoptotic process	experimental (any)	2	0.133	0.022
Gene Ontology - Biological Process	response to gravity	experimental (any)	2	0.133	0.022
Gene Ontology - Biological Process	response to peptide hormone	experimental (any)	11	4.65	0.022
Gene Ontology - Biological Process	regulation of neuron differentiation	predicted (union)	248	207.3	0.023

Gene Ontology - Biological Process	tube development	predicted (union)	404	351.8	0.023
Gene Ontology - Biological Process	cardiac chamber development	experimental (strong)	7	2.091	0.023
Gene Ontology - Biological Process	cellular component assembly	experimental (strong)	27	16.83	0.023
Gene Ontology - Biological Process	leukocyte proliferation	experimental (strong)	9	3.226	0.023
Gene Ontology - Biological Process	regulation of supramolecular fiber organization	experimental (strong)	8	2.633	0.023
Gene Ontology - Biological Process	cellular response to DNA damage stimulus	experimental (any)	18	9.58	0.023
Gene Ontology - Biological Process	covalent chromatin modification	experimental (any)	12	5.381	0.023
Gene Ontology - Biological Process	interleukin-8 production	experimental (any)	4	0.784	0.023
Gene Ontology - Biological Process	necroptotic process	experimental (any)	3	0.412	0.023
Gene Ontology - Biological Process	negative regulation of ERK1 and ERK2 cascade	experimental (any)	4	0.784	0.023

Gene Ontology - Biological Process	negative regulation of epithelial cell migration	experimental (any)	4	0.784	0.023
Gene Ontology - Biological Process	negative regulation of extrinsic apoptotic signaling pathway	experimental (any)	5	1.236	0.023
Gene Ontology - Biological Process	negative regulation of transcription, DNA-templated	experimental (any)	22	12.65	0.023
Gene Ontology - Biological Process	positive regulation of cell junction assembly	experimental (any)	3	0.412	0.023
Gene Ontology - Biological Process	positive regulation of cyclin-dependent protein kinase activity	experimental (any)	3	0.412	0.023
Gene Ontology - Biological Process	regulation of DNA replication	experimental (any)	5	1.236	0.023
Gene Ontology - Biological Process	regulation of cell cycle	experimental (any)	23	13.38	0.023
Gene Ontology - Biological Process	regulation of extracellular matrix organization	experimental (any)	3	0.412	0.023

Gene Ontology - Biological Process	response to inorganic substance	experimental (any)	13	6.019	0.023
Gene Ontology - Biological Process	central nervous system development	predicted (intersection)	192	150.8	0.024
Gene Ontology - Biological Process	organ growth	predicted (intersection)	44	25.59	0.024
Gene Ontology - Biological Process	response to hormone	predicted (intersection)	186	145.5	0.024
Gene Ontology - Biological Process	tube morphogenesis	predicted (intersection)	169	130.3	0.024
Gene Ontology - Biological Process	brain development	predicted (union)	289	245.2	0.024
Gene Ontology - Biological Process	negative regulation of transcription by RNA polymerase II	predicted (union)	308	262.8	0.024
Gene Ontology - Biological Process	animal organ regeneration	experimental (strong)	5	1.11	0.024
Gene Ontology - Biological Process	positive regulation of innate immune response	experimental (strong)	9	3.252	0.024
Gene Ontology - Biological Process	mesonephros development	experimental (any)	5	1.249	0.024

Gene Ontology - Biological Process	neural precursor cell proliferation	experimental (any)	6	1.754	0.024
Gene Ontology - Biological Process	neural tube formation	experimental (any)	5	1.249	0.024
Gene Ontology - Biological Process	regulation of protein ubiquitination	experimental (any)	7	2.285	0.024
Gene Ontology - Biological Process	tube formation	experimental (any)	6	1.754	0.024
Gene Ontology - Biological Process	central nervous system neuron development	predicted (union)	42	26.94	0.025
Gene Ontology - Biological Process	posttranscriptional regulation of gene expression	predicted (union)	231	192.1	0.025
Gene Ontology - Biological Process	protein dephosphorylation	predicted (union)	135	105.8	0.025
Gene Ontology - Biological Process	regulation of actin filament-based process	predicted (union)	145	114.8	0.025
Gene Ontology - Biological Process	regulation of phosphatase activity	predicted (union)	80	58.18	0.025
Gene Ontology - Biological Process	central nervous system development	experimental (strong)	17	8.827	0.025

Gene Ontology - Biological Process	detection of stimulus	experimental (strong)	5	1.136	0.025
Gene Ontology - Biological Process	innate immune response-activating signal transduction	experimental (strong)	8	2.71	0.025
Gene Ontology - Biological Process	neurotransmitter biosynthetic process	experimental (strong)	5	1.136	0.025
Gene Ontology - Biological Process	positive regulation of MAP kinase activity	experimental (strong)	9	3.278	0.025
Gene Ontology - Biological Process	positive regulation of catalytic activity	experimental (strong)	21	11.98	0.025
Gene Ontology - Biological Process	positive regulation of chemokine production	experimental (strong)	4	0.697	0.025
Gene Ontology - Biological Process	positive regulation of peptide secretion	experimental (strong)	8	2.684	0.025
Gene Ontology - Biological Process	regulation of extracellular matrix organization	experimental (strong)	3	0.361	0.025

Gene Ontology - Biological Process	regulation of granulocyte chemotaxis	experimental (strong)	3	0.361	0.025
Gene Ontology - Biological Process	regulation of peptidyl-serine phosphorylation	experimental (strong)	6	1.6	0.025
Gene Ontology - Biological Process	regulation of protein serine/threonine kinase activity	experimental (strong)	13	5.911	0.025
Gene Ontology - Biological Process	regulation of secretion	experimental (strong)	14	6.608	0.025
Gene Ontology - Biological Process	regulation of transcription, DNA-templated	experimental (strong)	36	25.04	0.025
Gene Ontology - Biological Process	response to ammonium ion	experimental (strong)	5	1.136	0.025
Gene Ontology - Biological Process	response to angiotensin	experimental (strong)	3	0.361	0.025
Gene Ontology - Biological Process	response to stimulus	experimental (strong)	60	50.41	0.025
Gene Ontology - Biological Process	response to tumor necrosis factor	experimental (strong)	8	2.684	0.025
Gene Ontology - Biological Process	ERBB2 signaling pathway	experimental (any)	3	0.425	0.025

Gene Ontology - Biological Process	Notch signaling involved in heart development	experimental (any)	2	0.146	0.025
Gene Ontology - Biological Process	T cell homeostasis	experimental (any)	3	0.425	0.025
Gene Ontology - Biological Process	T-helper 2 cell cytokine production	experimental (any)	2	0.146	0.025
Gene Ontology - Biological Process	bleb assembly	experimental (any)	2	0.146	0.025
Gene Ontology - Biological Process	cellular response to estradiol stimulus	experimental (any)	3	0.425	0.025
Gene Ontology - Biological Process	eyelid development in camera-type eye	experimental (any)	2	0.146	0.025
Gene Ontology - Biological Process	fibroblast migration	experimental (any)	3	0.425	0.025
Gene Ontology - Biological Process	heart valve formation	experimental (any)	2	0.146	0.025
Gene Ontology - Biological Process	macrophage apoptotic process	experimental (any)	2	0.146	0.025
Gene Ontology - Biological Process	positive regulation of DNA replication	experimental (any)	3	0.425	0.025

Gene Ontology - Biological Process	positive regulation of type I interferon-mediated signaling pathway	experimental (any)	2	0.146	0.025
Gene Ontology - Biological Process	pro-B cell differentiation	experimental (any)	2	0.146	0.025
Gene Ontology - Biological Process	regulation of G1/S transition of mitotic cell cycle	experimental (any)	6	1.767	0.025
Gene Ontology - Biological Process	regulation of histone H3-K9 acetylation	experimental (any)	2	0.146	0.025
Gene Ontology - Biological Process	regulation of neuroinflammatory response	experimental (any)	3	0.425	0.025
Gene Ontology - Biological Process	regulation of response to wounding	experimental (any)	6	1.767	0.025
Gene Ontology - Biological Process	regulation of sprouting angiogenesis	experimental (any)	4	0.811	0.025
Gene Ontology - Biological Process	response to ionizing radiation	experimental (any)	6	1.78	0.025
Gene Ontology - Biological Process	small GTPase mediated signal transduction	experimental (any)	12	5.421	0.025

Gene Ontology - Biological Process	sprouting angiogenesis	experimental (any)	5	1.262	0.025
Gene Ontology - Biological Process	response to transforming growth factor beta	predicted (intersection)	58	36.68	0.026
Gene Ontology - Biological Process	cell communication	experimental (strong)	51	40.19	0.026
Gene Ontology - Biological Process	lymphocyte homeostasis	experimental (strong)	4	0.723	0.026
Gene Ontology - Biological Process	positive regulation of small GTPase mediated signal transduction	experimental (strong)	4	0.723	0.026
Gene Ontology - Biological Process	regulation of cell migration involved in sprouting angiogenesis	experimental (strong)	4	0.723	0.026
Gene Ontology - Biological Process	response to steroid hormone	experimental (strong)	11	4.594	0.026
Gene Ontology - Biological Process	tissue remodeling	experimental (strong)	7	2.168	0.026
Gene Ontology - Biological Process	vasoconstriction	experimental (strong)	4	0.723	0.026

Gene Ontology - Biological Process	adaptive immune response	experimental (any)	10	4.159	0.026
Gene Ontology - Biological Process	cardiac muscle tissue development	experimental (any)	7	2.352	0.026
Gene Ontology - Biological Process	cell part morphogenesis	experimental (any)	14	6.883	0.026
Gene Ontology - Biological Process	cellular process	experimental (any)	170	156.4	0.026
Gene Ontology - Biological Process	negative regulation of transcription by RNA polymerase II	experimental (any)	17	9.048	0.026
Gene Ontology - Biological Process	prostaglandin metabolic process	experimental (any)	3	0.438	0.026
Gene Ontology - Biological Process	protein monoubiquitination	experimental (any)	4	0.824	0.026
Gene Ontology - Biological Process	regulation of cell cycle arrest	experimental (any)	5	1.289	0.026
Gene Ontology - Biological Process	tissue homeostasis	experimental (any)	7	2.352	0.026
Gene Ontology - Biological Process	vasoconstriction	experimental (any)	4	0.824	0.026

Gene Ontology - Biological Process	endothelium development	experimental (strong)	6	1.652	0.027
Gene Ontology - Biological Process	positive regulation of cell development	experimental (strong)	12	5.291	0.027
Gene Ontology - Biological Process	posttranscriptional regulation of gene expression	experimental (strong)	10	3.975	0.027
Gene Ontology - Biological Process	regulation of innate immune response	experimental (strong)	10	3.975	0.027
Gene Ontology - Biological Process	developmental growth	experimental (any)	14	6.909	0.027
Gene Ontology - Biological Process	phosphatidylinositol-mediated signaling	experimental (any)	6	1.807	0.027
Gene Ontology - Biological Process	positive regulation of B cell activation	experimental (any)	4	0.837	0.027
Gene Ontology - Biological Process	protein autoubiquitination	experimental (any)	4	0.837	0.027
Gene Ontology - Biological Process	regulation of chemokine production	experimental (any)	4	0.837	0.027
Gene Ontology - Biological Process	regulation of protein transport	experimental (strong)	13	6.014	0.028

Gene Ontology - Biological Process	response to radiation	experimental (strong)	10	4.001	0.028
Gene Ontology - Biological Process	bone mineralization	experimental (any)	5	1.315	0.028
Gene Ontology - Biological Process	cardiac ventricle morphogenesis	experimental (any)	4	0.850	0.028
Gene Ontology - Biological Process	cell development	experimental (any)	33	21.84	0.028
Gene Ontology - Biological Process	endocardial cushion morphogenesis	experimental (any)	3	0.452	0.028
Gene Ontology - Biological Process	hydrogen peroxide metabolic process	experimental (any)	3	0.452	0.028
Gene Ontology - Biological Process	negative regulation of cell cycle	experimental (any)	14	6.962	0.028
Gene Ontology - Biological Process	negative regulation of leukocyte migration	experimental (any)	3	0.452	0.028
Gene Ontology - Biological Process	regulation of B cell activation	experimental (any)	5	1.315	0.028
Gene Ontology - Biological Process	regulation of bone mineralization	experimental (any)	4	0.850	0.028
Gene Ontology - Biological Process	reproductive structure development	experimental (any)	11	4.89	0.028

Gene Ontology - Biological Process	response to xenobiotic stimulus	experimental (any)	8	2.963	0.028
Gene Ontology - Biological Process	cellular response to growth factor stimulus	predicted (intersection)	136	102.2	0.029
Gene Ontology - Biological Process	regulation of cellular protein localization	predicted (intersection)	116	84.99	0.029
Gene Ontology - Biological Process	tricuspid valve morphogenesis	predicted (intersection)	5	0.895	0.029
Gene Ontology - Biological Process	cardiac muscle cell differentiation	predicted (union)	53	35.92	0.029
Gene Ontology - Biological Process	cell projection organization	predicted (union)	546	487.3	0.029
Gene Ontology - Biological Process	forebrain development	predicted (union)	163	131.2	0.029
Gene Ontology - Biological Process	head development	predicted (union)	304	260	0.029
Gene Ontology - Biological Process	T cell extravasation	experimental (strong)	2	0.129	0.029
Gene Ontology - Biological Process	adaptive immune response	experimental (strong)	8	2.813	0.029

Gene Ontology - Biological Process	atrioventricular valve formation	experimental (strong)	2	0.129	0.029
Gene Ontology - Biological Process	bone resorption	experimental (strong)	4	0.749	0.029
Gene Ontology - Biological Process	cardiac left ventricle morphogenesis	experimental (strong)	2	0.129	0.029
Gene Ontology - Biological Process	cellular response to alkaloid	experimental (strong)	3	0.387	0.029
Gene Ontology - Biological Process	cellular response to dexamethasone stimulus	experimental (strong)	3	0.387	0.029
Gene Ontology - Biological Process	cellular response to hormone stimulus	experimental (strong)	14	6.814	0.029
Gene Ontology - Biological Process	cellular response to macrophage colony-stimulating factor stimulus	experimental (strong)	2	0.129	0.029
Gene Ontology - Biological Process	common-partner SMAD protein phosphorylation	experimental (strong)	2	0.129	0.029

Gene Ontology - Biological Process	cytoplasmic pattern recognition receptor signaling pathway	experimental (strong)	4	0.749	0.029
Gene Ontology - Biological Process	detection of mechanical stimulus involved in sensory perception of pain	experimental (strong)	2	0.129	0.029
Gene Ontology - Biological Process	detection of molecule of bacterial origin	experimental (strong)	2	0.129	0.029
Gene Ontology - Biological Process	endocardial cell differentiation	experimental (strong)	2	0.129	0.029
Gene Ontology - Biological Process	lymph node development	experimental (strong)	2	0.129	0.029
Gene Ontology - Biological Process	marginal zone B cell differentiation	experimental (strong)	2	0.129	0.029
Gene Ontology - Biological Process	membrane to membrane docking	experimental (strong)	2	0.129	0.029
Gene Ontology - Biological Process	necroptotic signaling pathway	experimental (strong)	2	0.129	0.029

Gene Ontology - Biological Process	negative regulation of cell cycle	experimental (strong)	12	5.42	0.029
Gene Ontology - Biological Process	negative regulation of cell migration	experimental (strong)	8	2.813	0.029
Gene Ontology - Biological Process	negative regulation of cell population proliferation	experimental (strong)	15	7.537	0.029
Gene Ontology - Biological Process	pericardium morphogenesis	experimental (strong)	2	0.129	0.029
Gene Ontology - Biological Process	positive regulation of neurogenesis	experimental (strong)	11	4.672	0.029
Gene Ontology - Biological Process	positive regulation of peptidyl-tyrosine phosphorylation	experimental (strong)	7	2.22	0.029
Gene Ontology - Biological Process	protein modification by small protein removal	experimental (strong)	7	2.22	0.029
Gene Ontology - Biological Process	regulation of angiogenesis	experimental (strong)	10	4.078	0.029
Gene Ontology - Biological Process	regulation of cell cycle arrest	experimental (strong)	5	1.187	0.029

Gene Ontology - Biological Process	regulation of epithelial to mesenchymal transition	experimental (strong)	5	1.187	0.029
Gene Ontology - Biological Process	regulation of extracellular matrix assembly	experimental (strong)	2	0.129	0.029
Gene Ontology - Biological Process	regulation of histone H3-K9 acetylation	experimental (strong)	2	0.129	0.029
Gene Ontology - Biological Process	regulation of kinase activity	experimental (strong)	17	9.06	0.029
Gene Ontology - Biological Process	toll-like receptor 2 signaling pathway	experimental (strong)	2	0.129	0.029
Gene Ontology - Biological Process	JNK cascade	experimental (any)	7	2.405	0.029
Gene Ontology - Biological Process	actomyosin structure organization	experimental (any)	6	1.86	0.029
Gene Ontology - Biological Process	astrocyte development	experimental (any)	3	0.465	0.029
Gene Ontology - Biological Process	cardiac left ventricle morphogenesis	experimental (any)	2	0.159	0.029

Gene Ontology - Biological Process	detection of temperature stimulus involved in sensory perception of pain	experimental (any)	2	0.159	0.029
Gene Ontology - Biological Process	formation of primary germ layer	experimental (any)	5	1.342	0.029
Gene Ontology - Biological Process	inositol lipid-mediated signaling	experimental (any)	6	1.847	0.029
Gene Ontology - Biological Process	male sex differentiation	experimental (any)	6	1.847	0.029
Gene Ontology - Biological Process	microglial cell activation	experimental (any)	3	0.465	0.029
Gene Ontology - Biological Process	negative regulation of bone remodeling	experimental (any)	2	0.159	0.029
Gene Ontology - Biological Process	negative regulation of cell differentiation	experimental (any)	15	7.706	0.029
Gene Ontology - Biological Process	negative regulation of gene expression	experimental (any)	31	20.22	0.029

Gene Ontology - Biological Process	negative regulation of intracellular steroid hormone receptor signaling pathway	experimental (any)	3	0.465	0.029
Gene Ontology - Biological Process	negative regulation of leukocyte chemotaxis	experimental (any)	2	0.159	0.029
Gene Ontology - Biological Process	neurogenesis	experimental (any)	27	17.01	0.029
Gene Ontology - Biological Process	nucleotide-binding oligomerization domain containing 2 signaling pathway	experimental (any)	2	0.159	0.029
Gene Ontology - Biological Process	positive regulation of SMAD protein signal transduction	experimental (any)	2	0.159	0.029
Gene Ontology - Biological Process	positive regulation of fibroblast migration	experimental (any)	2	0.159	0.029
Gene Ontology - Biological Process	positive regulation of hormone secretion	experimental (any)	5	1.342	0.029

Gene Ontology - Biological Process	positive regulation of interleukin-17 production	experimental (any)	2	0.159	0.029
Gene Ontology - Biological Process	positive regulation of receptor signaling pathway via JAK-STAT	experimental (any)	4	0.864	0.029
Gene Ontology - Biological Process	positive regulation of tumor necrosis factor production	experimental (any)	4	0.864	0.029
Gene Ontology - Biological Process	regulation of bone resorption	experimental (any)	3	0.465	0.029
Gene Ontology - Biological Process	regulation of cell-substrate adhesion	experimental (any)	7	2.405	0.029
Gene Ontology - Biological Process	regulation of granulocyte chemotaxis	experimental (any)	3	0.465	0.029
Gene Ontology - Biological Process	regulation of heart morphogenesis	experimental (any)	3	0.465	0.029
Gene Ontology - Biological Process	regulation of neurotransmitter levels	experimental (any)	9	3.627	0.029

Gene Ontology - Biological Process	regulation of phosphatidylinositol 3-kinase signaling	experimental (any)	5	1.329	0.029
Gene Ontology - Biological Process	reproductive system development	experimental (any)	11	4.929	0.029
Gene Ontology - Biological Process	response to estrogen	experimental (any)	4	0.864	0.029
Gene Ontology - Biological Process	response to progesterone	experimental (any)	3	0.465	0.029
Gene Ontology - Biological Process	sex differentiation	experimental (any)	8	3.003	0.029
Gene Ontology - Biological Process	response to growth factor	predicted (intersection)	141	106.8	0.030
Gene Ontology - Biological Process	activation of immune response	experimental (strong)	11	4.749	0.030
Gene Ontology - Biological Process	activation of innate immune response	experimental (strong)	8	2.839	0.030
Gene Ontology - Biological Process	cardiac muscle tissue morphogenesis	experimental (strong)	4	0.774	0.030
Gene Ontology - Biological Process	negative regulation of blood vessel diameter	experimental (strong)	4	0.774	0.030

Gene Ontology - Biological Process	regulation of MAP kinase activity	experimental (strong)	10	4.104	0.030
Gene Ontology - Biological Process	regulation of neurogenesis	experimental (strong)	15	7.588	0.030
Gene Ontology - Biological Process	regulation of nucleic acid-templated transcription	experimental (strong)	36	25.53	0.030
Gene Ontology - Biological Process	Rho protein signal transduction	experimental (any)	6	1.873	0.030
Gene Ontology - Biological Process	positive regulation of mitotic cell cycle phase transition	experimental (any)	4	0.877	0.030
Gene Ontology - Biological Process	positive regulation of protein polymerization	experimental (any)	5	1.355	0.030
Gene Ontology - Biological Process	response to peptide	experimental (any)	12	5.634	0.030
Gene Ontology - Biological Process	cell surface receptor signaling pathway	predicted (intersection)	504	440.5	0.031
Gene Ontology - Biological Process	Golgi vesicle transport	predicted (union)	136	107.4	0.031

Gene Ontology - Biological Process	actin cytoskeleton organization	experimental (any)	13	6.391	0.031
Gene Ontology - Biological Process	extracellular structure organization	experimental (any)	10	4.318	0.031
Gene Ontology - Biological Process	muscle organ development	experimental (any)	10	4.318	0.031
Gene Ontology - Biological Process	negative regulation of blood vessel diameter	experimental (any)	4	0.890	0.031
Gene Ontology - Biological Process	positive regulation of bone mineralization	experimental (any)	3	0.478	0.031
Gene Ontology - Biological Process	positive regulation of receptor signaling pathway via STAT	experimental (any)	4	0.890	0.031
Gene Ontology - Biological Process	programmed necrotic cell death	experimental (any)	3	0.478	0.031
Gene Ontology - Biological Process	response to dsRNA	experimental (any)	3	0.478	0.031
Gene Ontology - Biological Process	response to testosterone	experimental (any)	3	0.478	0.031

Gene Ontology - Biological Process	somatic stem cell population maintenance	experimental (any)	4	0.890	0.031
Gene Ontology - Biological Process	MyD88-independent toll-like receptor signaling pathway	experimental (strong)	3	0.413	0.032
Gene Ontology - Biological Process	cardiac septum morphogenesis	experimental (strong)	5	1.239	0.032
Gene Ontology - Biological Process	cellular protein-containing complex assembly	experimental (strong)	11	4.801	0.032
Gene Ontology - Biological Process	fibroblast migration	experimental (strong)	3	0.413	0.032
Gene Ontology - Biological Process	hydrogen peroxide metabolic process	experimental (strong)	3	0.413	0.032
Gene Ontology - Biological Process	negative regulation of cell motility	experimental (strong)	8	2.891	0.032
Gene Ontology - Biological Process	negative regulation of ion transport	experimental (strong)	5	1.239	0.032
Gene Ontology - Biological Process	regulation of gene expression	experimental (strong)	41	30.43	0.032
Gene Ontology - Biological Process	response to gonadotropin	experimental (strong)	3	0.413	0.032

Gene Ontology - Biological Process	response to morphine	experimental (strong)	3	0.413	0.032
Gene Ontology - Biological Process	actin filament organization	experimental (any)	9	3.72	0.032
Gene Ontology - Biological Process	endodermal cell fate commitment	experimental (any)	2	0.173	0.032
Gene Ontology - Biological Process	histone H3-K9 acetylation	experimental (any)	2	0.173	0.032
Gene Ontology - Biological Process	hyaluronan biosynthetic process	experimental (any)	2	0.173	0.032
Gene Ontology - Biological Process	positive regulation of cellular extravasation	experimental (any)	2	0.173	0.032
Gene Ontology - Biological Process	positive regulation of macrophage differentiation	experimental (any)	2	0.173	0.032
Gene Ontology - Biological Process	regulation of extracellular matrix disassembly	experimental (any)	2	0.173	0.032

Gene Ontology - Biological Process	cellular response to transforming growth factor beta stimulus	predicted (intersection)	56	35.61	0.033
Gene Ontology - Biological Process	positive regulation of cell cycle process	experimental (strong)	8	2.917	0.033
Gene Ontology - Biological Process	positive regulation of fibroblast proliferation	experimental (strong)	4	0.800	0.033
Gene Ontology - Biological Process	positive regulation of interleukin-6 production	experimental (strong)	4	0.800	0.033
Gene Ontology - Biological Process	regulation of cell activation	experimental (strong)	12	5.549	0.033
Gene Ontology - Biological Process	regulation of histone acetylation	experimental (strong)	4	0.800	0.033
Gene Ontology - Biological Process	translation	experimental (strong)	8	2.917	0.033
Gene Ontology - Biological Process	T cell cytokine production	experimental (any)	3	0.492	0.033
Gene Ontology - Biological Process	icosanoid biosynthetic process	experimental (any)	3	0.492	0.033

Gene Ontology - Biological Process	negative regulation of cell growth	experimental (any)	6	1.927	0.033
Gene Ontology - Biological Process	neuron projection morphogenesis	experimental (any)	13	6.457	0.033
Gene Ontology - Biological Process	regulation of animal organ morphogenesis	experimental (any)	8	3.109	0.033
Gene Ontology - Biological Process	regulation of cell-matrix adhesion	experimental (any)	5	1.395	0.033
Gene Ontology - Biological Process	regulation of oligodendrocyte differentiation	experimental (any)	3	0.492	0.033
Gene Ontology - Biological Process	response to ammonium ion	experimental (any)	5	1.395	0.033
Gene Ontology - Biological Process	response to dexamethasone	experimental (any)	3	0.492	0.033
Gene Ontology - Biological Process	cell-cell signaling by wnt	predicted (intersection)	109	79.62	0.034
Gene Ontology - Biological Process	phosphate-containing compound metabolic process	predicted (intersection)	553	487.8	0.034

Gene Ontology - Biological Process	cellular response to drug	experimental (strong)	10	4.207	0.034
Gene Ontology - Biological Process	entry into host cell	experimental (strong)	5	1.265	0.034
Gene Ontology - Biological Process	immune response-regulating signaling pathway	experimental (strong)	11	4.878	0.034
Gene Ontology - Biological Process	tissue homeostasis	experimental (strong)	6	1.781	0.034
Gene Ontology - Biological Process	astrocyte differentiation	experimental (any)	4	0.917	0.034
Gene Ontology - Biological Process	positive regulation of axonogenesis	experimental (any)	4	0.917	0.034
Gene Ontology - Biological Process	positive regulation of cellular protein localization	experimental (any)	9	3.76	0.034
Gene Ontology - Biological Process	positive regulation of organelle organization	experimental (any)	14	7.188	0.034
Gene Ontology - Biological Process	Notch signaling pathway	experimental (strong)	6	1.807	0.035
Gene Ontology - Biological Process	T cell mediated immunity	experimental (strong)	4	0.826	0.035

Gene Ontology - Biological Process	T-helper 1 type immune response	experimental (strong)	3	0.439	0.035
Gene Ontology - Biological Process	activation of MAPK activity	experimental (strong)	7	2.349	0.035
Gene Ontology - Biological Process	blood circulation	experimental (strong)	10	4.259	0.035
Gene Ontology - Biological Process	cardiac atrium morphogenesis	experimental (strong)	3	0.439	0.035
Gene Ontology - Biological Process	extrinsic apoptotic signaling pathway via death domain receptors	experimental (strong)	5	1.291	0.035
Gene Ontology - Biological Process	necroptotic process	experimental (strong)	3	0.439	0.035
Gene Ontology - Biological Process	negative regulation of chemotaxis	experimental (strong)	3	0.439	0.035
Gene Ontology - Biological Process	negative regulation of epithelial cell migration	experimental (strong)	4	0.826	0.035
Gene Ontology - Biological Process	positive regulation of BMP signaling pathway	experimental (strong)	3	0.439	0.035

Gene Ontology - Biological Process	positive regulation of cytokine biosynthetic process	experimental (strong)	4	0.826	0.035
Gene Ontology - Biological Process	positive regulation of interleukin-10 production	experimental (strong)	3	0.439	0.035
Gene Ontology - Biological Process	positive regulation of interleukin-8 production	experimental (strong)	3	0.439	0.035
Gene Ontology - Biological Process	positive regulation of mitotic cell cycle phase transition	experimental (strong)	4	0.826	0.035
Gene Ontology - Biological Process	positive regulation of tumor necrosis factor production	experimental (strong)	4	0.826	0.035
Gene Ontology - Biological Process	regulation of cytokine biosynthetic process	experimental (strong)	5	1.291	0.035
Gene Ontology - Biological Process	regulation of nitric oxide biosynthetic process	experimental (strong)	4	0.826	0.035

Gene Ontology - Biological Process	regulation of smooth muscle cell proliferation	experimental (strong)	7	2.375	0.035
Gene Ontology - Biological Process	smooth muscle cell proliferation	experimental (strong)	7	2.375	0.035
Gene Ontology - Biological Process	stress-activated MAPK cascade	experimental (strong)	9	3.588	0.035
Gene Ontology - Biological Process	actin polymerization or depolymerization	experimental (any)	6	1.966	0.035
Gene Ontology - Biological Process	activation of cysteine-type endopeptidase activity involved in apoptotic process	experimental (any)	4	0.930	0.035
Gene Ontology - Biological Process	biological_process	experimental (any)	183	172.5	0.035
Gene Ontology - Biological Process	cell-matrix adhesion	experimental (any)	7	2.538	0.035
Gene Ontology - Biological Process	cell-substrate adhesion	experimental (any)	9	3.8	0.035
Gene Ontology - Biological Process	chemokine production	experimental (any)	4	0.930	0.035

Gene Ontology - Biological Process	developmental growth involved in morphogenesis	experimental (any)	7	2.551	0.035
Gene Ontology - Biological Process	enzyme linked receptor protein signaling pathway	experimental (any)	19	10.98	0.035
Gene Ontology - Biological Process	establishment of protein localization	experimental (any)	32	21.52	0.035
Gene Ontology - Biological Process	macromolecule glycosylation	experimental (any)	7	2.551	0.035
Gene Ontology - Biological Process	macrophage differentiation	experimental (any)	3	0.505	0.035
Gene Ontology - Biological Process	negative regulation of T cell apoptotic process	experimental (any)	2	0.186	0.035
Gene Ontology - Biological Process	negative regulation of gene silencing by miRNA	experimental (any)	2	0.186	0.035
Gene Ontology - Biological Process	negative regulation of interleukin-17 production	experimental (any)	2	0.186	0.035

Gene Ontology - Biological Process	negative regulation of intracellular signal transduction	experimental (any)	12	5.806	0.035
Gene Ontology - Biological Process	negative regulation of viral entry into host cell	experimental (any)	2	0.186	0.035
Gene Ontology - Biological Process	nitrogen compound metabolic process	experimental (any)	120	104.1	0.035
Gene Ontology - Biological Process	positive regulation of regulatory T cell differentiation	experimental (any)	2	0.186	0.035
Gene Ontology - Biological Process	positive regulation of type 2 immune response	experimental (any)	2	0.186	0.035
Gene Ontology - Biological Process	protein glycosylation	experimental (any)	7	2.551	0.035
Gene Ontology - Biological Process	protein ubiquitination	experimental (any)	17	9.447	0.035
Gene Ontology - Biological Process	regulation of cell cycle G1/S phase transition	experimental (any)	6	1.953	0.035

Gene Ontology - Biological Process	regulation of stress-activated MAPK cascade	experimental (any)	7	2.538	0.035
Gene Ontology - Biological Process	regulation of transcription by RNA polymerase II	experimental (any)	41	29.2	0.035
Gene Ontology - Biological Process	regulation of wound healing	experimental (any)	5	1.422	0.035
Gene Ontology - Biological Process	response to antibiotic	experimental (any)	9	3.8	0.035
Gene Ontology - Biological Process	response to fungus	experimental (any)	3	0.505	0.035
Gene Ontology - Biological Process	toll-like receptor 2 signaling pathway	experimental (any)	2	0.186	0.035
Gene Ontology - Biological Process	ventricular septum development	experimental (any)	4	0.930	0.035
Gene Ontology - Biological Process	developmental cell growth	predicted (union)	92	69.11	0.036
Gene Ontology - Biological Process	regulation of axonogenesis	predicted (union)	70	50.37	0.036
Gene Ontology - Biological Process	Ras protein signal transduction	experimental (strong)	8	2.994	0.036

Gene Ontology - Biological Process	T-helper 1 type immune response	experimental (any)	3	0.518	0.036
Gene Ontology - Biological Process	amide transport	experimental (any)	31	20.75	0.036
Gene Ontology - Biological Process	cell cycle	experimental (any)	30	19.94	0.036
Gene Ontology - Biological Process	cell morphogenesis	experimental (any)	18	10.26	0.036
Gene Ontology - Biological Process	positive regulation of cell cycle G1/S phase transition	experimental (any)	3	0.518	0.036
Gene Ontology - Biological Process	positive regulation of cysteine-type endopeptidase activity involved in apoptotic process	experimental (any)	5	1.448	0.036
Gene Ontology - Biological Process	regulation of Ras protein signal transduction	experimental (any)	7	2.564	0.036
Gene Ontology - Biological Process	regulation of protein complex assembly	experimental (any)	10	4.491	0.036

Gene Ontology - Biological Process	regulation of stress-activated protein kinase signaling cascade	experimental (any)	7	2.564	0.036
Gene Ontology - Biological Process	response to extracellular stimulus	experimental (any)	12	5.846	0.036
Gene Ontology - Biological Process	response to osmotic stress	experimental (any)	4	0.943	0.036
Gene Ontology - Biological Process	transcription by RNA polymerase II	experimental (any)	43	31.03	0.036
Gene Ontology - Biological Process	regulation of actin cytoskeleton organization	predicted (union)	128	100.7	0.037
Gene Ontology - Biological Process	cellular response to insulin-like growth factor stimulus	experimental (strong)	2	0.155	0.037
Gene Ontology - Biological Process	cellular response to lipoteichoic acid	experimental (strong)	2	0.155	0.037

Gene Ontology - Biological Process	detection of mechanical stimulus involved in sensory perception	experimental (strong)	2	0.155	0.037
Gene Ontology - Biological Process	detection of temperature stimulus involved in sensory perception of pain	experimental (strong)	2	0.155	0.037
Gene Ontology - Biological Process	histone H3-K9 acetylation	experimental (strong)	2	0.155	0.037
Gene Ontology - Biological Process	homeostatic process	experimental (strong)	22	13.47	0.037
Gene Ontology - Biological Process	negative regulation of leukocyte chemotaxis	experimental (strong)	2	0.155	0.037
Gene Ontology - Biological Process	positive regulation of cellular component biogenesis	experimental (strong)	10	4.31	0.037
Gene Ontology - Biological Process	positive regulation of histone H3-K4 methylation	experimental (strong)	2	0.155	0.037

Gene Ontology - Biological Process	positive regulation of protein polymerization	experimental (strong)	4	0.852	0.037
Gene Ontology - Biological Process	pro-B cell differentiation	experimental (strong)	2	0.155	0.037
Gene Ontology - Biological Process	regulation of cytokine-mediated signaling pathway	experimental (strong)	6	1.833	0.037
Gene Ontology - Biological Process	regulation of ion transport	experimental (strong)	10	4.31	0.037
Gene Ontology - Biological Process	response to peptide hormone	experimental (strong)	10	4.31	0.037

Gene Ontology - Biological Process	anatomical structure formation involved in morphogenesis	experimental (strong)	20	11.85	0.038
Gene Ontology - Biological Process	cerebral cortex cell migration	experimental (any)	3	0.531	0.038
Gene Ontology - Biological Process	generation of neurons	experimental (any)	25	15.89	0.038
Gene Ontology - Biological Process	myelination	experimental (any)	5	1.462	0.038
Gene Ontology - Biological Process	negative regulation of BMP signaling pathway	experimental (any)	3	0.531	0.038

Gene Ontology - Biological Process	negative regulation of G protein-coupled receptor signaling pathway	experimental (any)	3	0.531	0.038
Gene Ontology - Biological Process	response to carbohydrate	experimental (any)	7	2.604	0.038
Gene Ontology - Biological Process	response to metal ion	experimental (any)	9	3.867	0.038
Gene Ontology - Biological Process	response to reactive oxygen species	experimental (any)	7	2.604	0.038
Gene Ontology - Biological Process	unsaturated fatty acid biosynthetic process	experimental (any)	3	0.531	0.038
Gene Ontology - Biological Process	cellular protein localization	predicted (union)	612	551.7	0.039
Gene Ontology - Biological Process	cardiac ventricle morphogenesis	experimental (strong)	4	0.878	0.039
Gene Ontology - Biological Process	cell development	experimental (strong)	26	17.01	0.039
Gene Ontology - Biological Process	cellular response to tumor necrosis factor	experimental (strong)	7	2.452	0.039
Gene Ontology - Biological Process	detection of abiotic stimulus	experimental (strong)	3	0.465	0.039

Gene Ontology - Biological Process	endothelial cell differentiation	experimental (strong)	5	1.342	0.039
Gene Ontology - Biological Process	fatty acid biosynthetic process	experimental (strong)	4	0.878	0.039
Gene Ontology - Biological Process	gene silencing by RNA	experimental (strong)	5	1.342	0.039
Gene Ontology - Biological Process	glial cell development	experimental (strong)	5	1.342	0.039
Gene Ontology - Biological Process	hemopoiesis	experimental (strong)	16	8.698	0.039
Gene Ontology - Biological Process	interleukin-17 production	experimental (strong)	3	0.465	0.039
Gene Ontology - Biological Process	muscle tissue morphogenesis	experimental (strong)	4	0.878	0.039
Gene Ontology - Biological Process	myeloid cell apoptotic process	experimental (strong)	3	0.465	0.039
Gene Ontology - Biological Process	negative regulation of cellular component movement	experimental (strong)	8	3.072	0.039
Gene Ontology - Biological Process	negative regulation of sprouting angiogenesis	experimental (strong)	3	0.465	0.039

Gene Ontology - Biological Process	positive regulation of DNA replication	experimental (strong)	3	0.465	0.039
Gene Ontology - Biological Process	positive regulation of T cell mediated immunity	experimental (strong)	3	0.465	0.039
Gene Ontology - Biological Process	positive regulation of cell cycle phase transition	experimental (strong)	4	0.878	0.039
Gene Ontology - Biological Process	positive regulation of cyclin-dependent protein serine/threonine kinase activity	experimental (strong)	3	0.465	0.039
Gene Ontology - Biological Process	positive regulation of protein transport	experimental (strong)	9	3.691	0.039
Gene Ontology - Biological Process	regulation of establishment of protein localization	experimental (strong)	13	6.453	0.039
Gene Ontology - Biological Process	regulation of oligodendrocyte differentiation	experimental (strong)	3	0.465	0.039

Gene Ontology - Biological Process	alpha-beta T cell lineage commitment	experimental (any)	2	0.199	0.039
Gene Ontology - Biological Process	axonogenesis	experimental (any)	10	4.544	0.039
Gene Ontology - Biological Process	calcium ion transport	experimental (any)	10	4.544	0.039
Gene Ontology - Biological Process	cell junction assembly	experimental (any)	7	2.631	0.039
Gene Ontology - Biological Process	detection of temperature stimulus	experimental (any)	2	0.199	0.039
Gene Ontology - Biological Process	lymph node development	experimental (any)	2	0.199	0.039
Gene Ontology - Biological Process	macrophage cytokine production	experimental (any)	2	0.199	0.039
Gene Ontology - Biological Process	metanephric mesenchyme development	experimental (any)	2	0.199	0.039
Gene Ontology - Biological Process	peptide hormone secretion	experimental (any)	7	2.631	0.039
Gene Ontology - Biological Process	positive regulation of monocyte chemotaxis	experimental (any)	2	0.199	0.039

Gene Ontology - Biological Process	regulation of hydrogen peroxide metabolic process	experimental (any)	2	0.199	0.039
Gene Ontology - Biological Process	response to muscle stretch	experimental (any)	2	0.199	0.039
Gene Ontology - Biological Process	Wnt signaling pathway	predicted (intersection)	108	79.27	0.040
Gene Ontology - Biological Process	neurogenesis	predicted (intersection)	284	236.5	0.040
Gene Ontology - Biological Process	positive regulation of metabolic process	predicted (intersection)	591	525.2	0.040
Gene Ontology - Biological Process	tube development	predicted (intersection)	201	161.2	0.040
Gene Ontology - Biological Process	intracellular protein transport	predicted (union)	352	306.1	0.040
Gene Ontology - Biological Process	nucleocytoplasmic transport	predicted (union)	133	105.4	0.040
Gene Ontology - Biological Process	actin cytoskeleton organization	experimental (strong)	10	4.388	0.040
Gene Ontology - Biological Process	metabolic process	experimental (strong)	63	55.21	0.040

Gene Ontology - Biological Process	negative regulation of angiogenesis	experimental (strong)	5	1.368	0.040
Gene Ontology - Biological Process	regulation of I-kappaB kinase/NF-kappaB signaling	experimental (strong)	7	2.478	0.040
Gene Ontology - Biological Process	regulation of type I interferon production	experimental (strong)	5	1.368	0.040
Gene Ontology - Biological Process	response to carbohydrate	experimental (strong)	7	2.478	0.040
Gene Ontology - Biological Process	response to toxic substance	experimental (strong)	11	5.085	0.040
Gene Ontology - Biological Process	type I interferon production	experimental (strong)	5	1.368	0.040
Gene Ontology - Biological Process	embryonic epithelial tube formation	experimental (any)	5	1.488	0.040
Gene Ontology - Biological Process	import into cell	experimental (any)	13	6.697	0.040
Gene Ontology - Biological Process	positive regulation of cytokine-mediated signaling pathway	experimental (any)	3	0.545	0.040
Gene Ontology - Biological Process	protein transport	experimental (any)	30	20.14	0.040

Gene Ontology - Biological Process	regulation of body fluid levels	experimental (any)	11	5.275	0.040
Gene Ontology - Biological Process	regulation of cytokine secretion	experimental (any)	6	2.046	0.040
Gene Ontology - Biological Process	temperature homeostasis	experimental (any)	6	2.046	0.040
Gene Ontology - Biological Process	ventricular septum morphogenesis	experimental (any)	3	0.545	0.040
Gene Ontology - Biological Process	cellular response to peptide hormone stimulus	predicted (intersection)	68	45.81	0.041
Gene Ontology - Biological Process	heart development	predicted (union)	224	187.8	0.041
Gene Ontology - Biological Process	hippo signaling	predicted (union)	24	13.67	0.041
Gene Ontology - Biological Process	regulation of translation	predicted (union)	146	117.1	0.041
Gene Ontology - Biological Process	roof of mouth development	predicted (union)	47	31.63	0.041
Gene Ontology - Biological Process	Fc receptor signaling pathway	experimental (strong)	6	1.91	0.041
Gene Ontology - Biological Process	leukocyte differentiation	experimental (strong)	12	5.808	0.041

Gene Ontology - Biological Process	stem cell population maintenance	experimental (strong)	6	1.91	0.041
Gene Ontology - Biological Process	regulation of cardiac muscle tissue development	experimental (any)	4	0.997	0.041
Gene Ontology - Biological Process	response to ethanol	experimental (any)	5	1.501	0.041
Gene Ontology - Biological Process	anatomical structure morphogenesis	experimental (strong)	32	22.58	0.042
Gene Ontology - Biological Process	cellular response to DNA damage stimulus	experimental (strong)	12	5.859	0.042
Gene Ontology - Biological Process	cytokine biosynthetic process	experimental (strong)	5	1.394	0.042
Gene Ontology - Biological Process	cytokine metabolic process	experimental (strong)	5	1.394	0.042
Gene Ontology - Biological Process	epithelium development	experimental (strong)	19	11.25	0.042
Gene Ontology - Biological Process	liver regeneration	experimental (strong)	3	0.490	0.042
Gene Ontology - Biological Process	macrophage derived foam cell differentiation	experimental (strong)	3	0.490	0.042

Gene Ontology - Biological Process	neuroinflammatory response	experimental (strong)	4	0.903	0.042
Gene Ontology - Biological Process	outflow tract septum morphogenesis	experimental (strong)	3	0.490	0.042
Gene Ontology - Biological Process	peptide biosynthetic process	experimental (strong)	8	3.123	0.042
Gene Ontology - Biological Process	positive regulation of cyclin-dependent protein kinase activity	experimental (strong)	3	0.490	0.042
Gene Ontology - Biological Process	positive regulation of receptor signaling pathway via JAK-STAT	experimental (strong)	4	0.903	0.042
Gene Ontology - Biological Process	positive regulation of receptor signaling pathway via STAT	experimental (strong)	4	0.903	0.042
Gene Ontology - Biological Process	response to xenobiotic stimulus	experimental (strong)	7	2.504	0.042
Gene Ontology - Biological Process	axon ensheathment	experimental (any)	5	1.515	0.042

Gene Ontology - Biological Process	cellular response to transforming growth factor beta stimulus	experimental (any)	7	2.671	0.042
Gene Ontology - Biological Process	glycosylation	experimental (any)	7	2.671	0.042
Gene Ontology - Biological Process	positive regulation of biomineral tissue development	experimental (any)	3	0.558	0.042
Gene Ontology - Biological Process	regulation of erythrocyte differentiation	experimental (any)	3	0.558	0.042
Gene Ontology - Biological Process	regulation of lymphocyte apoptotic process	experimental (any)	3	0.558	0.042
Gene Ontology - Biological Process	cardiac septum morphogenesis	predicted (intersection)	24	11.99	0.043
Gene Ontology - Biological Process	nuclear transport	predicted (union)	134	106.6	0.043
Gene Ontology - Biological Process	G protein-coupled receptor signaling pathway	experimental (strong)	10	4.465	0.043
Gene Ontology - Biological Process	actin filament polymerization	experimental (strong)	4	0.929	0.043

Gene Ontology - Biological Process	body fluid secretion	experimental (strong)	4	0.929	0.043
Gene Ontology - Biological Process	coronary artery morphogenesis	experimental (strong)	2	0.181	0.043
Gene Ontology - Biological Process	detection of temperature stimulus	experimental (strong)	2	0.181	0.043
Gene Ontology - Biological Process	forebrain development	experimental (strong)	9	3.82	0.043
Gene Ontology - Biological Process	immune response-activating signal transduction	experimental (strong)	10	4.465	0.043
Gene Ontology - Biological Process	leukocyte apoptotic process	experimental (strong)	5	1.42	0.043
Gene Ontology - Biological Process	mesenchymal cell differentiation	experimental (strong)	7	2.555	0.043
Gene Ontology - Biological Process	metal ion transport	experimental (strong)	10	4.491	0.043
Gene Ontology - Biological Process	muscle organ morphogenesis	experimental (strong)	4	0.929	0.043
Gene Ontology - Biological Process	negative regulation of apoptotic signaling pathway	experimental (strong)	8	3.149	0.043

Gene Ontology - Biological Process	negative regulation of calcium ion transport into cytosol	experimental (strong)	2	0.181	0.043
Gene Ontology - Biological Process	negative regulation of transport	experimental (strong)	9	3.794	0.043
Gene Ontology - Biological Process	positive regulation of I-kappaB kinase/NF-kappaB signaling	experimental (strong)	6	1.962	0.043
Gene Ontology - Biological Process	positive regulation of monocyte chemotaxis	experimental (strong)	2	0.181	0.043
Gene Ontology - Biological Process	positive regulation of production of miRNAs involved in gene silencing by miRNA	experimental (strong)	2	0.181	0.043
Gene Ontology - Biological Process	positive regulation of translation	experimental (strong)	4	0.929	0.043
Gene Ontology - Biological Process	regulation of actin filament length	experimental (strong)	4	0.929	0.043

Gene Ontology - Biological Process	regulation of actin polymerization or depolymerization	experimental (strong)	4	0.929	0.043
Gene Ontology - Biological Process	regulation of cell cycle	experimental (strong)	17	9.731	0.043
Gene Ontology - Biological Process	regulation of extracellular matrix disassembly	experimental (strong)	2	0.181	0.043
Gene Ontology - Biological Process	replication fork processing	experimental (strong)	2	0.181	0.043
Gene Ontology - Biological Process	response to temperature stimulus	experimental (strong)	6	1.962	0.043
Gene Ontology - Biological Process	response to wounding	experimental (strong)	13	6.633	0.043
Gene Ontology - Biological Process	sensory perception of mechanical stimulus	experimental (strong)	4	0.929	0.043
Gene Ontology - Biological Process	sensory perception of temperature stimulus	experimental (strong)	2	0.181	0.043
Gene Ontology - Biological Process	telencephalon development	experimental (strong)	7	2.555	0.043
Gene Ontology - Biological Process	actin filament-based process	experimental (any)	14	7.494	0.043

Gene Ontology - Biological Process	coronary vasculature morphogenesis	experimental (any)	2	0.213	0.043
Gene Ontology - Biological Process	detection of biotic stimulus	experimental (any)	2	0.213	0.043
Gene Ontology - Biological Process	endothelial tube morphogenesis	experimental (any)	2	0.213	0.043
Gene Ontology - Biological Process	mammary gland alveolus development	experimental (any)	2	0.213	0.043
Gene Ontology - Biological Process	mammary gland lobule development	experimental (any)	2	0.213	0.043
Gene Ontology - Biological Process	myeloid dendritic cell differentiation	experimental (any)	2	0.213	0.043
Gene Ontology - Biological Process	negative regulation of phagocytosis	experimental (any)	2	0.213	0.043
Gene Ontology - Biological Process	positive regulation of Rho protein signal transduction	experimental (any)	2	0.213	0.043
Gene Ontology - Biological Process	positive regulation of histone H3-K4 methylation	experimental (any)	2	0.213	0.043

Gene Ontology - Biological Process	positive regulation of neuroinflammatory response	experimental (any)	2	0.213	0.043
Gene Ontology - Biological Process	regulation of endopeptidase activity	experimental (any)	9	3.973	0.043
Gene Ontology - Biological Process	regulation of organelle organization	experimental (any)	22	13.7	0.043
Gene Ontology - Biological Process	regulation of tumor necrosis factor production	experimental (any)	5	1.528	0.043
Gene Ontology - Biological Process	endocardial cushion development	experimental (any)	3	0.571	0.044
Gene Ontology - Biological Process	endodermal cell differentiation	experimental (any)	3	0.571	0.044
Gene Ontology - Biological Process	histone ubiquitination	experimental (any)	3	0.571	0.044
Gene Ontology - Biological Process	positive regulation of Notch signaling pathway	experimental (any)	3	0.571	0.044

Gene Ontology - Biological Process	positive regulation of cardiac muscle tissue developm ent	experimen tal (any)	3	0.571	0.044
Gene Ontology - Biological Process	production of miRNAs involved in gene silencing by miRNA	experimen tal (any)	3	0.571	0.044
Gene Ontology - Biological Process	regulation of interleukin-10 production	experimen tal (any)	3	0.571	0.044
Gene Ontology - Biological Process	regulation of epithelial cell proliferatio n	experimen tal (strong)	10	4.543	0.045
Gene Ontology - Biological Process	cardiac ventricle developm ent	experimen tal (any)	5	1.555	0.045
Gene Ontology - Biological Process	icosanoid metabolic process	experimen tal (any)	4	1.036	0.045
Gene Ontology - Biological Process	transformi ng growth factor beta receptor signaling pathway	experimen tal (any)	6	2.126	0.045

Gene Ontology - Biological Process	vascular endothelial growth factor receptor signaling pathway	experimental (any)	4	1.036	0.045
Gene Ontology - Biological Process	cell fate commitment	experimental (strong)	8	3.226	0.046
Gene Ontology - Biological Process	histone acetylation	experimental (strong)	5	1.445	0.046
Gene Ontology - Biological Process	mitotic cell cycle checkpoint	experimental (strong)	5	1.445	0.046
Gene Ontology - Biological Process	negative regulation of locomotion	experimental (strong)	8	3.226	0.046
Gene Ontology - Biological Process	positive regulation of actin filament polymerization	experimental (strong)	3	0.516	0.046
Gene Ontology - Biological Process	programmed necrotic cell death	experimental (strong)	3	0.516	0.046
Gene Ontology - Biological Process	response to dexamethasone	experimental (strong)	3	0.516	0.046
Gene Ontology - Biological Process	BMP signaling pathway	experimental (any)	5	1.568	0.046

Gene Ontology - Biological Process	DNA damage response, signal transduction by p53 class mediator resulting in transcription of p21 class mediator	experimental (any)	2	0.226	0.046
Gene Ontology - Biological Process	I-kappaB phosphorylation	experimental (any)	2	0.226	0.046
Gene Ontology - Biological Process	cellular response to gonadotropin stimulus	experimental (any)	2	0.226	0.046
Gene Ontology - Biological Process	digestive tract morphogenesis	experimental (any)	3	0.585	0.046
Gene Ontology - Biological Process	epithelial to mesenchymal transition involved in endocardial cushion formation	experimental (any)	2	0.226	0.046
Gene Ontology - Biological Process	epithelial tube formation	experimental (any)	5	1.568	0.046

Gene Ontology - Biological Process	mature B cell differentiation involved in immune response	experimental (any)	2	0.226	0.046
Gene Ontology - Biological Process	muscle structure development	experimental (any)	13	6.869	0.046
Gene Ontology - Biological Process	negative regulation of calcium ion transport into cytosol	experimental (any)	2	0.226	0.046
Gene Ontology - Biological Process	negative regulation of tissue remodeling	experimental (any)	2	0.226	0.046
Gene Ontology - Biological Process	peptide transport	experimental (any)	30	20.44	0.046
Gene Ontology - Biological Process	positive regulation of histone deacetylation	experimental (any)	2	0.226	0.046
Gene Ontology - Biological Process	positive regulation of lamellipodium assembly	experimental (any)	2	0.226	0.046

Gene Ontology - Biological Process	positive regulation of transcription of Notch receptor target	experimental (any)	2	0.226	0.046
Gene Ontology - Biological Process	response to corticosterone	experimental (any)	2	0.226	0.046
Gene Ontology - Biological Process	positive regulation of transcription by RNA polymerase II	predicted (union)	415	366.2	0.047
Gene Ontology - Biological Process	cell migration involved in sprouting angiogenesis	experimental (strong)	4	0.955	0.047
Gene Ontology - Biological Process	regulation of translation	experimental (strong)	7	2.607	0.047
Gene Ontology - Biological Process	phosphatidylinositol 3-kinase signaling	experimental (any)	5	1.581	0.047
Gene Ontology - Biological Process	protein complex oligomerization	experimental (any)	12	6.165	0.047
Gene Ontology - Biological Process	protein localization to plasma membrane	experimental (any)	7	2.764	0.047

Gene Ontology - Biological Process	regulation of ion transport	experimental (any)	13	6.883	0.047
Gene Ontology - Biological Process	regulation of organelle assembly	experimental (any)	6	2.152	0.047
Gene Ontology - Biological Process	tumor necrosis factor production	experimental (any)	5	1.581	0.047
Gene Ontology - Biological Process	cardiac muscle cell development	predicted (intersection)	22	10.74	0.048
Gene Ontology - Biological Process	cell-cell signaling	predicted (intersection)	287	240.7	0.048
Gene Ontology - Biological Process	generation of neurons	predicted (intersection)	267	222.2	0.048
Gene Ontology - Biological Process	negative regulation of nucleic acid-templated transcription	predicted (intersection)	218	177.7	0.048
Gene Ontology - Biological Process	response to peptide hormone	predicted (intersection)	88	62.8	0.048
Gene Ontology - Biological Process	transmembrane receptor protein serine/threonine kinase signaling pathway	predicted (intersection)	72	49.56	0.048

Gene Ontology - Biological Process	regulation of neurogenesis	predicted (union)	304	262.4	0.048
Gene Ontology - Biological Process	gene expression	experimental (strong)	42	32.5	0.048
Gene Ontology - Biological Process	peptidyl-serine phosphorylation	experimental (strong)	8	3.252	0.048
Gene Ontology - Biological Process	actin filament bundle assembly	experimental (any)	5	1.594	0.048
Gene Ontology - Biological Process	anterior/posterior pattern specification	experimental (any)	6	2.166	0.048
Gene Ontology - Biological Process	phagocytosis	experimental (any)	7	2.777	0.048
Gene Ontology - Biological Process	positive regulation of cysteine-type endopeptidase activity	experimental (any)	5	1.594	0.048
Gene Ontology - Biological Process	pri-miRNA transcription by RNA polymerase II	experimental (any)	3	0.598	0.048
Gene Ontology - Biological Process	protein modification by small protein removal	experimental (any)	8	3.415	0.048

Gene Ontology - Biological Process	regulation of cellular response to stress	experimental (any)	15	8.424	0.048
Gene Ontology - Biological Process	regulation of hormone secretion	experimental (any)	7	2.777	0.048
Gene Ontology - Biological Process	response to nutrient levels	experimental (any)	11	5.461	0.048
Gene Ontology - Biological Process	regulation of mRNA stability	predicted (union)	72	52.71	0.049
Gene Ontology - Biological Process	actin filament organization	experimental (strong)	7	2.633	0.049
Gene Ontology - Biological Process	response to alcohol	experimental (strong)	7	2.633	0.049
Gene Ontology - Biological Process	tube morphogenesis	experimental (strong)	18	10.69	0.049
Gene Ontology - Biological Process	regulation of cellular protein localization	experimental (any)	12	6.218	0.049
Gene Ontology - Biological Process	regulation of protein complex assembly	experimental (strong)	8	3.278	0.050
Gene Ontology - Biological Process	positive regulation of supramolecular fiber organization	experimental (any)	6	2.192	0.050

Gene Ontology - Biological Process	response to glucose	experimental (any)	6	2.192	0.050
Gene Ontology - Biological Process	roof of mouth development	experimental (any)	4	1.076	0.050
Gene Ontology - Cellular Component	chromosome	predicted (union)	950	805.9	3.10e-9
Gene Ontology - Cellular Component	nuclear lumen	predicted (intersection)	920	773.2	1.09e-8
Gene Ontology - Cellular Component	nuclear chromatin	predicted (union)	732	613	4.01e-8
Gene Ontology - Cellular Component	nuclear chromosome	predicted (union)	788	666.1	4.46e-8
Gene Ontology - Cellular Component	chromatin	predicted (union)	775	659.5	2.25e-7
Gene Ontology - Cellular Component	Golgi apparatus	predicted (union)	590	506	8.37e-5
Gene Ontology - Cellular Component	nuclear chromatin	predicted (intersection)	354	280.9	8.49e-5
Gene Ontology - Cellular Component	nuclear chromosome	predicted (intersection)	381	305.3	8.49e-5

Gene Ontology - Cellular Component	nucleoplasm	predicted (intersection)	640	543.9	8.49e-5
Gene Ontology - Cellular Component	synapse	predicted (intersection)	240	182.3	2.04e-4
Gene Ontology - Cellular Component	chromosome	predicted (intersection)	445	369.3	3.48e-4
Gene Ontology - Cellular Component	chromatin	predicted (intersection)	370	302.2	5.09e-4
Gene Ontology - Cellular Component	synapse	predicted (union)	464	397.9	0.001
Gene Ontology - Cellular Component	protein-containing complex	experimental (strong)	42	23.98	0.001
Gene Ontology - Cellular Component	axon	predicted (intersection)	119	83.74	0.002
Gene Ontology - Cellular Component	cell junction	predicted (intersection)	246	194.5	0.002
Gene Ontology - Cellular Component	cytosol	predicted (intersection)	878	788.5	0.002

Gene Ontology - Cellular Component	cell leading edge	predicted (intersection)	90	60.66	0.003
Gene Ontology - Cellular Component	CD95 death-inducing signaling complex	experimental (any)	3	0.080	0.003
Gene Ontology - Cellular Component	cytoplasmic vesicle	experimental (any)	44	24.29	0.003
Gene Ontology - Cellular Component	endomembrane system	experimental (any)	74	47.09	0.003
Gene Ontology - Cellular Component	intracellular vesicle	experimental (any)	44	24.33	0.003
Gene Ontology - Cellular Component	presynapse	predicted (intersection)	111	79.09	0.004
Gene Ontology - Cellular Component	CD95 death-inducing signaling complex	experimental (strong)	3	0.103	0.005
Gene Ontology - Cellular Component	cell surface	experimental (any)	19	7.255	0.005
Gene Ontology - Cellular Component	death-inducing signaling complex	experimental (any)	3	0.106	0.005

Gene Ontology - Cellular Component	RISC complex	predicted (intersection)	7	1.61	0.007
Gene Ontology - Cellular Component	neuron projection	predicted (intersection)	216	172.7	0.007
Gene Ontology - Cellular Component	Golgi membrane	predicted (union)	292	244.8	0.007
Gene Ontology - Cellular Component	endosome	experimental (any)	22	9.726	0.007
Gene Ontology - Cellular Component	receptor complex	experimental (any)	11	3.083	0.007
Gene Ontology - Cellular Component	vesicle	experimental (any)	61	40.29	0.007
Gene Ontology - Cellular Component	Golgi apparatus	predicted (intersection)	280	231.9	0.009
Gene Ontology - Cellular Component	ruffle membrane	predicted (intersection)	25	12.35	0.009
Gene Ontology - Cellular Component	axon	predicted (union)	222	182.7	0.010

Gene Ontology - Cellular Component	cell leading edge	predicted (union)	166	132.4	0.010
Gene Ontology - Cellular Component	neuron projection	predicted (union)	432	376.8	0.010
Gene Ontology - Cellular Component	receptor complex	experimental (strong)	10	2.607	0.010
Gene Ontology - Cellular Component	activin responsive factor complex	experimental (any)	2	0.040	0.011
Gene Ontology - Cellular Component	death-inducing signaling complex	experimental (strong)	3	0.155	0.012
Gene Ontology - Cellular Component	ruffle	predicted (intersection)	42	25.23	0.014
Gene Ontology - Cellular Component	cell surface	experimental (strong)	15	5.833	0.014
Gene Ontology - Cellular Component	cytoplasm	experimental (any)	141	119.1	0.015
Gene Ontology - Cellular Component	activin responsive factor complex	experimental (strong)	2	0.052	0.017

Gene Ontology - Cellular Component	cytosol	experimental (any)	77	56.51	0.019
Gene Ontology - Cellular Component	plasma membrane bounded cell projection	predicted (intersection)	341	291.7	0.020
Gene Ontology - Cellular Component	extracellular region	experimental (any)	58	39.99	0.023
Gene Ontology - Cellular Component	endomembrane system	predicted (intersection)	737	670.5	0.026
Gene Ontology - Cellular Component	perinuclear region of cytoplasm	predicted (intersection)	145	113.6	0.026
Gene Ontology - Cellular Component	somatodendritic compartment	predicted (intersection)	147	115.6	0.026
Gene Ontology - Cellular Component	synaptonemal complex	experimental (any)	3	0.239	0.026
Gene Ontology - Cellular Component	cell projection	predicted (intersection)	349	301.7	0.028
Gene Ontology - Cellular Component	early endosome	predicted (intersection)	74	52.61	0.028

Gene Ontology - Cellular Component	glutamatergic synapse	predicted (intersection)	79	56.72	0.028
Gene Ontology - Cellular Component	distal axon	predicted (intersection)	61	41.87	0.029
Gene Ontology - Cellular Component	cell junction	predicted (union)	477	424.4	0.034
Gene Ontology - Cellular Component	early endosome	predicted (union)	143	114.8	0.034
Gene Ontology - Cellular Component	ruffle membrane	predicted (union)	41	26.94	0.034
Gene Ontology - Cellular Component	pyruvate dehydrogenase complex	predicted (union)	8	3.124	0.035
Gene Ontology - Cellular Component	SMAD protein complex	experimental (any)	2	0.093	0.035
Gene Ontology - Cellular Component	cell projection membrane	experimental (any)	10	3.455	0.035
Gene Ontology - Cellular Component	female pronucleus	experimental (any)	2	0.093	0.035

Gene Ontology - Cellular Component	lateral element	experimental (any)	2	0.093	0.035
Gene Ontology - Cellular Component	nucleoplasm	experimental (any)	58	41.1	0.035
Gene Ontology - Cellular Component	nucleus	experimental (any)	97	77.92	0.035
Gene Ontology - Cellular Component	secretory granule	experimental (any)	18	8.743	0.035
Gene Ontology - Cellular Component	secretory vesicle	experimental (any)	20	10.08	0.035
Gene Ontology - Cellular Component	vesicle lumen	experimental (any)	10	3.694	0.039
Gene Ontology - Cellular Component	endoplasmic reticulum	experimental (any)	32	19.88	0.042
Gene Ontology - Cellular Component	lateral element	experimental (strong)	2	0.077	0.043
Gene Ontology - Cellular Component	extracellular region	experimental (strong)	31	19.67	0.047

Gene Ontology - Cellular Component	cyclin-dependent protein kinase holoenzyme complex	experimental (any)	3	0.359	0.047
Gene Ontology - Cellular Component	adherens junction	predicted (intersection)	100	76.22	0.049
Gene Ontology - Cellular Component	cell	experimental (any)	180	167.2	0.049
Gene Ontology - Cellular Component	ficolin-1-rich granule membrane	experimental (any)	4	0.731	0.050
Gene Ontology - Molecular Function	DNA binding	predicted (union)	907	763	1.69e-9
Gene Ontology - Molecular Function	DNA-binding transcription factor activity, RNA polymerase II-specific	predicted (union)	675	556.4	1.20e-8
Gene Ontology - Molecular Function	DNA-binding transcription factor activity	predicted (union)	825	695.4	1.24e-8
Gene Ontology - Molecular Function	nucleic acid binding	predicted (intersection)	699	575.3	3.28e-7
Gene Ontology - Molecular Function	DNA binding	predicted (intersection)	443	349.6	3.55e-6

Gene Ontology - Molecular Function	DNA-binding transcription factor activity, RNA polymerase II-specific	predicted (intersection)	334	255	6.70e-6
Gene Ontology - Molecular Function	metal ion binding	predicted (intersection)	752	642.2	1.62e-5
Gene Ontology - Molecular Function	phosphatase activity	predicted (union)	183	134.7	2.81e-5
Gene Ontology - Molecular Function	transcription coregulator activity	predicted (union)	234	179.2	3.05e-5
Gene Ontology - Molecular Function	cation binding	predicted (intersection)	757	651.5	4.44e-5
Gene Ontology - Molecular Function	DNA-binding transcription factor activity	predicted (intersection)	397	318.7	5.98e-5
Gene Ontology - Molecular Function	phosphoric ester hydrolase activity	predicted (union)	232	179.2	6.94e-5
Gene Ontology - Molecular Function	transcription coactivator activity	predicted (union)	145	105.4	1.21e-4
Gene Ontology - Molecular Function	transcription regulatory region sequence-specific DNA binding	predicted (union)	282	224.9	1.21e-4

Gene Ontology - Molecular Function	transcription regulatory region DNA binding	predicted (union)	324	263.9	1.78e-4
Gene Ontology - Molecular Function	double-stranded DNA binding	predicted (union)	320	260.8	1.81e-4
Gene Ontology - Molecular Function	proximal promoter sequence-specific DNA binding	predicted (union)	203	156.6	1.81e-4
Gene Ontology - Molecular Function	sequence-specific double-stranded DNA binding	predicted (union)	293	237	2.08e-4
Gene Ontology - Molecular Function	RNA polymerase II regulatory region sequence-specific DNA binding	predicted (union)	261	208.5	2.09e-4
Gene Ontology - Molecular Function	RNA polymerase II regulatory region DNA binding	predicted (union)	261	208.9	2.34e-4

Gene Ontology - Molecular Function	RNA polymerase II proximal promoter sequence-specific DNA binding	predicted (union)	195	150.7	2.54e-4
Gene Ontology - Molecular Function	purine nucleotide binding	predicted (union)	708	628.2	0.001
Gene Ontology - Molecular Function	purine ribonucleoside triphosphate binding	predicted (union)	678	600.9	0.001
Gene Ontology - Molecular Function	purine ribonucleotide binding	predicted (union)	705	625.9	0.001
Gene Ontology - Molecular Function	RNA binding	predicted (union)	595	525.6	0.002
Gene Ontology - Molecular Function	guanyl ribonucleotide binding	predicted (union)	158	122.6	0.002
Gene Ontology - Molecular Function	nucleoside binding	predicted (union)	157	122.2	0.002
Gene Ontology - Molecular Function	nucleotide binding	predicted (union)	778	700.1	0.002
Gene Ontology - Molecular Function	protein domain specific binding	predicted (union)	281	231.9	0.002
Gene Ontology - Molecular Function	purine nucleoside binding	predicted (union)	154	119.5	0.002

Gene Ontology - Molecular Function	ribonucleoside binding	predicted (union)	155	119.5	0.002
Gene Ontology - Molecular Function	sequence-specific DNA binding	predicted (union)	376	320.6	0.002
Gene Ontology - Molecular Function	GTP binding	predicted (union)	145	112.1	0.003
Gene Ontology - Molecular Function	cytokine receptor binding	experimental (strong)	11	2.4	0.003
Gene Ontology - Molecular Function	DNA-binding transcription activator activity, RNA polymerase II-specific	predicted (union)	174	138.6	0.004
Gene Ontology - Molecular Function	carbohydrate derivative binding	predicted (union)	805	730.2	0.005
Gene Ontology - Molecular Function	cytokine receptor binding	experimental (any)	11	2.339	0.006
Gene Ontology - Molecular Function	protein domain specific binding	predicted (intersection)	144	106.3	0.007
Gene Ontology - Molecular Function	magnesium ion binding	predicted (union)	98	73.02	0.007
Gene Ontology - Molecular Function	enzyme binding	predicted (union)	742	672	0.008

Gene Ontology - Molecular Function	phosphoprotein phosphatase activity	predicted (union)	115	88.24	0.009
Gene Ontology - Molecular Function	MAP kinase activity	experimental (any)	13	3.867	0.009
Gene Ontology - Molecular Function	NF-kappaB-inducing kinase activity	experimental (any)	4	0.266	0.009
Gene Ontology - Molecular Function	Rho GDP-dissociation inhibitor binding	experimental (any)	2	0.027	0.009
Gene Ontology - Molecular Function	cytokine activity	experimental (any)	8	1.608	0.009
Gene Ontology - Molecular Function	protein domain specific binding	experimental (any)	20	8.092	0.009
Gene Ontology - Molecular Function	hydrolase activity, acting on ester bonds	predicted (union)	349	303.4	0.017
Gene Ontology - Molecular Function	GDP-dissociation inhibitor binding	experimental (strong)	2	0.052	0.018
Gene Ontology - Molecular Function	NF-kappaB-inducing kinase activity	experimental (strong)	4	0.387	0.018
Gene Ontology - Molecular Function	Rho GDP-dissociation inhibitor binding	experimental (strong)	2	0.052	0.018

Gene Ontology - Molecular Function	chemokine receptor binding	experimental (strong)	4	0.413	0.018
Gene Ontology - Molecular Function	cytokine activity	experimental (strong)	7	1.549	0.018
Gene Ontology - Molecular Function	signaling receptor activity	experimental (strong)	21	9.886	0.018
Gene Ontology - Molecular Function	tumor necrosis factor receptor binding	experimental (strong)	4	0.439	0.018
Gene Ontology - Molecular Function	GDP-dissociation inhibitor binding	experimental (any)	2	0.040	0.018
Gene Ontology - Molecular Function	protein serine/threonine kinase activity	experimental (any)	20	8.902	0.018
Gene Ontology - Molecular Function	tumor necrosis factor receptor binding	experimental (any)	4	0.385	0.018
Gene Ontology - Molecular Function	cysteine-type endopeptidase activity involved in apoptotic process	experimental (any)	9	2.458	0.019
Gene Ontology - Molecular Function	protein kinase activity	experimental (any)	25	12.56	0.019
Gene Ontology - Molecular Function	phosphoric ester hydrolase activity	predicted (intersection)	113	82.13	0.020

Gene Ontology - Molecular Function	signaling receptor activity	experimental (any)	28	14.92	0.020
Gene Ontology - Molecular Function	lipopolysaccharide receptor activity	experimental (any)	2	0.053	0.021
Gene Ontology - Molecular Function	cysteine-type endopeptidase activity	experimental (any)	10	3.189	0.025
Gene Ontology - Molecular Function	exogenous protein binding	experimental (any)	5	0.877	0.025
Gene Ontology - Molecular Function	mannosyl-oligosaccharide 1,2-alpha-mannosidase activity	experimental (any)	2	0.066	0.025
Gene Ontology - Molecular Function	prostaglandin-E synthase activity	experimental (any)	2	0.066	0.025
Gene Ontology - Molecular Function	signaling receptor binding	experimental (any)	28	15.41	0.025
Gene Ontology - Molecular Function	virus receptor activity	experimental (any)	5	0.864	0.025
Gene Ontology - Molecular Function	chemokine activity	experimental (strong)	3	0.232	0.026
Gene Ontology - Molecular Function	chemokine receptor binding	experimental (any)	4	0.545	0.026

Gene Ontology - Molecular Function	identical protein binding	experimental (any)	32	18.96	0.026
Gene Ontology - Molecular Function	tumor necrosis factor receptor superfamily binding	experimental (any)	4	0.545	0.026
Gene Ontology - Molecular Function	interleukin-1 receptor activity	experimental (any)	2	0.080	0.028
Gene Ontology - Molecular Function	phosphotransferase activity, alcohol group as acceptor	experimental (any)	25	13.74	0.028
Gene Ontology - Molecular Function	small molecule binding	predicted (union)	882	814.1	0.029
Gene Ontology - Molecular Function	lipopolysaccharide receptor activity	experimental (strong)	2	0.077	0.030
Gene Ontology - Molecular Function	pattern recognition receptor activity	experimental (strong)	2	0.077	0.030
Gene Ontology - Molecular Function	signaling pattern recognition receptor activity	experimental (strong)	2	0.077	0.030
Gene Ontology - Molecular Function	tumor necrosis factor receptor superfamily binding	experimental (strong)	4	0.568	0.030

Gene Ontology - Molecular Function	chromatin binding	predicted (union)	186	155	0.034
Gene Ontology - Molecular Function	cytoskeletal protein binding	predicted (union)	312	271.4	0.034
Gene Ontology - Molecular Function	kinase binding	predicted (union)	265	227.6	0.034
Gene Ontology - Molecular Function	CCR5 chemokine receptor binding	experimental (any)	2	0.093	0.034
Gene Ontology - Molecular Function	R-SMAD binding	experimental (any)	3	0.306	0.034
Gene Ontology - Molecular Function	ubiquitin-like protein ligase binding	experimental (any)	10	3.627	0.034
Gene Ontology - Molecular Function	identical protein binding	experimental (strong)	22	12.36	0.043
Gene Ontology - Molecular Function	chemokine activity	experimental (any)	3	0.345	0.044
Gene Ontology - Molecular Function	SH3 domain binding	predicted (intersection)	33	18.79	0.045
Gene Ontology - Molecular Function	carbohydrate derivative binding	predicted (intersection)	388	334.6	0.045
Gene Ontology - Molecular Function	transcription coregulator activity	predicted (intersection)	110	82.13	0.045

Gene Ontology - Molecular Function	transferase activity	predicted (intersection)	471	412.4	0.045
Gene Ontology - Molecular Function	transition metal ion binding	predicted (intersection)	205	166.6	0.045
Gene Ontology - Molecular Function	CXCR chemokine receptor binding	experimental (any)	2	0.133	0.045
Gene Ontology - Molecular Function	DNA binding	experimental (any)	38	25.36	0.045
Gene Ontology - Molecular Function	RNA polymerase II-specific DNA-binding transcription factor binding	experimental (any)	9	3.229	0.045
Gene Ontology - Molecular Function	cytokine receptor activity	experimental (any)	4	0.718	0.045
Gene Ontology - Molecular Function	double-stranded DNA binding	experimental (any)	17	8.676	0.045
Gene Ontology - Molecular Function	enzyme binding	experimental (any)	35	22.85	0.045
Gene Ontology - Molecular Function	kinase activity	experimental (any)	25	14.83	0.045
Gene Ontology - Molecular Function	mannosidase activity	experimental (any)	2	0.133	0.045

Gene Ontology - Molecular Function	signaling pattern recognition receptor activity	experimental (any)	2	0.133	0.045
Gene Ontology - Molecular Function	signaling receptor activator activity	experimental (any)	10	4.013	0.045
Gene Ontology - Molecular Function	thioesterase binding	experimental (any)	2	0.133	0.045
Gene Ontology - Molecular Function	transcription regulatory region DNA binding	experimental (any)	17	8.836	0.045
Gene Ontology - Molecular Function	transforming growth factor beta receptor, cytoplasmic mediator activity	experimental (any)	2	0.133	0.045
Gene Ontology - Molecular Function	type I transforming growth factor beta receptor binding	experimental (any)	2	0.120	0.045
Gene Ontology - Molecular Function	ubiquitin protein ligase binding	experimental (any)	9	3.415	0.045
Gene Ontology - Molecular Function	integrin binding	experimental (any)	5	1.236	0.046

Gene Ontology - Molecular Function	protein heterodimerization activity	experimental (any)	12	5.381	0.046
Gene Ontology - Molecular Function	exogenous protein binding	experimental (strong)	4	0.671	0.047
Gene Ontology - Molecular Function	virus receptor activity	experimental (strong)	4	0.671	0.047
Gene Ontology - Molecular Function	I-SMAD binding	experimental (any)	2	0.146	0.047
Gene Ontology - Molecular Function	catalytic activity	experimental (any)	85	68.39	0.047
Gene Ontology - Molecular Function	histone deacetylase binding	experimental (any)	5	1.262	0.047
Gene Ontology - Molecular Function	pattern recognition receptor activity	experimental (any)	2	0.146	0.047
Gene Ontology - Molecular Function	enzyme binding	experimental (strong)	27	16.83	0.048
KEGG	Toll-like receptor signaling pathway	experimental (any)	15	1.169	6.68e-11
KEGG	Chagas disease (American trypanosomiasis)	experimental (any)	13	1.302	3.20e-8
KEGG	Pathways in cancer	experimental (any)	21	4.159	4.58e-8
KEGG	Hepatitis B	experimental (any)	14	1.727	5.65e-8

KEGG	Rheumatoid arthritis	experimental (any)	11	0.997	9.51e-8
KEGG	Leishmaniasis	experimental (any)	10	0.811	1.35e-7
KEGG	Pertussis	experimental (any)	10	0.877	2.55e-7
KEGG	Malaria	experimental (any)	8	0.505	4.33e-7
KEGG	Measles	experimental (any)	12	1.528	5.31e-7
KEGG	NF-kappa B signaling pathway	experimental (any)	10	1.063	1.18e-6
KEGG	MicroRNAs in cancer	experimental (any)	13	2.073	1.72e-6
KEGG	Pancreatic cancer	experimental (any)	9	0.864	1.78e-6
KEGG	Intestinal immune network for IgA production	experimental (any)	7	0.478	3.60e-6
KEGG	Legionellosis	experimental (any)	8	0.704	3.78e-6
KEGG	Tuberculosis	experimental (any)	12	1.98	5.53e-6
KEGG	Chagas disease (American trypanosomiasis)	experimental (strong)	12	1.807	5.86e-6
KEGG	Rheumatoid arthritis	experimental (strong)	9	0.929	5.86e-6
KEGG	Toll-like receptor signaling pathway	experimental (strong)	12	1.781	5.86e-6
KEGG	Proteoglycans in cancer	experimental (any)	14	2.817	6.83e-6
KEGG	Toxoplasmosis	experimental (any)	10	1.395	9.16e-6

KEGG	Intestinal immune network for IgA production	experimental (strong)	6	0.387	2.68e-5
KEGG	Influenza A	experimental (any)	11	1.953	2.78e-5
KEGG	Herpes simplex infection	experimental (any)	11	2.059	4.20e-5
KEGG	Inflammatory bowel disease (IBD)	experimental (any)	7	0.731	4.20e-5
KEGG	RIG-I-like receptor signaling pathway	experimental (any)	7	0.731	4.20e-5
KEGG	TNF signaling pathway	experimental (any)	9	1.342	4.20e-5
KEGG	Salmonella infection	experimental (any)	8	1.05	5.04e-5
KEGG	Malaria	experimental (strong)	7	0.697	5.12e-5
KEGG	Pathways in cancer	experimental (strong)	19	5.988	5.12e-5
KEGG	Tuberculosis	experimental (strong)	11	2.013	5.12e-5
KEGG	Chemokine signaling pathway	experimental (any)	11	2.179	5.94e-5
KEGG	Colorectal cancer	experimental (any)	7	0.811	7.38e-5
KEGG	Leishmaniasis	experimental (strong)	8	1.058	8.59e-5
KEGG	Pertussis	experimental (strong)	8	1.084	9.24e-5

KEGG	Pancreatic cancer	experimental (strong)	9	1.445	9.38e-5
KEGG	NF-kappa B signaling pathway	experimental (strong)	9	1.471	9.91e-5
KEGG	Hepatitis B	experimental (strong)	12	2.762	1.08e-4
KEGG	Viral myocarditis	experimental (any)	6	0.624	1.58e-4
KEGG	Amoebiasis	experimental (any)	8	1.262	1.67e-4
KEGG	Proteoglycans in cancer	experimental (strong)	14	3.923	1.73e-4
KEGG	Measles	experimental (strong)	10	2.039	1.84e-4
KEGG	MicroRNAs in cancer	experimental (strong)	13	3.562	2.44e-4
KEGG	Apoptosis	experimental (any)	7	1.01	2.79e-4
KEGG	Inflammatory bowel disease (IBD)	experimental (strong)	7	1.007	2.85e-4
KEGG	Salmonella infection	experimental (strong)	7	1.007	2.85e-4
KEGG	Legionellosis	experimental (strong)	6	0.723	3.44e-4
KEGG	Cytokine-cytokine receptor interaction	experimental (any)	11	2.724	3.83e-4
KEGG	Hepatitis C	experimental (any)	8	1.448	4.01e-4
KEGG	Chemokine signaling pathway	experimental (strong)	10	2.323	4.28e-4

KEGG	Toxoplasmosis	experimental (strong)	9	1.936	5.25e-4
KEGG	Viral myocarditis	experimental (strong)	5	0.516	5.85e-4
KEGG	Dorso-ventral axis formation	experimental (any)	4	0.306	7.62e-4
KEGG	Focal adhesion	experimental (any)	10	2.511	7.62e-4
KEGG	FoxO signaling pathway	experimental (any)	8	1.608	7.62e-4
KEGG	Prolactin signaling pathway	experimental (any)	6	0.864	7.62e-4
KEGG	Adherens junction	experimental (any)	6	0.890	8.75e-4
KEGG	Amoebiasis	experimental (strong)	7	1.239	8.77e-4
KEGG	Leukocyte transendothelial migration	experimental (any)	7	1.302	0.001
KEGG	Dorso-ventral axis formation	experimental (strong)	4	0.387	0.002
KEGG	Axon guidance	experimental (any)	7	1.501	0.002
KEGG	Endocytosis	experimental (any)	9	2.485	0.002
KEGG	NOD-like receptor signaling pathway	experimental (any)	5	0.691	0.002
KEGG	Neurotrophin signaling pathway	experimental (any)	7	1.501	0.002
KEGG	PI3K-Akt signaling pathway	experimental (any)	12	3.933	0.002

KEGG	Prion diseases	experimental (any)	4	0.412	0.002
KEGG	Small cell lung cancer	experimental (any)	6	1.076	0.002
KEGG	Herpes simplex infection	experimental (strong)	8	1.962	0.003
KEGG	Epithelial cell signaling in Helicobacter pylori infection	experimental (any)	5	0.811	0.003
KEGG	Hippo signaling pathway	experimental (any)	8	2.02	0.003
KEGG	MAPK signaling pathway	experimental (any)	10	3.176	0.003
KEGG	Osteoclast differentiation	experimental (any)	7	1.581	0.003
KEGG	Shigellosis	experimental (any)	5	0.771	0.003
KEGG	Non-alcoholic fatty liver disease (NAFLD)	experimental (strong)	7	1.626	0.004
KEGG	HTLV-I infection	experimental (any)	10	3.202	0.004
KEGG	T cell receptor signaling pathway	experimental (any)	6	1.276	0.004
KEGG	Adherens junction	experimental (strong)	6	1.265	0.005
KEGG	Colorectal cancer	experimental (strong)	6	1.291	0.005

KEGG	Cytokine-cytokine receptor interaction	experimental (strong)	9	2.71	0.005
KEGG	Epithelial cell signaling in Helicobacter pylori infection	experimental (strong)	5	0.852	0.005
KEGG	Influenza A	experimental (strong)	8	2.194	0.005
KEGG	TNF signaling pathway	experimental (strong)	8	2.194	0.005
KEGG	B cell receptor signaling pathway	experimental (any)	5	0.890	0.005
KEGG	Non-alcoholic fatty liver disease (NAFLD)	experimental (any)	7	1.794	0.005
KEGG	Notch signaling pathway	experimental (any)	4	0.545	0.005
KEGG	Apoptosis	experimental (strong)	6	1.316	0.006
KEGG	Chronic myeloid leukemia	experimental (any)	5	0.957	0.006
KEGG	Cytosolic DNA-sensing pathway	experimental (any)	4	0.585	0.006
KEGG	Fanconi anemia pathway	experimental (any)	4	0.598	0.006
KEGG	Autoimmune thyroid disease	experimental (strong)	3	0.284	0.007

KEGG	Endocytosis	experimental (strong)	7	1.884	0.007
KEGG	FoxO signaling pathway	experimental (strong)	8	2.426	0.007
KEGG	Graft-versus-host disease	experimental (strong)	3	0.284	0.007
KEGG	Leukocyte transendothelial migration	experimental (strong)	6	1.394	0.007
KEGG	NOD-like receptor signaling pathway	experimental (strong)	5	0.955	0.007
KEGG	RIG-I-like receptor signaling pathway	experimental (strong)	5	0.929	0.007
KEGG	Shigellosis	experimental (strong)	5	0.981	0.007
KEGG	TGF-beta signaling pathway	experimental (any)	5	1.023	0.007
KEGG	Other types of O-glycan biosynthesis	experimental (any)	3	0.332	0.008
KEGG	Regulation of actin cytoskeleton	experimental (any)	8	2.525	0.008
KEGG	Viral carcinogenesis	experimental (any)	8	2.538	0.008
KEGG	Cell cycle	experimental (any)	6	1.568	0.010
KEGG	Axon guidance	experimental (strong)	6	1.574	0.011

KEGG	Neurotrophin signaling pathway	experimental (strong)	7	2.065	0.011
KEGG	Notch signaling pathway	experimental (strong)	4	0.697	0.011
KEGG	Non-small cell lung cancer	experimental (any)	4	0.718	0.011
KEGG	Allograft rejection	experimental (strong)	3	0.361	0.012
KEGG	Fanconi anemia pathway	experimental (strong)	3	0.361	0.012
KEGG	Small cell lung cancer	experimental (strong)	6	1.626	0.012
KEGG	Allograft rejection	experimental (any)	3	0.385	0.012
KEGG	Graft-versus-host disease	experimental (any)	3	0.385	0.012
KEGG	PI3K-Akt signaling pathway	experimental (strong)	11	4.646	0.013
KEGG	African trypanosomiasis	experimental (any)	3	0.399	0.013
KEGG	Prion diseases	experimental (strong)	3	0.413	0.016
KEGG	Natural killer cell mediated cytotoxicity	experimental (any)	5	1.302	0.017
KEGG	Wnt signaling pathway	experimental (any)	6	1.794	0.017
KEGG	Autoimmune thyroid disease	experimental (any)	3	0.452	0.018

KEGG	p53 signaling pathway	experimental (any)	4	0.864	0.019
KEGG	Focal adhesion	experimental (strong)	8	2.994	0.020
KEGG	Phagosome	experimental (strong)	4	0.852	0.020
KEGG	Staphylococcus aureus infection	experimental (strong)	2	0.155	0.020
KEGG	African trypanosomiasis	experimental (strong)	3	0.465	0.021
KEGG	Hepatitis C	experimental (strong)	6	1.884	0.021
KEGG	Cell cycle	experimental (strong)	6	1.91	0.022
KEGG	TGF-beta signaling pathway	experimental (strong)	5	1.368	0.022
KEGG	Staphylococcus aureus infection	experimental (any)	3	0.492	0.022
KEGG	Prolactin signaling pathway	experimental (strong)	5	1.394	0.023
KEGG	Bladder cancer	experimental (any)	3	0.505	0.023
KEGG	Cell adhesion molecules (CAMs)	experimental (any)	5	1.448	0.025
KEGG	Cytosolic DNA-sensing pathway	experimental (strong)	3	0.516	0.026
KEGG	Pathogenic Escherichia coli infection	experimental (strong)	3	0.542	0.029

KEGG	Jak-STAT signaling pathway	experimental (any)	5	1.555	0.032
KEGG	N-Glycan biosynthesis	experimental (any)	3	0.624	0.039
KEGG	Prostate cancer	experimental (any)	4	1.103	0.039
KEGG	Chronic myeloid leukemia	experimental (strong)	5	1.626	0.040
KEGG	Alzheimer's disease	experimental (strong)	4	1.11	0.042
KEGG	Phagosome	experimental (any)	5	1.687	0.042
KEGG	Endometrial cancer	experimental (any)	3	0.664	0.043
KEGG	Pathogenic Escherichia coli infection	experimental (any)	3	0.664	0.043
KEGG	Cell adhesion molecules (CAMs)	experimental (strong)	4	1.136	0.044
KEGG	MAPK signaling pathway	experimental (strong)	8	3.588	0.044
KEGG	Non-small cell lung cancer	experimental (strong)	4	1.161	0.047
KEGG	HTLV-I infection	experimental (strong)	8	3.691	0.049
KEGG	Epstein-Barr virus infection	experimental (any)	6	2.392	0.050
Reactome	Cytokine Signaling in Immune system	experimental (any)	38	9.793	1.55e-10

Reactome	Immune System	experimental (any)	60	23.6	5.65e-10
Reactome	Interferon alpha/beta signaling	experimental (any)	11	0.731	1.53e-8
Reactome	Gene expression (Transcription)	predicted (union)	614	508	3.53e-7
Reactome	Generic Transcription Pathway	predicted (union)	508	413.1	3.53e-7
Reactome	RNA Polymerase II Transcription	predicted (union)	557	457.6	3.53e-7
Reactome	Signaling by Interleukins	experimental (any)	26	7.334	1.89e-6
Reactome	Innate Immune System	experimental (any)	31	11.65	2.90e-5
Reactome	Interferon Signaling	experimental (any)	13	2.285	2.90e-5
Reactome	Toll Like Receptor 4 (TLR4) Cascade	experimental (any)	11	1.541	2.90e-5
Reactome	Interleukin-4 and Interleukin-13 signaling	experimental (any)	10	1.355	4.65e-5
Reactome	Toll-like Receptor Cascades	experimental (any)	11	1.873	1.16e-4
Reactome	MyD88-independent TLR4 cascade	experimental (any)	9	1.222	1.21e-4

Reactome	TRIF(TICAM1)-mediated TLR4 signaling	experimental (any)	9	1.222	1.21e-4
Reactome	Immune System	experimental (strong)	35	15.95	1.24e-4
Reactome	Gene expression (Transcription)	predicted (intersection)	305	232.8	1.25e-4
Reactome	Death Receptor Signalling	experimental (any)	10	1.634	1.72e-4
Reactome	Signal Transduction	experimental (any)	49	26.22	2.30e-4
Reactome	RNA Polymerase II Transcription	predicted (intersection)	275	209.7	2.63e-4
Reactome	Generic Transcription Pathway	predicted (intersection)	250	189.3	3.44e-4
Reactome	NF-kB is activated and signals survival	experimental (any)	4	0.159	3.49e-4
Reactome	p75 NTR receptor-mediated signalling	experimental (any)	8	1.103	3.49e-4
Reactome	Synthesis of PIPs at the plasma membrane	predicted (union)	35	18.35	3.76e-4

Reactome	DDX58/IFIH1-mediated induction of interferon-alpha/beta	experimental (any)	7	0.850	4.13e-4
Reactome	Defective LFNG causes SCDO3	experimental (any)	3	0.066	4.13e-4
Reactome	Interleukin-10 signaling	experimental (any)	6	0.558	4.13e-4
Reactome	MyD88 dependent cascade initiated on endosome	experimental (any)	8	1.169	4.13e-4
Reactome	MyD88:MAL(TIRAP) cascade initiated on plasma membrane	experimental (any)	8	1.222	4.13e-4
Reactome	TAK1 activates NFkB by phosphorylation and activation of IKKs complex	experimental (any)	5	0.372	4.13e-4
Reactome	Toll Like Receptor 2 (TLR2) Cascade	experimental (any)	8	1.222	4.13e-4
Reactome	Toll Like Receptor 3 (TLR3) Cascade	experimental (any)	8	1.183	4.13e-4

Reactome	Toll Like Receptor 7/8 (TLR7/8) Cascade	experimental (any)	8	1.169	4.13e-4
Reactome	Toll Like Receptor 9 (TLR9) Cascade	experimental (any)	8	1.222	4.13e-4
Reactome	Toll Like Receptor TLR1:TLR2 Cascade	experimental (any)	8	1.222	4.13e-4
Reactome	Toll Like Receptor TLR6:TLR2 Cascade	experimental (any)	8	1.222	4.13e-4
Reactome	p75NTR signals via NF-kB	experimental (any)	4	0.199	4.85e-4
Reactome	Loss of Function of SMAD2/3 in Cancer	experimental (any)	3	0.093	9.89e-4
Reactome	Innate Immune System	experimental (strong)	21	7.588	0.001
Reactome	Chemokine receptors bind chemokines	experimental (any)	5	0.505	0.002
Reactome	SHC1 events in ERBB2 signaling	experimental (any)	4	0.279	0.002
Reactome	Signaling by TGF-beta Receptor Complex in Cancer	experimental (any)	3	0.106	0.002

Reactome	TRAF6 mediated IRF7 activation in TLR7/8 or 9 signaling	experimental (any)	3	0.120	0.002
Reactome	TRAF6 mediated induction of NFkB and MAP kinases upon TLR7/8 or 9 activation	experimental (any)	7	1.156	0.002
Reactome	CD28 co-stimulation	predicted (union)	23	11.32	0.003
Reactome	Membrane Trafficking	predicted (union)	262	214	0.003
Reactome	Regulation of MECP2 expression and activity	predicted (union)	23	11.32	0.003
Reactome	Transcriptional Regulation by MECP2	predicted (union)	40	23.04	0.003
Reactome	Vesicle-mediated transport	predicted (union)	276	227.2	0.003
Reactome	Interleukin-10 signaling	experimental (strong)	6	0.671	0.003
Reactome	Signal Transduction	experimental (strong)	36	19.93	0.003
Reactome	Interleukin-1 family signaling	experimental (any)	8	1.727	0.003

Reactome	Interleukin-1 signaling	experimental (any)	7	1.302	0.003
Reactome	PI5P, PP2A and IER3 Regulate PI3K/AKT Signaling	experimental (any)	7	1.289	0.003
Reactome	Death Receptor Signalling	experimental (strong)	8	1.445	0.004
Reactome	Loss of Function of SMAD4 in Cancer	experimental (any)	2	0.040	0.004
Reactome	Negative regulation of the PI3K/AKT network	experimental (any)	7	1.382	0.004
Reactome	Neutrophil degranulation	experimental (any)	15	5.554	0.004
Reactome	SMAD2/3 MH2 Domain Mutants in Cancer	experimental (any)	2	0.040	0.004
Reactome	SMAD4 MH2 Domain Mutants in Cancer	experimental (any)	2	0.040	0.004
Reactome	Signaling by ERBB2	experimental (any)	5	0.651	0.004
Reactome	p75NTR recruits signalling complexes	experimental (any)	3	0.159	0.004

Reactome	Neuronal System	predicted (intersection)	95	62.98	0.005
Reactome	Activation of RAC1	experimental (any)	3	0.173	0.005
Reactome	Caspase activation via Death Receptors in the presence of ligand	experimental (any)	3	0.186	0.005
Reactome	ERBB2 Regulates Cell Motility	experimental (any)	3	0.186	0.005
Reactome	IRAK2 mediated activation of TAK1 complex upon TLR7/8 or 9 stimulation	experimental (any)	3	0.186	0.005
Reactome	Pre-NOTCH Processing in Golgi	experimental (any)	3	0.186	0.005
Reactome	Signaling by Non-Receptor Tyrosine Kinases	experimental (any)	5	0.678	0.005
Reactome	Signaling by PTK6	experimental (any)	5	0.678	0.005
Reactome	Neurotransmitter release cycle	predicted (intersection)	21	8.41	0.006
Reactome	Signaling by Non-Receptor Tyrosine Kinases	predicted (intersection)	21	8.589	0.006

Reactome	Signaling by PTK6	predicted (intersection)	21	8.589	0.006
Reactome	Transcriptional Regulation by MECP2	predicted (intersection)	24	10.56	0.006
Reactome	Diseases of signal transduction	predicted (union)	173	136.3	0.006
Reactome	Defective LFNG causes SCDO3	experimental (strong)	3	0.129	0.006
Reactome	Signaling by Interleukins	experimental (strong)	19	7.924	0.006
Reactome	p75 NTR receptor-mediated signalling	experimental (strong)	6	0.826	0.006
Reactome	GRB2 events in ERBB2 signaling	experimental (any)	3	0.199	0.006
Reactome	MyD88 cascade initiated on plasma membrane	experimental (any)	6	1.103	0.006
Reactome	TRAF6-mediated induction of TAK1 complex within TLR4 complex	experimental (any)	3	0.199	0.006
Reactome	Toll Like Receptor 10 (TLR10) Cascade	experimental (any)	6	1.103	0.006

Reactome	Toll Like Receptor 5 (TLR5) Cascade	experimental (any)	6	1.103	0.006
Reactome	DAP12 interactions	experimental (any)	4	0.465	0.007
Reactome	FCERI mediated MAPK activation	experimental (any)	4	0.465	0.007
Reactome	SLIT2:ROBO1 increases RHOA activity	experimental (any)	2	0.053	0.007
Reactome	MECP2 regulates neuronal receptors and channels	predicted (intersection)	11	3.221	0.008
Reactome	Signaling by ERBB2	predicted (intersection)	20	8.231	0.008
Reactome	PI Metabolism	predicted (union)	46	28.89	0.008
Reactome	MAP kinase activation	experimental (any)	5	0.824	0.008
Reactome	PIP3 activates AKT signaling	predicted (union)	123	93.32	0.009
Reactome	CD28 dependent PI3K/Akt signaling	predicted (union)	16	7.419	0.010
Reactome	Cell death signalling via NRAGE, NRIF and NADE	experimental (any)	5	0.864	0.010

Reactome	FasL/ CD95L signaling	experimental (any)	2	0.066	0.010
Reactome	Interleukin-17 signaling	experimental (any)	5	0.890	0.010
Reactome	Interleukin-36 pathway	experimental (any)	2	0.066	0.010
Reactome	JNK (c-Jun kinases) phosphorylation and activation mediated by activated human TAK1	experimental (any)	3	0.239	0.010
Reactome	Signal transduction by L1	experimental (any)	3	0.252	0.010
Reactome	Signaling by SCF-KIT	experimental (any)	4	0.531	0.010
Reactome	TRAF6 mediated IRF7 activation	experimental (any)	3	0.239	0.010
Reactome	activated TAK1 mediates p38 MAPK activation	experimental (any)	3	0.252	0.010
Reactome	Netrin-1 signaling	predicted (union)	31	17.96	0.012
Reactome	Generic Transcription Pathway	experimental (any)	26	14.32	0.012

Reactome	Pre-NOTCH Processing in the Endoplasmic Reticulum	experimental (any)	2	0.080	0.013
Reactome	SMAD2/3 Phosphorylation Motif Mutants in Cancer	experimental (any)	2	0.080	0.013
Reactome	TGFBR1 KD Mutants in Cancer	experimental (any)	2	0.080	0.013
Reactome	TRIF-mediated programmed cell death	experimental (any)	2	0.080	0.013
Reactome	Chemokine receptors bind chemokines	experimental (strong)	4	0.413	0.014
Reactome	Cytokine Signaling in Immune system	experimental (strong)	20	9.395	0.014
Reactome	Loss of Function of SMAD2/3 in Cancer	experimental (strong)	3	0.181	0.014
Reactome	NF-kB is activated and signals survival	experimental (strong)	3	0.181	0.014

Reactome	Signaling by TGF-beta Receptor Complex in Cancer	experimental (strong)	3	0.181	0.014
Reactome	Intracellular signaling by second messengers	predicted (union)	138	107.8	0.015
Reactome	Transport to the Golgi and subsequent modification	predicted (union)	81	58.57	0.015
Reactome	Neutrophil degranulation	experimental (strong)	10	3.02	0.015
Reactome	Anchoring fibril formation	experimental (any)	2	0.093	0.015
Reactome	Caspase activation via extrinsic apoptotic signalling pathway	experimental (any)	3	0.306	0.015
Reactome	DEx/H-box helicases activate type I IFN and inflammatory cytokines production	experimental (any)	2	0.093	0.015

Reactome	Diseases associated with the TLR signaling cascade	experimental (any)	3	0.306	0.015
Reactome	Diseases of Immune System	experimental (any)	3	0.306	0.015
Reactome	Inactivation of CDC42 and RAC1	experimental (any)	2	0.093	0.015
Reactome	Loss of Function of TGFBR1 in Cancer	experimental (any)	2	0.093	0.015
Reactome	Nucleotide-binding domain, leucine rich repeat containing receptor (NLR) signaling pathways	experimental (any)	4	0.624	0.015
Reactome	RUNX3 regulates CDKN1A transcription	experimental (any)	2	0.093	0.015
Reactome	Sema4D in semaphorin signaling	experimental (any)	3	0.306	0.015

Reactome	Senescence-Associated Secretory Phenotype (SASP)	experimental (any)	5	0.983	0.015
Reactome	Antiviral mechanism by IFN-stimulated genes	experimental (any)	5	1.036	0.016
Reactome	Estrogen-dependent nuclear events downstream of ESR-membrane signaling	experimental (any)	3	0.319	0.016
Reactome	Interferon gamma signaling	experimental (any)	5	1.05	0.016
Reactome	Signaling by Receptor Tyrosine Kinases	experimental (any)	13	5.554	0.016
Reactome	TRAF6 mediated NF-kB activation	experimental (any)	3	0.319	0.016
Reactome	p75NTR signals via NF-kB	experimental (strong)	3	0.206	0.017
Reactome	SHC1 events in ERBB2 signaling	experimental (strong)	4	0.465	0.018

Reactome	RNA Polymerase II Transcription	experimental (any)	27	15.85	0.018
Reactome	ERBB2 Regulates Cell Motility	experimental (strong)	3	0.232	0.019
Reactome	IRAK2 mediated activation of TAK1 complex upon TLR7/8 or 9 stimulation	experimental (strong)	3	0.232	0.019
Reactome	Signaling by ERBB2	experimental (strong)	5	0.826	0.019
Reactome	TRAF6-mediated induction of TAK1 complex within TLR4 complex	experimental (strong)	3	0.232	0.019
Reactome	DAP12 signaling	experimental (any)	3	0.345	0.019
Reactome	Sema4D mediated inhibition of cell attachment and migration	experimental (any)	2	0.106	0.019
Reactome	Transcriptional regulation of pluripotent stem cells	experimental (any)	3	0.345	0.019

Reactome	Interleukin-4 and Interleukin-13 signaling	experimental (strong)	8	2.22	0.020
Reactome	Intracellular signaling by second messengers	experimental (any)	10	3.827	0.020
Reactome	Signaling by ERBB4	experimental (any)	4	0.704	0.020
Reactome	GPCR downstream signalling	experimental (strong)	11	4.104	0.021
Reactome	Loss of Function of SMAD4 in Cancer	experimental (strong)	2	0.077	0.021
Reactome	Pre-NOTCH Processing in Golgi	experimental (strong)	3	0.258	0.021
Reactome	SMAD2/3 MH2 Domain Mutants in Cancer	experimental (strong)	2	0.077	0.021
Reactome	SMAD4 MH2 Domain Mutants in Cancer	experimental (strong)	2	0.077	0.021
Reactome	Signaling by Non-Receptor Tyrosine Kinases	experimental (strong)	5	0.929	0.021

Reactome	Signaling by PTK6	experimental (strong)	5	0.929	0.021
Reactome	TAK1 activates NFkB by phosphorylation and activation of IKKs complex	experimental (strong)	4	0.568	0.021
Reactome	Toll Like Receptor 4 (TLR4) Cascade	experimental (strong)	7	1.807	0.021
Reactome	Netrin-1 signaling	predicted (intersection)	19	8.231	0.022
Reactome	Signaling by EGFR	predicted (intersection)	19	8.231	0.022
Reactome	DAP12 interactions	predicted (union)	25	14.06	0.022
Reactome	EGFR Transactivation by Gastrin	experimental (any)	2	0.120	0.022
Reactome	MyD88 deficiency (TLR2/4)	experimental (any)	2	0.120	0.022
Reactome	Regulated proteolysis of p75NTR	experimental (any)	2	0.120	0.022
Reactome	Transcriptional Regulation by MECP2	experimental (any)	4	0.731	0.022
Reactome	NOD1/2 Signaling Pathway	experimental (any)	3	0.385	0.024

Reactome	Signaling by ROBO receptors	experimental (strong)	5	0.981	0.025
Reactome	CASP8 activity is inhibited	experimental (any)	2	0.133	0.025
Reactome	Dimerization of procaspase-8	experimental (any)	2	0.133	0.025
Reactome	Fc epsilon receptor (FCERI) signaling	experimental (any)	6	1.714	0.025
Reactome	IRAK1 recruits IKK complex	experimental (any)	2	0.133	0.025
Reactome	IRAK1 recruits IKK complex upon TLR7/8 or 9 stimulation	experimental (any)	2	0.133	0.025
Reactome	IRAK2 mediated activation of TAK1 complex	experimental (any)	2	0.133	0.025
Reactome	IRAK4 deficiency (TLR2/4)	experimental (any)	2	0.133	0.025
Reactome	PIP3 activates AKT signaling	experimental (any)	9	3.362	0.025
Reactome	RUNX3 regulates p14-ARF	experimental (any)	2	0.133	0.025
Reactome	Regulation by c-FLIP	experimental (any)	2	0.133	0.025

Reactome	EPHB-mediated forward signaling	experimental (any)	3	0.412	0.026
Reactome	Negative regulation of the PI3K/AKT network	predicted (union)	58	40.22	0.027
Reactome	Transcriptional regulation by RUNX3	experimental (any)	5	1.249	0.027
Reactome	Caspase activation via Death Receptors in the presence of ligand	experimental (strong)	3	0.310	0.028
Reactome	GRB2 events in ERBB2 signaling	experimental (strong)	3	0.310	0.028
Reactome	GPCR downstream signalling	experimental (any)	15	7.52	0.028
Reactome	Gene expression (Transcription)	experimental (any)	28	17.55	0.028
Reactome	Interleukin-6 signaling	experimental (any)	2	0.146	0.028
Reactome	RHO GTPases activate KTN1	experimental (any)	2	0.146	0.028
Reactome	TGF-beta receptor signaling activates SMADs	experimental (any)	3	0.425	0.028

Reactome	Asparagine N-linked glycosylation	predicted (union)	128	100.7	0.029
Reactome	Signaling by NTRKs	predicted (union)	52	35.53	0.029
Reactome	Signaling by Receptor Tyrosine Kinases	predicted (union)	197	162.8	0.029
Reactome	CD28 co-stimulation	experimental (any)	3	0.438	0.029
Reactome	FasL/ CD95L signaling	experimental (strong)	2	0.103	0.030
Reactome	MyD88:MAL(TIRAP) cascade initiated on plasma membrane	experimental (strong)	6	1.549	0.030
Reactome	Pre-NOTCH Processing in the Endoplasmic Reticulum	experimental (strong)	2	0.103	0.030
Reactome	Synthesis of Prostaglandins (PG) and Thromboxanes (TX)	experimental (strong)	2	0.103	0.030
Reactome	Toll Like Receptor 2 (TLR2) Cascade	experimental (strong)	6	1.549	0.030

Reactome	Toll Like Receptor TLR1:TLR2 Cascade	experimental (strong)	6	1.549	0.030
Reactome	Toll Like Receptor TLR6:TLR2 Cascade	experimental (strong)	6	1.549	0.030
Reactome	Signaling by ERBB4	predicted (intersection)	20	9.125	0.031
Reactome	MyD88-independent TLR4 cascade	experimental (strong)	6	1.6	0.031
Reactome	PI5P, PP2A and IER3 Regulate PI3K/AKT Signaling	experimental (strong)	6	1.6	0.031
Reactome	Signaling by NOTCH	experimental (strong)	7	2.091	0.031
Reactome	TRIF(TICAM1)-mediated TLR4 signaling	experimental (strong)	6	1.6	0.031
Reactome	Toll-like Receptor Cascades	experimental (strong)	7	2.091	0.031
Reactome	Diseases associated with O-glycosylation of proteins	experimental (strong)	3	0.361	0.032
Reactome	CD28 dependent Vav1 pathway	experimental (any)	2	0.159	0.032

Reactome	ERBB2 Activates PTK6 Signaling	experimental (any)	2	0.159	0.032
Reactome	Signaling by GPCR	experimental (strong)	11	4.62	0.034
Reactome	Activation of NMDA receptors and postsynaptic events	predicted (intersection)	25	12.7	0.035
Reactome	Competing endogenous RNAs (ceRNAs) regulate PTEN translation	predicted (intersection)	7	1.789	0.035
Reactome	Intracellular signaling by second messengers	predicted (intersection)	72	49.38	0.035
Reactome	Negative regulation of the PI3K/AKT network	predicted (intersection)	33	18.43	0.035
Reactome	PIP3 activates AKT signaling	predicted (intersection)	64	42.76	0.035
Reactome	Deubiquitination	experimental (strong)	7	2.246	0.035
Reactome	Developmental Biology	experimental (strong)	16	8.182	0.035

Reactome	Generic Transcription Pathway	experimental (strong)	18	9.782	0.035
Reactome	JNK (c-Jun kinases) phosphorylation and activation mediated by activated human TAK1	experimental (strong)	3	0.387	0.035
Reactome	MyD88 deficiency (TLR2/4)	experimental (strong)	2	0.129	0.035
Reactome	TRIF-mediated programmed cell death	experimental (strong)	2	0.129	0.035
Reactome	activated TAK1 mediates p38 MAPK activation	experimental (strong)	3	0.387	0.035
Reactome	p75NTR recruits signalling complexes	experimental (strong)	2	0.129	0.035
Reactome	FCERI mediated MAPK activation	predicted (union)	19	10.15	0.036
Reactome	Caspase activation via extrinsic apoptotic signalling pathway	experimental (strong)	3	0.439	0.036

Reactome	Class A/1 (Rhodopsin-like receptors)	experimental (strong)	5	1.265	0.036
Reactome	Disease	experimental (strong)	16	8.621	0.036
Reactome	Diseases associated with the TLR signaling cascade	experimental (strong)	3	0.413	0.036
Reactome	Diseases of Immune System	experimental (strong)	3	0.413	0.036
Reactome	Gene expression (Transcription)	experimental (strong)	19	10.79	0.036
Reactome	IRAK1 recruits IKK complex	experimental (strong)	2	0.155	0.036
Reactome	IRAK1 recruits IKK complex upon TLR7/8 or 9 stimulation	experimental (strong)	2	0.155	0.036
Reactome	IRAK2 mediated activation of TAK1 complex	experimental (strong)	2	0.155	0.036
Reactome	IRAK4 deficiency (TLR2/4)	experimental (strong)	2	0.155	0.036

Reactome	Loss of Function of TGFBR1 in Cancer	experimental (strong)	2	0.155	0.036
Reactome	Negative regulation of the PI3K/AKT network	experimental (strong)	6	1.755	0.036
Reactome	Nucleotide-binding domain, leucine rich repeat containing receptor (NLR) signaling pathways	experimental (strong)	4	0.852	0.036
Reactome	Peptide ligand-binding receptors	experimental (strong)	4	0.774	0.036
Reactome	Pre-NOTCH Expression and Processing	experimental (strong)	4	0.800	0.036
Reactome	RHO GTPases activate KTN1	experimental (strong)	2	0.155	0.036
Reactome	RNA Polymerase II Transcription	experimental (strong)	18	10.01	0.036
Reactome	RUNX3 regulates CDKN1A transcription	experimental (strong)	2	0.155	0.036

Reactome	SMAD2/3 Phosphorylation Motif Mutants in Cancer	experimental (strong)	2	0.155	0.036
Reactome	Sema4D in semaphorin signaling	experimental (strong)	3	0.413	0.036
Reactome	Senescence-Associated Secretory Phenotype (SASP)	experimental (strong)	4	0.826	0.036
Reactome	Signaling by ERBB4	experimental (strong)	4	0.826	0.036
Reactome	TGF-beta receptor signaling activates SMADs	experimental (strong)	3	0.439	0.036
Reactome	TGFBR1 KD Mutants in Cancer	experimental (strong)	2	0.155	0.036
Reactome	GRB2 events in EGFR signaling	experimental (any)	2	0.173	0.036
Reactome	NRIF signals cell death from the nucleus	experimental (any)	2	0.173	0.036
Reactome	RIPK1-mediated regulated necrosis	experimental (any)	2	0.173	0.036
Reactome	Regulated Necrosis	experimental (any)	2	0.173	0.036

Reactome	Regulation of necroptotic cell death	experimental (any)	2	0.173	0.036
Reactome	SHC1 events in ERBB4 signaling	experimental (any)	2	0.173	0.036
Reactome	Signaling by Activin	experimental (any)	2	0.173	0.036
Reactome	Signaling by NOTCH	experimental (any)	7	2.485	0.036
Reactome	TRAF3-dependent IRF activation pathway	experimental (any)	2	0.173	0.036
Reactome	Costimulation by the CD28 family	predicted (union)	37	23.82	0.038
Reactome	Signaling by ERBB4	predicted (union)	32	19.91	0.038
Reactome	EPHB-mediated forward signaling	experimental (strong)	3	0.465	0.038
Reactome	Signal transduction by L1	experimental (strong)	3	0.465	0.038
Reactome	Immunoregulatory interactions between a Lymphoid and a non-Lymphoid cell	experimental (any)	5	1.408	0.038

Reactome	Constitutive Signaling by Aberrant PI3K in Cancer	experimental (any)	4	0.943	0.039
Reactome	EPHA-mediated growth cone collapse	experimental (any)	2	0.186	0.039
Reactome	G alpha (12/13) signalling events	experimental (any)	4	0.930	0.039
Reactome	ISG15 antiviral mechanism	experimental (any)	4	0.930	0.039
Reactome	PTK6 Regulates RHO GTPases, RAS GTPase and MAP kinases	experimental (any)	2	0.186	0.039
Reactome	SHC1 events in EGFR signaling	experimental (any)	2	0.186	0.039
Reactome	Synthesis of Prostaglandins (PG) and Thromboxanes (TX)	experimental (any)	2	0.186	0.039
Reactome	Role of LAT2/NTAL/LAB on calcium mobilization	predicted (union)	10	4.295	0.041

Reactome	Pre-NOTCH Expression and Processing	experimental (any)	4	0.957	0.041
Reactome	Activation of RAC1	experimental (strong)	2	0.181	0.042
Reactome	Cell death signalling via NRAGE, NRIF and NADE	experimental (strong)	3	0.490	0.042
Reactome	DDX58/IFIH1-mediated induction of interferon-alpha/beta	experimental (strong)	4	0.929	0.042
Reactome	G alpha (12/13) signalling events	experimental (strong)	3	0.490	0.042
Reactome	Intracellular signaling by second messengers	experimental (strong)	9	3.794	0.042
Reactome	Regulated proteolysis of p75NTR	experimental (strong)	2	0.181	0.042
Reactome	Resolution of D-Loop Structures	experimental (strong)	2	0.181	0.042

Reactome	Resolution of D-loop Structures through Holliday Junction Intermediates	experimental (strong)	2	0.181	0.042
Reactome	Resolution of D-loop Structures through Synthesis-Dependent Strand Annealing (SDSA)	experimental (strong)	2	0.181	0.042
Reactome	Sema4D mediated inhibition of cell attachment and migration	experimental (strong)	2	0.181	0.042
Reactome	TRAF6 mediated IRF7 activation in TLR7/8 or 9 signaling	experimental (strong)	2	0.181	0.042
Reactome	Transcriptional regulation of pluripotent stem cells	experimental (strong)	3	0.490	0.042

Reactome	Activation of IRF3/IRF7 mediated by TBK1/IKK epsilon	experimental (any)	2	0.199	0.042
Reactome	Constitutive Signaling by EGFRvIII	experimental (any)	2	0.199	0.042
Reactome	PI3K events in ERBB2 signaling	experimental (any)	2	0.199	0.042
Reactome	Regulation of TLR by endogenous ligand	experimental (any)	2	0.199	0.042
Reactome	Signaling by EGFRvIII in Cancer	experimental (any)	2	0.199	0.042
Reactome	Signaling by TGF-beta Receptor Complex	experimental (any)	4	0.970	0.042
Reactome	PI5P, PP2A and IER3 Regulate PI3K/AKT Signaling	predicted (intersection)	31	17.36	0.043
Reactome	PI5P, PP2A and IER3 Regulate PI3K/AKT Signaling	predicted (union)	54	37.87	0.044
Reactome	Signaling by GPCR	experimental (any)	15	8.172	0.044

Reactome	Signaling by Receptor Tyrosine Kinases	predicted (intersection)	101	74.61	0.045
Reactome	Phospholipid metabolism	predicted (union)	95	73.02	0.045
Reactome	Signaling by NTRK2 (TRKB)	predicted (union)	17	8.981	0.046
Reactome	Cellular Senescence	experimental (any)	6	2.099	0.046
Reactome	FOXO-mediated transcription of cell cycle genes	experimental (any)	2	0.213	0.046
Reactome	G alpha (i) signalling events	experimental (any)	9	3.986	0.046
Reactome	TGF-beta receptor signaling in EMT (epithelial to mesenchymal transition)	experimental (any)	2	0.213	0.046

Reactome	Transcription of E2F targets under negative control by p107 (RBL1) and p130 (RBL2) in complex with HDAC1	experimental (any)	2	0.213	0.046
Reactome	Transcriptional regulation by RUNX2	experimental (any)	5	1.528	0.046
Reactome	Deubiquitination	experimental (any)	8	3.362	0.048
Reactome	Cell surface interactions at the vascular wall	experimental (any)	5	1.568	0.049
Reactome	Gastrin-CREB signalling pathway via PKC and MAPK	experimental (any)	2	0.226	0.049
Reactome	Regulation of RUNX1 Expression and Activity	experimental (any)	2	0.226	0.049
Reactome	Signaling by NODAL	experimental (any)	2	0.226	0.049
Reactome	CD28 dependent Vav1 pathway	experimental (strong)	2	0.206	0.050

Reactome	ERBB2 Activates PTK6 Signaling	experimental (strong)	2	0.206	0.050
Reactome	Interleukin-1 signaling	experimental (strong)	4	0.981	0.050
Reactome	MyD88 dependent cascade initiated on endosome	experimental (strong)	5	1.523	0.050
Reactome	Signaling by Activin	experimental (strong)	2	0.206	0.050
Reactome	Signaling by Receptor Tyrosine Kinases	experimental (strong)	11	5.42	0.050
Reactome	TRAF6 mediated induction of NFkB and MAP kinases upon TLR7/8 or 9 activation	experimental (strong)	5	1.497	0.050
Reactome	Toll Like Receptor 7/8 (TLR7/8) Cascade	experimental (strong)	5	1.523	0.050
Reactome	Toll Like Receptor 9 (TLR9) Cascade	experimental (strong)	5	1.523	0.050
Reactome	Transcriptional regulation by RUNX3	experimental (strong)	4	1.007	0.050

WikiPathways	Regulation of toll-like receptor signaling pathway	experimental (any)	17	1.594	4.99e-11
WikiPathways	Toll-like Receptor Signaling Pathway	experimental (any)	15	1.156	4.99e-11
WikiPathways	Hepatitis C and Hepatocellular Carcinoma	experimental (any)	11	0.651	2.27e-9
WikiPathways	Photodynamic therapy-induced NF-kB survival signaling	experimental (any)	9	0.452	1.84e-8
WikiPathways	Viral Acute Myocarditis	experimental (any)	12	1.036	1.84e-8
WikiPathways	Non-genomic actions of 1,25 dihydroxy vitamin D3	experimental (any)	11	0.877	3.06e-8
WikiPathways	Spinal Cord Injury	experimental (any)	13	1.369	3.06e-8
WikiPathways	LTF danger signal response pathway	experimental (any)	7	0.252	7.60e-8
WikiPathways	Fibrin Complement Receptor 3 Signaling Pathway	experimental (any)	8	0.438	2.06e-7

WikiPathways	miRNAs involvement in the immune response in sepsis	experimental (any)	8	0.465	3.07e-7
WikiPathways	Platelet-mediated interactions with vascular and circulating cells	experimental (any)	6	0.213	6.40e-7
WikiPathways	TLR4 Signaling and Tolerance	experimental (any)	7	0.345	6.40e-7
WikiPathways	VEGFA-VEGFR2 Signaling Pathway	experimental (any)	16	3.043	1.12e-6
WikiPathways	Hepatitis C and Hepatocellular Carcinoma	experimental (strong)	11	1.161	1.48e-6
WikiPathways	LTF danger signal response pathway	experimental (strong)	7	0.387	2.97e-6
WikiPathways	Pancreatic adenocarcinoma pathway	experimental (any)	10	1.156	3.15e-6
WikiPathways	Signal transduction through IL1R	experimental (any)	7	0.438	3.15e-6
WikiPathways	Apoptosis Modulation and Signaling	experimental (any)	10	1.169	3.30e-6

WikiPathways	Regulation of toll-like receptor signaling pathway	experimental (strong)	13	2.117	3.85e-6
WikiPathways	Viral Acute Myocarditis	experimental (strong)	11	1.394	3.85e-6
WikiPathways	Toll-like Receptor Signaling Pathway	experimental (strong)	12	1.833	4.96e-6
WikiPathways	Chromosomal and microsatellite instability in colorectal cancer	experimental (any)	9	0.957	5.50e-6
WikiPathways	Spinal Cord Injury	experimental (strong)	12	1.91	6.67e-6
WikiPathways	Hair Follicle Development: Cytodifferentiation (Part 3 of 3)	experimental (any)	9	1.01	7.87e-6
WikiPathways	socs1	experimental (any)	8	0.744	7.87e-6
WikiPathways	PDGF Pathway	experimental (any)	7	0.531	8.88e-6
WikiPathways	Photodynamic therapy-induced NF-kB survival signaling	experimental (strong)	8	0.749	1.01e-5

WikiPathways	Platelet-mediated interactions with vascular and circulating cells	experimental (strong)	6	0.361	1.47e-5
WikiPathways	Interactions between immune cells and microRNAs in tumor microenvironment	experimental (any)	6	0.372	1.51e-5
WikiPathways	Toll-like Receptor Signaling	experimental (any)	6	0.385	1.79e-5
WikiPathways	ApoE and miR-146 in inflammation and atherosclerosis	experimental (any)	4	0.106	1.85e-5
WikiPathways	Simplified Depiction of MYD88 Distinct Input-Output Pathway	experimental (any)	5	0.226	1.87e-5
WikiPathways	Apoptosis Modulation by HSP70	experimental (any)	5	0.239	2.46e-5
WikiPathways	Photodynamic therapy-induced AP-1 survival signaling.	experimental (any)	7	0.651	2.74e-5

WikiPathways	Structural Pathway of Interleukin 1 (IL-1)	experimental (any)	7	0.651	2.74e-5
WikiPathways	Integrated Breast Cancer Pathway	experimental (any)	11	1.966	3.39e-5
WikiPathways	Interleukin-1 Induced Activation of NF-kappa-B	experimental (any)	4	0.133	4.32e-5
WikiPathways	Allograft Rejection	experimental (any)	8	0.997	4.51e-5
WikiPathways	RANKL/RANK (Receptor activator of NFKB (ligand)) Signaling Pathway	experimental (any)	7	0.718	4.51e-5
WikiPathways	TGF-beta Receptor Signaling	experimental (any)	7	0.718	4.51e-5
WikiPathways	Simplified Depiction of MYD88 Distinct Input-Output Pathway	experimental (strong)	5	0.258	4.85e-5
WikiPathways	TGF-beta Signaling Pathway	experimental (any)	10	1.701	5.07e-5
WikiPathways	Allograft Rejection	experimental (strong)	8	0.981	6.68e-5
WikiPathways	EBV LMP1 signaling	experimental (any)	5	0.306	6.74e-5

WikiPathways	T-Cell antigen Receptor (TCR) Signaling Pathway	experimental (any)	8	1.076	7.10e-5
WikiPathways	DNA Damage Response (only ATM dependent)	experimental (any)	9	1.422	7.33e-5
WikiPathways	TNF related weak inducer of apoptosis (TWEAK) Signaling Pathway	experimental (any)	6	0.545	8.84e-5
WikiPathways	Fibrin Complement Receptor 3 Signaling Pathway	experimental (strong)	7	0.749	9.82e-5
WikiPathways	Envelope proteins and their potential roles in EDMD physiopathology	experimental (any)	6	0.558	9.93e-5
WikiPathways	AGE/RAGE pathway	experimental (any)	7	0.877	1.42e-4

WikiPathways	Mammary gland development pathway - Embryonic development (Stage 1 of 4)	experimental (any)	4	0.186	1.43e-4
WikiPathways	Interactions between immune cells and microRNAs in tumor microenvironment	experimental (strong)	6	0.542	1.52e-4
WikiPathways	Pancreatic adenocarcinoma pathway	experimental (strong)	10	1.858	1.52e-4
WikiPathways	Non-genomic actions of 1,25 dihydroxy vitamin D3	experimental (strong)	8	1.161	1.82e-4
WikiPathways	miRNAs involvement in the immune response in sepsis	experimental (strong)	7	0.852	1.82e-4
WikiPathways	Human Complement System	experimental (any)	7	0.930	1.99e-4

WikiPathways	Cells and Molecules involved in local acute inflammatory response	experimental (any)	4	0.213	2.43e-4
WikiPathways	Endoderm Differentiation	experimental (any)	9	1.741	3.08e-4
WikiPathways	Leptin signaling pathway	experimental (any)	7	1.01	3.16e-4
WikiPathways	ApoE and miR-146 in inflammation and atherosclerosis	experimental (strong)	4	0.206	3.18e-4
WikiPathways	TLR4 Signaling and Tolerance	experimental (strong)	6	0.645	3.25e-4
WikiPathways	Integrated Breast Cancer Pathway	experimental (strong)	11	2.555	3.39e-4
WikiPathways	Extracellular vesicles in the crosstalk of cardiac cells	experimental (any)	4	0.239	3.73e-4
WikiPathways	IL-1 signaling pathway	experimental (any)	6	0.731	3.93e-4
WikiPathways	VEGFA-VEGFR2 Signaling Pathway	experimental (strong)	14	4.156	4.10e-4

WikiPathways	Signal transduction through IL1R	experimental (strong)	6	0.697	4.44e-4
WikiPathways	BMP Signaling Pathway in Eyelid Development	experimental (any)	4	0.252	4.48e-4
WikiPathways	Chemokine signaling pathway	experimental (any)	9	1.953	6.62e-4
WikiPathways	Focal Adhesion	experimental (any)	10	2.405	6.62e-4
WikiPathways	Androgen receptor signaling pathway	experimental (any)	7	1.169	6.96e-4
WikiPathways	Association Between Physico-Chemical Features and Toxicity Associated Pathways	experimental (any)	6	0.824	6.96e-4
WikiPathways	IL1 and megakaryocytes in obesity	experimental (any)	4	0.292	7.13e-4
WikiPathways	The human immune response to tuberculosis	experimental (any)	4	0.292	7.13e-4
WikiPathways	Vitamin D in inflammatory diseases	experimental (any)	4	0.292	7.13e-4

WikiPathways	IL-2 Signaling Pathway	experimental (any)	5	0.545	7.56e-4
WikiPathways	Oncostatin M Signaling Pathway	experimental (any)	6	0.850	7.56e-4
WikiPathways	RAC1/PAK1/p38/MMP2 Pathway	experimental (any)	6	0.864	8.09e-4
WikiPathways	Vitamin B12 Metabolism	experimental (any)	5	0.571	9.11e-4
WikiPathways	Physiological and Pathological Hypertrophy of the Heart	experimental (any)	4	0.319	9.29e-4
WikiPathways	Apoptosis Modulation by HSP70	experimental (strong)	4	0.310	0.001
WikiPathways	EBV LMP1 signaling	experimental (strong)	5	0.542	0.001
WikiPathways	Hair Follicle Development: Cytodifferentiation (Part 3 of 3)	experimental (strong)	7	1.239	0.001
WikiPathways	PDGF Pathway	experimental (strong)	6	0.878	0.001
WikiPathways	Chromosomal and microsatellite instability in colorectal cancer	experimental (strong)	7	1.42	0.002

WikiPathways	Envelope proteins and their potential roles in EDMD physiopathology	experimental (strong)	5	0.645	0.002
WikiPathways	Human Complement System	experimental (strong)	5	0.645	0.002
WikiPathways	Interleukin-1 Induced Activation of NF-kappa-B	experimental (strong)	3	0.155	0.002
WikiPathways	Nonalcoholic fatty liver disease	experimental (strong)	8	1.833	0.002
WikiPathways	TNF related weak inducer of apoptosis (TWEAK) Signaling Pathway	experimental (strong)	6	0.955	0.002
WikiPathways	Toll-like Receptor Signaling	experimental (strong)	5	0.594	0.002
WikiPathways	Vitamin B12 Metabolism	experimental (strong)	5	0.645	0.002
WikiPathways	socs1	experimental (strong)	6	0.955	0.002
WikiPathways	Apoptosis	experimental (any)	6	1.063	0.002

WikiPathways	Control of immune tolerance by vasoactive intestinal peptide	experimental (any)	3	0.173	0.002
WikiPathways	Extracellular vesicle-mediated signaling in recipient cells	experimental (any)	4	0.372	0.002
WikiPathways	MicroRNAs in cardiomyocyte hypertrophy	experimental (any)	6	1.063	0.002
WikiPathways	Nonalcoholic fatty liver disease	experimental (any)	8	1.887	0.002
WikiPathways	PI3K-Akt Signaling Pathway	experimental (any)	12	3.867	0.002
WikiPathways	AGE/RAGE pathway	experimental (strong)	7	1.497	0.003
WikiPathways	Androgen receptor signaling pathway	experimental (strong)	7	1.445	0.003
WikiPathways	Cells and Molecules involved in local acute inflammatory response	experimental (strong)	3	0.181	0.003

WikiPathways	IL1 and megakaryocytes in obesity	experimental (strong)	4	0.413	0.003
WikiPathways	TGF-beta Signaling Pathway	experimental (strong)	9	2.478	0.003
WikiPathways	Alpha 6 Beta 4 signaling pathway	experimental (any)	4	0.438	0.003
WikiPathways	Breast cancer pathway	experimental (any)	8	1.966	0.003
WikiPathways	Corticotropin-releasing hormone signaling pathway	experimental (any)	6	1.143	0.003
WikiPathways	Ebola Virus Pathway on Host	experimental (any)	7	1.568	0.003
WikiPathways	Lung fibrosis	experimental (any)	5	0.744	0.003
WikiPathways	MAPK Signaling Pathway	experimental (any)	10	3.069	0.003
WikiPathways	Notch Signaling Pathway	experimental (any)	5	0.731	0.003
WikiPathways	T-Cell antigen Receptor (TCR) pathway during Staphylococcus aureus infection	experimental (any)	5	0.757	0.003
WikiPathways	TNF alpha Signaling Pathway	experimental (any)	6	1.156	0.003

WikiPathways	Amplification and Expansion of Oncogenic Pathways as Metastatic Traits	experimental (any)	3	0.226	0.004
WikiPathways	Canonical and Non-Canonical TGF-B signaling	experimental (any)	3	0.226	0.004
WikiPathways	Overview of nanoparticle effects	experimental (any)	3	0.239	0.004
WikiPathways	TGF-B Signaling in Thyroid Cells for Epithelial-Mesenchymal Transition	experimental (any)	3	0.226	0.004
WikiPathways	Leptin signaling pathway	experimental (strong)	7	1.652	0.005
WikiPathways	Photodynamic therapy-induced AP-1 survival signaling.	experimental (strong)	6	1.187	0.005
WikiPathways	DNA Damage Response	experimental (any)	5	0.864	0.005
WikiPathways	Nanomaterial induced apoptosis	experimental (any)	3	0.252	0.005

WikiPathways	Notch Signaling	experimental (any)	4	0.518	0.005
WikiPathways	miRNA Regulation of DNA Damage Response	experimental (any)	5	0.890	0.005
WikiPathways	VEGFA-VEGFR2 Signaling Pathway	predicted (union)	113	82.39	0.006
WikiPathways	Chemokine signaling pathway	experimental (strong)	8	2.168	0.006
WikiPathways	Control of immune tolerance by vasoactive intestinal peptide	experimental (strong)	3	0.232	0.006
WikiPathways	Vitamin D in inflammatory diseases	experimental (strong)	4	0.516	0.006
WikiPathways	Brain-Derived Neurotrophic Factor (BDNF) signaling pathway	experimental (any)	7	1.794	0.006
WikiPathways	EV release from cardiac cells and their functional effects	experimental (any)	2	0.080	0.006

WikiPathways	Fas Ligand (FasL) pathway and Stress induction of Heat Shock Proteins (HSP) regulation	experimental (any)	4	0.558	0.006
WikiPathways	IL-3 Signaling Pathway	experimental (any)	4	0.585	0.006
WikiPathways	IL-6 signaling pathway	experimental (any)	4	0.558	0.006
WikiPathways	Interleukin-11 Signaling Pathway	experimental (any)	4	0.585	0.006
WikiPathways	Primary Focal Segmental Glomerulosclerosis FSGS	experimental (any)	5	0.957	0.006
WikiPathways	Regulation of Actin Cytoskeleton	experimental (any)	7	1.807	0.006
WikiPathways	Senescence and Autophagy in Cancer	experimental (any)	6	1.382	0.006
WikiPathways	T-Cell antigen Receptor (TCR) Signaling Pathway	predicted (union)	51	32.41	0.007
WikiPathways	Prolactin Signaling Pathway	experimental (any)	5	0.983	0.007

WikiPathways	Thymic Stromal LymphoPoietin (TSLP) Signaling Pathway	experimental (any)	4	0.598	0.007
WikiPathways	Phosphodiesterases in neuronal function	predicted (intersection)	21	8.41	0.008
WikiPathways	Epithelial to mesenchymal transition in colorectal cancer	experimental (any)	7	1.966	0.008
WikiPathways	Hematopoietic Stem Cell Differentiation	experimental (any)	4	0.638	0.008
WikiPathways	DNA Damage Response (only ATM dependent)	experimental (strong)	8	2.375	0.009
WikiPathways	Endoderm Differentiation	experimental (strong)	7	1.858	0.009
WikiPathways	Cardiac Progenitor Differentiation	experimental (any)	4	0.664	0.009
WikiPathways	Cell Cycle	experimental (any)	6	1.528	0.009

WikiPathways	Selective expression of chemokine receptors during T-cell polarization	experimental (any)	3	0.332	0.009
WikiPathways	Signaling Pathways in Glioblastoma	experimental (any)	5	1.063	0.009
WikiPathways	Vitamin D Receptor Pathway	experimental (any)	7	2.046	0.009
WikiPathways	Apoptosis-related network due to altered Notch3 in ovarian cancer	experimental (any)	4	0.678	0.010
WikiPathways	EGF/EGFR Signaling Pathway	experimental (any)	7	2.086	0.010
WikiPathways	TFs Regulate miRNAs related to cardiac hypertrophy	experimental (any)	2	0.106	0.010
WikiPathways	VEGFA-VEGFR2 Signaling Pathway	predicted (intersection)	61	37.75	0.011
WikiPathways	Apoptosis Modulation and Signaling	experimental (strong)	6	1.445	0.011

WikiPathways	Notch Signaling Pathway	experimental (strong)	5	0.981	0.011
WikiPathways	Primary Focal Segmental Glomerulosclerosis FSGS	experimental (strong)	5	0.981	0.011
WikiPathways	IL-4 Signaling Pathway	experimental (any)	4	0.704	0.011
WikiPathways	Nanomaterial induced apoptosis	experimental (strong)	3	0.310	0.012
WikiPathways	RAC1/PAK1/p38/MMP2 Pathway	experimental (strong)	6	1.471	0.012
WikiPathways	Mammary gland development pathway - Pregnancy and lactation (Stage 3 of 4)	experimental (any)	3	0.372	0.012
WikiPathways	Pathways Regulating Hippo Signaling	experimental (any)	5	1.169	0.012
WikiPathways	Role Altered Glycolysation of MUC1 in Tumour Microenvironment	experimental (any)	2	0.120	0.012

WikiPathways	Extracellular vesicle-mediated signaling in recipient cells	experimental (strong)	4	0.645	0.013
WikiPathways	Inflammatory Response Pathway	experimental (any)	3	0.385	0.013
WikiPathways	Integrin-mediated Cell Adhesion	experimental (any)	5	1.196	0.013
WikiPathways	PDGFR-beta pathway	experimental (any)	3	0.385	0.013
WikiPathways	Ebola Virus Pathway on Host	experimental (strong)	6	1.523	0.014
WikiPathways	Mammary gland development pathway - Embryonic development (Stage 1 of 4)	experimental (strong)	3	0.336	0.014
WikiPathways	Notch Signaling	experimental (strong)	4	0.671	0.014
WikiPathways	Overview of nanoparticle effects	experimental (strong)	3	0.336	0.014
WikiPathways	T-Cell antigen Receptor (TCR) Signaling Pathway	experimental (strong)	6	1.574	0.014

WikiPathways	TGF-beta Receptor Signaling	experimental (strong)	5	1.084	0.014
WikiPathways	TNF alpha Signaling Pathway	experimental (strong)	6	1.574	0.014
WikiPathways	Gene regulatory network modelling somitogenesis	experimental (any)	2	0.133	0.014
WikiPathways	Alpha 6 Beta 4 signaling pathway	experimental (strong)	4	0.697	0.015
WikiPathways	Folate Metabolism	experimental (any)	4	0.784	0.015
WikiPathways	Resistin as a regulator of inflammation	experimental (any)	3	0.412	0.015
WikiPathways	RANKL/RANK (Receptor activator of NFKB (ligand)) Signaling Pathway	predicted (union)	33	19.52	0.016
WikiPathways	Association Between Physico-Chemical Features and Toxicity Associated Pathways	experimental (strong)	5	1.136	0.016

WikiPathways	PI3K-Akt Signaling Pathway	experimental (strong)	11	4.569	0.016
WikiPathways	Endometrial cancer	experimental (any)	4	0.811	0.016
WikiPathways	RANKL/RANK (Receptor activator of NFKB (ligand)) Signaling Pathway	experimental (strong)	5	1.161	0.017
WikiPathways	Melatonin metabolism and effects	experimental (any)	3	0.438	0.017
WikiPathways	miRNA regulation of prostate cancer signaling pathways	experimental (any)	3	0.438	0.017
WikiPathways	Extracellular vesicles in the crosstalk of cardiac cells	experimental (strong)	3	0.387	0.018
WikiPathways	Inflammatory Response Pathway	experimental (strong)	3	0.387	0.018
WikiPathways	IL-10 Anti-inflammatory Signaling Pathway	experimental (any)	2	0.159	0.018

WikiPathways	MAPK and NFkB Signalling Pathways Inhibited by Yersinia YopJ	experimental (any)	2	0.159	0.018
WikiPathways	Mesodermal Commitment Pathway	experimental (any)	6	1.834	0.018
WikiPathways	Non-small cell lung cancer	experimental (any)	4	0.850	0.018
WikiPathways	Pathways Affected in Adenoid Cystic Carcinoma	experimental (any)	4	0.837	0.018
WikiPathways	Wnt Signaling Pathway and Pluripotency	experimental (any)	5	1.315	0.018
WikiPathways	Neural Crest Cell Migration during Development	experimental (any)	3	0.465	0.019
WikiPathways	Type II interferon signaling (IFNG)	experimental (any)	3	0.465	0.019

WikiPathways	BMP Signaling Pathway in Eyelid Development	experimental (strong)	3	0.413	0.020
WikiPathways	Canonical and Non-Canonical TGF-B signaling	experimental (strong)	3	0.413	0.020
WikiPathways	Fas Ligand (FasL) pathway and Stress induction of Heat Shock Proteins (HSP) regulation	experimental (strong)	4	0.774	0.020
WikiPathways	Focal Adhesion	experimental (strong)	8	2.891	0.020
WikiPathways	Folate Metabolism	experimental (strong)	4	0.800	0.020
WikiPathways	Hedgehog Signaling Pathway	experimental (strong)	3	0.413	0.020
WikiPathways	Selective expression of chemokine receptors during T-cell polarization	experimental (strong)	3	0.413	0.020
WikiPathways	Selenium Micronutrient Network	experimental (strong)	4	0.800	0.020

WikiPathways	TGF-B Signaling in Thyroid Cells for Epithelial-Mesenchymal Transition	experimental (strong)	3	0.413	0.020
WikiPathways	G13 Signaling Pathway	experimental (any)	3	0.478	0.020
WikiPathways	Photodynamic therapy-induced HIF-1 survival signaling	experimental (any)	3	0.478	0.020
WikiPathways	Osteopontin Signaling	experimental (any)	2	0.173	0.021
WikiPathways	Selenium Micronutrient Network	experimental (any)	4	0.904	0.021
WikiPathways	Cell Cycle	experimental (strong)	6	1.858	0.023
WikiPathways	EV release from cardiac cells and their functional effects	experimental (strong)	2	0.155	0.023
WikiPathways	Eicosanoid Synthesis	experimental (strong)	2	0.155	0.023
WikiPathways	IL-6 signaling pathway	experimental (strong)	4	0.852	0.023

WikiPathways	Physiological and Pathological Hypertrophy of the Heart	experimental (strong)	3	0.439	0.023
WikiPathways	TFs Regulate miRNAs related to cardiac hypertrophy	experimental (strong)	2	0.155	0.023
WikiPathways	Thymic Stromal LymphoPoietin (TSLP) Signaling Pathway	experimental (strong)	4	0.852	0.023
WikiPathways	Bladder Cancer	experimental (any)	3	0.518	0.023
WikiPathways	ERK Pathway in Huntington's Disease	experimental (any)	2	0.186	0.023
WikiPathways	H19 action Rb-E2F1 signaling and CDK-Beta-catenin activity	experimental (any)	2	0.186	0.023
WikiPathways	IL-5 Signaling Pathway	experimental (any)	3	0.518	0.023
WikiPathways	Neural Crest Cell Migration in Cancer	experimental (any)	3	0.505	0.023

WikiPathways	ncRNAs involved in STAT3 signaling in hepatocellular carcinoma	experimental (any)	2	0.186	0.023
WikiPathways	Corticotropin-releasing hormone signaling pathway	experimental (strong)	5	1.342	0.024
WikiPathways	Cardiac Progenitor Differentiation	experimental (strong)	4	0.878	0.025
WikiPathways	Wnt Signaling	experimental (any)	5	1.475	0.025
WikiPathways	Regulation of Actin Cytoskeleton	experimental (strong)	6	1.91	0.026
WikiPathways	Melatonin metabolism and effects	experimental (strong)	3	0.490	0.027
WikiPathways	Gene regulatory network modelling somitogenesis	experimental (strong)	2	0.181	0.028
WikiPathways	Pathways Regulating Hippo Signaling	experimental (strong)	5	1.42	0.028
WikiPathways	Hedgehog Signaling Pathway	experimental (any)	3	0.558	0.028

WikiPathways	Structural Pathway of Interleukin 1 (IL-1)	experimental (strong)	4	0.929	0.029
WikiPathways	Integrated Cancer Pathway	experimental (any)	3	0.571	0.029
WikiPathways	Role of Osx and miRNAs in tooth development	experimental (any)	2	0.213	0.029
WikiPathways	Androgen receptor signaling pathway	predicted (union)	47	31.24	0.030
WikiPathways	MECP2 and Associated Rett Syndrome	predicted (union)	35	21.87	0.030
WikiPathways	G13 Signaling Pathway	experimental (strong)	3	0.516	0.030
WikiPathways	Aryl Hydrocarbon Receptor	experimental (any)	3	0.585	0.031
WikiPathways	Adipogenesis	experimental (any)	5	1.581	0.032
WikiPathways	Alzheimers Disease	experimental (strong)	4	0.981	0.034
WikiPathways	Pathogenic Escherichia coli infection	experimental (strong)	3	0.542	0.034
WikiPathways	Alzheimers Disease	experimental (any)	4	1.076	0.034

WikiPathways	Eicosanoid Synthesis	experimental (any)	2	0.239	0.034
WikiPathways	Regulation of Microtubule Cytoskeleton	experimental (any)	3	0.611	0.034
WikiPathways	Differentiation Pathway	experimental (any)	3	0.624	0.035
WikiPathways	ncRNAs involved in Wnt signaling in hepatocellular carcinoma	experimental (any)	4	1.09	0.035
WikiPathways	Oncostatin M Signaling Pathway	experimental (strong)	5	1.523	0.036
WikiPathways	Epithelial to mesenchymal transition in colorectal cancer	experimental (strong)	7	2.762	0.039
WikiPathways	Lung fibrosis	experimental (strong)	4	1.032	0.039
WikiPathways	Wnt Signaling Pathway	experimental (any)	3	0.651	0.039
WikiPathways	Pathogenic Escherichia coli infection	experimental (any)	3	0.664	0.041
WikiPathways	Retinoblastoma Gene in Cancer	experimental (any)	4	1.156	0.041

WikiPathways	IL-10 Anti-inflammatory Signaling Pathway	experimental (strong)	2	0.232	0.043
WikiPathways	Role Altered Glycolysation of MUC1 in Tumour Microenvironment	experimental (strong)	2	0.232	0.043
WikiPathways	Cancer immunotherapy by PD-1 blockade	experimental (any)	2	0.279	0.043
WikiPathways	Canonical and Non-canonical Notch signaling	experimental (any)	2	0.279	0.043
WikiPathways	Cardiac Hypertrophic Response	experimental (any)	3	0.678	0.043
WikiPathways	ErbB Signaling Pathway	experimental (any)	4	1.183	0.043
WikiPathways	Myometrial Relaxation and Contraction Pathways	experimental (any)	5	1.754	0.043
WikiPathways	Neovascularisation processes	experimental (any)	2	0.279	0.043
WikiPathways	Ciliary landscape	experimental (strong)	4	1.084	0.044

WikiPathways	MAPK Signaling Pathway	experimental (strong)	8	3.51	0.044
WikiPathways	Mammary gland development pathway - Pregnancy and lactation (Stage 3 of 4)	experimental (strong)	3	0.619	0.044
WikiPathways	Resistin as a regulator of inflammation	experimental (strong)	3	0.619	0.044
WikiPathways	Breast cancer pathway	experimental (strong)	7	2.891	0.045
WikiPathways	IL-1 signaling pathway	experimental (strong)	4	1.11	0.045
WikiPathways	DNA Damage Response	experimental (strong)	4	1.136	0.048
WikiPathways	DNA IR-Double Strand Breaks (DSBs) and cellular response via ATM	experimental (any)	3	0.731	0.050
WikiPathways	Estrogen signaling pathway	experimental (any)	2	0.306	0.050

Supplementary Table 1b

Supplementary Table 1: A) Pathways influenced by miR148-5p according to miRPathDB; B) Pathways influenced by miR146-5p according to miRPathDB.

gene_id	A2780M1	A2780M2	A2780M3	A2780C1	A2780C2	A2780C3	A2780M	A2780C
ENSG00000106366	20.03857093	9.785884864	20.99019155	188.2531575	111.05448	128.7540793	16.93821578	142.6872389
ENSG00000125740	52.10028442	18.48444919	34.63381606	217.3644705	227.1568909	204.9764942	35.07284989	216.4992852
ENSG00000269466	19.03664238	3.261961621	3.148528733	95.09695585	15.14379272	46.35146855	8.482377579	52.19740571
ENSG00000148677	38.07328477	31.53229567	40.93087352	116.4452521	222.10896	237.9375385	36.84548466	192.1639169
ENSG00000163545	25.04821366	42.40550108	44.07940226	155.2603361	198.5519491	202.916429	37.17770567	185.576238
ENSG00000184216	1349.597752	1594.011912	1183.846803	5234.21408	4718.974077	4669.137932	1375.818823	4874.108696
ENSG00000126768	349.6730627	339.2440086	504.8141068	1205.208359	1358.734736	1623.331432	397.9103927	1395.758176
ENSG00000073756	186.3587097	223.9880313	282.3180764	827.7316667	829.5433126	694.2419956	230.8882724	783.8389916
ENSG00000126903	348.6711342	384.9114713	362.0808043	1038.303498	1104.655547	1370.973436	365.2211366	1171.310827
ENSG00000128564	1372.642109	1675.560953	1260.461003	4888.759832	4042.551336	4726.819759	1436.221355	4552.710309
ENSG00000183092	13.0250711	21.74641081	14.69313409	75.68941384	42.90741272	32.9610443	16.48820533	50.51929029
ENSG00000178878	11.02121401	26.09569297	13.64362451	52.40036343	42.90741272	55.62176226	16.92017716	50.30984613
ENSG00000139572	35.06749913	27.18301351	14.69313409	127.1194002	51.3206309	50.47159908	25.64788224	76.30387672
ENSG00000143257	14.02699965	26.09569297	18.8911724	58.22262603	53.00327454	55.62176226	19.67128834	55.61588761
ENSG00000177679	44.08485605	40.23086	30.43577775	131.0009086	70.67103271	111.2435245	38.25049793	104.3051553
ENSG00000196747	16.03085674	30.44497513	27.28724902	98.00808715	35.33551636	66.95212123	24.58769363	66.76524158
ENSG00000174827	133.2564967	102.2081308	117.5450727	370.6840524	268.38166	320.3401493	117.6699001	319.8019539
ENSG00000198959	36.06942767	18.48444919	20.99019155	93.15620165	43.74873454	63.86202333	25.18135614	66.92231984
ENSG00000135097	17.03278529	33.70693675	18.8911724	68.89677414	47.95534363	65.9220886	23.21029815	60.92473545
ENSG00000100065	87.16778355	57.62798864	91.30733325	160.1122216	211.1717763	242.0576691	78.70103515	204.4472223
ENSG00000111305	76.14656953	109.8193746	74.51518001	257.1499316	193.5040181	220.4269838	86.82704138	223.6936445
ENSG00000144867	547.0529864	700.2344281	709.4684744	1590.448068	1668.341165	1773.716196	652.251963	1677.50181

ENSG00000126391	516.99513	576.2798864	504.8141068	1564.247886	1304.89014	1238.099226	532.6963744	1369.079084
ENSG00000153832	79.15235517	41.31818054	27.28724902	115.474875	112.7371236	147.2946667	49.25259491	125.1688884
ENSG00000213123	23.04435657	31.53229567	17.84166282	60.16338023	53.84459635	67.98215387	24.13943835	60.66337682
ENSG00000272405	29.05592785	39.14353946	25.18822986	111.5933666	67.30574544	55.62176226	31.12923239	78.17362475
ENSG00000210195	57.10992715	119.6052594	116.4955631	226.0978644	211.1717763	298.709464	97.73691657	245.3263682
ENSG00000128342	160.3085674	101.1208103	55.62400761	352.2468875	190.9800527	246.1777996	105.6844618	263.1349133
ENSG00000108932	54.10414151	23.92105189	29.38626817	102.8599727	81.60821635	80.34254548	35.80382052	88.27024483
ENSG00000166881	1028.980617	936.1829853	1019.0738	2612.255155	2484.423329	2212.510099	994.7458008	2436.396194
ENSG00000124641	244.4705653	271.8301351	255.0308273	688.9677414	567.8922272	630.3799722	257.1105093	629.0799802
ENSG00000167264	126.2429969	289.2272638	207.8028964	469.6625166	468.6162526	575.7882426	207.757719	504.689004
ENSG00000119878	116.2237114	134.827747	116.4955631	259.0906858	319.7022909	312.0998882	122.5156738	296.9642883
ENSG00000159388	533.0259867	346.8552524	449.1900992	1588.507314	799.2557271	831.2363359	443.0237794	1072.999792
ENSG00000179388	130.250711	46.75478324	59.82204592	193.105043	192.6626963	187.4659395	78.9425134	191.0778929
ENSG00000151790	84.16199791	60.88995027	48.27744057	120.3267605	187.6147654	155.5349278	64.44312958	154.4921512
ENSG00000172331	144.2777107	101.1208103	139.5847738	270.735211	333.1634399	318.280084	128.3277649	307.3929117

ENSG00000205583	114.2198543	67.41387351	44.07940226	262.0018171	70.67103271	203.9464616	75.23771002	178.8731038
ENSG00000186280	50.09642733	40.23086	31.48528733	128.0897773	79.08425089	81.37257811	40.60419155	96.18220209
ENSG00000101605	38.07328477	53.27870648	46.17842141	94.12657875	106.8478709	112.2735571	45.84347089	104.4160023
ENSG00000137801	50.09642733	36.96889838	37.78234479	117.4156292	94.22804362	73.13231704	41.61589016	94.92532994
ENSG00000171246	41.07907041	41.31818054	24.13872028	97.03771005	72.35367635	73.13231704	35.51199041	80.84123448
ENSG00000148943	624.2014845	538.2236675	421.9028502	1281.86815	1304.048818	1017.672243	528.1093341	1201.196403
ENSG00000169093	382.7367048	449.0633832	420.8533406	879.1651631	826.1780253	1115.525343	417.5511429	940.2883405

ENSG00000073969	551.0607006	578.4545275	544.6954708	1324.564742	1198.883591	1244.279422	558.070233	1255.909252
ENSG00000147509	170.3278529	157.6614784	118.5945823	338.6616081	301.1932109	365.6615852	148.8613045	335.1721347
ENSG00000086506	128.246854	148.962914	121.743111	250.3572919	292.7799927	354.3312262	132.984293	299.1561703
ENSG00000168917	180.3471384	216.3767876	156.3769271	412.4102677	414.7716563	415.1031516	184.366951	414.0950252
ENSG00000142871	1328.557253	1412.429382	1397.946757	2967.413173	3240.771643	3089.06787	1379.644464	3099.084229
ENSG00000197903	857.6508358	725.2428005	665.3890722	2061.080961	1536.25364	1454.40608	749.4275695	1683.91356
ENSG00000006327	851.6392645	853.5466243	661.1910339	1867.005541	1645.625476	1742.815217	788.7923075	1751.815412
ENSG00000064199	39.07521331	56.5406681	104.9509578	163.0233529	132.0875254	148.3246993	66.85561306	147.8118592
ENSG00000144455	193.3722095	223.9880313	235.0901454	491.0108129	444.2179199	489.2655013	217.4834621	474.8314114
ENSG00000177721	128.246854	155.4868373	99.70340987	318.283689	237.2527527	281.1989092	127.812367	278.9117836
ENSG00000059804	1068.055831	1263.466468	1305.589914	2724.818898	2606.414992	2589.502043	1212.370738	2640.245311
ENSG00000135698	354.6827055	353.3791756	342.1401223	653.0637886	748.776418	883.7680003	350.0673345	761.8694023
ENSG00000128045	32.06171349	31.53229567	30.43577775	63.07451153	67.30574544	74.16234967	31.3432623	68.18086888
ENSG00000124171	360.6942767	214.2021465	221.4465209	612.3079504	577.1467672	542.8271983	265.447648	577.4273053
ENSG00000078967	103.1986403	164.1854016	172.1195707	298.876147	300.351889	351.2411283	146.5012042	316.8230548
ENSG00000166670	4859.353451	4741.804877	4647.228409	10154.99636	9572.559646	10960.57726	4749.462246	10229.37775
ENSG00000139921	1054.028831	801.3552383	797.6272789	1658.374465	2003.187249	2007.533604	884.3371161	1889.698439
ENSG00000143333	633.2188414	653.4796448	669.5871105	1465.269422	1224.123245	1465.736439	652.0951989	1385.043035
ENSG00000137331	514.9912729	379.4748686	431.3484364	1231.408541	721.0127981	859.0472171	441.9381926	937.1561852
ENSG00000082153	4125.941755	3054.283398	3295.460074	7072.108308	7693.046704	7259.670007	3491.895075	7341.60834
ENSG00000275064	85.16392645	90.24760486	101.802429	150.4084506	199.3932709	229.6972775	92.40465344	193.166333
ENSG00000124380	273.5264932	257.6949681	270.773471	520.1221259	577.1467672	563.427851	267.3316441	553.5655813
ENSG00000087085	73.1407839	65.23923243	71.36665127	137.7935483	138.8181	157.5949931	69.91555587	144.7355471

ENSG00000171174	48.09257023	54.36602702	55.62400761	103.8303498	115.2610891	106.0933613	52.69420162	108.3949334
ENSG00000237036	45.08678459	84.81100216	62.97057465	157.2010903	116.1024109	122.5738835	64.2894538	131.9591282
ENSG00000120885	1030.984474	1283.038238	1360.164413	2162.00018	2587.905912	2717.226089	1224.729042	2489.044061
ENSG00000116675	40.07714186	43.49282162	54.57449803	115.474875	89.18011271	75.19238231	46.04815384	93.28245666
ENSG00000137720	244.4705653	213.1148259	225.6445592	424.0547929	414.7716563	534.5869372	227.7433168	457.8044621
ENSG00000170345	102.1967117	150.0502346	187.8622144	196.9865514	307.9237854	378.0219768	146.7030536	294.3107712

log2Fold change	pvalue	padj	gene_name	gene_chr	gene_start	gene_end	gene_strand	gene_lenght
-3.0687294	1.38E- 14	4.95E-12	SERPINE1	7	101127089	101139266	+	3190
-2.6199490	6.39E- 16	3.06E-13	FOSB	19	45467995	45475179	+	5553
-2.6091492	0.000532753	0.009513864	H3.Y	5	17654870	17655847		978
-2.3820226	1.53E- 12	4.31E-10	ANKRD1	10	-90912096	90921276	-	1979
-2.3229827	1.05E- 13	3.32E-11	NUAK2	1	205302059	205321791	-	3441
-1.8250487	6.5E- 50	4.21E-46	IRAK1	X	154010500	154019980		4001
-1.8103241	5.74E- 26	8.26E-23	TIMM17B	X	-48893447	48898143	-	2562
-1.7644604	5.38E- 23	5.8E-20	PTGS2	1	186671791	186680427	-	5269
-1.6811286	3.08E- 29	6.65E-26	SLC10A3	X	154487306	154490690	-	2247
-1.6645273	1.09E- 38	4.7E-35	VGF	7	101162509	101165593	-	2704
-1.6182851	0.002378216	0.028749541	BEGAIN	14	100537147	100587413		7878
-1.5756383	0.002203826	0.027278138	APOLD1	12	-12725917	12829975	+	7243
-1.5705228	0.001474358	0.020029914	GPR84	12	54362445	54364487	-	2043
-1.5026547	0.001316283	0.018564175	NR1I3	1	161229666	161238302		3305
-1.4442954	0.000103115	0.002730455	SRRM3	7	-76201900	76287288	+	6873
-1.4410503	0.003198062	0.035602156	HIST1H2AI	6	27808199	27808701	+	503
-1.4402894	1.1E- 10	2.13E-08	PDZK1	1	145670852	145708148		2480
-1.4046128	0.002703522	0.03173391	TGM2	20	-38127387	38166578	-	8036
-1.3940773	0.002509412	0.030054909	MSI1	12	120341330	120369180		3090
-1.3754498	0.000000879	0.0000552	CARD10	22	-37490362	37519542		5652
-1.3661769	0.000000139	0.0000109	GSG1	12	-13083560	13103683	-	3005
-1.3632873	4.66E- 24	6.03E-21	SRPRB	3	133784033	133825772	+	4180

-1.3620884	1.05E- 22	1.04E-19	FRMD8	11	65386599	65413525	+	4091
-1.3415552	0.000386745	0.007540949	FBXO36	2	229922302	230013109	+	3945
-1.3298308	0.002600694	0.030778054	TCTEX1D2	3	196291219	196318299	-	1246
-1.3297772	0.001663581	0.021955342	AL365181.3	1	156641666	156644887	-	3222
-1.3288589	0.00000389	0.000193157	MT-TT	MT	15888	15953	+	66
-1.3137714	0.0000607	0.001809691	LIF	22	30240447	30246851		3969
-1.2976076 -	0.001174506	0.017104982	SLC16A6	17	-68267026	68291267		4061
1.2923317	7.19E- 29	1.33E-25	NEMP1	12	-57055643	57088063		5708
-1.2907173	1.4E- 15	6.46E-13	MED20	6	-41905354	41921139		3278
-1.2818565	0.000000114	0.00000918	DUS2	16	-67987746	68079320	+	4472
-1.2783763	2.5E-09	0.000000356	CRIPT	2	46616416	46625742	+	2561
-1.2759139	0.000620261	0.010650553	BTG2	1	203305491	203309602	+	2757
-1.2720023	0.000132265	0.003305853	EGR3	8	22687659	22693302	-	4855
-1.2610894	0.0000546 -	0.001704637	TDO2	4	155854738	155920406	+	5264
1.2593021	1.44E-08	0.00000168	BPGM	7	13464680 8	134679813	+	2539
-1.246038584	0.002691097	0.031616727	STAG3L1	7	75359194	75395383	+	2094
-1.2421171	0.00087708	0.013984667	KDM4D	11	94973681	94999518	+	2978
-1.1892037	0.000330334	0.006756454	MYOM1	18	3066807	3220108	-	6574
-1.1888078	0.000994152	0.01521429	THBS1	15	39581079	39599466	+	9158
-1.1861457	0.002337216	0.028505108	NPTX1	17	80467148	80477843		6703
-1.1856302	4.4E- 13	1.3E-10	LIN7C	11	-27494576	27506773	-	4688
-1.1707717	1.03E- 13	3.32E-11	ASMTL	X	1403139	1453762	-	3506

-1.1701657	6.07E- 21	5.24E-18	NSF	17	46590669	46757464	+	5005
-1.1696220	0.000000038	0.00000373	RGS20	8	53851808	53959303	+	3192
-1.1695742	0.000000138	0.0000109	HBQ1	16	180453	181181	+	536
-1.1681012	6.87E-10 -	0.000000111	SLC35G2	3	136818647	136855892	+	4287
1.1677902	1.1E- 29	2.86E-26	CYR61	1	85580761	85583962	+	2455
-1.1676100	5.43E- 14	1.85E-11	HIST1H2BK	6	27146418	27146798	-	381
-1.1509348	7.67E- 18	4.97E-15	TNFRSF12A	16	3018445	3022383	+	1848
-1.1452016	0.000919851	0.014453051	SPA17	11	124673798	124697518	+	5205
-1.1266174	9.78E- 11	1.95E-08	SUMF1	3	3700814	4467281	-	6952
-1.1255765	0.00000192	0.000107088	ANXA2R	5	43039081	43043170	-	2386
-1.1231241	2.09E- 22	1.94E-19	SLC2A3	12	7919230	7936275	-	6159
-1.1214660	1.8E- 12	4.95E-10	MPHOSPH6	16	82147798	82170226		2190
-1.1206196	0.004149671	0.042772286	RASL11B	4	-52862290	52866835	+	2472
-1.1198856	3.62E-08	0.00000363	PARD6B	20	50731544	50756795	+	4726
-1.1137317 -	0.00000141	0.000082	UBE2D4	7	43926438	43956136	+	4713
1.1067726	2.13E- 31	6.88E-28	MMP10	11	102770503	102780628		1758
-1.0950977	1.34E- 14	4.95E-12	TMX1	14	-51240162	51257546	+	4499
-1.0863051	3.76E- 17	2.12E-14	RGS16	1	182598623	182604408		2427
-1.0834327	1.94E-08 -	0.00000213	IER3	6	-30743199	30744554	-	1356
1.0719527	3.29E- 19	2.37E-16	BZW1	2	200810594	200827338	+	8085
-1.063717742	0.0000503	0.001611034	AC239860.1	1	145509047	145520594	+	1270
-1.0501227	3.44E- 11	7.19E-09	SNRNP27	2	69893560	69905575	+	2275
-1.0484821	0.000136312	0.003374435	ACHE	7	100889994	100896974	-	3783

-1.0418827	0.000845113	0.013660029	RBKS	2	27781364	27891098		3585
-1.0393409	0.001199187	0.017375928	ZEB1-AS1	10	-31206278	31320447		3831
-1.0233963	4.56E-14	1.59E-11	CLU	8	-27596917	27615031		6616
-1.0192638	0.003889384	0.040873262	DNAJC6	1	-65248219	65415869	+	6954
-1.0058072	3.67E-08	0.00000366	C11orf1	11	111878935	111885975	+	3831
-1.0049928	0.000272626	0.005873025	FOS	14	75278774	75282230	+	3238

gene_biotype	tf_family	A2780M1_count	A2780M2_count	A2780M3_count	A2780C1_count	A2780C2_count	A2780C3_count	A2780M1_fpkm
protein_coding	-	20	9	20	194	132	125	0.308173016
protein_coding	TF_bZIP	52	17	33	224	270	199	0.460289393
protein_coding	-	19	3	3	98	18	45	0.954926714
protein_coding	-	38	29	39	120	264	231	0.943828526
protein_coding	-	25	39	42	160	236	197	0.357117089
protein_coding	-	1347	1466	1128	5394	5609	4533	16.54833641
protein_coding	-	349	312	481	1242	1615	1576	6.695786511
protein_coding	-	186	206	269	853	986	674	1.735162057
protein_coding	-	348	354	345	1070	1313	1331	7.612572962
protein_coding	-	1370	1541	1201	5038	4805	4589	24.90400395
protein_coding	-	13	20	14	78	51	32	0.081111545
protein_coding	-	11	24	13	54	51	54	0.074649946
protein_coding	-	35	25	14	131	61	49	0.842083144
protein_coding	THR-like	14	24	18	60	63	54	0.208214931
protein_coding	-	44	37	29	135	84	108	0.314674557
protein_coding	-	16	28	26	101	42	65	1.563533872
protein_coding	-	133	94	112	382	319	311	2.636059791
protein_coding	-	36	17	20	96	52	62	0.220200281
protein_coding	-	17	31	18	71	57	64	0.270424315
protein_coding	-	87	53	87	165	251	235	0.756610556
protein_coding	-	76	101	71	265	230	214	1.243152514
protein_coding	-	546	644	676	1639	1983	1722	6.420541501

protein_coding	-	516	530	481	1612	1551	1202	6.19976915
protein_coding	-	79	38	26	119	134	143	0.984317894
protein_coding	-	23	29	17	62	64	66	0.907329623
antisense	-	29	36	24	115	80	54	0.442412876
Mt_tRNA	-	57	110	111	233	251	290	42.450833
protein_coding	-	160	93	53	363	227	239	1.981500473
protein_coding	-	54	22	28	106	97	78	0.653606055
protein_coding	-	1027	861	971	2692	2953	2148	8.843858305
protein_coding	-	244	250	243	710	675	612	3.658778966
protein_coding	-	126	266	198	484	557	559	1.384917958
protein_coding	-	116	124	111	267	380	303	2.226402635
protein_coding	-	532	319	428	1637	950	807	9.484843355
protein_coding	zf-C2H2	130	43	57	199	229	182	1.316162203
protein_coding	-	84	56	46	124	223	151	0.784365895
protein_coding	-	144	93	133	279	396	309	2.787758109
transcribed_unprocessed_pseudogene	-	114	62	42	270	84	198	2.675983742
protein_coding	-	50	37	30	132	94	79	0.825278645
protein_coding	-	38	49	44	97	127	109	0.284124833
protein_coding	-	50	34	36	121	112	71	0.26836425
protein_coding	-	41	38	23	100	86	71	0.300656041
protein_coding	-	623	495	402	1321	1550	988	6.532143852
protein_coding	-	382	413	401	906	982	1083	5.355582919

protein_coding	-	550	532	519	1365	1425	1208	5.401494078
protein_coding	-	170	145	113	349	358	355	2.617829367
protein_coding	-	128	137	116	258	348	344	11.73817221
protein_coding	-	180	199	149	425	493	403	2.063831887
protein_coding	-	1326	1299	1332	3058	3852	2999	26.54894845
protein_coding	-	856	667	634	2124	1826	1412	110.4343262
protein_coding	-	850	785	630	1924	1956	1692	22.60852635
protein_coding	-	39	52	100	168	157	144	0.368297838
protein_coding	-	193	206	224	506	528	475	1.364592067
protein_coding	-	128	143	95	328	282	273	2.636907084
protein_coding	-	1066	1162	1244	2808	3098	2514	8.507506649
protein_coding	-	354	325	326	673	890	858	7.945375809
protein_coding	-	32	29	29	65	80	72	0.636292506
protein_coding	-	360	197	211	631	686	527	3.744243462
protein_coding	-	103	151	164	308	357	341	1.07422457
protein_coding	-	4850	4361	4428	10465	11378	10641	135.6057686
protein_coding	-	1052	737	760	1709	2381	1949	11.4935726
protein_coding	-	632	601	638	1510	1455	1423	12.79978275
protein_coding	-	514	349	411	1269	857	834	18.63196785
protein_coding	-	4118	2809	3140	7288	9144	7048	25.03580814
unprocessed_pseudogene	-	85	83	97	155	237	223	3.289807614
protein_coding	-	273	237	258	536	686	547	5.898431533
protein_coding	-	73	60	68	142	165	153	0.948509785

protein_coding	-	48	50	53	107	137	103	0.658123463
antisense	-	45	78	60	162	138	119	0.57737192
protein_coding	-	1029	1180	1296	2228	3076	2638	7.64495925
protein_coding	-	40	40	52	119	106	73	0.282735669
protein_coding	-	244	196	215	437	493	519	3.130638854
protein_coding	TF_bZIP	102	138	179	203	366	367	1.548383818

A2780M2 _fpkm	A2780M3 _fpkm	A2780C1_fpkm	A2780C2_ fpkm	A2780C3_ fpkm
0.149157658	0.320665963	2.894508081	1.687023193	1.962168056
0.161850847	0.30394837	1.919924118	1.982320607	1.794495089
0.162172096	0.156890249	4.769259356	0.750363076	2.304042736
0.774722078	1.007934626	2.886018903	5.438710444	5.844980369
0.599202564	0.624278201	2.213089764	2.796180066	2.866806792
19.37131698	14.41962946	64.16616891	57.15506114	56.73277656
6.438270174	9.602393592	23.07311283	25.69988656	30.80306689
2.066963229	2.611184949	7.705211286	7.629332127	6.405434125
8.329020258	7.852891101	22.66441035	23.823165	29.66141425
30.12934429	22.71694217	88.67785823	72.44768454	84.98225319
0.134217068	0.09089199	0.471240215	0.263931973	0.203399965
0.175180792	0.091799099	0.354845248	0.287071115	0.37332936
0.646942036	0.350488055	3.051874346	1.217304629	1.201004362
0.383913608	0.278557332	0.864059215	0.777153649	0.818161741
0.284609314	0.21580684	0.934869829	0.498276892	0.786854228
2.942956041	2.643741055	9.556901463	3.404231691	6.470863562
2.003871652	2.309829343	7.331205639	5.244170549	6.279507441
0.111841433	0.127292736	0.568584511	0.263816054	0.386339072
0.530391973	0.297939152	1.09361281	0.752062928	1.037142344
0.495755391	0.787282598	1.389458278	1.810545208	2.082008893
1.776931254	1.20844649	4.197252699	3.120478285	3.566039654
8.145229291	8.271494142	18.66235263	19.34123659	20.62878913

6.849204272	6.013525393	18.75423171	15.45681907	14.71268238
0.509249156	0.337085361	1.435699911	1.384827234	1.815122336
1.230477521	0.697821637	2.368303554	2.094111186	2.652422873
0.590705063	0.380977439	1.698775636	1.012283728	0.839237913
88.11350527	86.0186447	168.0256967	155.0485078	220.0244447
1.238783974	0.68298053	4.353005798	2.331753103	3.01532184
0.286406863	0.352645701	1.24232892	0.973815202	0.961786074
7.974673585	8.700592284	22.44681931	21.09197693	18.84374349
4.032039594	3.791498397	10.3089188	8.395230577	9.348874808
3.144659088	2.264524104	5.151186646	5.077982564	6.259316099
2.559798825	2.216802245	4.962098744	6.04939333	5.924475683
6.117129093	7.940000965	28.26022614	14.04832818	14.65728862
0.468244786	0.600480866	1.950866547	1.923021116	1.877150204
0.562426629	0.446946462	1.12116498	1.727136998	1.436408406
1.936484283	2.679183701	5.230042546	6.358728615	6.094143126

1.565340208	1.025855439	6.136924866	1.635461834	4.734840831
0.65685689	0.515240643	2.109662374	1.286888634	1.328370643
0.394057964	0.342323354	0.702272648	0.787611097	0.830259148
0.196278173	0.201055248	0.628852006	0.498603346	0.388217028
0.299714743	0.175497999	0.710059104	0.523078701	0.530403035
5.582276251	4.385832106	13.4115488	13.47975194	10.55324967
6.227760013	5.849867282	12.29928897	11.41924474	15.46797338

5.61954663	5.303674084	12.98052593	11.60776447	12.08592024
2.401589899	1.810627504	5.203867903	4.572544587	5.569065702
13.51292935	11.06895831	22.90965957	26.46989748	32.13738415
2.454106025	1.777650328	4.718447921	4.688471072	4.707262679
27.97379471	27.75021043	59.28566615	63.96944562	61.17047326
92.55387089	85.1094599	265.3342151	195.3950703	185.5780437
22.45750053	17.43621176	49.55264263	43.15237355	45.84745818
0.528173173	0.982636334	1.536215941	1.229749695	1.385347194
1.566574979	1.647979508	3.464212089	3.096434974	3.421375313
3.168540969	2.036417021	6.54285241	4.818551156	5.729399145
9.974465964	10.33055677	21.69954456	20.50732501	20.43957873
7.845723072	7.613547079	14.62630646	16.56851926	19.61824005
0.620216421	0.600016349	1.251493533	1.319408646	1.458481421
2.203769852	2.283506701	6.354763047	5.91790792	5.583850333
1.693843322	1.77975393	3.110405393	3.08822249	3.623045686
131.1477804	128.8256356	283.3244676	263.8668689	303.0961666
8.660545017	8.639948453	18.07964043	21.57651042	21.69265539
13.09177173	13.44511294	29.61221476	24.44167821	29.35972578
13.60690856	15.50228385	44.54158344	25.76670809	30.79805089
18.36815112	19.86383853	42.90343445	46.10988066	43.65185886
3.455159498	3.906443664	5.808870527	7.608208926	8.792614111
5.507578219	5.800318709	11.21365288	12.2936408	12.03989769
0.838510756	0.9193611	1.786552251	1.778219662	2.025218849

0.737351509	0.756136603	1.420555929	1.55800631	1.438682417
1.076406172	0.801037137	2.012642235	1.468604111	1.555434332
9.429317069	10.01896956	16.02815042	18.95521172	19.96623442
0.30410183	0.382456644	0.814471692	0.621453995	0.525660138
2.70481551	2.870383073	5.429164547	5.246534975	6.783785028
2.253180431	2.82741618	2.983890714	4.608313817	5.675525654

Supplementary Table 2: Decreased differential expression of genes in A2780 cells transfected with mir146 (A2780M) and A2780 cells transfected with miRctrl (A2780C).

**Transcript Id**	Gene Id(name)	**Mirna Name**	**miTG score**
ENST00000223095	ENSG00000106366 (SERPINE1)	hsa-miR-1825	0.997635826
UTR3	7:100780748-100780761	0.007831621	
UTR3	7:100781232-100781251	0.006438778	
UTR3	7:100782381-100782388	0.0986726	
UTR3	7:100777125-100777131	0.013094968	
ENST00000223095	ENSG00000106366 (SERPINE1)	hsa-miR-5011-5p	0.994960038
UTR3	7:100782182-100782188	0.010920577	
UTR3	7:100782183-100782190	0.019426852	
UTR3	7:100782207-100782213	0.010762316	
UTR3	7:100782208-100782214	0.008821141	
UTR3	7:100782230-100782236	0.009068804	

UTR3	7:100782231-100782238	0.01855238	
UTR3	7:100782235-100782242	0.019536452	
ENST00000223095	ENSG00000106366 (SERPINE1)	hsa-miR-2110	0.988454941
UTR3	7:100781238-100781265	0.001791457	
UTR3	7:100781319-100781346	0.003564891	
UTR3	7:100781453-100781477	0.001918674	
UTR3	7:100781888-100781905	0.076159462	
ENST00000223095	ENSG00000106366 (SERPINE1)	hsa-miR-548az-5p	0.96870096
UTR3	7:100780824-100780847	0.007260622	
UTR3	7:100781092-100781112	0.004870355	
UTR3	7:100782458-100782474	0.054673259	

ENST00000223095	ENSG00000106366 (SERPINE1)	hsa-miR-548t-5p	0.968445299
UTR3	7:100780830-100780847	0.00727993	
UTR3	7:100781085-100781112	0.004713773	
UTR3	7:100782458-100782474	0.054673259	
ENST00000223095	ENSG00000106366 (SERPINE1)	hsa-miR-6875-3p	0.966975366
UTR3	7:100780768-100780792	0.01135348	
UTR3	7:100780785-100780798	0.009062388	
UTR3	7:100781190-100781216	0.01058925	
UTR3	7:100781429-100781443	0.024454929	
UTR3	7:100782523-100782532	0.010437929	
ENST00000223095	ENSG00000106366 (SERPINE1)	hsa-miR-1277-5p	0.96565924

UTR3	7:100782182-100782193	0.025271695	
UTR3	7:100782197-100782216	0.01619347	
UTR3	7:100782230-100782245	0.023771843	
ENST00000223095	ENSG00000106366 (SERPINE1)	hsa-miR-4795-3p	0.956862971
UTR3	7:100781549-100781571	0.015891226	
UTR3	7:100782017-100782042	0.009771618	
UTR3	7:100782214-100782223	0.005794902	
UTR3	7:100782505-100782511	0.029902483	
ENST00000223095	ENSG00000106366 (SERPINE1)	hsa-miR-5681a	0.956059381
UTR3	7:100781201-100781226	0.049508561	
UTR3	7:100782351-100782365	0.006065312	

UTR3	7:100782351-100782369	0.005470958	
ENST00000223095	ENSG00000106366 (SERPINE1)	hsa-miR-190a-3p	0.953698689
UTR3	7:100782182-100782188	0.009644948	
UTR3	7:100782184-100782190	0.008130071	
UTR3	7:100782196-100782213	0.009066539	
UTR3	7:100782219-100782236	0.008427183	
UTR3	7:100782219-100782238	0.008087967	
UTR3	7:100782229-100782240	0.008681286	
UTR3	7:100782229-100782242	0.008111133	
ENST00000223095	ENSG00000106366 (SERPINE1)	hsa-miR-627-3p	0.9519028
UTR3	7:100780779-100780796	0.054750156	

UTR3	7:100780840-100780855	0.004746218	
ENST00000223095	ENSG00000106366 (SERPINE1)	hsa-miR-942-5p	0.943661625
UTR3	7:100780756-100780780	0.006275233	
UTR3	7:100780819-100780834	0.042528151	
UTR3	7:100780927-100780949	0.007966047	
ENST00000223095	ENSG00000106366 (SERPINE1)	hsa-miR-583	0.935994236
UTR3	7:100780801-100780818	0.007716477	
UTR3	7:100781586-100781600	0.002062419	
UTR3	7:100781724-100781733	0.002255503	
UTR3	7:100782163-100782177	0.003216421	
UTR3	7:100782321-100782346	0.035041481	

UTR3	7:100782340-100782351	0.003384174	
UTR3	7:100780323-100780344	0.015866171	
ENST00000223095	ENSG00000106366 (SERPINE1)	hsa-miR-548ah-5p	0.926505806
UTR3	7:100781140-100781158	0.002794142	
UTR3	7:100782075-100782097	0.003172988	
UTR3	7:100782453-100782473	0.046157712	
ENST00000223095	ENSG00000106366 (SERPINE1)	hsa-miR-6873-3p	0.925409107
UTR3	7:100780810-100780832	0.007785077	
UTR3	7:100780938-100780948	0.007606214	
UTR3	7:100781526-100781546	0.029700946	
UTR3	7:100781526-100781552	0.001158619	

UTR3	7:100781985-100782007	0.002062749	
UTR3	7:100781985-100782008	0.003549793	
ENST00000223095	ENSG00000106366 (SERPINE1)	hsa-miR-6833-3p	0.921957818
UTR3	7:100780819-100780833	0.008853651	
UTR3	7:100780930-100780949	0.00877637	
UTR3	7:100781526-100781547	0.0250342	
UTR3	7:100781526-100781552	0.002057273	
UTR3	7:100781989-100782008	0.004593504	
UTR3	7:100781989-100782008	0.001748078	
ENST00000223095	ENSG00000106366 (SERPINE1)	hsa-miR-6740-3p	0.921387221
UTR3	7:100780819-100780836	0.050432155	

UTR3	7:100773910-100773925	0.006921725	
ENST00000223095	ENSG00000106366 (SERPINE1)	hsa-miR-34a-5p	0.905589079
UTR3	7:100781145-100781169	0.001730397	
UTR3	7:100781150-100781174	0.004608328	
UTR3	7:100781664-100781689	0.041319561	
ENST00000223095	ENSG00000106366 (SERPINE1)	hsa-miR-3925-3p	0.905236509
UTR3	7:100780748-100780762	0.041749797	
UTR3	7:100781357-100781374	0.005841203	
ENST00000223095	ENSG00000106366 (SERPINE1)	hsa-miR-937-5p	0.893200844
UTR3	7:100781791-100781813	0.002757756	
UTR3	7:100781896-100781921	0.013473886	

UTR3	7:100781921-100781940	0.02918642	
ENST00000223095	ENSG00000106366 (SERPINE1)	hsa-miR-8063	0.891001385
UTR3	7:100781345-100781371	0.004845774	
UTR3	7:100782352-100782376	0.004253444	
UTR3	7:100782364-100782381	0.023908285	
UTR3	7:100782460-100782475	0.01203709	
ENST00000223095	ENSG00000106366 (SERPINE1)	hsa-miR-449a	0.890923422
UTR3	7:100781150-100781169	0.001667749	
UTR3	7:100781150-100781174	0.003537364	
UTR3	7:100781664-100781689	0.039826364	
ENST00000223095	ENSG00000106366 (SERPINE1)	hsa-miR-642a-3p	0.886393906

UTR3	7:100781592-100781611	0.00358874	
UTR3	7:100781931-100781951	0.040694445	
ENST00000223095	ENSG00000106366 (SERPINE1)	hsa-miR-4713-5p	0.882336243
UTR3	7:100780929-100780946	0.007249487	
UTR3	7:100782252-100782267	0.036385136	
ENST00000223095	ENSG00000106366 (SERPINE1)	hsa-miR-143-3p	0.880986481
UTR3	7:100781206-100781232	0.039294929	
UTR3	7:100781322-100781337	0.002314947	
UTR3	7:100781605-100781622	0.001813306	
ENST00000223095	ENSG00000106366 (SERPINE1)	hsa-miR-4433b-5p	0.879250919
UTR3	7:100780727-100780753	0.039605692	

UTR3	7:100781640-100781663	0.002492038	
UTR3	7:100781790-100781805	0.0010566	
ENST00000223095	ENSG00000106366 (SERPINE1)	hsa-miR-642b-3p	0.879127526
UTR3	7:100781590-100781611	0.003481363	
UTR3	7:100781931-100781951	0.039653981	
ENST00000223095	ENSG00000106366 (SERPINE1)	hsa-miR-494-3p	0.877294909
UTR3	7:100781580-100781596	0.002545872	
UTR3	7:100782486-100782500	0.039529726	
UTR3	7:100771789-100771805	0.006648048	
ENST00000223095	ENSG00000106366 (SERPINE1)	hsa-miR-5195-3p	0.861871702
UTR3	7:100780738-100780761	0.027695242	

UTR3	7:100782364-100782388	0.012935057	
ENST00000223095	ENSG00000106366 (SERPINE1)	hsa-miR-450a-2-3p	0.860651033
UTR3	7:100781288-100781301	0.003227189	
UTR3	7:100781451-100781478	0.003439338	
UTR3	7:100781891-100781906	0.033796808	
ENST00000223095	ENSG00000106366 (SERPINE1)	hsa-miR-548e-5p	0.857714087
UTR3	7:100781085-100781112	0.008230865	
UTR3	7:100782440-100782465	0.016752853	
UTR3	7:100782459-100782474	0.01508286	
ENST00000223095	ENSG00000106366 (SERPINE1)	hsa-miR-3620-3p	0.851869791
UTR3	7:100781909-100781916	0.03826738	

UTR3	7:100781977-100782002	0.001029555	
ENST00000223095	ENSG00000106366 (SERPINE1)	hsa-miR-486-5p	0.848971624
UTR3	7:100781089-100781105	0.004321364	
UTR3	7:100781244-100781272	0.005534687	
UTR3	7:100781886-100781913	0.004723331	
UTR3	7:100782389-100782412	0.024345174	
ENST00000223095	ENSG00000106366 (SERPINE1)	hsa-miR-139-3p	0.84525307
UTR3	7:100780863-100780877	0.011006012	
UTR3	7:100780902-100780928	0.006103746	
UTR3	7:100780961-100780978	0.017129362	
UTR3	7:100782306-100782330	0.004216151	

ENST00000223095	ENSG00000106366 (SERPINE1)	hsa-miR-5689	0.843261737
UTR3	7:100782446-100782462	0.038207744	
ENST00000223095	ENSG00000106366 (SERPINE1)	hsa-miR-6882-5p	0.840420999
UTR3	7:100781136-100781164	0.004109333	
UTR3	7:100781366-100781387	0.033749692	
ENST00000223095	ENSG00000106366 (SERPINE1)	hsa-miR-149-3p	0.838501942
UTR3	7:100780902-100780920	0.004375449	
UTR3	7:100780941-100780967	0.003346436	
UTR3	7:100781245-100781262	0.00581052	
UTR3	7:100781316-100781340	0.001045278	
UTR3	7:100781325-100781343	0.023048604	

ENST00000223095	ENSG00000106366 (SERPINE1)	hsa-miR-148a-3p	0.832001974
UTR3	7:100781050-100781069	0.002902166	
UTR3	7:100781231-100781251	0.001961603	
UTR3	7:100782318-100782334	0.003231279	
UTR3	7:100782371-100782387	0.028759131	
ENST00000223095	ENSG00000106366 (SERPINE1)	hsa-miR-5702	0.830357909
UTR3	7:100780803-100780815	0.004919744	
UTR3	7:100781795-100781813	0.001791076	
UTR3	7:100781909-100781920	0.010897069	
UTR3	7:100781929-100781939	0.019054794	
ENST00000223095	ENSG00000106366 (SERPINE1)	hsa-miR-4311	0.8294151

UTR3	7:100780799-100780819	0.011268698	
UTR3	7:100781712-100781733	0.001456111	
UTR3	7:100782155-100782177	0.00840966	
UTR3	7:100782329-100782347	0.015419064	
ENST00000223095	ENSG00000106366 (SERPINE1)	hsa-miR-3977	0.827006728
UTR3	7:100780940-100780959	0.024271334	
UTR3	7:100781377-100781402	0.012005536	
ENST00000223095	ENSG00000106366 (SERPINE1)	hsa-miR-4269	0.822116229
UTR3	7:100780981-100780998	0.00194273	
UTR3	7:100781101-100781120	0.030366483	
UTR3	7:100781150-100781172	0.003415089	

ENST00000223095	ENSG00000106366 (SERPINE1)	hsa-miR-6764-5p	0.819392395
UTR3	7:100780924-100780943	0.030328506	
UTR3	7:100782133-100782151	0.003582763	
UTR3	7:100778800-100778826	0.009378793	
ENST00000223095	ENSG00000106366 (SERPINE1)	hsa-miR-148b-3p	0.818363233
UTR3	7:100781043-100781069	0.002880755	
UTR3	7:100782318-100782334	0.003231279	
UTR3	7:100782371-100782387	0.029196248	
ENST00000223095	ENSG00000106366 (SERPINE1)	hsa-miR-152-3p	0.81512564
UTR3	7:100781042-100781069	0.002786637	
UTR3	7:100782319-100782334	0.002971852	

UTR3	7:100782371-100782387	0.029196248	
ENST00000223095	ENSG00000106366 (SERPINE1)	hsa-miR-34c-5p	0.814115119
UTR3	7:100781150-100781170	0.002411372	
UTR3	7:100781665-100781688	0.032433999	
ENST00000223095	ENSG00000106366 (SERPINE1)	hsa-miR-6809-3p	0.813365483
UTR3	7:100780819-100780833	0.018204809	
UTR3	7:100780930-100780949	0.007393292	
UTR3	7:100781540-100781546	0.004033458	
UTR3	7:100781540-100781552	0.005132976	
ENST00000223095	ENSG00000106366 (SERPINE1)	hsa-miR-6825-5p	0.813118475
UTR3	7:100781248-100781264	0.005908855	

UTR3	7:100781322-100781345	0.024147623	
UTR3	7:100781878-100781904	0.004681475	
ENST00000223095	ENSG00000106366 (SERPINE1)	hsa-miR-6763-5p	0.811818804
UTR3	7:100781248-100781265	0.006078236	
UTR3	7:100781319-100781346	0.019261608	
UTR3	7:100781885-100781904	0.009258694	
ENST00000223095	ENSG00000106366 (SERPINE1)	hsa-miR-3153	0.81104848
UTR3	7:100781725-100781739	0.005035744	
UTR3	7:100781877-100781903	0.02948051	
ENST00000223095	ENSG00000106366 (SERPINE1)	hsa-miR-6730-5p	0.803825582
UTR3	7:100780810-100780820	0.007658443	

UTR3	7:100781217-100781227	0.009870424	
UTR3	7:100782320-100782348	0.005386617	
UTR3	7:100782339-100782365	0.005380421	
UTR3	7:100782348-100782369	0.005461022	
ENST00000223095	ENSG00000106366 (SERPINE1)	hsa-miR-676-3p	0.803163924
UTR3	7:100781789-100781806	0.002023686	
UTR3	7:100781808-100781824	0.005765452	
UTR3	7:100781871-100781893	0.010280204	
UTR3	7:100782530-100782544	0.015619094	
ENST00000223095	ENSG00000106366 (SERPINE1)	hsa-miR-541-3p	0.80305785
UTR3	7:100782300-100782321	0.033097131	

UTR3	7:100771759-100771774	0.003508486	
ENST00000223095	ENSG00000106366 (SERPINE1)	hsa-miR-4770	0.801958764
UTR3	7:100781206-100781232	0.031355584	
UTR3	7:100781322-100781337	0.002208546	
ENST00000223095	ENSG00000106366 (SERPINE1)	hsa-miR-3620-5p	0.800656831
UTR3	7:100781021-100781041	0.003322177	
UTR3	7:100781655-100781682	0.002894824	
UTR3	7:100782115-100782131	0.002736555	
UTR3	7:100782304-100782319	0.024476925	
ENST00000223095	ENSG00000106366 (SERPINE1)	hsa-miR-10a-5p	0.796589119
UTR3	7:100781890-100781915	0.030531703	

UTR3	7:100782045-100782069	0.002485381	
ENST00000223095	ENSG00000106366 (SERPINE1)	hsa-miR-10b-5p	0.796589119
UTR3	7:100781890-100781915	0.030531703	
UTR3	7:100782045-100782069	0.002485381	
ENST00000223095	ENSG00000106366 (SERPINE1)	hsa-miR-449b-5p	0.795275996
UTR3	7:100781150-100781170	0.002447674	
UTR3	7:100781664-100781688	0.030437277	
ENST00000223095	ENSG00000106366 (SERPINE1)	hsa-miR-6752-5p	0.795260505
UTR3	7:100781457-100781475	0.029744328	
UTR3	7:100782142-100782169	0.003139067	
ENST00000223095	ENSG00000106366 (SERPINE1)	hsa-miR-654-5p	0.794939037

UTR3	7:100782312-100782321	0.032056509	
UTR3	7:100771764-100771774	0.004647293	
ENST00000223095	ENSG00000106366 (SERPINE1)	hsa-miR-4450	0.794768556
UTR3	7:100781244-100781264	0.001629698	
UTR3	7:100781330-100781345	0.002308638	
UTR3	7:100781883-100781904	0.00543628	
UTR3	7:100782317-100782343	0.023459442	
ENST00000223095	ENSG00000106366 (SERPINE1)	hsa-miR-5093	0.794483963
UTR3	7:100781936-100781957	0.032805556	
ENST00000223095	ENSG00000106366 (SERPINE1)	hsa-miR-573	0.792639387
UTR3	7:100781921-100781944	0.032621527	

ENST00000223095	ENSG00000106366 (SERPINE1)	hsa-miR-656-3p	0.789267252
UTR3	7:100781547-100781572	0.015225455	
UTR3	7:100782026-100782043	0.01115956	
UTR3	7:100782218-100782223	0.005903181	
ENST00000223095	ENSG00000106366 (SERPINE1)	hsa-miR-609	0.786276832
UTR3	7:100781202-100781223	0.029909036	
UTR3	7:100781638-100781653	0.002086818	
ENST00000223095	ENSG00000106366 (SERPINE1)	hsa-miR-134-5p	0.785887598
UTR3	7:100781131-100781147	0.031958022	
ENST00000223095	ENSG00000106366 (SERPINE1)	hsa-miR-425-5p	0.784172712
UTR3	7:100781927-100781954	0.031791938	

ENST00000223095	ENSG00000106366 (SERPINE1)	hsa-miR-148b-5p	0.782356878
UTR3	7:100781527-100781554	0.002965748	
UTR3	7:100781881-100781898	0.028651367	
ENST00000223095	ENSG00000106366 (SERPINE1)	hsa-miR-6857-5p	0.781939374
UTR3	7:100781247-100781265	0.001405382	
UTR3	7:100781453-100781477	0.002164388	
UTR3	7:100781878-100781905	0.028007298	
ENST00000223095	ENSG00000106366 (SERPINE1)	hsa-miR-548n	0.774355642
UTR3	7:100780827-100780847	0.00729816	
UTR3	7:100781091-100781112	0.004679775	
UTR3	7:100782446-100782474	0.018881095	

ENST00000223095	ENSG00000106366 (SERPINE1)	hsa-miR-4531	0.771245423
UTR3	7:100780864-100780877	0.004385417	
UTR3	7:100780905-100780928	0.003402097	
UTR3	7:100780953-100780978	0.006972477	
UTR3	7:100782311-100782330	0.01580952	
ENST00000223095	ENSG00000106366 (SERPINE1)	hsa-miR-1343-3p	0.767631267
UTR3	7:100781084-100781106	0.003089262	
UTR3	7:100781864-100781890	0.004430233	
UTR3	7:100782109-100782134	0.022717042	
ENST00000223095	ENSG00000106366 (SERPINE1)	hsa-miR-4666a-5p	0.766633082
UTR3	7:100782386-100782407	0.003821669	

UTR3	7:100782483-100782489	0.026323543	
ENST00000223095	ENSG00000106366 (SERPINE1)	hsa-miR-150-5p	0.766096367
UTR3	7:100780927-100780945	0.006973748	
UTR3	7:100782081-100782103	0.006685377	
UTR3	7:100782260-100782266	0.016437094	
ENST00000223095	ENSG00000106366 (SERPINE1)	hsa-miR-106a-5p	0.765853509
UTR3	7:100782072-100782097	0.007424559	
UTR3	7:100782449-100782473	0.022649518	
ENST00000223095	ENSG00000106366 (SERPINE1)	hsa-miR-378a-5p	0.765175639
UTR3	7:100781090-100781106	0.00303355	
UTR3	7:100781612-100781625	0.00321962	

UTR3	7:100782107-100782134	0.003421746	
UTR3	7:100782522-100782541	0.02033744	
ENST00000223095	ENSG00000106366 (SERPINE1)	hsa-miR-4656	0.763575164
UTR3	7:100781656-100781681	0.001054621	
UTR3	7:100782109-100782130	0.001716536	
UTR3	7:100782299-100782318	0.024699976	
UTR3	7:100779079;100780282-100779082;100780300	0.012251369	
ENST00000223095	ENSG00000106366 (SERPINE1)	hsa-miR-2116-3p	0.762394082
UTR3	7:100780921-100780944	0.003697272	
UTR3	7:100782082-100782103	0.005520466	
UTR3	7:100782098-100782116	0.001795028	

UTR3	7:100782258-100782265	0.018747607	
ENST00000223095	ENSG00000106366 (SERPINE1)	hsa-miR-224-5p	0.762128251
UTR3	7:100781369-100781386	0.029736398	
ENST00000223095	ENSG00000106366 (SERPINE1)	hsa-miR-4646-3p	0.760284233
UTR3	7:100780729-100780754	0.029570593	
ENST00000223095	ENSG00000106366 (SERPINE1)	hsa-miR-8064	0.757236157
UTR3	7:100781096-100781117	0.00582646	
UTR3	7:100781336-100781357	0.023471966	
ENST00000223095	ENSG00000106366 (SERPINE1)	hsa-miR-6842-3p	0.757208745
UTR3	7:100781619-100781635	0.00563068	
UTR3	7:100782045-100782063	0.021183293	

UTR3	7:100782379-100782403	0.002482017	
ENST00000223095	ENSG00000106366 (SERPINE1)	hsa-miR-6852-3p	0.754890232
UTR3	7:100780896-100780902	0.006689343	
UTR3	7:100781798-100781823	0.01056538	
UTR3	7:100781868-100781892	0.004099112	
UTR3	7:100781963-100781982	0.00331646	
UTR3	7:100782525-100782543	0.004420239	
ENST00000223095	ENSG00000106366 (SERPINE1)	hsa-miR-6879-3p	0.750534037
UTR3	7:100781169-100781195	0.002423079	
UTR3	7:100781359-100781383	0.005831009	
UTR3	7:100781903-100781917	0.014057383	

UTR3	7:100781911-100781936	0.006396555	
ENST00000223095	ENSG00000106366 (SERPINE1)	hsa-miR-6719-3p	0.747412723
UTR3	7:100781427-100781451	0.013691503	
UTR3	7:100782525-100782540	0.014745184	
ENST00000223095	ENSG00000106366 (SERPINE1)	hsa-miR-3118	0.743975692
UTR3	7:100781123-100781147	0.028140457	
ENST00000223095	ENSG00000106366 (SERPINE1)	hsa-miR-4753-3p	0.742424568
UTR3	7:100780812-100780832	0.011178365	
UTR3	7:100780927-100780948	0.00309384	
UTR3	7:100781528-100781552	0.013735419	
ENST00000223095	ENSG00000106366 (SERPINE1)	hsa-miR-196b-5p	0.740873888

UTR3	7:100781479-100781503	0.001999781	
UTR3	7:100782011-100782032	0.02587557	
ENST00000223095	ENSG00000106366 (SERPINE1)	hsa-miR-7159-5p	0.739589791
UTR3	7:100782432-100782453	0.027766207	
ENST00000223095	ENSG00000106366 (SERPINE1)	hsa-miR-4302	0.738600827
UTR3	7:100781229-100781251	0.002545336	
UTR3	7:100781668-100781686	0.010540899	
UTR3	7:100782368-100782387	0.014596151	
ENST00000223095	ENSG00000106366 (SERPINE1)	hsa-miR-1276	0.737296966
UTR3	7:100780813-100780818	0.008029756	
UTR3	7:100781720-100781733	0.001428314	

UTR3	7:100782165-100782178	0.0074278	
UTR3	7:100782214-100782228	0.003917485	
UTR3	7:100782322-100782346	0.006768831	
ENST00000223095	ENSG00000106366 (SERPINE1)	hsa-miR-153-5p	0.735718407
UTR3	7:100782268-100782288	0.005740472	
UTR3	7:100782472-100782495	0.021698769	
ENST00000223095	ENSG00000106366 (SERPINE1)	hsa-miR-3613-3p	0.735345441
UTR3	7:100780821-100780848	0.007338874	
UTR3	7:100781086-100781113	0.004777744	
UTR3	7:100782351-100782375	0.015291286	
ENST00000223095	ENSG00000106366 (SERPINE1)	hsa-miR-519c-3p	0.731499972

UTR3	7:100782089-100782097	0.010896821	
UTR3	7:100782449-100782472	0.016189617	
ENST00000223095	ENSG00000106366 (SERPINE1)	hsa-miR-519d-3p	0.731019026
UTR3	7:100782073-100782098	0.011689438	
UTR3	7:100782460-100782472	0.015357002	
ENST00000223095	ENSG00000106366 (SERPINE1)	hsa-miR-519b-3p	0.730120544
UTR3	7:100782077-100782097	0.010974121	
UTR3	7:100782458-100782472	0.015997716	
ENST00000223095	ENSG00000106366 (SERPINE1)	hsa-miR-7110-3p	0.729821255
UTR3	7:100781520-100781545	0.014717419	
UTR3	7:100781985-100782006	0.001501586	

UTR3	7:100781985-100782008	0.008588356	
UTR3	7:100773881-100773893	0.01019625	
ENST00000223095	ENSG00000106366 (SERPINE1)	hsa-miR-6783-3p	0.726774705
UTR3	7:100781864-100781890	0.00428711	
UTR3	7:100782109-100782134	0.022408278	
ENST00000223095	ENSG00000106366 (SERPINE1)	hsa-miR-4306	0.726340232
UTR3	7:100780868-100780877	0.004083175	
UTR3	7:100780881-100780893	0.006167539	
UTR3	7:100780914-100780928	0.002574168	
UTR3	7:100780961-100780978	0.005450141	
UTR3	7:100781311-100781322	0.001637292	

UTR3	7:100782314-100782330	0.005258227	
UTR3	7:100778867;100778996-100778875;100779011	0.007162562	
ENST00000223095	ENSG00000106366 (SERPINE1)	hsa-miR-186-5p	0.726181944
UTR3	7:100781580-100781600	0.003341787	
UTR3	7:100782331-100782351	0.007486211	
UTR3	7:100782339-100782363	0.005183556	
UTR3	7:100782341-100782367	0.005403993	
UTR3	7:100782346-100782371	0.005231086	
ENST00000223095	ENSG00000106366 (SERPINE1)	hsa-miR-548an	0.724761706
UTR3	7:100780827-100780846	0.02341227	
UTR3	7:100781401-100781419	0.003117812	

ENST00000223095	ENSG00000106366 (SERPINE1)	hsa-miR-510-3p	0.723572764
UTR3	7:100781924-100781946	0.00592147	
UTR3	7:100782484-100782501	0.02051133	
ENST00000223095	ENSG00000106366 (SERPINE1)	hsa-miR-627-5p	0.722212065
UTR3	7:100781805-100781813	0.009063749	
UTR3	7:100781899-100781921	0.006804113	
UTR3	7:100781924-100781940	0.010453919	
ENST00000223095	ENSG00000106366 (SERPINE1)	hsa-miR-629-3p	0.721750991
UTR3	7:100781526-100781553	0.003400887	
UTR3	7:100782256-100782268	0.022883352	
ENST00000223095	ENSG00000106366 (SERPINE1)	hsa-miR-548aw	0.721655894

UTR3	7:100782250-100782273	0.007392903	
UTR3	7:100782374-100782384	0.018883596	
ENST00000223095	ENSG00000106366 (SERPINE1)	hsa-miR-4417	0.721507854
UTR3	7:100781025-100781042	0.002891767	
UTR3	7:100781663-100781682	0.002923337	
UTR3	7:100782306-100782320	0.020449353	
ENST00000223095	ENSG00000106366 (SERPINE1)	hsa-miR-4700-5p	0.72091557
UTR3	7:100781245-100781265	0.003381828	
UTR3	7:100781322-100781347	0.022834484	
ENST00000223095	ENSG00000106366 (SERPINE1)	hsa-miR-5571-5p	0.719879718
UTR3	7:100780779-100780799	0.008371815	

UTR3	7:100782256-100782270	0.010702948	
UTR3	7:100782434-100782456	0.007057497	
ENST00000223095	ENSG00000106366 (SERPINE1)	hsa-miR-6865-5p	0.719255062
UTR3	7:100780907-100780932	0.002259464	
UTR3	7:100781674-100781692	0.023822203	
ENST00000223095	ENSG00000106366 (SERPINE1)	hsa-miR-196a-5p	0.718193945
UTR3	7:100781479-100781503	0.002378739	
UTR3	7:100782011-100782032	0.023617139	
ENST00000223095	ENSG00000106366 (SERPINE1)	hsa-miR-185-5p	0.715063469
UTR3	7:100780864-100780877	0.003908891	
UTR3	7:100780881-100780893	0.006167539	

UTR3	7:100780905-100780928	0.002179067	
UTR3	7:100780960-100780978	0.005419617	
UTR3	7:100781304-100781322	0.001542223	
UTR3	7:100782311-100782330	0.005000923	
UTR3	7:100778867;100778996-100778875;100779011	0.007162562	
ENST00000223095	ENSG00000106366 (SERPINE1)	hsa-miR-519a-3p	0.713673753
UTR3	7:100782072-100782097	0.011193023	
UTR3	7:100782467-100782472	0.014439586	
ENST00000223095	ENSG00000106366 (SERPINE1)	hsa-miR-3133	0.708115406
UTR3	7:100782223-100782228	0.004551498	
UTR3	7:100782345-100782351	0.005402735	

UTR3	7:100782352-100782363	0.005231437	
UTR3	7:100782352-100782367	0.005161163	
UTR3	7:100782352-100782371	0.004843747	
ENST00000223095	ENSG00000106366 (SERPINE1)	hsa-miR-141-3p	0.707945317
UTR3	7:100780785-100780808	0.022896105	
UTR3	7:100781571-100781594	0.002281027	
ENST00000223095	ENSG00000106366 (SERPINE1)	hsa-miR-6731-3p	0.707765931
UTR3	7:100781429-100781436	0.016421203	
UTR3	7:100781823-100781849	0.003290554	
UTR3	7:100782276-100782303	0.005451198	
ENST00000223095	ENSG00000106366 (SERPINE1)	hsa-miR-299-5p	0.706059227

UTR3	7:100780846-100780865	0.0220396	
UTR3	7:100780971-100780983	0.002988721	
ENST00000223095	ENSG00000106366 (SERPINE1)	hsa-miR-6817-3p	0.702402554
UTR3	7:100780926-100780947	0.003701855	
UTR3	7:100781521-100781545	0.014176444	
UTR3	7:100781985-100782006	0.002113285	
UTR3	7:100781995-100782008	0.004749805	
ENST00000223095	ENSG00000106366 (SERPINE1)	hsa-miR-19b-3p	0.702177595
UTR3	7:100782258-100782274	0.013807608	
UTR3	7:100782367-100782384	0.010916196	
ENST00000223095	ENSG00000106366 (SERPINE1)	hsa-miR-4422	0.702097274

UTR3	7:100782446-100782465	0.024717527	
ENST00000223095	ENSG00000106366 (SERPINE1)	hsa-miR-19a-3p	0.701418361
UTR3	7:100782260-100782274	0.013748314	
UTR3	7:100782367-100782384	0.010916196	
ENST00000223095	ENSG00000106366 (SERPINE1)	hsa-miR-7974	0.700877749
UTR3	7:100781909-100781925	0.023006242	
UTR3	7:100782126-100782148	0.0016161	

Supplementary Table 3: targets with miRNAs found in genes ENSG0000010636 (SERPINE1)

Transcript Id	Gene Id(name)	Mirna Name	miTG score
ENST00000599847	ENSG00000152439 (ZNF773)	hsa-miR-146a-5p	0.999996
UTR3	19:58024644-58024660	0.162166849	
UTR3	19:58026567-58026594	0.00549672	
UTR3	19:58026679-58026693	0.002766695	
UTR3	19:58028097-58028113	0.004807814	
UTR3	19:58029501-58029522	0.037791942	
ENST00000526995	ENSG00000175104 (TRAF6)	hsa-miR-146a-5p	0.999978
UTR3	11:36511323-36511346	0.022150809	
UTR3	11:36510908-36510929	0.069665204	
UTR3	11:36510843-36510867	0.081734012	
UTR3	11:36510109-36510127	0.012250438	
ENST00000369980	ENSG00000184216 (IRAK1)	hsa-miR-146a-5p	0.999972
UTR3	X:153277263-153277288	0.10883198	
UTR3	X:153277247-153277266	0.072978108	
ENST00000370900	ENSG00000147255 (IGSF1)	hsa-miR-146a-5p	0.999866
UTR3	X:130418887-130418912	0.01756038	
UTR3	X:130418158-130418179	0.138879909	
ENST00000551765	ENSG00000257218 (GATC)	hsa-miR-146a-5p	0.999528
UTR3	12:120899289-120899315	0.135881871	
ENST00000303296	ENSG00000110422 (HIPK3)	hsa-miR-146a-5p	0.999371
UTR3	11:33378413-33378426	0.131167285	
ENST00000361264	ENSG00000198876 (DCAF12)	hsa-miR-146a-5p	0.99899
UTR3	9:34086854-34086875	0.002749271	
UTR3	9:34086698-34086719	0.005392711	
UTR3	9:34086570-34086593	0.115283263	
ENST00000540664	ENSG00000196233 (LCOR)	hsa-miR-146a-5p	0.998541
UTR3	10:98718333-98718354	0.007648157	
UTR3	10:98719279-98719304	0.003221005	
UTR3	10:98719941-98719961	0.001179491	
UTR3	10:98720586-98720604	0.001349985	
UTR3	10:98721368-98721378	0.00256124	
UTR3	10:98722676-98722703	0.002038013	
UTR3	10:98723405-98723420	0.012527313	
UTR3	10:98724122-98724137	0.08688245	
ENST00000311015	ENSG00000172687 (ZNF738)	hsa-miR-146a-5p	0.998472
UTR3	19:21560902-21560921	0.007837571	
UTR3	19:21560918-21560936	0.019164832	
UTR3	19:21561492-21561511	0.053871422	
UTR3	19:21561920-21561947	0.03577717	
ENST00000325144	ENSG00000181472 (ZBTB2)	hsa-miR-146a-5p	0.99803
UTR3	6:151686574-151686598	0.020209292	
UTR3	6:151686543-151686562	0.004069393	
UTR3	6:151686289-151686310	0.004942405	
UTR3	6:151685305-151685326	0.083272067	

ENST00000309446	ENSG00000118263 (KLF7)	hsa-miR-146a-5p	0.997917
UTR3	2:207945824-207945852	0.08048381	
UTR3	2:207945519-207945547	0.005714625	
UTR3	2:207944876-207944900	0.005998419	
UTR3	2:207943584-207943595	0.00584079	
UTR3	2:207943302-207943323	0.002827208	
UTR3	2:207942677-207942689	0.001928592	
UTR3	2:207940603-207940622	0.005706465	
UTR3	2:207939171-207939190	0.003074982	
ENST00000295025	ENSG00000162924 (REL)	hsa-miR-146a-5p	0.996836
UTR3	2:61151027-61151044	0.001637971	
UTR3	2:61151217-61151234	0.001537235	
UTR3	2:61151958-61151985	0.001533778	
UTR3	2:61152432-61152455	0.001577663	
UTR3	2:61153748-61153767	0.003562602	
UTR3	2:61154027-61154049	0.002116903	
UTR3	2:61155043-61155068	0.001251838	
UTR3	2:61156712-61156733	0.001070489	
UTR3	2:61157488-61157507	0.001576509	
UTR3	2:61158582-61158589	0.088861545	
ENST00000589717	ENSG00000256771 (ZNF253)	hsa-miR-146a-5p	0.996391
UTR3	19:20003682-20003702	0.043465348	
UTR3	19:20003771-20003789	0.042273763	
UTR3	19:20005318-20005333	0.023045069	
UTR3	19:19990947;20002283-19990955;20002295	0.025854842	
ENST00000375256	ENSG00000165244 (ZNF367)	hsa-miR-146a-5p	0.994372
UTR3	9:99150215-99150235	0.090060273	
UTR3	9:99149779-99149804	0.005216236	
ENST00000592773	ENSG00000198597 (ZNF536)	hsa-miR-146a-5p	0.993696
UTR3	19:31202624-31202649	0.002316742	
UTR3	19:31203616-31203639	0.067381133	
UTR3	19:31204165-31204181	0.023711034	
ENST00000368599	ENSG00000178033 (FAM26E)	hsa-miR-146a-5p	0.993304
UTR3	6:116837627-116837655	0.059125776	
UTR3	6:116838112-116838124	0.001946717	
UTR3	6:116838207-116838222	0.00121829	
UTR3	6:116838310-116838326	0.001223976	
UTR3	6:116839704-116839722	0.003290396	
UTR3	6:116840077-116840097	0.005254453	
UTR3	6:116841391-116841419	0.001576348	
UTR3	6:116842165-116842191	0.001106404	
UTR3	6:116844081-116844102	0.006878417	
UTR3	6:116844207-116844231	0.002300399	
UTR3	6:116845617-116845636	0.002722865	
UTR3	6:116845794-116845805	0.005774964	

ENST00000485895	ENSG00000157578 (LCA5L)	hsa-miR-146a-5p	0.992713
UTR3	21:40793523-40793543	0.032139976	
UTR3	21:40793506-40793524	0.065419046	
UTR3	21:40800229-40800250	0.036268404	
ENST00000576251	ENSG00000101574 (METTL4)	hsa-miR-146a-5p	0.990927
UTR3	18:2539046-2539071	0.07588562	
UTR3	18:2537938-2537954	0.004395617	
UTR3	18:2537532-2537551	0.007132501	
ENST00000286614	ENSG00000156103 (MMP16)	hsa-miR-146a-5p	0.989699
UTR3	8:89052958-89052986	0.016858117	
UTR3	8:89052202-89052229	0.057045567	
UTR3	8:89051674-89051694	0.002189713	
UTR3	8:89045113-89045141	0.009225133	
ENST00000382852	ENSG00000169953 (HSFY2)	hsa-miR-146a-5p	0.988862
UTR3	Y:20931130-20931157	0.084027364	
ENST00000290271	ENSG00000159167 (STC1)	hsa-miR-146a-5p	0.988056
UTR3	8:23699890-23699915	0.082872295	
ENST00000334681	ENSG00000187094 (CCK)	hsa-miR-146a-5p	0.987618
UTR3	3:42299458-42299477	0.082276863	
ENST00000433818	ENSG00000149262 (INTS4)	hsa-miR-146a-5p	0.986014
UTR3	11:77652272-77652294	0.066292961	
UTR3	11:77632452-77632469	0.010846087	
UTR3	11:77632405-77632426	0.001715156	
UTR3	11:77618768-77618795	0.001404855	
ENST00000338965	ENSG00000188211 (NCR3LG1)	hsa-miR-146a-5p	0.984424
UTR3	11:17394132-17394148	0.038371797	
UTR3	11:17395248-17395265	0.006769936	
UTR3	11:17397082-17397108	0.011408701	
UTR3	11:17397559-17397576	0.021922331	
ENST00000369649	ENSG00000126903 (SLC10A3)	hsa-miR-146a-5p	0.984234
UTR3	X:153715790-153715816	0.078824117	
UTR3	X:153716237-153716258	0.006924871	
ENST00000264246	ENSG00000121594 (CD80)	hsa-miR-146a-5p	0.983963
UTR3	3:119244014-119244036	0.00456856	
UTR3	3:119243789-119243807	0.068361995	
UTR3	3:119243349-119243370	0.005057847	
ENST00000555818	ENSG00000197555 (SIPA1L1)	hsa-miR-146a-5p	0.983428
UTR3	14:72206516-72206541	0.001932333	
UTR3	14:72207605-72207626	0.073432655	
UTR3	14:72207870-72207896	0.003735818	
UTR3	14:72054753-72054777	0.014745367	
UTR3	14:72197011;72200383-72197018;72200393	0.005666713	

ENST00000446113	ENSG00000177054 (ZDHHC13)	hsa-miR-146a-5p	0.982959
UTR3	11:19197628-19197653	0.076979167	
ENST00000313899	ENSG00000138668 (HNRNPD)	hsa-miR-146a-5p	0.98259
UTR3	4:83275160-83275187	0.076623141	
ENST00000371388	ENSG00000131721 (RHOXF2)	hsa-miR-146a-5p	0.98248
UTR3	X:119297888-119297911	0.076518911	
ENST00000379391	ENSG00000166881 (TMEM194A)	hsa-miR-146a-5p	0.98155
UTR3	12:57452981-57452999	0.068012716	
UTR3	12:57452706-57452727	0.003267033	
UTR3	12:57452588-57452611	0.001285431	
UTR3	12:57452281-57452308	0.001279032	
UTR3	12:57451801-57451817	0.001813596	
ENST00000591639	ENSG00000081665 (ZNF506)	hsa-miR-146a-5p	0.980274
UTR3	19:19905215-19905235	0.034181582	
UTR3	19:19904826-19904844	0.040361526	
ENST00000605860	ENSG00000007001 (UPP2)	hsa-miR-146a-5p	0.980101
UTR3	2:158991475-158991501	0.070411781	
UTR3	2:158992322-158992344	0.003986281	
ENST00000299167	ENSG00000102893 (PHKB)	hsa-miR-146a-5p	0.977294
UTR3	16:47733466-47733488	0.053193894	
UTR3	16:47733755-47733778	0.019637868	
UTR3	16:47684491-47684518	0.013391224	
ENST00000608872	ENSG00000119402 (FBXW2)	hsa-miR-146a-5p	0.976567
UTR3	9:123526740-123526762	0.068016148	
UTR3	9:123523696-123523717	0.001775084	
UTR3	9:123520365-123520378	0.00187573	
ENST00000490715	ENSG00000007341 (ST7L)	hsa-miR-146a-5p	0.975657
UTR3	1:113067415-113067439	0.0042105	
UTR3	1:113067037-113067062	0.004095764	
UTR3	1:113066159-113066187	0.062722518	
ENST00000342788	ENSG00000178568 (ERBB4)	hsa-miR-146a-5p	0.975636
UTR3	2:212247377-212247394	0.004531221	
UTR3	2:212246814-212246836	0.002219412	
UTR3	2:212244136-212244156	0.002083036	
UTR3	2:212243884-212243902	0.001060149	
UTR3	2:212243301-212243319	0.002388579	
UTR3	2:212241602-212241626	0.00192031	
UTR3	2:212240641-212240654	0.057144256	
UTR3	2:212248401-212248426	0.008386047	
ENST00000420940	ENSG00000114742 (WDR48)	hsa-miR-146a-5p	0.974994
UTR3	3:39116412-39116439	0.049608154	
UTR3	3:39125718-39125735	0.020970741	
ENST00000526148	ENSG00000110321 (EIF4G2)	hsa-miR-146a-5p	0.974218

UTR3	11:10819100-10819127	0.070066405	
ENST00000325881	ENSG00000157734 (SNX22)	hsa-miR-146a-5p	0.974077
UTR3	15:64448598-64448619	0.001360365	
UTR3	15:64449050-64449077	0.068614747	
ENST00000423270	ENSG00000181827 (RFX7)	hsa-miR-146a-5p	0.974012
UTR3	15:56382807-56382819	0.07030419	
UTR3	15:56388518-56388540	0.010916094	
ENST00000369962	ENSG00000085433 (WDR47)	hsa-miR-146a-5p	0.972803
UTR3	1:109513595-109513623	0.004430319	
UTR3	1:109512956-109512979	0.064738903	
ENST00000335877	ENSG00000125818 (PSMF1)	hsa-miR-146a-5p	0.972281
UTR3	20:1146213-1146237	0.003487842	
UTR3	20:1146841-1146856	0.003957562	
UTR3	20:1148219-1148234	0.032232406	
UTR3	20:1148898-1148915	0.029172153	
ENST00000354957	ENSG00000198093 (ZNF649)	hsa-miR-146a-5p	0.971925
UTR3	19:52393575-52393601	0.007636897	
UTR3	19:52393442-52393461	0.055621714	
UTR3	19:52393078-52393097	0.005376865	
ENST00000274747	ENSG00000124532 (MRS2)	hsa-miR-146a-5p	0.971303
UTR3	6:24424318-24424341	0.001332653	
UTR3	6:24424506-24424529	0.002817953	
UTR3	6:24424771-24424795	0.002510468	
UTR3	6:24425759-24425786	0.061605742	
ENST00000502732	ENSG00000143322 (ABL2)	hsa-miR-146a-5p	0.971234
UTR3	1:179076710-179076731	0.048888516	
UTR3	1:179075949-179075963	0.007319827	
UTR3	1:179075661-179075678	0.001967829	
UTR3	1:179075104-179075127	0.00470782	
UTR3	1:179069360-179069382	0.00166731	
UTR3	1:179068523-179068546	0.003675112	
ENST00000458646	ENSG00000077092 (RARB)	hsa-miR-146a-5p	0.971037
UTR3	3:25639229-25639249	0.068111561	
ENST00000318588	ENSG00000147044 (CASK)	hsa-miR-146a-5p	0.970985
UTR3	X:41379460-41379479	0.058467887	
UTR3	X:41378099-41378118	0.003460778	
UTR3	X:41377184-41377200	0.002033892	
UTR3	X:41376837-41376857	0.001339655	
UTR3	X:41375890-41375916	0.001271731	
UTR3	X:41375737-41375751	0.001507484	
ENST00000426654	ENSG00000102908 (NFAT5)	hsa-miR-146a-5p	0.970846
UTR3	16:69681433-69681447	0.005047255	
UTR3	16:69726570-69726592	0.00198929	
UTR3	16:69726951-69726979	0.002064471	
UTR3	16:69727036-69727056	0.001146055	
UTR3	16:69727866-69727885	0.018591107	
UTR3	16:69729133-69729156	0.002342665	

UTR3	16:69730904-69730924	0.003376283	
UTR3	16:69731625-69731647	0.001326825	
UTR3	16:69731973-69732000	0.002436764	
UTR3	16:69733289-69733307	0.002000631	
UTR3	16:69736424-69736438	0.0050489	
UTR3	16:69736425-69736452	0.001216539	
UTR3	16:69736719-69736746	0.002038788	
UTR3	16:69738193-69738219	0.012206378	
UTR3	16:69738383-69738405	0.007169048	
ENST00000531960	ENSG00000175664 (TEX26)	hsa-miR-146a-5p	0.969694
UTR3	13:31531165;31540359-31531166;31540383	0.052837536	
UTR3	13:31540503-31540527	0.006178791	
UTR3	13:31549093-31549118	0.008331502	
ENST00000451804	ENSG00000123843 (C4BPB)	hsa-miR-146a-5p	0.968445
UTR3	1:207269090-207269109	0.007509318	
UTR3	1:207269144-207269167	0.059348067	
UTR3	1:207268766-207268786	0.012248576	
ENST00000312960	ENSG00000181788 (SIAH2)	hsa-miR-146a-5p	0.967726
UTR3	3:150459129-150459146	0.066286302	
ENST00000522677	ENSG00000185697 (MYBL1)	hsa-miR-146a-5p	0.967611
UTR3	8:67476854-67476878	0.005352398	
UTR3	8:67476647-67476674	0.005901183	
UTR3	8:67475390-67475411	0.049925049	
UTR3	8:67475021-67475040	0.005047602	
ENST00000303697	ENSG00000170959 (DCDC1)	hsa-miR-146a-5p	0.967499
UTR3	11:30904673-30904687	0.00691475	
UTR3	11:30904662-30904687	0.004648429	
UTR3	11:30904216-30904239	0.009681834	
UTR3	11:30886688-30886708	0.001045666	
UTR3	11:30885758-30885774	0.02908735	
UTR3	11:30885305-30885318	0.005801897	
UTR3	11:30885258-30885285	0.008987909	
ENST00000394767	ENSG00000168772 (CXXC4)	hsa-miR-146a-5p	0.967096
UTR3	4:105392273-105392294	0.002979569	
UTR3	4:105390842-105390867	0.024136922	
UTR3	4:105389892-105389909	0.026498739	
UTR3	4:105389713-105389732	0.012344391	
ENST00000397358	ENSG00000147421 (HMBOX1)	hsa-miR-146a-5p	0.966784
UTR3	8:28908976-28908990	0.060699779	
UTR3	8:28909825-28909851	0.001942684	
UTR3	8:28910510-28910535	0.003158026	
ENST00000372491	ENSG00000188483 (IER5L)	hsa-miR-146a-5p	0.966423
UTR3	9:131938528-131938554	0.059644552	
UTR3	9:131938425-131938444	0.001707461	
UTR3	9:131937995-131938010	0.004265577	

ENST00000331070	ENSG00000183753 (BPY2)	hsa-miR-146a-5p	0.964886
UTR3	Y:25151353-25151381	0.014207961	
UTR3	Y:25151392-25151420	0.050651878	
ENST00000326972	ENSG00000180438 (TPRXL)	hsa-miR-146a-5p	0.964825
UTR3	3:14107339-14107362	0.064830578	
ENST00000316398	ENSG00000179071 (CCDC89)	hsa-miR-146a-5p	0.964203
UTR3	11:85395766-85395787	0.005468352	
UTR3	11:85395080-85395106	0.052315425	
UTR3	11:85394980-85394996	0.006749639	
ENST00000388975	ENSG00000154997 (SEPT14)	hsa-miR-146a-5p	0.963043
UTR3	7:55863516-55863531	0.05159133	
UTR3	7:55862002-55862024	0.003538476	
UTR3	7:55861575-55861589	0.004177488	
UTR3	7:55861511-55861533	0.004685542	
ENST00000369075	ENSG00000107651 (SEC23IP)	hsa-miR-146a-5p	0.962913
UTR3	10:121701122-121701141	0.063930929	
UTR3	10:121689879-121689907	0.017987386	
ENST00000540052	ENSG00000085185 (BCORL1)	hsa-miR-146a-5p	0.962412
UTR3	X:129190279-129190297	0.055280716	
UTR3	X:129191651-129191668	0.006202261	
UTR3	X:129191734-129191753	0.002222371	
ENST00000509876	ENSG00000118482 (PHF3)	hsa-miR-146a-5p	0.960985
UTR3	6:64388863-64388883	0.039464627	
UTR3	6:64401675-64401695	0.001936082	
UTR3	6:64416077-64416102	0.000959486	
UTR3	6:64422306-64422327	0.006576867	
UTR3	6:64422654-64422679	0.001684375	
UTR3	6:64424327-64424346	0.012450422	
ENST00000505939	ENSG00000131127 (ZNF141)	hsa-miR-146a-5p	0.959868
UTR3	4:377476-377500	0.055187942	
UTR3	4:377715-377735	0.007403891	
ENST00000534668	ENSG00000156738 (MS4A1)	hsa-miR-146a-5p	0.958265
UTR3	11:60237468-60237484	0.049682071	
UTR3	11:60237677-60237702	0.012025072	
UTR3	11:60234523;60235723-60234533;60235734	0.018023781	
ENST00000352690	ENSG00000153283 (CD96)	hsa-miR-146a-5p	0.957927
UTR3	3:111370018-111370039	0.001672588	
UTR3	3:111371104-111371119	0.060028197	
UTR3	3:111297993-111298019	0.007114806	
ENST00000421627	ENSG00000198010 (DLGAP2)	hsa-miR-146a-5p	0.957255
UTR3	8:1649837-1649862	0.013787664	
UTR3	8:1650195-1650211	0.008738723	

UTR3	8:1650468-1650493	0.002831756	
UTR3	8:1650961-1650988	0.016207131	
UTR3	8:1652162-1652188	0.011872031	
UTR3	8:1652728-1652741	0.002124407	
UTR3	8:1653852-1653870	0.001489885	
UTR3	8:1653942-1653968	0.000862255	
UTR3	8:1656043-1656069	0.003602114	
ENST00000382387	ENSG00000183784 (C9orf66)	hsa-miR-146a-5p	0.95659
UTR3	9:214327-214355	0.061252549	
ENST00000400477	ENSG00000198054 (DSCR8)	hsa-miR-146a-5p	0.95643
UTR3	21:39528509-39528533	0.061189496	
ENST00000371402	ENSG00000203989 (RHOXF2B)	hsa-miR-146a-5p	0.956011
UTR3	X:119206263-119206286	0.061025966	
ENST00000317775	ENSG00000089250 (NOS1)	hsa-miR-146a-5p	0.955282
UTR3	12:117646967-117646980	0.00312639	
UTR3	12:117646368-117646385	0.004048539	
UTR3	12:117646026-117646053	0.053226965	
UTR3	12:117728132-117728158	0.009613168	
UTR3	12:117698399;117701694-117698414;117701704	0.002600023	
UTR3	12:117685321;117691443-117685327;117691461	0.005807081	
ENST00000382392	ENSG00000183795 (BPY2B)	hsa-miR-146a-5p	0.955036
UTR3	Y:26785095-26785123	0.015053225	
UTR3	Y:26785134-26785162	0.045597779	
ENST00000335117	ENSG00000256229 (ZNF486)	hsa-miR-146a-5p	0.954494
UTR3	19:20309217-20309237	0.028528132	
UTR3	19:20309297-20309315	0.031606914	
UTR3	19:20296883;20307773-20296891;20307785	0.015189044	
ENST00000229201	ENSG00000111602 (TIMELESS)	hsa-miR-146a-5p	0.954485
UTR3	12:56811258-56811280	0.015340971	
UTR3	12:56810907-56810928	0.045101519	
ENST00000382287	ENSG00000185894 (BPY2C)	hsa-miR-146a-5p	0.953835
UTR3	Y:27177279-27177307	0.015001567	
UTR3	Y:27177240-27177268	0.045198006	
ENST00000409600	ENSG00000082153 (BZW1)	hsa-miR-146a-5p	0.953493
UTR3	2:201687952-201687977	0.004181261	
UTR3	2:201688497-201688518	0.055891885	
ENST00000427390	ENSG00000215405 (GOLGA6L6)	hsa-miR-146a-5p	0.953376
UTR3	15:20738351-20738366	0.010868893	
UTR3	15:20738100-20738119	0.006816508	
UTR3	15:20737326-20737340	0.042344835	

ENST00000418724	ENSG00000236104 (ZBTB22)	hsa-miR-146a-5p	0.95281
UTR3	6:33282289-33282316	0.059823276	
ENST00000427431	ENSG00000151718 (WWC2)	hsa-miR-146a-5p	0.95149
UTR3	4:184182481-184182497	0.001425662	
UTR3	4:184239294-184239312	0.057615359	
UTR3	4:184129276-184129301	0.011717538	
ENST00000304801	ENSG00000170950 (PGK2)	hsa-miR-146a-5p	0.949116
UTR3	6:49753444-49753468	0.058527915	
ENST00000373664	ENSG00000080839 (RBL1)	hsa-miR-146a-5p	0.947538
UTR3	20:35626118-35626134	0.041295883	
UTR3	20:35625851-35625867	0.00821791	
UTR3	20:35625375-35625398	0.003819123	
UTR3	20:35624861-35624889	0.003810607	
UTR3	20:35675482-35675504	0.012623947	
UTR3	20:35663864-35663884	0.010874714	
ENST00000371079	ENSG00000185483 (ROR1)	hsa-miR-146a-5p	0.944332
UTR3	1:64644620-64644632	0.032064351	
UTR3	1:64645289-64645309	0.007921362	
UTR3	1:64645731-64645756	0.016575153	
UTR3	1:64605822-64605843	0.010494527	
ENST00000584307	ENSG00000176105 (YES1)	hsa-miR-146a-5p	0.944138
UTR3	18:723949-723963	0.004999566	
UTR3	18:721758-721784	0.046396654	
UTR3	18:721714-721740	0.005520395	
ENST00000366518	ENSG00000162849 (KIF26B)	hsa-miR-146a-5p	0.943158
UTR3	1:245867013-245867033	0.001396727	
UTR3	1:245868440-245868461	0.003124656	
UTR3	1:245869105-245869124	0.000935753	
UTR3	1:245870404-245870417	0.002302977	
UTR3	1:245870795-245870806	0.001441941	
UTR3	1:245870899-245870913	0.000691472	
UTR3	1:245872529-245872557	0.046210219	
UTR3	1:245852103;245861408-245852109;245861414	0.012154847	
ENST00000424906	ENSG00000164053 (ATRIP)	hsa-miR-146a-5p	0.943077
UTR3	3:48493144-48493162	0.049888184	
UTR3	3:48498687-48498705	0.006702531	
ENST00000361621	ENSG00000123560 (PLP1)	hsa-miR-146a-5p	0.942031
UTR3	X:103045833-103045861	0.008635428	
UTR3	X:103046249-103046276	0.003698811	
UTR3	X:103047532-103047542	0.043940552	
ENST00000379982	ENSG00000164292 (RHOBTB3)	hsa-miR-146a-5p	0.941498
UTR3	5:95128996-95129019	0.016897218	
UTR3	5:95129373-95129389	0.033267653	
UTR3	5:95131310-95131323	0.005951106	
ENST00000519163	ENSG00000104218 (CSPP1)	hsa-miR-146a-5p	0.941364

UTR3	8:68026223-68026240	0.044563842	
UTR3	8:68026323-68026331	0.007979779	
UTR3	8:68028274-68028300	0.003532602	
ENST00000263773	ENSG00000109920 (FNBP4)	hsa-miR-146a-5p	0.940914
UTR3	11:47738744-47738763	0.006688765	
UTR3	11:47738457-47738476	0.048080432	
UTR3	11:47745613-47745627	0.014972637	
UTR3	11:47745605-47745627	0.008874075	
ENST00000354371	ENSG00000198612 (COPS8)	hsa-miR-146a-5p	0.939224
UTR3	2:238006581-238006589	0.041859037	
UTR3	2:238008654-238008673	0.013594174	
ENST00000395375	ENSG00000198039 (ZNF273)	hsa-miR-146a-5p	0.939075
UTR3	7:64378662;64388032-64378670;64388044	0.014125925	
UTR3	7:64388261-64388279	0.005364395	
UTR3	7:64388429-64388447	0.030797464	
UTR3	7:64388674-64388699	0.003209394	
UTR3	7:64389010-64389035	0.001913377	
ENST00000374507	ENSG00000204193 (TXNDC8)	hsa-miR-146a-5p	0.936609
UTR3	9:113085880-113085891	0.008513856	
UTR3	9:113085408-113085427	0.004058142	
UTR3	9:113085220-113085246	0.004977702	
UTR3	9:113084886-113084900	0.037169489	
ENST00000554546	ENSG00000133961 (NUMB)	hsa-miR-146a-5p	0.935898
UTR3	14:73742656-73742664	0.053802238	
UTR3	14:73743841-73743858	0.013267449	
ENST00000340749	ENSG00000173262 (SLC2A14)	hsa-miR-146a-5p	0.935551
UTR3	12:7966791-7966807	0.003726273	
UTR3	12:7966171-7966199	0.005629484	
UTR3	12:7965353-7965377	0.014027086	
UTR3	12:7965346-7965371	0.024269016	
UTR3	12:7965199-7965224	0.006778283	
ENST00000374864	ENSG00000078747 (ITCH)	hsa-miR-146a-5p	0.934822
UTR3	20:33095721-33095734	0.017221974	
UTR3	20:33096061-33096079	0.002212767	
UTR3	20:33096918-33096938	0.001713671	
UTR3	20:33096979-33096996	0.001650712	
UTR3	20:33097624-33097643	0.001772097	
UTR3	20:33098706-33098730	0.029662258	
ENST00000396847	ENSG00000132256 (TRIM5)	hsa-miR-146a-5p	0.934152
UTR3	11:5686140-5686153	0.003644808	
UTR3	11:5686124-5686152	0.002112568	
UTR3	11:5686109-5686127	0.001897926	
UTR3	11:5685000-5685017	0.046399409	

ENST00000460236	ENSG00000129292 (PHF20L1)	hsa-miR-146a-5p	0.932921
UTR3	8:133844538-133844560	0.009228918	
UTR3	8:133851700-133851725	0.00469759	
UTR3	8:133858432-133858452	0.010108046	
UTR3	8:133858614-133858634	0.00158917	
UTR3	8:133858696-133858718	0.00444692	
UTR3	8:133858921-133858932	0.003045201	
UTR3	8:133859639-133859663	0.02061466	
ENST00000302350	ENSG00000168564 (CDKN2AIP)	hsa-miR-146a-5p	0.931422
UTR3	4:184368018-184368045	0.035372107	
UTR3	4:184368429-184368446	0.005628986	
UTR3	4:184368700-184368718	0.012341869	
ENST00000316529	ENSG00000180919 (OR56B4)	hsa-miR-146a-5p	0.93079
UTR3	11:6130007-6130024	0.053181943	
ENST00000366113	ENSG00000161570 (CCL5)	hsa-miR-146a-5p	0.930675
UTR3	17:34198508-34198522	0.053152711	
ENST00000450366	ENSG00000115365 (LANCL1)	hsa-miR-146a-5p	0.930108
UTR3	2:211297293-211297318	0.009482455	
UTR3	2:211295997-211296012	0.042959185	
UTR3	2:211336783;211341040-211336800;211341047	0.009201432	
ENST00000366918	ENSG00000116141 (MARK1)	hsa-miR-146a-5p	0.929721
UTR3	1:220837230-220837245	0.048516216	
UTR3	1:220837502-220837527	0.004396329	
ENST00000366997	ENSG00000123684 (LPGAT1)	hsa-miR-146a-5p	0.928543
UTR3	1:211922841-211922862	0.010840692	
UTR3	1:211922561-211922589	0.003129534	
UTR3	1:211922433-211922452	0.002264568	
UTR3	1:211922178-211922202	0.00623912	
UTR3	1:211920909-211920935	0.010475883	
UTR3	1:211920631-211920657	0.002319689	
UTR3	1:211920563-211920580	0.001169805	
UTR3	1:211919651-211919672	0.006850212	
UTR3	1:211918391-211918414	0.002940592	
UTR3	1:211917953-211917975	0.001648773	
UTR3	1:211917530-211917556	0.004741397	
ENST00000544604	ENSG00000183579 (ZNRF3)	hsa-miR-146a-5p	0.928019
UTR3	22:29449881-29449904	0.010205315	
UTR3	22:29451365-29451388	0.001173897	
UTR3	22:29451526-29451544	0.000662302	
UTR3	22:29452413-29452431	0.001380347	
UTR3	22:29453216-29453236	0.039069593	
ENST00000260113	ENSG00000137558 (PI15)	hsa-miR-146a-5p	0.925588
UTR3	8:75761935-75761959	0.003424527	

UTR3	8:75762125-75762147	0.042990623	
UTR3	8:75762409-75762428	0.002062434	
UTR3	8:75764483-75764508	0.001569849	
UTR3	8:75764893-75764912	0.001858343	
ENST00000370251	ENSG00000063587 (ZNF275)	hsa-miR-146a-5p	0.925555
UTR3	X:152614970-152614987	0.002206311	
UTR3	X:152616122-152616140	0.000940463	
UTR3	X:152616680-152616692	0.001484893	
UTR3	X:152617754-152617778	0.002756319	
UTR3	X:152617768-152617785	0.002120668	
UTR3	X:152617911-152617921	0.003238824	
UTR3	X:152617989-152618017	0.034558842	
UTR3	X:152618134-152618160	0.004591758	
ENST00000554234	ENSG00000166866 (MYO1A)	hsa-miR-146a-5p	0.924313
UTR3	12:57423326-57423342	0.005336016	
UTR3	12:57422659;57422916-57422665;57422928	0.045615654	
UTR3	12:57431812-57431836	0.009380617	
ENST00000369535	ENSG00000213281 (NRAS)	hsa-miR-146a-5p	0.923161
UTR3	1:115250536-115250554	0.046811517	
UTR3	1:115249522-115249540	0.001726009	
UTR3	1:115249198-115249223	0.001529511	
UTR3	1:115248973-115248988	0.001271336	
ENST00000321437	ENSG00000166128 (RAB8B)	hsa-miR-146a-5p	0.922907
UTR3	15:63557120-63557145	0.002114001	
UTR3	15:63559104-63559125	0.049165886	
ENST00000429071	ENSG00000006652 (IFRD1)	hsa-miR-146a-5p	0.922065
UTR3	7:112097939-112097954	0.005885721	
UTR3	7:112098266-112098288	0.045201794	
ENST00000261177	ENSG00000110900 (TSPAN11)	hsa-miR-146a-5p	0.92173
UTR3	12:31145155-31145171	0.001666292	
UTR3	12:31145630-31145647	0.002280426	
UTR3	12:31145897-31145909	0.00371389	
UTR3	12:31146440-31146453	0.000963115	
UTR3	12:31146643-31146661	0.002592661	
UTR3	12:31147861-31147882	0.006836254	
UTR3	12:31148669-31148681	0.007370486	
UTR3	12:31148740-31148757	0.001943008	
UTR3	12:31149417-31149438	0.023064694	
UTR3	12:31136085;31144791-31136085;31144809	0.008007945	
ENST00000336125	ENSG00000185829 (ARL17A)	hsa-miR-146a-5p	0.921515
UTR3	17:44634232-44634259	0.026691515	
UTR3	17:44633999-44634025	0.008348582	
UTR3	17:44632949-44632971	0.000886323	
UTR3	17:44632032-44632059	0.006943577	

UTR3	17:44631869-44631884	0.002938094	
UTR3	17:44631701-44631721	0.005154619	
ENST00000318426	ENSG00000180861 (C12orf36)	hsa-miR-146a-5p	0.921444
UTR3	12:13525962-13525987	0.005662039	
UTR3	12:13525427-13525448	0.045284717	
ENST00000265074	ENSG00000113389 (NPR3)	hsa-miR-146a-5p	0.920783
UTR3	5:32788326-32788341	0.005063542	
UTR3	5:32789259-32789282	0.001856385	
UTR3	5:32790776-32790803	0.004052495	
UTR3	5:32791778-32791803	0.039825615	
ENST00000424319	ENSG00000086991 (NOX4)	hsa-miR-146a-5p	0.920029
UTR3	11:89059686-89059705	0.050629889	
ENST00000476605	ENSG00000107745 (MICU1)	hsa-miR-146a-5p	0.919173
UTR3	10:74127739-74127766	0.021375193	
UTR3	10:74127607-74127631	0.029065472	
ENST00000360669	ENSG00000170074 (FAM153A)	hsa-miR-146a-5p	0.918663
UTR3	5:177144235-177144262	0.001179086	
UTR3	5:177143395-177143415	0.031010738	
UTR3	5:177139381-177139401	0.013043851	
UTR3	5:177139117-177139140	0.005095101	
ENST00000299550	ENSG00000166436 (TRIM66)	hsa-miR-146a-5p	0.918273
UTR3	11:8638466-8638486	0.01929223	
UTR3	11:8638206-8638226	0.005438195	
UTR3	11:8637527-8637540	0.003362398	
UTR3	11:8637227-8637245	0.00153674	
UTR3	11:8635936-8635951	0.001573441	
UTR3	11:8635132-8635150	0.005661055	
UTR3	11:8634841-8634856	0.001369855	
UTR3	11:8634624-8634640	0.009883872	
UTR3	11:8667374-8667389	0.022137617	
UTR3	11:8661979-8662004	0.002903856	
ENST00000335154	ENSG00000161791 (FMNL3)	hsa-miR-146a-5p	0.917803
UTR3	12:50037697-50037717	0.002261838	
UTR3	12:50037210-50037232	0.004385479	
UTR3	12:50036966-50036985	0.001008182	
UTR3	12:50036618-50036642	0.00119013	
UTR3	12:50034965-50034986	0.001897461	
UTR3	12:50034472-50034492	0.002054045	
UTR3	12:50034022-50034042	0.001624567	
UTR3	12:50033814-50033834	0.001833513	
UTR3	12:50031814-50031836	0.033886252	
ENST00000442398	ENSG00000168014 (C2CD3)	hsa-miR-146a-5p	0.916923
UTR3	11:73745278-73745305	0.04254337	
UTR3	11:73744766-73744782	0.004660191	
UTR3	11:73724449-73724477	0.002354983	

UTR3	11:73796788-73796800	0.00509441	
ENST00000408967	ENSG00000221870 (TMEM257)	hsa-miR-146a-5p	0.916465
UTR3	X:144909686-144909709	0.008712472	
UTR3	X:144910653-144910680	0.005533041	
UTR3	X:144911080-144911102	0.003643757	
UTR3	X:144911277-144911293	0.031964498	
ENST00000395407	ENSG00000137819 (PAQR5)	hsa-miR-146a-5p	0.916403
UTR3	15:69696768-69696789	0.029856955	
UTR3	15:69697095-69697121	0.006735723	
UTR3	15:69697431-69697449	0.004094715	
UTR3	15:69698728-69698753	0.002134986	
UTR3	15:69698851-69698868	0.00090138	
UTR3	15:69699893-69699907	0.006116821	
ENST00000524849	ENSG00000173653 (RCE1)	hsa-miR-146a-5p	0.916164
UTR3	11:66612705;66612971-66612708;66612983	0.041078409	
UTR3	11:66613919-66613940	0.00871117	
ENST00000394323	ENSG00000213462 (ERV3-1)	hsa-miR-146a-5p	0.914867
UTR3	7:64451557-64451579	0.04951551	
ENST00000362063	ENSG00000198740 (ZNF652)	hsa-miR-146a-5p	0.9135
UTR3	17:47375686-47375712	0.023335745	
UTR3	17:47373732-47373758	0.001634123	
UTR3	17:47372307-47372323	0.004963695	
UTR3	17:47372208-47372230	0.014409093	
UTR3	17:47371929-47371956	0.004887987	
ENST00000333926	ENSG00000122873 (CISD1)	hsa-miR-146a-5p	0.913124
UTR3	10:60047725-60047744	0.007840666	
UTR3	10:60048611-60048639	0.003933345	
UTR3	10:60049120-60049138	0.03737915	
ENST00000398146	ENSG00000185304 (RGPD2)	hsa-miR-146a-5p	0.913064
UTR3	2:88055771-88055793	0.049140704	
ENST00000327827	ENSG00000182831 (C16orf72)	hsa-miR-146a-5p	0.912795
UTR3	16:9211053-9211075	0.008908725	
UTR3	16:9211896-9211916	0.03717986	
UTR3	16:9215427-9215444	0.002996832	
ENST00000415914	ENSG00000177683 (THAP5)	hsa-miR-146a-5p	0.912487
UTR3	7:108204540-108204557	0.049022337	
ENST00000450908	ENSG00000231880 (KBTBD4)	hsa-miR-146a-5p	0.910796
UTR3	11:47599497-47599516	0.048679269	
ENST00000436010	ENSG00000169855 (ROBO1)	hsa-miR-146a-5p	0.910675
UTR3	3:78646395-78646421	0.048654833	
ENST00000394670	ENSG00000010244 (ZNF207)	hsa-miR-146a-5p	0.910317
UTR3	17:30696800-30696825	0.01318423	
UTR3	17:30698222-30698243	0.001600884	

UTR3	17:30699372-30699383	0.001199639	
UTR3	17:30702968-30702979	0.001943835	
UTR3	17:30703542-30703555	0.001183252	
UTR3	17:30704495-30704515	0.001390638	
UTR3	17:30705340-30705354	0.001717228	
UTR3	17:30708425-30708448	0.026363328	
ENST00000306434	ENSG00000168906 (MAT2A)	hsa-miR-146a-5p	0.910234
UTR3	2:85772336-85772353	0.048566439	
ENST00000300087	ENSG00000166847 (DCTN5)	hsa-miR-146a-5p	0.910023
UTR3	16:23678593-23678608	0.007561034	
UTR3	16:23680144-23680172	0.021977088	
UTR3	16:23680764-23680784	0.017467979	
UTR3	16:23654313-23654336	0.016710802	
ENST00000379638	ENSG00000129625 (REEP5)	hsa-miR-146a-5p	0.908794
UTR3	5:112212087-112212099	0.048280475	
ENST00000375255	ENSG00000115827 (DCAF17)	hsa-miR-146a-5p	0.908514
UTR3	2:172338227-172338243	0.003050827	
UTR3	2:172338359-172338375	0.009737604	
UTR3	2:172340399-172340424	0.014980952	
UTR3	2:172341266-172341292	0.017594567	
UTR3	2:172333382-172333408	0.028713584	
ENST00000381605	ENSG00000101307 (SIRPB1)	hsa-miR-146a-5p	0.907723
UTR3	20:1545749-1545769	0.004855969	
UTR3	20:1544460-1544479	0.027308652	
UTR3	20:1544439-1544463	0.015905696	
ENST00000316433	ENSG00000171817 (ZNF540)	hsa-miR-146a-5p	0.907103
UTR3	19:38104290-38104304	0.046686974	
UTR3	19:38103603-38103623	0.013643207	
ENST00000453634	ENSG00000254413 (CHKB-CPT1B)	hsa-miR-146a-5p	0.907012
UTR3	22:51008017-51008031	0.047932122	
ENST00000569722	ENSG00000267127 (RP11-795F19.5)	hsa-miR-146a-5p	0.905808
UTR3	18:77829827-77829842	0.011041003	
UTR3	18:77854277-77854303	0.002062248	
UTR3	18:77854744-77854767	0.006212056	
UTR3	18:77854764-77854782	0.028384965	
ENST00000326505	ENSG00000176597 (B3GNT5)	hsa-miR-146a-5p	0.905589
UTR3	3:182988964-182988988	0.04534622	
UTR3	3:182990217-182990240	0.002311973	
ENST00000306156	ENSG00000171132 (PRKCE)	hsa-miR-146a-5p	0.905578
UTR3	2:46413327-46413339	0.002120682	
UTR3	2:46413959-46413981	0.018235749	
UTR3	2:46414504-46414528	0.021886314	
UTR3	2:46414757-46414780	0.005413373	

ENST00000326080	ENSG00000170271 (FAXDC2)	hsa-miR-146a-5p	0.90524
UTR3	5:154198689-154198705	0.029777327	
UTR3	5:154198409-154198437	0.017814433	
ENST00000539768	ENSG00000177058 (SLC38A9)	hsa-miR-146a-5p	0.903343
UTR3	5:54931034-54931054	0.032035071	
UTR3	5:54930538-54930564	0.009853264	
UTR3	5:54922107-54922116	0.005345041	
ENST00000398532	ENSG00000188895 (MSL1)	hsa-miR-146a-5p	0.902927
UTR3	17:38290995-38291015	0.003315317	
UTR3	17:38292279-38292300	0.006035168	
UTR3	17:38292586-38292603	0.007284066	
UTR3	17:38292805-38292819	0.03052112	
ENST00000263816	ENSG00000081479 (LRP2)	hsa-miR-146a-5p	0.902583
UTR3	2:169984508-169984532	0.002551397	
UTR3	2:169983727-169983753	0.042135638	
UTR3	2:170136060-170136075	0.01078508	
UTR3	2:170033059-170033081	0.006143526	
UTR3	2:170011040-170011058	0.006353665	
ENST00000491143	ENSG00000119547 (ONECUT2)	hsa-miR-146a-5p	0.901601
UTR3	18:55144188-55144204	0.003519184	
UTR3	18:55145988-55146008	0.003749262	
UTR3	18:55146219-55146241	0.002982475	
UTR3	18:55146682-55146703	0.00150389	
UTR3	18:55147026-55147044	0.001423307	
UTR3	18:55147099-55147115	0.00055766	
UTR3	18:55148184-55148203	0.006033746	
UTR3	18:55149145-55149168	0.000613774	
UTR3	18:55149622-55149648	0.00183181	
UTR3	18:55149927-55149938	0.004258154	
UTR3	18:55153730-55153748	0.003697044	
UTR3	18:55157187-55157201	0.001715215	
UTR3	18:55157278-55157300	0.004850741	
UTR3	18:55157307-55157333	0.000834621	
UTR3	18:55157510-55157529	0.00854619	
UTR3	18:55157541-55157559	0.000792845	
ENST00000338957	ENSG00000102053 (ZC3H12B)	hsa-miR-146a-5p	0.901432
UTR3	X:64726080-64726102	0.005939501	
UTR3	X:64727241-64727268	0.040939248	
ENST00000392288	ENSG00000196268 (ZNF493)	hsa-miR-146a-5p	0.901147
UTR3	19:21607918-21607943	0.00585188	
UTR3	19:21608004-21608027	0.038476993	
UTR3	19:21588650;21605715-21588658;21605727	0.023740422	

ENST00000447404	ENSG00000165209 (STRBP)	hsa-miR-146a-5p	0.900243
UTR3	9:125887711-125887732	0.026839795	
UTR3	9:125885022-125885047	0.003336137	
UTR3	9:125884552-125884571	0.008603742	
UTR3	9:125884008-125884035	0.007881634	
ENST00000591537	ENSG00000196263 (ZNF471)	hsa-miR-146a-5p	0.899449
UTR3	19:57041044-57041067	0.023458668	
UTR3	19:57041301-57041321	0.023058558	
ENST00000551568	ENSG00000135678 (CPM)	hsa-miR-146a-5p	0.898884
UTR3	12:69249441-69249463	0.03162357	
UTR3	12:69247760-69247787	0.001653194	
UTR3	12:69247753-69247773	0.001957846	
UTR3	12:69245942-69245968	0.003060302	
UTR3	12:69244979-69245002	0.008120599	
ENST00000326958	ENSG00000181495 (AC026703.1)	hsa-miR-146a-5p	0.898505
UTR3	5:32790776-32790803	0.004539658	
UTR3	5:32791778-32791803	0.04180779	
ENST00000397121	ENSG00000196109 (ZNF676)	hsa-miR-146a-5p	0.898419
UTR3	19:22362713-22362732	0.003751524	
UTR3	19:22362378-22362404	0.033317623	
UTR3	19:22364376;22375818-22364388;22375826	0.021221949	
UTR3	19:22363889-22363907	0.029598937	
UTR3	19:22363049-22363074	0.013451312	
ENST00000449592	ENSG00000188735 (TMEM120B)	hsa-miR-146a-5p	0.897705
UTR3	12:122214126-122214152	0.038357299	
UTR3	12:122216502-122216515	0.007847142	
ENST00000595401	ENSG00000118620 (ZNF430)	hsa-miR-146a-5p	0.897022
UTR3	19:21217638-21217664	0.023164185	
UTR3	19:21218082-21218093	0.022919049	
ENST00000379882	ENSG00000107201 (DDX58)	hsa-miR-146a-5p	0.896644
UTR3	9:32456226-32456244	0.003854819	
UTR3	9:32456205-32456218	0.001906753	
UTR3	9:32456145-32456171	0.003219557	
UTR3	9:32455476-32455501	0.037035461	
ENST00000355585	ENSG00000078269 (SYNJ2)	hsa-miR-146a-5p	0.8965
UTR3	6:158517676-158517701	0.02126433	
UTR3	6:158518254-158518271	0.002872755	
UTR3	6:158519169-158519188	0.001796125	
UTR3	6:158519527-158519553	0.012844049	
UTR3	6:158519773-158519797	0.003605396	
UTR3	6:158519873-158519896	0.003608452	
ENST00000336078	ENSG00000074657 (ZNF532)	hsa-miR-146a-5p	0.896344
UTR3	18:56652900-56652915	0.014475897	
UTR3	18:56653289-56653316	0.030729767	

UTR3	18:56606724-56606742	0.007542597	
ENST00000359856	ENSG00000162704 (ARPC5)	hsa-miR-146a-5p	0.896009
UTR3	1:183595583-183595599	0.045904877	
ENST00000375537	ENSG00000204371 (EHMT2)	hsa-miR-146a-5p	0.894287
UTR3	6:31847560-31847585	0.045605021	
ENST00000424486	ENSG00000166292 (TMEM100)	hsa-miR-146a-5p	0.894059
UTR3	17:53797191-53797215	0.045565667	
ENST00000313732	ENSG00000153786 (ZDHHC7)	hsa-miR-146a-5p	0.893061
UTR3	16:85008068-85008076	0.044685195	
UTR3	16:85009955-85009982	0.006863536	
ENST00000483536	ENSG00000139910 (NOVA1)	hsa-miR-146a-5p	0.892508
UTR3	14:26916955-26916970	0.00399913	
UTR3	14:26916559-26916568	0.003843866	
UTR3	14:26916476-26916503	0.02011346	
UTR3	14:26915325-26915351	0.010102665	
UTR3	14:26913829-26913849	0.003147154	
UTR3	14:27064730-27064754	0.033568342	
ENST00000268182	ENSG00000140575 (IQGAP1)	hsa-miR-146a-5p	0.89201
UTR3	15:91045257-91045285	0.045215006	
ENST00000491718	ENSG00000090905 (TNRC6A)	hsa-miR-146a-5p	0.891757
UTR3	16:24801462-24801480	0.004358792	
UTR3	16:24815565-24815588	0.002044444	
UTR3	16:24817017-24817038	0.002737361	
UTR3	16:24835516-24835539	0.003755898	
UTR3	16:24835837-24835860	0.005342668	
UTR3	16:24836045-24836069	0.0136901	
UTR3	16:24836846-24836864	0.013242877	
ENST00000377200	ENSG00000188647 (PTAR1)	hsa-miR-146a-5p	0.891389
UTR3	9:72333010-72333037	0.005492955	
UTR3	9:72332979-72332991	0.003742988	
UTR3	9:72332509-72332524	0.008094394	
UTR3	9:72329658-72329675	0.012031297	
UTR3	9:72329117-72329143	0.00251719	
UTR3	9:72326404-72326423	0.001804407	
UTR3	9:72325873-72325899	0.011426724	
ENST00000486333	ENSG00000225932 (CTAGE4)	hsa-miR-146a-5p	0.890329
UTR3	7:143883082-143883105	0.017471703	
UTR3	7:143883118-143883135	0.027460037	
ENST00000487179	ENSG00000244693 (CTAGE8)	hsa-miR-146a-5p	0.890214
UTR3	7:143963835-143963858	0.017426257	
UTR3	7:143963805-143963822	0.027486165	

ENST00000409085	ENSG00000115977 (AAK1)	hsa-miR-146a-5p	0.88979
UTR3	2:69702769-69702795	0.007645494	
UTR3	2:69702291-69702312	0.00306278	
UTR3	2:69699316-69699334	0.001339128	
UTR3	2:69699055-69699080	0.001739327	
UTR3	2:69697383-69697409	0.0014199	
UTR3	2:69696254-69696277	0.001304689	
UTR3	2:69696000-69696015	0.002036837	
UTR3	2:69695887-69695911	0.026293546	
ENST00000237937	ENSG00000107372 (ZFAND5)	hsa-miR-146a-5p	0.88909
UTR3	9:74970770-74970795	0.011220361	
UTR3	9:74966898-74966919	0.033504992	
ENST00000296028	ENSG00000163736 (PPBP)	hsa-miR-146a-5p	0.888667
UTR3	4:74852912-74852935	0.044655385	
ENST00000532519	ENSG00000149428 (HYOU1)	hsa-miR-146a-5p	0.888643
UTR3	11:118925757;118925897-118925772;118925901	0.023742511	
UTR3	11:118925729-118925757	0.017257334	
UTR3	11:118925714-118925739	0.003651588	
ENST00000398417	ENSG00000141646 (SMAD4)	hsa-miR-146a-5p	0.88844
UTR3	18:48605219-48605234	0.042237913	
UTR3	18:48609915-48609940	0.00237992	
ENST00000376351	ENSG00000151025 (GPR158)	hsa-miR-146a-5p	0.888382
UTR3	10:25889496-25889516	0.003005168	
UTR3	10:25890087-25890113	0.00275966	
UTR3	10:25890697-25890713	0.005908377	
UTR3	10:25890869-25890894	0.025376522	
UTR3	10:25891023-25891036	0.006377155	
UTR3	10:25887823-25887840	0.010736726	
ENST00000409558	ENSG00000143952 (VPS54)	hsa-miR-146a-5p	0.88802
UTR3	2:64119695-64119713	0.044548806	
ENST00000217130	ENSG00000196700 (ZNF512B)	hsa-miR-146a-5p	0.887447
UTR3	20:62591149-62591175	0.015982295	
UTR3	20:62588308-62588331	0.028472553	
ENST00000434009	ENSG00000009413 (REV3L)	hsa-miR-146a-5p	0.886964
UTR3	6:111793223-111793241	0.007616599	
UTR3	6:111737582-111737603	0.005373804	
UTR3	6:111710332-111710358	0.003835714	
UTR3	6:111709223-111709249	0.003202364	
UTR3	6:111709069-111709081	0.003993808	
UTR3	6:111697636-111697658	0.002273759	
UTR3	6:111695228-111695248	0.00264594	
UTR3	6:111695035-111695052	0.002568533	
UTR3	6:111694760-111694772	0.001761053	
UTR3	6:111688770-111688794	0.001953287	

UTR3	6:111685138-111685160	0.002288742	
UTR3	6:111654417-111654443	0.001460498	
UTR3	6:111632447-111632460	0.003524855	
UTR3	6:111631101-111631122	0.00187689	
ENST00000249064	ENSG00000159873 (CCDC117)	hsa-miR-146a-5p	0.886368
UTR3	22:29184985-29185006	0.044278921	
ENST00000335251	ENSG00000164066 (INTU)	hsa-miR-146a-5p	0.885591
UTR3	4:128637931-128637959	0.00461416	
UTR3	4:128638344-128638357	0.00488379	
UTR3	4:128639350-128639370	0.002532676	
UTR3	4:128639403-128639422	0.000519055	
UTR3	4:128639456-128639475	0.002227915	
UTR3	4:128639816-128639836	0.005405006	
UTR3	4:128642615-128642641	0.000779584	
UTR3	4:128642792-128642804	0.006355016	
UTR3	4:128642925-128642951	0.015527957	
UTR3	4:128643219-128643237	0.001308123	
ENST00000376773	ENSG00000204619 (PPP1R11)	hsa-miR-146a-5p	0.885378
UTR3	6:30037325-30037348	0.025670123	
UTR3	6:30037614-30037624	0.002681037	
UTR3	6:30037941-30037960	0.015767847	
ENST00000541208	ENSG00000182983 (ZNF662)	hsa-miR-146a-5p	0.883529
UTR3	3:42957613-42957630	0.004358254	
UTR3	3:42958676-42958701	0.001037858	
UTR3	3:42959183-42959209	0.001931125	
UTR3	3:42959920-42959943	0.00118073	
UTR3	3:42960751-42960764	0.035315211	
ENST00000608642	ENSG00000133424 (LARGE)	hsa-miR-146a-5p	0.883427
UTR3	22:33562218-33562242	0.005438238	
UTR3	22:33561426-33561447	0.00204529	
UTR3	22:33560358-33560379	0.002123222	
UTR3	22:33559949-33559964	0.000860168	
UTR3	22:33558499-33558512	0.00934069	
UTR3	22:33558373-33558396	0.02399942	
ENST00000240093	ENSG00000104290 (FZD3)	hsa-miR-146a-5p	0.883314
UTR3	8:28420938-28420959	0.005381406	
UTR3	8:28421095-28421112	0.003649304	
UTR3	8:28422617-28422627	0.001436096	
UTR3	8:28423412-28423436	0.005613947	
UTR3	8:28426923-28426946	0.001676104	
UTR3	8:28426993-28427015	0.003267751	
UTR3	8:28427337-28427354	0.003280566	
UTR3	8:28428185-28428206	0.002777862	
UTR3	8:28428204-28428225	0.000592374	
UTR3	8:28428488-28428502	0.001073201	

UTR3	8:28428566-28428583	0.00165531	
UTR3	8:28429191-28429209	0.002333534	
UTR3	8:28429650-28429670	0.001477027	
UTR3	8:28429843-28429859	0.001904996	
UTR3	8:28431166-28431180	0.003786649	
UTR3	8:28420388-28420407	0.030120813	
ENST00000361320	ENSG00000198739 (LRRTM3)	hsa-miR-146a-5p	0.882552
UTR3	10:68858248-68858256	0.043668663	
ENST00000367667	ENSG00000143207 (RFWD2)	hsa-miR-146a-5p	0.882188
UTR3	1:176085761-176085784	0.040439318	
UTR3	1:175956150-175956167	0.003172006	
ENST00000318887	ENSG00000179021 (C3orf38)	hsa-miR-146a-5p	0.882017
UTR3	3:88206295-88206309	0.040289984	
UTR3	3:88206476-88206500	0.003294384	
ENST00000295461	ENSG00000163293 (NIPAL1)	hsa-miR-146a-5p	0.881988
UTR3	4:48038629-48038643	0.004049145	
UTR3	4:48039236-48039264	0.002710576	
UTR3	4:48039901-48039916	0.002117295	
UTR3	4:48041848-48041875	0.005744103	
UTR3	4:48042049-48042063	0.02698864	
UTR3	4:48035069-48035096	0.016447884	
ENST00000538204	ENSG00000076770 (MBNL3)	hsa-miR-146a-5p	0.8819
UTR3	X:131512813-131512832	0.003660399	
UTR3	X:131510555-131510567	0.00127024	
UTR3	X:131507596-131507615	0.022315026	
UTR3	X:131505209-131505226	0.003127786	
UTR3	X:131504841-131504867	0.001775066	
UTR3	X:131504727-131504743	0.000713365	
UTR3	X:131503558-131503585	0.010704179	
ENST00000581347	ENSG00000206432 (TMEM200C)	hsa-miR-146a-5p	0.880879
UTR3	18:5889722-5889748	0.033788632	
UTR3	18:5889178-5889194	0.002272513	
UTR3	18:5888359-5888375	0.001716028	
UTR3	18:5882620-5882641	0.005629241	
ENST00000331343	ENSG00000186866 (POFUT2)	hsa-miR-146a-5p	0.880671
UTR3	21:46687294-46687309	0.033412011	
UTR3	21:46686477-46686505	0.001633298	
UTR3	21:46685616-46685639	0.003438071	
UTR3	21:46685583-46685604	0.000804293	
UTR3	21:46684063-46684081	0.004086357	
ENST00000371655	ENSG00000169213 (RAB3B)	hsa-miR-146a-5p	0.880267
UTR3	1:52385438-52385452	0.004249603	
UTR3	1:52385176-52385199	0.003765413	

UTR3	1:52380232-52380257	0.002078708	
UTR3	1:52378822-52378846	0.001544235	
UTR3	1:52378484-52378510	0.001376216	
UTR3	1:52377133-52377149	0.007168358	
UTR3	1:52377124-52377149	0.00065915	
UTR3	1:52376920-52376930	0.00179184	
UTR3	1:52376902-52376923	0.003109456	
UTR3	1:52376756-52376773	0.000882303	
UTR3	1:52375950-52375975	0.001363112	
UTR3	1:52373898-52373922	0.011145842	
UTR3	1:52373852-52373865	0.004177053	
ENST00000368069	ENSG00000122218 (COPA)	hsa-miR-146a-5p	0.880251
UTR3	1:160259756-160259781	0.004712116	
UTR3	1:160259103-160259114	0.030142655	
UTR3	1:160259094-160259114	0.008454108	
ENST00000310823	ENSG00000151694 (ADAM17)	hsa-miR-146a-5p	0.879744
UTR3	2:9628760-9628782	0.043230345	
ENST00000395745	ENSG00000132153 (DHX30)	hsa-miR-146a-5p	0.879602
UTR3	3:47868867-47868884	0.010499306	
UTR3	3:47890563;47890682-47890569;47890701	0.032709078	
ENST00000339140	ENSG00000189299 (FOXR2)	hsa-miR-146a-5p	0.879586
UTR3	X:55651752-55651771	0.043206027	
ENST00000309395	ENSG00000173542 (MOB1B)	hsa-miR-146a-5p	0.879435
UTR3	4:71849989-71850014	0.00375675	
UTR3	4:71851570-71851590	0.001170711	
UTR3	4:71851860-71851876	0.002008575	
UTR3	4:71852727-71852748	0.023385586	
UTR3	4:71853025-71853045	0.011921542	
UTR3	4:71824633-71824647	0.008073214	
ENST00000328252	ENSG00000182752 (PAPPA)	hsa-miR-146a-5p	0.879265
UTR3	9:119160213-119160234	0.004440191	
UTR3	9:119164075-119164103	0.024064301	
UTR3	9:119164424-119164449	0.014652078	
ENST00000608933	ENSG00000163644 (PPM1K)	hsa-miR-146a-5p	0.879089
UTR3	4:89183238-89183265	0.010689188	
UTR3	4:89182614-89182626	0.019410223	
UTR3	4:89180468-89180488	0.001313765	
UTR3	4:89179317-89179329	0.011716216	
ENST00000380317	ENSG00000099385 (BCL7C)	hsa-miR-146a-5p	0.878849
UTR3	16:30845329-30845350	0.035413459	
UTR3	16:30845301-30845322	0.004898895	
UTR3	16:30844989-30845017	0.00278017	
ENST00000460930	ENSG00000203690 (TCP10)	hsa-miR-146a-5p	0.878305
UTR3	6:167787895-167787923	0.001763689	
UTR3	6:167787702-167787713	0.003032952	

UTR3	6:167787514-167787535	0.038212567	
ENST00000357758	ENSG00000100523 (DDHD1)	hsa-miR-146a-5p	0.878237
UTR3	14:53512826-53512840	0.005164165	
UTR3	14:53512326-53512336	0.006423995	
UTR3	14:53511768-53511787	0.002381937	
UTR3	14:53511282-53511300	0.027661878	
UTR3	14:53521304-53521326	0.011422172	
ENST00000194672	ENSG00000082269 (FAM135A)	hsa-miR-146a-5p	0.877001
UTR3	6:71195889-71195908	0.008035908	
UTR3	6:71233713-71233728	0.016936981	
UTR3	6:71235897-71235924	0.011808879	
UTR3	6:71270245-71270273	0.006029027	
ENST00000530332	ENSG00000170619 (COMMD5)	hsa-miR-146a-5p	0.876235
UTR3	8:146066574-146066591	0.042694905	
ENST00000304129	ENSG00000169129 (AFAP1L2)	hsa-miR-146a-5p	0.875534
UTR3	10:116055322-116055340	0.009606495	
UTR3	10:116054710-116054735	0.031486802	
UTR3	10:116061203-116061224	0.012209933	
ENST00000361170	ENSG00000183856 (IQGAP3)	hsa-miR-146a-5p	0.875294
UTR3	1:156496153-156496168	0.002159496	
UTR3	1:156495653-156495677	0.003310611	
UTR3	1:156495502-156495520	0.033857824	
UTR3	1:156495337-156495352	0.003225716	
ENST00000288266	ENSG00000157500 (APPL1)	hsa-miR-146a-5p	0.874913
UTR3	3:57304401-57304419	0.038551479	
UTR3	3:57306092-57306118	0.001315601	
UTR3	3:57306834-57306849	0.00262959	
ENST00000559228	ENSG00000128923 (FAM63B)	hsa-miR-146a-5p	0.874039
UTR3	15:59150524-59150539	0.002401236	
UTR3	15:59151236-59151262	0.00215856	
UTR3	15:59151686-59151708	0.003835109	
UTR3	15:59151766-59151792	0.000673299	
UTR3	15:59152813-59152828	0.018873515	
UTR3	15:59153503-59153529	0.014424807	
ENST00000377669	ENSG00000118922 (KLF12)	hsa-miR-146a-5p	0.873972
UTR3	13:74268339-74268365	0.017585594	
UTR3	13:74264581-74264602	0.001824137	
UTR3	13:74264573-74264597	0.000583357	
UTR3	13:74262746-74262767	0.001549094	
UTR3	13:74262675-74262694	0.00055678	
UTR3	13:74262479-74262505	0.008213742	
UTR3	13:74262110-74262137	0.000972156	

UTR3	13:74260424-74260448	0.006680963	
UTR3	13:74289534-74289559	0.031517243	
ENST00000449987	ENSG00000139746 (RBM26)	hsa-miR-146a-5p	0.873388
UTR3	13:79888956-79888963	0.027473727	
UTR3	13:79888831-79888857	0.010952664	
UTR3	13:79887432-79887458	0.002008815	
UTR3	13:79887314-79887330	0.001834874	
ENST00000282878	ENSG00000152932 (RAB3C)	hsa-miR-146a-5p	0.872873
UTR3	5:58147434-58147461	0.010292296	
UTR3	5:58147660-58147681	0.003076543	
UTR3	5:58148190-58148211	0.00175417	
UTR3	5:58148675-58148702	0.001637334	
UTR3	5:58148870-58148885	0.001226166	
UTR3	5:58149192-58149211	0.001025845	
UTR3	5:58149583-58149603	0.001710345	
UTR3	5:58151839-58151862	0.001465906	
UTR3	5:58151942-58151959	0.000887452	
UTR3	5:58152209-58152229	0.009631851	
UTR3	5:58153341-58153360	0.002007053	
UTR3	5:58153941-58153954	0.005441356	
UTR3	5:58154861-58154886	0.002037737	
ENST00000527531	ENSG00000166323 (C11orf65)	hsa-miR-146a-5p	0.872651
UTR3	11:108226508-108226533	0.036921392	
UTR3	11:108202116-108202141	0.005239956	
ENST00000402045	ENSG00000106003 (LFNG)	hsa-miR-146a-5p	0.872625
UTR3	7:2567985-2567997	0.042157568	
ENST00000428797	ENSG00000022355 (GABRA1)	hsa-miR-146a-5p	0.871911
UTR3	5:161324464-161324487	0.031101429	
UTR3	5:161325265-161325288	0.002762582	
UTR3	5:161326187-161326214	0.00419722	
UTR3	5:161326481-161326497	0.00338891	
UTR3	5:161322772-161322793	0.00499573	
ENST00000360380	ENSG00000123473 (STIL)	hsa-miR-146a-5p	0.871764
UTR3	1:47716631-47716655	0.039198069	
UTR3	1:47755221-47755246	0.021349662	
ENST00000437844	ENSG00000148516 (ZEB1)	hsa-miR-146a-5p	0.871648
UTR3	10:31676171-31676190	0.00853504	
UTR3	10:31809398-31809422	0.011144163	
UTR3	10:31815704-31815727	0.001732788	
UTR3	10:31815729-31815747	0.000613922	
UTR3	10:31817689-31817703	0.004730585	
UTR3	10:31818590-31818615	0.015257919	
ENST00000335712	ENSG00000119866 (BCL11A)	hsa-miR-146a-5p	0.870897
UTR3	2:60687290-60687304	0.027979964	
UTR3	2:60686689-60686714	0.004554857	

UTR3	2:60685213-60685229	0.009370192	
ENST00000429989	ENSG00000108219 (TSPAN14)	hsa-miR-146a-5p	0.870433
UTR3	10:82278521-82278545	0.008876355	
UTR3	10:82280054-82280073	0.001397661	
UTR3	10:82280256-82280282	0.001211949	
UTR3	10:82280630-82280650	0.001685567	
UTR3	10:82280967-82280987	0.000794967	
UTR3	10:82281347-82281359	0.003193286	
UTR3	10:82283117-82283141	0.002288527	
UTR3	10:82283744-82283770	0.000929983	
UTR3	10:82287965-82287989	0.002650809	
UTR3	10:82288712-82288736	0.008832677	
UTR3	10:82289091-82289114	0.001102502	
UTR3	10:82289257-82289273	0.001330126	
UTR3	10:82291783-82291804	0.005551705	
UTR3	10:82291789-82291817	0.001991551	
ENST00000457153	ENSG00000067715 (SYT1)	hsa-miR-146a-5p	0.869427
UTR3	12:79844490-79844498	0.041692355	
ENST00000257290	ENSG00000134853 (PDGFRA)	hsa-miR-146a-5p	0.86926
UTR3	4:55162416-55162440	0.023389211	
UTR3	4:55162454-55162478	0.001578997	
UTR3	4:55162731-55162751	0.012663279	
UTR3	4:55163739-55163763	0.004036736	
ENST00000454189	ENSG00000046653 (GPM6B)	hsa-miR-146a-5p	0.868404
UTR3	X:13792398-13792421	0.0184068	
UTR3	X:13792102-13792116	0.005043944	
UTR3	X:13791945-13791961	0.013682538	
UTR3	X:13791189-13791210	0.002298578	
UTR3	X:13790465-13790482	0.002113711	
ENST00000478500	ENSG00000107242 (PIP5K1B)	hsa-miR-146a-5p	0.867783
UTR3	9:71624080-71624091	0.041456815	
ENST00000610020	ENSG00000122484 (RPAP2)	hsa-miR-146a-5p	0.867238
UTR3	1:92854576-92854600	0.001274171	
UTR3	1:92859217-92859244	0.002354295	
UTR3	1:92859789-92859810	0.002882308	
UTR3	1:92860306-92860318	0.005335236	
UTR3	1:92864870-92864888	0.024695178	
UTR3	1:92865283-92865305	0.002280382	
UTR3	1:92867223-92867240	0.002557845	
ENST00000361717	ENSG00000145365 (TIFA)	hsa-miR-146a-5p	0.867095
UTR3	4:113198740-113198766	0.011690925	
UTR3	4:113198425-113198442	0.017154173	
UTR3	4:113196720-113196747	0.004409104	
UTR3	4:113196268-113196295	0.004299323	

UTR3	4:113199324-113199343	0.014130796	
UTR3	4:113199047-113199061	0.012759007	
ENST00000297107	ENSG00000164574 (GALNT10)	hsa-miR-146a-5p	0.867065
UTR3	5:153797062-153797081	0.002484148	
UTR3	5:153797535-153797552	0.036181836	
UTR3	5:153799093-153799109	0.002688871	
ENST00000357085	ENSG00000112343 (TRIM38)	hsa-miR-146a-5p	0.866765
UTR3	6:25984221-25984248	0.004799072	
UTR3	6:25987280-25987292	0.036513247	
ENST00000523756	ENSG00000042832 (TG)	hsa-miR-146a-5p	0.86628
UTR3	8:133953670-133953687	0.004375484	
UTR3	8:134042194-134042221	0.002170721	
UTR3	8:134108563-134108580	0.005962653	
UTR3	8:134145725-134145741	0.028734872	
ENST00000330796	ENSG00000185238 (PRMT3)	hsa-miR-146a-5p	0.865991
UTR3	11:20417367-20417389	0.006716912	
UTR3	11:20515505;20515713-20515515;20515723	0.00207816	
UTR3	11:20529894-20529906	0.002747902	
UTR3	11:20530080-20530101	0.014009601	
UTR3	11:20530519-20530541	0.015650479	
ENST00000439944	ENSG00000172061 (LRRC15)	hsa-miR-146a-5p	0.865799
UTR3	3:194079633-194079650	0.013725037	
UTR3	3:194079522-194079540	0.00795652	
UTR3	3:194079484-194079497	0.004549488	
UTR3	3:194076205-194076225	0.014944986	
ENST00000356722	ENSG00000145965 (C6ORF50)	hsa-miR-146a-5p	0.865701
UTR3	6:3984021-3984044	0.005609265	
UTR3	6:3984308-3984320	0.035552923	
ENST00000588072	ENSG00000154889 (MPPE1)	hsa-miR-146a-5p	0.865636
UTR3	18:11884320-11884343	0.004845538	
UTR3	18:11884270-11884278	0.016917076	
UTR3	18:11884225-11884249	0.003567424	
UTR3	18:11884034-11884052	0.01480929	
UTR3	18:11885782-11885802	0.007941433	
ENST00000242728	ENSG00000123095 (BHLHE41)	hsa-miR-146a-5p	0.864661
UTR3	12:26274510-26274529	0.020606212	
UTR3	12:26273097-26273121	0.020410338	
ENST00000372692	ENSG00000119335 (SET)	hsa-miR-146a-5p	0.86459
UTR3	9:131457082-131457096	0.037058691	
UTR3	9:131455159-131455185	0.027382555	
ENST00000424789	ENSG00000170921 (TANC2)	hsa-miR-146a-5p	0.864081
UTR3	17:61499380-61499395	0.011513763	
UTR3	17:61499382-61499410	0.008250654	

UTR3	17:61500174-61500186	0.002701374	
UTR3	17:61500855-61500875	0.001871319	
UTR3	17:61501183-61501202	0.000961625	
UTR3	17:61501209-61501237	0.000345591	
UTR3	17:61501431-61501458	0.001824288	
UTR3	17:61502718-61502739	0.001905023	
UTR3	17:61504765-61504785	0.009389586	
UTR3	17:61432300-61432325	0.016087132	
ENST00000496384	ENSG00000157764 (BRAF)	hsa-miR-146a-5p	0.862903
UTR3	7:140425859-140425874	0.005114732	
UTR3	7:140425543-140425565	0.00332037	
UTR3	7:140425476-140425497	0.004025534	
UTR3	7:140420736-140420758	0.005561867	
UTR3	7:140419632-140419655	0.003050794	
UTR3	7:140419451-140419471	0.003741559	
UTR3	7:140419381-140419406	0.006898935	
UTR3	7:140419337-140419354	0.0090586	
ENST00000504228	ENSG00000109265 (KIAA1211)	hsa-miR-146a-5p	0.862686
UTR3	4:57194164-57194189	0.037143102	
UTR3	4:57173864;57176832-57173865;57176853	0.015365145	
UTR3	4:57181808-57181827	0.009677894	
ENST00000327245	ENSG00000118322 (ATP10B)	hsa-miR-146a-5p	0.862312
UTR3	5:159992191-159992210	0.035918896	
UTR3	5:159990382-159990405	0.004771925	
ENST00000531813	ENSG00000075239 (ACAT1)	hsa-miR-146a-5p	0.861283
UTR3	11:108013198-108013223	0.012830262	
UTR3	11:108014184-108014202	0.027719424	
ENST00000475243	ENSG00000124164 (VAPB)	hsa-miR-146a-5p	0.861256
UTR3	20:57020221-57020241	0.001756563	
UTR3	20:57021822-57021849	0.001216652	
UTR3	20:57023185-57023196	0.004317453	
UTR3	20:57023845-57023865	0.001837278	
UTR3	20:57025521-57025543	0.02403161	
UTR3	20:57025680-57025695	0.001689584	
UTR3	20:57026027-57026048	0.00569678	
ENST00000361138	ENSG00000198887 (SMC5)	hsa-miR-146a-5p	0.861086
UTR3	9:72967481-72967501	0.03611848	
UTR3	9:72968122-72968143	0.004404149	
ENST00000460813	ENSG00000163617 (KIAA1407)	hsa-miR-146a-5p	0.860884
UTR3	3:113755907-113755934	0.029494793	
UTR3	3:113750597-113750616	0.005915099	
UTR3	3:113729826-113729843	0.005085227	
ENST00000415541	ENSG00000160584 (SIK3)	hsa-miR-146a-5p	0.86074
UTR3	11:116734465-116734483	0.004381748	

UTR3	11:116719964-116719985	0.013519895	
UTR3	11:116718243-116718267	0.022573782	
ENST00000373656	ENSG00000171448 (ZBTB26)	hsa-miR-146a-5p	0.860656
UTR3	9:125678455-125678476	0.035830244	
UTR3	9:125682099-125682119	0.030735829	
ENST00000283365	ENSG00000134769 (DTNA)	hsa-miR-146a-5p	0.860642
UTR3	18:32467909-32467931	0.010646252	
UTR3	18:32469185-32469210	0.012845126	
UTR3	18:32469763-32469791	0.010736779	
UTR3	18:32391983-32392000	0.029386347	
UTR3	18:32398242-32398257	0.009735065	
ENST00000282849	ENSG00000140873 (ADAMTS18)	hsa-miR-146a-5p	0.860332
UTR3	16:77316639-77316664	0.019225708	
UTR3	16:77316160-77316178	0.021194214	
ENST00000444766	ENSG00000108506 (INTS2)	hsa-miR-146a-5p	0.860187
UTR3	17:59944375-59944399	0.040400118	
ENST00000020926	ENSG00000019505 (SYT13)	hsa-miR-146a-5p	0.860152
UTR3	11:45265119-45265133	0.022504246	
UTR3	11:45264599-45264626	0.001354432	
UTR3	11:45262736-45262754	0.016536805	
ENST00000357345	ENSG00000159082 (SYNJ1)	hsa-miR-146a-5p	0.859695
UTR3	21:34003850-34003873	0.017225826	
UTR3	21:34003735-34003759	0.005268533	
UTR3	21:34003120-34003130	0.003712285	
UTR3	21:34002231-34002247	0.002241891	
UTR3	21:34001240-34001265	0.010552489	
UTR3	21:34018094;34018703-34018099;34018715	0.009960936	
ENST00000507740	ENSG00000176406 (RIMS2)	hsa-miR-146a-5p	0.859322
UTR3	8:105264180-105264194	0.023223596	
UTR3	8:105265189-105265214	0.003519293	
UTR3	8:105267240-105267257	0.009181043	
UTR3	8:105267654-105267672	0.00435899	
ENST00000498619	ENSG00000036828 (CASR)	hsa-miR-146a-5p	0.85922
UTR3	3:122004962-122004983	0.040269164	
ENST00000426923	ENSG00000145287 (PLAC8)	hsa-miR-146a-5p	0.859188
UTR3	4:84015597-84015620	0.007747126	
UTR3	4:84015251-84015276	0.006203951	
UTR3	4:84014713-84014741	0.026313765	
ENST00000381709	ENSG00000205777 (GAGE1)	hsa-miR-146a-5p	0.858677
UTR3	X:49370654-49370681	0.014032276	
UTR3	X:49370800-49370826	0.005331823	
UTR3	X:49372209-49372231	0.00123034	
UTR3	X:49372609-49372627	0.003342216	
UTR3	X:49373100-49373117	0.016259258	

ENST00000403263	ENSG00000114933 (INO80D)	hsa-miR-146a-5p	0.858554
UTR3	2:206868450-206868477	0.002723621	
UTR3	2:206867032-206867060	0.001898931	
UTR3	2:206865359-206865386	0.005096148	
UTR3	2:206862779-206862794	0.006577901	
UTR3	2:206862537-206862552	0.00272778	
UTR3	2:206860581-206860595	0.002359512	
UTR3	2:206860515-206860532	0.001097425	
UTR3	2:206859084-206859102	0.007240888	
UTR3	2:206859059-206859082	0.008054176	
UTR3	2:206869906-206869923	0.002519111	
UTR3	2:206869219-206869245	0.014605128	
ENST00000360150	ENSG00000087502 (ERGIC2)	hsa-miR-146a-5p	0.858323
UTR3	12:29493423-29493438	0.00300637	
UTR3	12:29491274-29491296	0.029942719	
UTR3	12:29491244-29491252	0.002527938	
UTR3	12:29496143-29496168	0.030599954	
ENST00000327381	ENSG00000206579 (XKR4)	hsa-miR-146a-5p	0.858229
UTR3	8:56437309-56437333	0.004827051	
UTR3	8:56437894-56437914	0.008252979	
UTR3	8:56438162-56438178	0.002406801	
UTR3	8:56438759-56438784	0.003735967	
UTR3	8:56439306-56439331	0.002536554	
UTR3	8:56443003-56443027	0.001570633	
UTR3	8:56443151-56443167	0.00128767	
UTR3	8:56443589-56443612	0.001222552	
UTR3	8:56446845-56446872	0.001404559	
UTR3	8:56448422-56448445	0.005856192	
UTR3	8:56453300-56453318	0.001319395	
UTR3	8:56454025-56454043	0.005715276	
ENST00000237654	ENSG00000118816 (CCNI)	hsa-miR-146a-5p	0.857495
UTR3	4:77968808-77968836	0.040037295	
ENST00000582913	ENSG00000154864 (PIEZO2)	hsa-miR-146a-5p	0.857321
UTR3	18:10708407-10708420	0.008974848	
UTR3	18:10705538-10705564	0.006927301	
UTR3	18:10702038-10702053	0.001614812	
UTR3	18:10672793-10672814	0.016289845	
UTR3	18:10671702-10671729	0.005068118	
UTR3	18:11066151-11066169	0.008473069	
ENST00000608382	ENSG00000089775 (ZBTB25)	hsa-miR-146a-5p	0.85709
UTR3	14:64953488-64953511	0.006535235	
UTR3	14:64949562-64949575	0.004256125	
UTR3	14:64949248-64949266	0.003800384	
UTR3	14:64946684-64946704	0.014141788	
UTR3	14:64945017-64945033	0.003027337	
UTR3	14:64944953-64944980	0.005328258	

UTR3	14:64954592-64954616	0.006922593	
UTR3	14:64953726-64953741	0.013172766	
ENST00000329255	ENSG00000052850 (ALX4)	hsa-miR-146a-5p	0.856624
UTR3	11:44285479-44285500	0.003899906	
UTR3	11:44284629-44284649	0.004796028	
UTR3	11:44284263-44284279	0.000922684	
UTR3	11:44283574-44283592	0.004644822	
UTR3	11:44283035-44283062	0.002372694	
UTR3	11:44282388-44282408	0.023284978	
ENST00000558084	ENSG00000055609 (KMT2C)	hsa-miR-146a-5p	0.855986
UTR3	7:151927048-151927075	0.009590812	
UTR3	7:151884390-151884408	0.004203949	
UTR3	7:151879573-151879597	0.005256612	
UTR3	7:151879336-151879358	0.001283183	
UTR3	7:151879321-151879339	0.000576629	
UTR3	7:151878685-151878706	0.002262331	
UTR3	7:151877896-151877914	0.002085102	
UTR3	7:151874120-151874132	0.001367202	
UTR3	7:151873784-151873810	0.008306029	
UTR3	7:151873584-151873598	0.002302111	
UTR3	7:151859592-151859610	0.001111922	
UTR3	7:151949757-151949776	0.010857601	
ENST00000312675	ENSG00000145012 (LPP)	hsa-miR-146a-5p	0.855293
UTR3	3:188593383-188593404	0.002804742	
UTR3	3:188594207-188594218	0.003733323	
UTR3	3:188594221-188594242	0.000919612	
UTR3	3:188596832-188596858	0.003967759	
UTR3	3:188596919-188596940	0.000994604	
UTR3	3:188597355-188597379	0.001212435	
UTR3	3:188598529-188598544	0.006753888	
UTR3	3:188599764-188599791	0.002146874	
UTR3	3:188600895-188600916	0.001646333	
UTR3	3:188602996-188603008	0.001517009	
UTR3	3:188603150-188603176	0.001235532	
UTR3	3:188603846-188603867	0.002549689	
UTR3	3:188604458-188604471	0.001389814	
UTR3	3:188607180-188607199	0.008873021	
ENST00000251691	ENSG00000112379 (KIAA1244)	hsa-miR-146a-5p	0.855277
UTR3	6:138660262-138660290	0.001202645	
UTR3	6:138660789-138660808	0.001648708	
UTR3	6:138661589-138661611	0.002178916	
UTR3	6:138662759-138662782	0.019983684	
UTR3	6:138663259-138663279	0.007043601	
UTR3	6:138655441-138655462	0.004525262	
UTR3	6:138657437-138657462	0.040420547	
ENST00000304735	ENSG00000168140 (VASN)	hsa-miR-146a-5p	0.854768

UTR3	16:4432972-4432987	0.039675431	
ENST00000262135	ENSG00000103494 (RPGRIP1L)	hsa-miR-146a-5p	0.854722
UTR3	16:53635794-53635812	0.003767088	
UTR3	16:53635764-53635785	0.024778849	
UTR3	16:53635742-53635764	0.00679633	
UTR3	16:53632169-53632177	0.004327081	
ENST00000354650	ENSG00000010810 (FYN)	hsa-miR-146a-5p	0.854505
UTR3	6:111982720-111982736	0.006183548	
UTR3	6:111982115-111982136	0.015504305	
UTR3	6:111981923-111981941	0.006940798	
UTR3	6:111981805-111981817	0.011012175	
ENST00000281092	ENSG00000151422 (FER)	hsa-miR-146a-5p	0.854244
UTR3	5:108523317-108523343	0.029241723	
UTR3	5:108524971-108524996	0.007053107	
UTR3	5:108529174-108529200	0.001690294	
UTR3	5:108529398-108529413	0.001621417	
ENST00000427401	ENSG00000237440 (ZNF737)	hsa-miR-146a-5p	0.853988
UTR3	19:20726641-20726659	0.037738335	
UTR3	19:20728770;20735281-20728782;20735290	0.013054425	
ENST00000575022	ENSG00000177294 (FBXO39)	hsa-miR-146a-5p	0.853434
UTR3	17:6699363-6699381	0.039500419	
ENST00000373647	ENSG00000011454 (RABGAP1)	hsa-miR-146a-5p	0.85286
UTR3	9:125866641-125866663	0.037623847	
UTR3	9:125861068;125861757-125861077;125861767	0.012764399	
ENST00000395792	ENSG00000081051 (AFP)	hsa-miR-146a-5p	0.852227
UTR3	4:74321803-74321819	0.005625655	
UTR3	4:74321816-74321835	0.031124891	
UTR3	4:74313186-74313207	0.017779227	
ENST00000471923	ENSG00000048707 (VPS13D)	hsa-miR-146a-5p	0.85221
UTR3	1:12569728-12569747	0.001862968	
UTR3	1:12569867-12569894	0.002365042	
UTR3	1:12571103-12571128	0.002490058	
UTR3	1:12571458-12571472	0.005781212	
UTR3	1:12571963-12571985	0.007038247	
UTR3	1:12572023-12572043	0.019803514	
ENST00000234313	ENSG00000115956 (PLEK)	hsa-miR-146a-5p	0.851961
UTR3	2:68623043-68623065	0.010414537	
UTR3	2:68623452-68623476	0.006755162	
UTR3	2:68624040-68624059	0.022139112	
ENST00000265022	ENSG00000058866 (DGKG)	hsa-miR-146a-5p	0.851734
UTR3	3:185866658-185866675	0.004387634	

UTR3	3:185866224-185866236	0.001414116	
UTR3	3:185865478-185865498	0.00315961	
UTR3	3:185865125-185865151	0.004853848	
UTR3	3:185865041-185865064	0.025464152	
ENST00000539173	ENSG00000197381 (ADARB1)	hsa-miR-146a-5p	0.849589
UTR3	21:46642872-46642890	0.002333242	
UTR3	21:46645579-46645601	0.003688989	
UTR3	21:46646317-46646332	0.006077283	
UTR3	21:46646430-46646453	0.025782105	
UTR3	21:46642075-46642100	0.008025978	
ENST00000511739	ENSG00000170185 (USP38)	hsa-miR-146a-5p	0.849336
UTR3	4:144127209-144127228	0.010008504	
UTR3	4:144127235-144127250	0.008174562	
UTR3	4:144142773-144142800	0.005464425	
UTR3	4:144143098-144143115	0.002273483	
UTR3	4:144144834-144144852	0.004017163	
UTR3	4:144144947-144144963	0.005465667	
UTR3	4:144109033-144109053	0.02330858	
ENST00000467067	ENSG00000116285 (ERRFI1)	hsa-miR-146a-5p	0.849148
UTR3	1:8075201-8075228	0.004177511	
UTR3	1:8075105-8075123	0.033655423	
UTR3	1:8074586-8074599	0.001114113	
ENST00000534277	ENSG00000197302 (ZNF720)	hsa-miR-146a-5p	0.848927
UTR3	16:31734665;31765087-31734674;31765099	0.004201446	
UTR3	16:31767381-31767397	0.001813278	
UTR3	16:31767890-31767908	0.002574628	
UTR3	16:31768248-31768272	0.016389561	
UTR3	16:31768453-31768467	0.013939956	
ENST00000455584	ENSG00000251537 (RP11-385D13.1)	hsa-miR-146a-5p	0.84846
UTR3	17:15477218-15477243	0.026225729	
UTR3	17:15476104-15476131	0.004125517	
UTR3	17:15476009-15476026	0.006134479	
UTR3	17:15475686-15475712	0.00237367	
ENST00000456874	ENSG00000197057 (DTHD1)	hsa-miR-146a-5p	0.84823
UTR3	4:36345830-36345857	0.038830154	
ENST00000267884	ENSG00000140319 (SRP14)	hsa-miR-146a-5p	0.848011
UTR3	15:40328018-40328038	0.038802385	
ENST00000375826	ENSG00000116771 (AGMAT)	hsa-miR-146a-5p	0.847798
UTR3	1:15900097-15900113	0.03600743	
UTR3	1:15899986-15900012	0.002767977	
ENST00000299563	ENSG00000166439 (RNF169)	hsa-miR-146a-5p	0.847721
UTR3	11:74547827-74547850	0.026333335	
UTR3	11:74548436-74548462	0.004790655	
UTR3	11:74549318-74549338	0.004002639	

UTR3	11:74549335-74549354	0.000620631	
UTR3	11:74549641-74549662	0.000974438	
UTR3	11:74552304-74552321	0.002043926	
ENST00000409652	ENSG00000221963 (APOL6)	hsa-miR-146a-5p	0.847715
UTR3	22:36056750-36056778	0.003688126	
UTR3	22:36058236-36058262	0.001756655	
UTR3	22:36059578-36059592	0.001418743	
UTR3	22:36063553-36063573	0.026409593	
UTR3	22:36064356-36064382	0.005491812	
ENST00000295887	ENSG00000163624 (CDS1)	hsa-miR-146a-5p	0.847488
UTR3	4:85570261-85570284	0.011905614	
UTR3	4:85571856-85571881	0.002929674	
UTR3	4:85572409-85572436	0.023900967	
ENST00000606080	ENSG00000111816 (FRK)	hsa-miR-146a-5p	0.847303
UTR3	6:116262456-116262469	0.006705798	
UTR3	6:116260958-116260977	0.005016461	
UTR3	6:116259440-116259467	0.006203776	
UTR3	6:116259000-116259025	0.005979556	
UTR3	6:116258134-116258160	0.001423959	
UTR3	6:116258081-116258099	0.000555513	
UTR3	6:116257824-116257838	0.010319329	
UTR3	6:116255874-116255899	0.001457713	
UTR3	6:116255617-116255641	0.001050755	
ENST00000262352	ENSG00000106688 (SLC1A1)	hsa-miR-146a-5p	0.846919
UTR3	9:4585858-4585886	0.006274354	
UTR3	9:4586281-4586307	0.007769795	
UTR3	9:4586943-4586962	0.024620229	
ENST00000546136	ENSG00000134532 (SOX5)	hsa-miR-146a-5p	0.846556
UTR3	12:23687064-23687092	0.009914959	
UTR3	12:23686797-23686820	0.011804644	
UTR3	12:23686264-23686288	0.012213074	
UTR3	12:23684525-23684535	0.002665041	
UTR3	12:23684136-23684153	0.002020973	
ENST00000253144	ENSG00000130844 (ZNF331)	hsa-miR-146a-5p	0.846392
UTR3	19:54081623-54081636	0.038598057	
ENST00000296402	ENSG00000145349 (CAMK2D)	hsa-miR-146a-5p	0.845834
UTR3	4:114375632-114375658	0.017801652	
UTR3	4:114375331-114375351	0.0055279	
UTR3	4:114374954-114374976	0.003114999	
UTR3	4:114374282-114374305	0.002711897	
UTR3	4:114374242-114374260	0.000743129	
UTR3	4:114373718-114373743	0.003302019	
UTR3	4:114373425-114373444	0.002634639	
UTR3	4:114372718-114372743	0.00269178	
ENST00000538183	ENSG00000112096 (SOD2)	hsa-miR-146a-5p	0.8458
UTR3	6:160099466-160099490	0.000775212	

UTR3	6:160098820-160098845	0.001941728	
UTR3	6:160098324-160098339	0.001623366	
UTR3	6:160097579-160097598	0.003379259	
UTR3	6:160096095-160096114	0.004689574	
UTR3	6:160095330-160095354	0.007054545	
UTR3	6:160094581-160094608	0.001825159	
UTR3	6:160093269-160093295	0.001103695	
UTR3	6:160093216-160093240	0.001753146	
UTR3	6:160093054-160093072	0.001302045	
UTR3	6:160092958-160092985	0.004520262	
UTR3	6:160092306-160092333	0.001755003	
UTR3	6:160091513-160091532	0.006800766	
ENST00000358691	ENSG00000198265 (HELZ)	hsa-miR-146a-5p	0.845011
UTR3	17:65074246-65074262	0.024570002	
UTR3	17:65073502-65073528	0.003478352	
UTR3	17:65071922-65071950	0.001388923	
UTR3	17:65069146-65069169	0.008987742	
ENST00000342771	ENSG00000158321 (AUTS2)	hsa-miR-146a-5p	0.844949
UTR3	7:70256179-70256198	0.015883036	
UTR3	7:70257043-70257071	0.003169963	
UTR3	7:70257779-70257797	0.019364331	
ENST00000525284	ENSG00000154237 (LRRK1)	hsa-miR-146a-5p	0.844906
UTR3	15:101589908-101589933	0.001703729	
UTR3	15:101595419;101597061-101595428;101597074	0.00405716	
UTR3	15:101598266-101598293	0.001593201	
UTR3	15:101609118-101609136	0.011966078	
UTR3	15:101609141-101609165	0.005550374	
UTR3	15:101609207-101609231	0.004275327	
UTR3	15:101609220-101609239	0.003078149	
UTR3	15:101609237-101609254	0.006187941	
ENST00000334567	ENSG00000186329 (TMEM212)	hsa-miR-146a-5p	0.843873
UTR3	3:171576846-171576867	0.03828342	
ENST00000508624	ENSG00000134982 (APC)	hsa-miR-146a-5p	0.843444
UTR3	5:112173799-112173825	0.007332636	
UTR3	5:112176690-112176709	0.00195377	
UTR3	5:112176869-112176884	0.008399541	
UTR3	5:112177166-112177185	0.001772431	
UTR3	5:112177182-112177205	0.002023995	
UTR3	5:112177354-112177370	0.00982467	
UTR3	5:112177354-112177379	0.006923252	
ENST00000328902	ENSG00000133561 (GIMAP6)	hsa-miR-146a-5p	0.842574
UTR3	7:150324368-150324391	0.003872322	
UTR3	7:150323014-150323031	0.029907014	
UTR3	7:150322849-150322863	0.004343574	

ENST00000339570	ENSG00000073060 (SCARB1)	hsa-miR-146a-5p	0.842316
UTR3	12:125261732-125261759	0.038091023	
ENST00000465065	ENSG00000163728 (TTC14)	hsa-miR-146a-5p	0.841991
UTR3	3:180321980-180322006	0.002884282	
UTR3	3:180322022-180322044	0.002079273	
UTR3	3:180322235-180322248	0.010446992	
UTR3	3:180323806-180323827	0.001237929	
UTR3	3:180325907-180325927	0.003185602	
UTR3	3:180326870-180326889	0.004852231	
UTR3	3:180327113-180327137	0.006404825	
UTR3	3:180328453-180328480	0.006960046	
ENST00000261942	ENSG00000113194 (FAF2)	hsa-miR-146a-5p	0.841295
UTR3	5:175934289-175934302	0.035455277	
UTR3	5:175935765-175935777	0.002510449	
ENST00000568578	ENSG00000147274 (RBMX)	hsa-miR-146a-5p	0.840722
UTR3	X:135957513-135957525	0.018341125	
UTR3	X:135955832-135955856	0.006373582	
UTR3	X:135951946-135951967	0.013181008	
ENST00000344419	ENSG00000009765 (IYD)	hsa-miR-146a-5p	0.840546
UTR3	6:150719472-150719499	0.002494505	
UTR3	6:150719760-150719781	0.002129818	
UTR3	6:150720652-150720678	0.001321462	
UTR3	6:150721442-150721454	0.003702191	
UTR3	6:150722219-150722246	0.015194171	
UTR3	6:150722726-150722746	0.001218708	
UTR3	6:150726039-150726061	0.002907563	
UTR3	6:150726283-150726308	0.00210062	
UTR3	6:150726852-150726864	0.006805258	
ENST00000371172	ENSG00000197430 (OPALIN)	hsa-miR-146a-5p	0.840493
UTR3	10:98105377-98105404	0.004978911	
UTR3	10:98105350-98105362	0.004954325	
UTR3	10:98104953-98104973	0.008603823	
UTR3	10:98103604-98103625	0.003048014	
UTR3	10:98103243-98103270	0.016282728	
ENST00000275072	ENSG00000146281 (PM20D2)	hsa-miR-146a-5p	0.840122
UTR3	6:89872421-89872437	0.004037484	
UTR3	6:89873535-89873559	0.002048324	
UTR3	6:89874694-89874719	0.031736765	
ENST00000394650	ENSG00000005059 (CCDC109B)	hsa-miR-146a-5p	0.840103
UTR3	4:110608874-110608891	0.037820311	
ENST00000422265	ENSG00000144648 (ACKR2)	hsa-miR-146a-5p	0.840072
UTR3	3:42907248-42907267	0.016600797	
UTR3	3:42907248-42907274	0.02121579	

ENST00000441535	ENSG00000094963 (FMO2)	hsa-miR-146a-5p	0.840012
UTR3	1:171178182-171178209	0.003128683	
UTR3	1:171178704-171178728	0.03112554	
UTR3	1:171179000-171179018	0.003555032	
ENST00000513291	ENSG00000175054 (ATR)	hsa-miR-146a-5p	0.839732
UTR3	3:142171825-142171843	0.014008067	
UTR3	3:142171424-142171444	0.00803428	
UTR3	3:142170362-142170388	0.001509749	
UTR3	3:142168162-142168179	0.014223052	
ENST00000381052	ENSG00000049192 (ADAMTS6)	hsa-miR-146a-5p	0.839592
UTR3	5:64747971-64747990	0.025700259	
UTR3	5:64445769-64445784	0.00801707	
UTR3	5:64769406-64769431	0.024930067	
ENST00000216513	ENSG00000100625 (SIX4)	hsa-miR-146a-5p	0.839419
UTR3	14:61178171-61178187	0.003641602	
UTR3	14:61176592-61176617	0.034095666	
ENST00000356264	ENSG00000078053 (AMPH)	hsa-miR-146a-5p	0.837819
UTR3	7:38423942-38423961	0.037543996	
ENST00000381779	ENSG00000179542 (SLITRK4)	hsa-miR-146a-5p	0.837783
UTR3	X:142715602-142715621	0.017982249	
UTR3	X:142713810-142713833	0.001455954	
UTR3	X:142710726-142710745	0.007862679	
UTR3	X:142718444-142718467	0.052110743	
ENST00000368744	ENSG00000203783 (PRR9)	hsa-miR-146a-5p	0.837478
UTR3	1:153191155-153191181	0.014985065	
UTR3	1:153191639-153191662	0.018732695	
UTR3	1:153191701-153191721	0.003785258	
ENST00000256637	ENSG00000134243 (SORT1)	hsa-miR-146a-5p	0.837432
UTR3	1:109855549-109855565	0.001901908	
UTR3	1:109855082-109855100	0.002694417	
UTR3	1:109853469-109853486	0.005133756	
UTR3	1:109852714-109852737	0.025783105	
UTR3	1:109867706;109869614-109867711;109869627	0.013015178	
ENST00000254958	ENSG00000101384 (JAG1)	hsa-miR-146a-5p	0.837387
UTR3	20:10619390-10619418	0.002829436	
UTR3	20:10618453-10618471	0.034662667	
ENST00000366857	ENSG00000143771 (CNIH4)	hsa-miR-146a-5p	0.836519
UTR3	1:224563703-224563723	0.030424477	
UTR3	1:224565497-224565519	0.002221402	
UTR3	1:224566128-224566144	0.001923766	
UTR3	1:224566620-224566633	0.002818462	
ENST00000322434	ENSG00000178338 (ZNF354B)	hsa-miR-146a-5p	0.836402
UTR3	5:178311356-178311378	0.037374193	

ENST00000396578	ENSG00000169031 (COL4A3)	hsa-miR-146a-5p	0.835188
UTR3	2:228176775-228176798	0.008615809	
UTR3	2:228177550-228177570	0.002474424	
UTR3	2:228178535-228178560	0.01832064	
UTR3	2:228178913-228178933	0.002409671	
UTR3	2:228179180-228179207	0.005409072	
ENST00000609129	ENSG00000273274 (ZBTB8B)	hsa-miR-146a-5p	0.835146
UTR3	1:32952808-32952833	0.003281662	
UTR3	1:32954102-32954125	0.001266589	
UTR3	1:32955354-32955381	0.001161934	
UTR3	1:32956005-32956024	0.003194311	
UTR3	1:32957329-32957351	0.009143861	
UTR3	1:32957464-32957482	0.00175395	
UTR3	1:32958608-32958626	0.001454578	
UTR3	1:32959900-32959927	0.005876584	
UTR3	1:32960489-32960510	0.005908212	
UTR3	1:32961561-32961588	0.004182919	
ENST00000409198	ENSG00000183091 (NEB)	hsa-miR-146a-5p	0.834599
UTR3	2:152342184-152342205	0.03551503	
UTR3	2:152507242-152507259	0.006088684	
UTR3	2:152466477-152466501	0.004700124	
ENST00000471389	ENSG00000140718 (FTO)	hsa-miR-146a-5p	0.834394
UTR3	16:54146464-54146479	0.002579032	
UTR3	16:54148438-54148460	0.02908429	
UTR3	16:54150629-54150652	0.001112845	
UTR3	16:54150700-54150718	0.000842553	
UTR3	16:54151038-54151063	0.00106127	
UTR3	16:54153520-54153546	0.00245555	
ENST00000361987	ENSG00000242689 (CNTF)	hsa-miR-146a-5p	0.833886
UTR3	11:58392956-58392973	0.037075474	
ENST00000398653	ENSG00000115970 (THADA)	hsa-miR-146a-5p	0.833749
UTR3	2:43798857-43798884	0.002124442	
UTR3	2:43797621-43797638	0.014859856	
UTR3	2:43520084-43520105	0.003049931	
UTR3	2:43458152-43458164	0.017025107	
ENST00000404138	ENSG00000184677 (ZBTB40)	hsa-miR-146a-5p	0.832945
UTR3	1:22853029-22853054	0.025498974	
UTR3	1:22855007-22855025	0.001321592	
UTR3	1:22855250-22855273	0.002183428	
UTR3	1:22855474-22855492	0.004707455	
UTR3	1:22848053-22848072	0.020067452	
ENST00000371322	ENSG00000158290 (CUL4B)	hsa-miR-146a-5p	0.832538
UTR3	X:119660468-119660482	0.003371082	
UTR3	X:119659422-119659439	0.009816474	
UTR3	X:119659068-119659094	0.021252098	
UTR3	X:119658934-119658948	0.002477323	

ENST00000399639	ENSG00000143469 (SYT14)	hsa-miR-146a-5p	0.8325
UTR3	1:210334832-210334849	0.010441792	
UTR3	1:210335377-210335399	0.003721008	
UTR3	1:210335439-210335460	0.010259535	
UTR3	1:210335887-210335904	0.002193639	
UTR3	1:210336966-210336990	0.003196109	
UTR3	1:210337559-210337586	0.00710043	
ENST00000375290	ENSG00000185920 (PTCH1)	hsa-miR-146a-5p	0.832437
UTR3	9:98231136-98231155	0.017736586	
UTR3	9:98214374-98214402	0.001167429	
UTR3	9:98214245-98214265	0.000497656	
UTR3	9:98212176-98212193	0.006424267	
UTR3	9:98209498-98209520	0.002536672	
UTR3	9:98207147-98207164	0.00185277	
UTR3	9:98205798-98205823	0.006689733	
ENST00000416913	ENSG00000162869 (PPP1R21)	hsa-miR-146a-5p	0.83189
UTR3	2:48685269-48685287	0.005811932	
UTR3	2:48692766;48698226-48692781;48698231	0.029010169	
UTR3	2:48722859-48722875	0.002018985	
ENST00000371065	ENSG00000213625 (LEPROT)	hsa-miR-146a-5p	0.831087
UTR3	1:65897745-65897773	0.003434168	
UTR3	1:65898333-65898353	0.005562506	
UTR3	1:65898476-65898503	0.009633874	
UTR3	1:65900524-65900540	0.008562254	
UTR3	1:65900710-65900725	0.002134515	
UTR3	1:65900988-65901006	0.007420103	
ENST00000354200	ENSG00000125875 (TBC1D20)	hsa-miR-146a-5p	0.83055
UTR3	20:418405-418429	0.036684987	
ENST00000393203	ENSG00000134247 (PTGFRN)	hsa-miR-146a-5p	0.830271
UTR3	1:117532705-117532727	0.028240992	
UTR3	1:117532729-117532751	0.006108076	
UTR3	1:117509634-117509652	0.014515316	
ENST00000361902	ENSG00000198704 (GPX6)	hsa-miR-146a-5p	0.830223
UTR3	6:28471504-28471518	0.028787543	
UTR3	6:28471117-28471144	0.007859475	
ENST00000373075	ENSG00000163875 (MEAF6)	hsa-miR-146a-5p	0.829713
UTR3	1:37959349-37959368	0.005170844	
UTR3	1:37958906-37958925	0.003563336	
UTR3	1:37958199-37958225	0.027853774	
ENST00000536441	ENSG00000149212 (SESN3)	hsa-miR-146a-5p	0.829673
UTR3	11:94906319-94906339	0.023541325	
UTR3	11:94905500-94905518	0.003963915	
UTR3	11:94904191-94904206	0.002627982	

UTR3	11:94903446-94903473	0.002153479	
UTR3	11:94900125-94900151	0.002596264	
UTR3	11:94899920-94899940	0.001700323	
ENST00000272748	ENSG00000144320 (KIAA1715)	hsa-miR-146a-5p	0.829255
UTR3	2:176794330-176794353	0.01659684	
UTR3	2:176794156-176794183	0.004109183	
UTR3	2:176794093-176794116	0.002807133	
UTR3	2:176792260-176792286	0.003710581	
UTR3	2:176791459-176791485	0.002543192	
UTR3	2:176790159-176790178	0.001148933	
UTR3	2:176789976-176789998	0.001554077	
UTR3	2:176857057-176857078	0.024086454	
ENST00000368494	ENSG00000153989 (NUS1)	hsa-miR-146a-5p	0.828745
UTR3	6:118029369-118029377	0.00355471	
UTR3	6:118030557-118030573	0.012090992	
UTR3	6:118031091-118031114	0.003872541	
UTR3	6:118031534-118031553	0.016958035	
ENST00000371818	ENSG00000119917 (IFIT3)	hsa-miR-146a-5p	0.827671
UTR3	10:91100339-91100352	0.003730126	
UTR3	10:91100427-91100453	0.029930356	
UTR3	10:91098969-91098987	0.016579866	
ENST00000244763	ENSG00000124783 (SSR1)	hsa-miR-146a-5p	0.827525
UTR3	6:7287053-7287076	0.002921925	
UTR3	6:7284462-7284474	0.001292585	
UTR3	6:7283037-7283052	0.001953488	
UTR3	6:7282522-7282547	0.0022037	
UTR3	6:7282364-7282382	0.001882027	
UTR3	6:7282157-7282177	0.009482739	
UTR3	6:7281850-7281877	0.016599654	
ENST00000397609	ENSG00000171262 (FAM98B)	hsa-miR-146a-5p	0.827366
UTR3	15:38777873-38777900	0.023209398	
UTR3	15:38779177-38779188	0.013108531	
ENST00000566400	ENSG00000140463 (BBS4)	hsa-miR-146a-5p	0.827336
UTR3	15:73016988;73020281-73016996;73020287	0.033244536	
UTR3	15:73023995-73024013	0.00307001	
ENST00000514770	ENSG00000138658 (C4orf21)	hsa-miR-146a-5p	0.827262
UTR3	4:113539835-113539857	0.01613986	
UTR3	4:113533684-113533705	0.007832682	
UTR3	4:113531935-113531956	0.012333472	
ENST00000375360	ENSG00000158079 (PTPDC1)	hsa-miR-146a-5p	0.827255
UTR3	9:96870303-96870321	0.005261173	
UTR3	9:96870935-96870960	0.01623888	

UTR3	9:96871885-96871908	0.014805162	
ENST00000410015	ENSG00000115919 (KYNU)	hsa-miR-146a-5p	0.826924
UTR3	2:143704771-143704796	0.036267436	
ENST00000428210	ENSG00000082898 (XPO1)	hsa-miR-146a-5p	0.826839
UTR3	2:61726030;61726848-61726048;61726852	0.001918986	
UTR3	2:61725917;61726000-61725927;61726015	0.001224756	
UTR3	2:61720853-61720880	0.001146475	
UTR3	2:61713055-61713077	0.03196745	
ENST00000554659	ENSG00000100568 (VTI1B)	hsa-miR-146a-5p	0.826065
UTR3	14:68117742-68117764	0.020694763	
UTR3	14:68117627-68117654	0.004576677	
UTR3	14:68116718-68116737	0.003443384	
UTR3	14:68115814-68115828	0.001617218	
UTR3	14:68115652-68115672	0.001449771	
UTR3	14:68123224-68123246	0.02544167	
ENST00000299333	ENSG00000166257 (SCN3B)	hsa-miR-146a-5p	0.825592
UTR3	11:123503950-123503967	0.003993802	
UTR3	11:123501813-123501825	0.001951447	
UTR3	11:123501604-123501622	0.009463242	
UTR3	11:123501519-123501545	0.001155742	
UTR3	11:123501418-123501432	0.00129164	
UTR3	11:123501183-123501210	0.016106078	
UTR3	11:123500912-123500928	0.002153856	
ENST00000326524	ENSG00000168621 (GDNF)	hsa-miR-146a-5p	0.825522
UTR3	5:37812845-37812857	0.036107832	
ENST00000354833	ENSG00000172058 (SERF1A)	hsa-miR-146a-5p	0.824887
UTR3	5:70213256-70213276	0.020300214	
UTR3	5:70213949-70213967	0.009536279	
UTR3	5:70214062-70214088	0.006199432	
ENST00000408907	ENSG00000222028 (PSMB11)	hsa-miR-146a-5p	0.824791
UTR3	14:23512897-23512919	0.036025031	
ENST00000512733	ENSG00000163291 (PAQR3)	hsa-miR-146a-5p	0.824329
UTR3	4:79841040-79841055	0.002823661	
UTR3	4:79839865-79839892	0.002647571	
UTR3	4:79836873-79836898	0.001506855	
UTR3	4:79836741-79836766	0.023543436	
UTR3	4:79836710-79836725	0.000692242	
UTR3	4:79835729-79835749	0.001892191	
UTR3	4:79835720-79835748	0.000547132	
UTR3	4:79833500-79833522	0.002319718	
ENST00000283943	ENSG00000153827 (TRIP12)	hsa-miR-146a-5p	0.823965
UTR3	2:230631707-230631721	0.027311756	
UTR3	2:230631667-230631694	0.002080421	
UTR3	2:230631157-230631184	0.004783985	

UTR3	2:230629631-230629652	0.001755559	
ENST00000415083	ENSG00000141380 (SS18)	hsa-miR-146a-5p	0.823843
UTR3	18:23597711-23597728	0.007961416	
UTR3	18:23596819-23596838	0.017233809	
UTR3	18:23596595-23596614	0.009695367	
UTR3	18:23658125;23667465-23658124;23667482	0.006594678	
ENST00000435820	ENSG00000197134 (ZNF257)	hsa-miR-146a-5p	0.823556
UTR3	19:22256358;22270779-22256366;22270791	0.013190719	
UTR3	19:22272041-22272063	0.001510305	
UTR3	19:22272447-22272465	0.010085155	
UTR3	19:22272529-22272546	0.011099574	
ENST00000609686	ENSG00000273079 (GRIN2B)	hsa-miR-146a-5p	0.823203
UTR3	12:13715512-13715531	0.011999721	
UTR3	12:13713300-13713317	0.003206195	
UTR3	12:13709899-13709920	0.001399555	
UTR3	12:13708931-13708952	0.001571386	
UTR3	12:13708338-13708349	0.001919985	
UTR3	12:13708121-13708144	0.002978573	
UTR3	12:13706412-13706433	0.001546158	
UTR3	12:13704166-13704191	0.003082128	
UTR3	12:13704119-13704140	0.000517781	
UTR3	12:13704071-13704098	0.000619487	
UTR3	12:13703843-13703863	0.002394843	
UTR3	12:13699983-13699997	0.001549966	
UTR3	12:13699762-13699782	0.001802554	
UTR3	12:14019057-14019084	0.007989697	
ENST00000301785	ENSG00000214753 (HNRNPUL2)	hsa-miR-146a-5p	0.822912
UTR3	11:62481185-62481202	0.005537309	
UTR3	11:62480176-62480186	0.030276082	
ENST00000422875	ENSG00000160218 (TRAPPC10)	hsa-miR-146a-5p	0.822303
UTR3	21:45483541-45483563	0.00704606	
UTR3	21:45507670-45507689	0.003957201	
UTR3	21:45523589-45523617	0.00153099	
UTR3	21:45524594-45524610	0.023210879	
ENST00000296575	ENSG00000164161 (HHIP)	hsa-miR-146a-5p	0.822149
UTR3	4:145661578-145661594	0.001785952	
UTR3	4:145663299-145663319	0.006387757	
UTR3	4:145663402-145663428	0.001360881	
UTR3	4:145664352-145664379	0.00156666	
UTR3	4:145664660-145664678	0.001574764	
UTR3	4:145665792-145665818	0.023051938	
ENST00000262096	ENSG00000104219 (ZDHHC2)	hsa-miR-146a-5p	0.822061

UTR3	8:17077852-17077874	0.014421892	
UTR3	8:17081112-17081128	0.00216024	
UTR3	8:17082167-17082185	0.015787039	
UTR3	8:17082184-17082202	0.003348923	
ENST00000282869	ENSG00000152926 (ZNF117)	hsa-miR-146a-5p	0.821796
UTR3	7:64438155-64438180	0.026706056	
UTR3	7:64437483-64437508	0.008982432	
ENST00000556250	ENSG00000187790 (FANCM)	hsa-miR-146a-5p	0.821615
UTR3	14:45669896-45669922	0.021497994	
UTR3	14:45669959-45669972	0.014170353	
ENST00000389534	ENSG00000197608 (ZNF841)	hsa-miR-146a-5p	0.821024
UTR3	19:52568231-52568250	0.00496667	
UTR3	19:52568182-52568199	0.030635808	
ENST00000369466	ENSG00000116830 (TTF2)	hsa-miR-146a-5p	0.82009
UTR3	1:117644959-117644977	0.005284154	
UTR3	1:117645967-117645995	0.001803199	
UTR3	1:117647168-117647190	0.001390362	
UTR3	1:117650049-117650074	0.027021147	
ENST00000303635	ENSG00000171735 (CAMTA1)	hsa-miR-146a-5p	0.819997
UTR3	1:7826839-7826865	0.002786948	
UTR3	1:7826862-7826887	0.005694066	
UTR3	1:7827024-7827044	0.002931474	
UTR3	1:7827068-7827094	0.01425906	
UTR3	1:7829134-7829157	0.006515012	
UTR3	1:6880274-6880289	0.013035222	
UTR3	1:7724909-7724928	0.006354744	
ENST00000402775	ENSG00000172478 (C2orf54)	hsa-miR-146a-5p	0.819808
UTR3	2:241825675-241825699	0.013157252	
UTR3	2:241825655-241825678	0.022310365	
ENST00000383083	ENSG00000114698 (PLSCR4)	hsa-miR-146a-5p	0.819478
UTR3	3:145911887-145911901	0.026444213	
UTR3	3:145911548-145911565	0.004284452	
UTR3	3:145911197-145911210	0.004702441	
ENST00000379096	ENSG00000076554 (TPD52)	hsa-miR-146a-5p	0.818928
UTR3	8:80949928-80949950	0.005406207	
UTR3	8:80948638-80948658	0.001244509	
UTR3	8:80947175-80947196	0.02871975	
ENST00000238961	ENSG00000119906 (FAM178A)	hsa-miR-146a-5p	0.81875
UTR3	10:102722870-102722889	0.001601739	
UTR3	10:102723398-102723416	0.003823771	
UTR3	10:102723780-102723808	0.011639954	
UTR3	10:102724860-102724884	0.01286295	
UTR3	10:102710465-102710488	0.029728867	

ENST00000379119	ENSG00000075790 (BCAP29)	hsa-miR-146a-5p	0.81767
UTR3	7:107260439-107260463	0.002114259	
UTR3	7:107261947-107261972	0.001731328	
UTR3	7:107263092-107263104	0.03138659	
ENST00000361421	ENSG00000198846 (TOX)	hsa-miR-146a-5p	0.816953
UTR3	8:59720222-59720237	0.025981799	
UTR3	8:59719023-59719041	0.00917191	
ENST00000532485	ENSG00000078124 (ACER3)	hsa-miR-146a-5p	0.816796
UTR3	11:76731856-76731872	0.01743904	
UTR3	11:76732268-76732291	0.012409535	
UTR3	11:76735793-76735810	0.002203705	
UTR3	11:76737233-76737245	0.003084301	
ENST00000329468	ENSG00000185823 (NPAP1)	hsa-miR-146a-5p	0.816362
UTR3	15:24925614-24925633	0.018300963	
UTR3	15:24925883-24925906	0.003981422	
UTR3	15:24928276-24928285	0.004644839	
UTR3	15:24928303-24928321	0.005222014	
UTR3	15:24921637-24921665	0.017244096	
ENST00000350082	ENSG00000112144 (ICK)	hsa-miR-146a-5p	0.814819
UTR3	6:52868993-52869020	0.009217637	
UTR3	6:52868939-52868967	0.002945417	
UTR3	6:52867894-52867918	0.00240255	
UTR3	6:52867112-52867126	0.006767501	
UTR3	6:52866723-52866747	0.005276483	
UTR3	6:52866462-52866484	0.004904195	
UTR3	6:52866398-52866419	0.003407761	
ENST00000414716	ENSG00000099814 (CEP170B)	hsa-miR-146a-5p	0.814324
UTR3	14:105361371-105361387	0.005517849	
UTR3	14:105362278-105362301	0.00405838	
UTR3	14:105362965-105362993	0.025291736	
ENST00000343407	ENSG00000128536 (CDHR3)	hsa-miR-146a-5p	0.814321
UTR3	7:105673770-105673790	0.012277562	
UTR3	7:105676041-105676068	0.001889964	
UTR3	7:105676454-105676474	0.004429973	
UTR3	7:105676684-105676710	0.010792787	
UTR3	7:105676801-105676816	0.005477345	
ENST00000319550	ENSG00000178425 (NT5DC1)	hsa-miR-146a-5p	0.814305
UTR3	6:116565478-116565497	0.010523001	
UTR3	6:116565868-116565889	0.003136586	
UTR3	6:116568307-116568330	0.001434296	
UTR3	6:116569206-116569215	0.001229383	
UTR3	6:116569390-116569413	0.001679868	
UTR3	6:116570335-116570358	0.012005825	
UTR3	6:116429571-116429597	0.007464838	

UTR3	6:116542326-116542352	0.019226713	
ENST00000370457	ENSG00000155966 (AFF2)	hsa-miR-146a-5p	0.814182
UTR3	X:148072941-148072960	0.008736328	
UTR3	X:148073619-148073638	0.001342664	
UTR3	X:148076382-148076399	0.001658614	
UTR3	X:148076701-148076721	0.00226229	
UTR3	X:148077490-148077513	0.00128606	
UTR3	X:148081990-148082015	0.019566605	
ENST00000299163	ENSG00000166135 (HIF1AN)	hsa-miR-146a-5p	0.812644
UTR3	10:102308688-102308701	0.005071994	
UTR3	10:102308877-102308896	0.003489716	
UTR3	10:102309669-102309685	0.001891869	
UTR3	10:102309708-102309722	0.000709555	
UTR3	10:102309845-102309872	0.001429449	
UTR3	10:102310125-102310147	0.001518464	
UTR3	10:102311872-102311895	0.001490624	
UTR3	10:102312007-102312026	0.001036965	
UTR3	10:102312293-102312308	0.001816893	
UTR3	10:102313342-102313362	0.001903774	
UTR3	10:102315086-102315103	0.004776411	
UTR3	10:102315196-102315213	0.000867296	
UTR3	10:102315298-102315310	0.004519592	
UTR3	10:102316392-102316408	0.004164319	
ENST00000240189	ENSG00000120952 (PRAMEF2)	hsa-miR-146a-5p	0.812298
UTR3	1:12921661-12921674	0.034649855	
ENST00000322244	ENSG00000033178 (UBA6)	hsa-miR-146a-5p	0.812105
UTR3	4:68479257-68479272	0.001495473	
UTR3	4:68479234-68479257	0.02861081	
UTR3	4:68478596-68478624	0.003232694	
UTR3	4:68539431-68539454	0.007861871	
ENST00000425817	ENSG00000135917 (SLC19A3)	hsa-miR-146a-5p	0.811911
UTR3	2:228551948-228551971	0.013725957	
UTR3	2:228551607-228551619	0.020882441	
ENST00000602526	ENSG00000106692 (FKTN)	hsa-miR-146a-5p	0.811769
UTR3	9:108363517-108363544	0.006213445	
UTR3	9:108370123-108370143	0.024500604	
UTR3	9:108398366-108398388	0.001580433	
UTR3	9:108401479-108401500	0.002298701	
ENST00000514049	ENSG00000103599 (IQCH)	hsa-miR-146a-5p	0.811425
UTR3	15:67713783-67713808	0.018751338	
UTR3	15:67793315-67793329	0.003438897	
UTR3	15:67793772-67793794	0.012366219	
ENST00000392316	ENSG00000128641 (MYO1B)	hsa-miR-146a-5p	0.81075
UTR3	2:192289973-192289994	0.007750911	
UTR3	2:192290089-192290109	0.026733539	

ENST00000333407	ENSG00000133477 (FAM83F)	hsa-miR-146a-5p	0.810718
UTR3	22:40427908-40427936	0.001016567	
UTR3	22:40430198-40430223	0.00135714	
UTR3	22:40433631-40433649	0.004239232	
UTR3	22:40435201-40435220	0.003226278	
UTR3	22:40435575-40435594	0.001239826	
UTR3	22:40436463-40436479	0.002981752	
UTR3	22:40438200-40438228	0.002463448	
UTR3	22:40438445-40438463	0.012835697	
UTR3	22:40438539-40438555	0.0013258	
UTR3	22:40439481-40439504	0.003795279	
ENST00000469044	ENSG00000114098 (ARMC8)	hsa-miR-146a-5p	0.810457
UTR3	3:138015515-138015536	0.034453296	
ENST00000409711	ENSG00000071082 (RPL31)	hsa-miR-146a-5p	0.810425
UTR3	2:101621275-101621294	0.019571079	
UTR3	2:101622521-101622543	0.014878721	
ENST00000412050	ENSG00000107864 (CPEB3)	hsa-miR-146a-5p	0.809908
UTR3	10:93811861-93811880	0.007206258	
UTR3	10:93810100-93810120	0.002187882	
UTR3	10:93808715-93808733	0.003518094	
UTR3	10:93808320-93808344	0.006192437	
UTR3	10:93806645-93806664	0.010864799	
UTR3	10:93904786-93904811	0.011406407	
UTR3	10:93851646-93851668	0.012885785	
ENST00000555640	ENSG00000165792 (METTL17)	hsa-miR-146a-5p	0.809809
UTR3	14:21463056-21463083	0.003154288	
UTR3	14:21463341-21463366	0.005820414	
UTR3	14:21464281-21464306	0.002796868	
UTR3	14:21464693-21464714	0.02129171	
UTR3	14:21460260-21460276	0.007978054	
ENST00000312413	ENSG00000011021 (CLCN6)	hsa-miR-146a-5p	0.809612
UTR3	1:11893951-11893974	0.005850941	
UTR3	1:11900742-11900767	0.001516545	
UTR3	1:11903123-11903147	0.024956919	
UTR3	1:11882830-11882853	0.012026165	
ENST00000259154	ENSG00000136636 (KCTD3)	hsa-miR-146a-5p	0.809079
UTR3	1:215794540-215794565	0.004540572	
UTR3	1:215795081-215795104	0.026274943	
UTR3	1:215751014-215751035	0.019655202	
ENST00000608703	ENSG00000204389 (HSPA1A)	hsa-miR-146a-5p	0.808696
UTR3	6:31785512-31785538	0.034266568	
ENST00000493950	ENSG00000118492 (ADGB)	hsa-miR-146a-5p	0.8083
UTR3	6:146997491-146997519	0.004564742	

UTR3	6:147038722;147042539-147038735;147042544	0.023540276	
UTR3	6:147045342-147045363	0.000975903	
UTR3	6:147045565-147045581	0.001350422	
UTR3	6:147045794-147045807	0.001691989	
UTR3	6:147045817-147045832	0.000794179	
UTR3	6:147105911;147106746-147105930;147106753	0.001307287	
ENST00000351698	ENSG00000136827 (TOR1A)	hsa-miR-146a-5p	0.808176
UTR3	9:132575819-132575826	0.034211707	
ENST00000423591	ENSG00000188938 (FAM120AOS)	hsa-miR-146a-5p	0.807513
UTR3	9:96209771-96209791	0.008450432	
UTR3	9:96209303-96209325	0.009916228	
UTR3	9:96209027-96209055	0.012073602	
UTR3	9:96208970-96208988	0.003701613	
ENST00000336505	ENSG00000173611 (SCAI)	hsa-miR-146a-5p	0.807302
UTR3	9:127714797-127714818	0.005612537	
UTR3	9:127714622-127714642	0.003480565	
UTR3	9:127713242-127713265	0.000948215	
UTR3	9:127710808-127710822	0.00120627	
UTR3	9:127707066-127707080	0.002398881	
UTR3	9:127706412-127706430	0.007802349	
UTR3	9:127705404-127705418	0.00514937	
UTR3	9:127705001-127705020	0.007521476	
ENST00000585524	ENSG00000134042 (MRO)	hsa-miR-146a-5p	0.807072
UTR3	18:48326483-48326493	0.00613563	
UTR3	18:48325619-48325639	0.001514596	
UTR3	18:48325410-48325429	0.003975182	
UTR3	18:48325169-48325196	0.00311712	
UTR3	18:48324939-48324955	0.019353024	
ENST00000370483	ENSG00000014919 (COX15)	hsa-miR-146a-5p	0.806945
UTR3	10:101472031-101472051	0.016392187	
UTR3	10:101471820-101471843	0.017689976	
ENST00000542527	ENSG00000185716 (C16orf52)	hsa-miR-146a-5p	0.806875
UTR3	16:22092350-22092368	0.030686888	
UTR3	16:22095000-22095015	0.00338797	
ENST00000340106	ENSG00000242384 (TBC1D3H)	hsa-miR-146a-5p	0.806183
UTR3	17:34746453-34746471	0.034002386	
ENST00000523377	ENSG00000083099 (LYRM2)	hsa-miR-146a-5p	0.806008
UTR3	6:90345539-90345557	0.006140542	
UTR3	6:90342858-90342868	0.002949671	
UTR3	6:90342589-90342611	0.024893825	
ENST00000228865	ENSG00000111269 (CREBL2)	hsa-miR-146a-5p	0.805575
UTR3	12:12795370-12795395	0.024429945	

UTR3	12:12796321-12796340	0.001588396	
UTR3	12:12797656-12797678	0.002738456	
UTR3	12:12797914-12797930	0.005182072	
ENST00000585558	ENSG00000153822 (KCNJ16)	hsa-miR-146a-5p	0.805145
UTR3	17:68129633-68129656	0.031531036	
UTR3	17:68130229-68130251	0.002362973	
ENST00000244227	ENSG00000124380 (SNRNP27)	hsa-miR-146a-5p	0.804338
UTR3	2:70131526-70131545	0.029025064	
UTR3	2:70131973-70131987	0.004784998	
ENST00000251527	ENSG00000117868 (ESYT2)	hsa-miR-146a-5p	0.803535
UTR3	7:158526411-158526433	0.032044149	
UTR3	7:158525768-158525789	0.001682703	
ENST00000381854	ENSG00000106993 (CDC37L1)	hsa-miR-146a-5p	0.803127
UTR3	9:4706370-4706385	0.027901642	
UTR3	9:4706726-4706743	0.00504483	
UTR3	9:4679848-4679864	0.004440741	
ENST00000515655	ENSG00000118777 (ABCG2)	hsa-miR-146a-5p	0.802691
UTR3	4:89012455-89012475	0.002832751	
UTR3	4:89011547-89011560	0.029199285	
UTR3	4:89052315-89052340	0.009394266	
ENST00000360647	ENSG00000163848 (ZNF148)	hsa-miR-146a-5p	0.801928
UTR3	3:124950230-124950256	0.00504166	
UTR3	3:124949452-124949467	0.009013353	
UTR3	3:124948350-124948362	0.00183743	
UTR3	3:124946938-124946964	0.008322163	
UTR3	3:124946114-124946135	0.001994864	
UTR3	3:124952393-124952413	0.036334123	
ENST00000358823	ENSG00000118200 (CAMSAP2)	hsa-miR-146a-5p	0.801428
UTR3	1:200828090-200828107	0.025074353	
UTR3	1:200828632-200828659	0.004654824	
UTR3	1:200818011-200818026	0.020605511	
ENST00000392638	ENSG00000148814 (LRRC27)	hsa-miR-146a-5p	0.80105
UTR3	10:134191227-134191251	0.002930487	
UTR3	10:134191915-134191934	0.001350091	
UTR3	10:134192442-134192463	0.001188846	
UTR3	10:134192518-134192529	0.000400964	
UTR3	10:134192581-134192595	0.00045294	
UTR3	10:134192650-134192661	0.00040451	
UTR3	10:134192716-134192727	0.000406323	
UTR3	10:134192782-134192793	0.000646071	
UTR3	10:134192848-134192859	0.000416738	
UTR3	10:134192980-134192991	0.000897048	
UTR3	10:134192980-134192997	0.001572886	
UTR3	10:134194961-134194987	0.022803859	

ENST00000346248	ENSG00000182022 (CHST15)	hsa-miR-146a-5p	0.800751
UTR3	10:125768818-125768838	0.003204433	
UTR3	10:125768266-125768286	0.008572478	
UTR3	10:125767411-125767433	0.002705675	
UTR3	10:125767338-125767366	0.017636594	
UTR3	10:125804367-125804393	0.003043532	
UTR3	10:125804137-125804155	0.004696971	
ENST00000543630	ENSG00000086717 (PPEF1)	hsa-miR-146a-5p	0.800496
UTR3	X:18845418-18845445	0.033414056	
ENST00000418596	ENSG00000155744 (FAM126B)	hsa-miR-146a-5p	0.800482
UTR3	2:201845585-201845605	0.017169595	
UTR3	2:201845282-201845302	0.004812318	
UTR3	2:201844985-201844995	0.005636291	
UTR3	2:201841363-201841390	0.001324138	
UTR3	2:201839100-201839115	0.004470238	
ENST00000554908	ENSG00000197045 (GMFB)	hsa-miR-146a-5p	0.800375
UTR3	14:54946618-54946634	0.002032688	
UTR3	14:54942715-54942736	0.0113847	
UTR3	14:54942343-54942366	0.00369351	
UTR3	14:54941351-54941375	0.016290765	
ENST00000244020	ENSG00000124193 (SRSF6)	hsa-miR-146a-5p	0.800366
UTR3	20:42090530-42090552	0.009990153	
UTR3	20:42091598-42091610	0.011165082	
UTR3	20:42091877-42091896	0.012245467	
ENST00000366553	ENSG00000203668 (CHML)	hsa-miR-146a-5p	0.800147
UTR3	1:241797040-241797062	0.007759747	
UTR3	1:241795712-241795732	0.001850773	
UTR3	1:241795648-241795671	0.001945436	
UTR3	1:241794297-241794312	0.003100815	
UTR3	1:241794261-241794274	0.000908702	
UTR3	1:241793508-241793527	0.017812824	
ENST00000369331	ENSG00000165806 (CASP7)	hsa-miR-146a-5p	0.799681
UTR3	10:115489435-115489461	0.003901283	
UTR3	10:115489776-115489793	0.029429444	
ENST00000453386	ENSG00000136527 (TRA2B)	hsa-miR-146a-5p	0.799364
UTR3	3:185634035-185634062	0.010738691	
UTR3	3:185633805-185633832	0.022559763	
ENST00000294383	ENSG00000162402 (USP24)	hsa-miR-146a-5p	0.79929
UTR3	1:55534574-55534599	0.00378	
UTR3	1:55533997-55534009	0.005121792	
UTR3	1:55532948-55532959	0.001786562	
UTR3	1:55532439-55532463	0.01883046	
UTR3	1:55620352-55620368	0.009324735	
UTR3	1:55598345-55598361	0.011109797	
ENST00000467709	ENSG00000147459 (DOCK5)	hsa-miR-146a-5p	0.798886
UTR3	8:25222161-25222182	0.025380208	

UTR3	8:25224435-25224457	0.002822646	
UTR3	8:25250351-25250369	0.00237147	
UTR3	8:25269223-25269241	0.002675448	
ENST00000529084	ENSG00000140798 (ABCC12)	hsa-miR-146a-5p	0.79857
UTR3	16:48122519-48122534	0.002132746	
UTR3	16:48119558-48119585	0.006108519	
UTR3	16:48117855-48117879	0.019636209	
UTR3	16:48174822;48175117-48174831;48175129	0.027613133	
ENST00000371207	ENSG00000138185 (ENTPD1)	hsa-miR-146a-5p	0.79806
UTR3	10:97626715-97626739	0.020952474	
UTR3	10:97627826-97627853	0.002408102	
UTR3	10:97629589-97629598	0.002106478	
UTR3	10:97630696-97630722	0.001942588	
UTR3	10:97630737-97630754	0.003138774	
UTR3	10:97632180-97632207	0.001227857	
UTR3	10:97634232-97634251	0.000858445	
UTR3	10:97634342-97634369	0.000531118	
ENST00000273854	ENSG00000145242 (EPHA5)	hsa-miR-146a-5p	0.797077
UTR3	4:66187893-66187904	0.021495963	
UTR3	4:66186329-66186351	0.003184204	
UTR3	4:66186190-66186209	0.002314721	
UTR3	4:66185383-66185406	0.006071478	
ENST00000316660	ENSG00000141682 (PMAIP1)	hsa-miR-146a-5p	0.796965
UTR3	18:57570010-57570033	0.003198924	
UTR3	18:57570980-57570996	0.029856057	
ENST00000372687	ENSG00000156671 (SAMD8)	hsa-miR-146a-5p	0.796521
UTR3	10:76936180-76936193	0.020288469	
UTR3	10:76936349-76936359	0.00527722	
UTR3	10:76936507-76936520	0.00355433	
UTR3	10:76938002-76938023	0.002193215	
UTR3	10:76941161-76941185	0.001696951	
ENST00000360587	ENSG00000197361 (FBXL22)	hsa-miR-146a-5p	0.795943
UTR3	15:63893949-63893971	0.017833057	
UTR3	15:63894411-63894437	0.015118952	
ENST00000478199	ENSG00000162885 (B3GALNT2)	hsa-miR-146a-5p	0.795631
UTR3	1:235647737-235647755	0.01373484	
UTR3	1:235634175-235634194	0.019185797	
ENST00000440598	ENSG00000197008 (ZNF138)	hsa-miR-146a-5p	0.794882
UTR3	7:64276018;64291829-64276031;64291841	0.004359443	
UTR3	7:64293234-64293252	0.015200889	
UTR3	7:64293311-64293336	0.012238925	

UTR3	7:64275307-64275333	0.006069365	
ENST00000254605	ENSG00000132275 (RRP8)	hsa-miR-146a-5p	0.794735
UTR3	11:6619691-6619714	0.001391633	
UTR3	11:6618097-6618112	0.004717997	
UTR3	11:6617684-6617703	0.002656561	
UTR3	11:6617323-6617343	0.024064514	
ENST00000407780	ENSG00000160223 (ICOSLG)	hsa-miR-146a-5p	0.794277
UTR3	21:45648010-45648035	0.001252197	
UTR3	21:45644994-45645011	0.000789679	
UTR3	21:45643299-45643316	0.013107945	
UTR3	21:45643117-45643129	0.002441588	
UTR3	21:45642996-45643024	0.002396386	
UTR3	21:45642984-45642999	0.008114795	
UTR3	21:45642916-45642936	0.004682218	
ENST00000391884	ENSG00000182324 (KCNJ14)	hsa-miR-146a-5p	0.793783
UTR3	19:48968454-48968477	0.003501821	
UTR3	19:48969105-48969121	0.02497217	
UTR3	19:48967704-48967728	0.010993673	
UTR3	19:48967987-48968002	0.011412551	
ENST00000217289	ENSG00000101311 (FERMT1)	hsa-miR-146a-5p	0.793587
UTR3	20:6057529-6057553	0.010345656	
UTR3	20:6056769-6056794	0.002687995	
UTR3	20:6056399-6056415	0.01527335	
UTR3	20:6056006-6056030	0.002873177	
UTR3	20:6093145-6093160	0.008741098	
ENST00000467604	ENSG00000121578 (B4GALT4)	hsa-miR-146a-5p	0.793391
UTR3	3:118934903-118934925	0.02367734	
UTR3	3:118934455-118934477	0.00294538	
UTR3	3:118934427-118934452	0.001582977	
UTR3	3:118931724-118931744	0.001744108	
UTR3	3:118930997-118931013	0.002746548	
ENST00000373625	ENSG00000025800 (KPNA6)	hsa-miR-146a-5p	0.793314
UTR3	1:32638817-32638829	0.001857727	
UTR3	1:32639943-32639959	0.003815813	
UTR3	1:32639956-32639983	0.000778364	
UTR3	1:32640971-32640987	0.001778075	
UTR3	1:32641876-32641904	0.005061106	
UTR3	1:32641954-32641975	0.019397565	
ENST00000225983	ENSG00000108840 (HDAC5)	hsa-miR-146a-5p	0.79306
UTR3	17:42155016-42155028	0.004340476	
UTR3	17:42154230-42154252	0.025987229	
UTR3	17:42164097;42164780-42164105;42164796	0.005803407	
UTR3	17:42157560-42157581	0.00715405	

ENST00000396981	ENSG00000132388 (UBE2G1)	hsa-miR-146a-5p	0.792305
UTR3	17:4175255-4175273	0.01720093	
UTR3	17:4174831-4174852	0.003536714	
UTR3	17:4173279-4173299	0.00210752	
UTR3	17:4173041-4173063	0.00974311	
ENST00000355459	ENSG00000198390 (KRTAP13-1)	hsa-miR-146a-5p	0.791614
UTR3	21:31769024-31769044	0.032519754	
ENST00000436544	ENSG00000163803 (PLB1)	hsa-miR-146a-5p	0.790442
UTR3	2:28817217-28817232	0.005300229	
UTR3	2:28817245-28817261	0.024005107	
UTR3	2:28817446-28817472	0.003098494	
ENST00000366624	ENSG00000143674 (MLK4)	hsa-miR-146a-5p	0.790407
UTR3	1:233518734-233518760	0.019230497	
UTR3	1:233519048-233519075	0.013169906	
ENST00000423035	ENSG00000197121 (PGAP1)	hsa-miR-146a-5p	0.790173
UTR3	2:197784755-197784779	0.022302573	
UTR3	2:197729715-197729740	0.001737863	
UTR3	2:197704417-197704434	0.001106659	
UTR3	2:197703814-197703842	0.00161561	
UTR3	2:197703442-197703460	0.001747779	
UTR3	2:197702039-197702056	0.001717825	
UTR3	2:197701652-197701676	0.002149048	
ENST00000548411	ENSG00000151746 (BICD1)	hsa-miR-146a-5p	0.790171
UTR3	12:32532605-32532616	0.001157788	
UTR3	12:32533047-32533067	0.002619715	
UTR3	12:32534450-32534472	0.00422622	
UTR3	12:32535634-32535651	0.00459224	
UTR3	12:32536241-32536266	0.019781213	
ENST00000274459	ENSG00000145782 (ATG12)	hsa-miR-146a-5p	0.78979
UTR3	5:115167179-115167195	0.028945651	
UTR3	5:115166121-115166140	0.001621194	
UTR3	5:115165026-115165052	0.001772738	
ENST00000592780	ENSG00000256294 (ZNF225)	hsa-miR-146a-5p	0.789555
UTR3	19:44635116-44635141	0.027657062	
UTR3	19:44636001-44636020	0.001884249	
UTR3	19:44636331-44636356	0.002775217	
ENST00000396373	ENSG00000139083 (ETV6)	hsa-miR-146a-5p	0.789387
UTR3	12:12047232-12047255	0.022345914	
UTR3	12:12048243-12048255	0.009954017	
ENST00000545826	ENSG00000168769 (TET2)	hsa-miR-146a-5p	0.789295
UTR3	4:106196925-106196949	0.002291143	
UTR3	4:106197265-106197284	0.002675414	
UTR3	4:106197722-106197745	0.014317461	
UTR3	4:106158376-106158403	0.026515474	
UTR3	4:106164037-106164062	0.027803408	

ENST00000301919	ENSG00000170903 (MSANTD4)	hsa-miR-146a-5p	0.788917
UTR3	11:105880211-105880228	0.006236186	
UTR3	11:105879957-105879972	0.021967007	
UTR3	11:105879576-105879598	0.002515412	
UTR3	11:105879503-105879527	0.001535181	
ENST00000199320	ENSG00000086189 (DIMT1)	hsa-miR-146a-5p	0.788691
UTR3	5:61684334-61684340	0.003895811	
UTR3	5:61683481-61683495	0.002358427	
UTR3	5:61683123-61683143	0.025977343	
ENST00000508296	ENSG00000182230 (FAM153B)	hsa-miR-146a-5p	0.788363
UTR3	5:175547885-175547912	0.001410119	
UTR3	5:175548749-175548769	0.016404945	
UTR3	5:175552791-175552811	0.009939724	
UTR3	5:175553052-175553075	0.004444682	
ENST00000343776	ENSG00000181450 (ZNF678)	hsa-miR-146a-5p	0.788306
UTR3	1:227844847-227844868	0.001411325	
UTR3	1:227845589-227845617	0.001705863	
UTR3	1:227845656-227845677	0.000915698	
UTR3	1:227846289-227846308	0.001496296	
UTR3	1:227846289-227846316	0.000538665	
UTR3	1:227847602-227847607	0.002471476	
UTR3	1:227848675-227848692	0.001296164	
UTR3	1:227848927-227848942	0.001675199	
UTR3	1:227842599-227842617	0.014931939	
UTR3	1:227842683-227842701	0.016890148	
UTR3	1:227843096-227843121	0.02368399	
UTR3	1:227843355-227843373	0.018067396	
ENST00000531224	ENSG00000029363 (BCLAF1)	hsa-miR-146a-5p	0.786736
UTR3	6:136581529-136581547	0.004846552	
UTR3	6:136578908-136578926	0.001669199	
UTR3	6:136578778-136578802	0.018482407	
UTR3	6:136600940-136600964	0.010111164	
UTR3	6:136589406-136589426	0.023612824	
ENST00000272217	ENSG00000143862 (ARL8A)	hsa-miR-146a-5p	0.786392
UTR3	1:202103382-202103406	0.003178564	
UTR3	1:202102761-202102784	0.028828498	
ENST00000361752	ENSG00000112531 (QKI)	hsa-miR-146a-5p	0.786332
UTR3	6:163992133-163992154	0.005361624	
UTR3	6:163993552-163993579	0.002089316	
UTR3	6:163994244-163994267	0.010371853	
UTR3	6:163995782-163995799	0.00761089	
UTR3	6:163995962-163995975	0.00101077	
UTR3	6:163999561-163999584	0.005556766	
ENST00000434724	ENSG00000103342 (GSPT1)	hsa-miR-146a-5p	0.786138
UTR3	16:11966817-11966841	0.014792598	

UTR3	16:11964549-11964574	0.008569907	
UTR3	16:11964312-11964329	0.00188468	
UTR3	16:11962760-11962774	0.004784729	
UTR3	16:11962438-11962459	0.001950414	
ENST00000394252	ENSG00000170439 (METTL7B)	hsa-miR-146a-5p	0.786106
UTR3	12:56078335-56078362	0.029783908	
UTR3	12:56077729-56077751	0.011985331	
ENST00000574951	ENSG00000108509 (CAMTA2)	hsa-miR-146a-5p	0.785389
UTR3	17:4884609-4884627	0.02027205	
UTR3	17:4883473-4883491	0.00380982	
UTR3	17:4872263;4872445-4872276;4872455	0.002864043	
UTR3	17:4872031-4872052	0.001654475	
UTR3	17:4871386-4871404	0.003309232	
ENST00000488550	ENSG00000143995 (MEIS1)	hsa-miR-146a-5p	0.785228
UTR3	2:66796057-66796083	0.00673039	
UTR3	2:66796326-66796350	0.007606194	
UTR3	2:66797828-66797852	0.016552362	
UTR3	2:66798262-66798288	0.001005052	
ENST00000601773	ENSG00000160321 (ZNF208)	hsa-miR-146a-5p	0.785072
UTR3	19:22122904-22122919	0.014799106	
UTR3	19:22122675-22122703	0.017079764	
ENST00000398844	ENSG00000113615 (SEC24A)	hsa-miR-146a-5p	0.784522
UTR3	5:134060879-134060906	0.019176859	
UTR3	5:134061114-134061128	0.004695416	
UTR3	5:134062733-134062753	0.00480242	
UTR3	5:133996936-133996950	0.016661386	
ENST00000449622	ENSG00000160221 (C21orf33)	hsa-miR-146a-5p	0.784354
UTR3	21:45564953-45564969	0.003070965	
UTR3	21:45565044-45565067	0.002458611	
UTR3	21:45565422-45565441	0.026279919	
ENST00000588388	ENSG00000131941 (RHPN2)	hsa-miR-146a-5p	0.784007
UTR3	19:33484917-33484928	0.01217738	
UTR3	19:33470925-33470947	0.00447251	
UTR3	19:33470803-33470821	0.01216308	
UTR3	19:33470787-33470802	0.002963009	
ENST00000323816	ENSG00000007237 (GAS7)	hsa-miR-146a-5p	0.78372
UTR3	17:9819999-9820019	0.001889811	
UTR3	17:9819725-9819753	0.0250281	
UTR3	17:9815638-9815664	0.001388028	
UTR3	17:9885172-9885198	0.00986756	
UTR3	17:9837490-9837502	0.00814599	
ENST00000366910	ENSG00000186205 (MARC1)	hsa-miR-146a-5p	0.783695
UTR3	1:220986788-220986809	0.025819244	

UTR3	1:220987229-220987251	0.002806726	
UTR3	1:220987340-220987356	0.003119829	
ENST00000367701	ENSG00000185278 (ZBTB37)	hsa-miR-146a-5p	0.783595
UTR3	1:173858183-173858200	0.00228515	
UTR3	1:173858643-173858668	0.001273208	
UTR3	1:173858801-173858821	0.001397985	
UTR3	1:173863654-173863666	0.001351184	
UTR3	1:173868348-173868364	0.002863164	
UTR3	1:173869126-173869151	0.001467548	
UTR3	1:173869489-173869507	0.001791897	
UTR3	1:173870384-173870409	0.004704759	
UTR3	1:173871031-173871044	0.002600582	
UTR3	1:173871153-173871178	0.001475049	
UTR3	1:173872248-173872261	0.004746865	
UTR3	1:173872261-173872284	0.005778841	
ENST00000374690	ENSG00000169083 (AR)	hsa-miR-146a-5p	0.783528
UTR3	X:66944625-66944646	0.004070186	
UTR3	X:66944728-66944755	0.001031808	
UTR3	X:66944801-66944815	0.001405671	
UTR3	X:66945221-66945243	0.00905965	
UTR3	X:66945765-66945780	0.001941879	
UTR3	X:66946542-66946558	0.001323751	
UTR3	X:66947785-66947811	0.006607271	
UTR3	X:66950155-66950174	0.00628956	
ENST00000295049	ENSG00000162944 (RFTN2)	hsa-miR-146a-5p	0.783372
UTR3	2:198436363-198436384	0.028462722	
UTR3	2:198435002-198435024	0.001580439	
UTR3	2:198434341-198434364	0.001671552	
ENST00000339613	ENSG00000124440 (HIF3A)	hsa-miR-146a-5p	0.782943
UTR3	19:46843124-46843148	0.014072197	
UTR3	19:46844388-46844406	0.005290508	
UTR3	19:46844797-46844818	0.007277617	
UTR3	19:46845234-46845256	0.005033146	
ENST00000376621	ENSG00000204590 (GNL1)	hsa-miR-146a-5p	0.782601
UTR3	6:30513624-30513638	0.016893899	
UTR3	6:30513602-30513626	0.009393176	
UTR3	6:30513180-30513194	0.005353484	
ENST00000262032	ENSG00000123411 (IKZF4)	hsa-miR-146a-5p	0.782453
UTR3	12:56429821-56429841	0.019352813	
UTR3	12:56429961-56429989	0.003664538	
UTR3	12:56430824-56430840	0.002616798	
UTR3	12:56432046-56432064	0.005992206	
ENST00000437887	ENSG00000233863 (AC012215.1)	hsa-miR-146a-5p	0.782242
UTR3	8:35651347-35651363	0.014314424	
UTR3	8:35651477-35651497	0.006533456	
UTR3	8:35651765-35651788	0.004819132	

UTR3	8:35653295-35653318	0.00593908	
ENST00000395076	ENSG00000100614 (PPM1A)	hsa-miR-146a-5p	0.782121
UTR3	14:60762033-60762052	0.015023491	
UTR3	14:60762574-60762594	0.001465463	
UTR3	14:60764246-60764273	0.00232132	
UTR3	14:60765638-60765658	0.009758521	
UTR3	14:60749598-60749618	0.015951123	
ENST00000318833	ENSG00000180787 (ZFP3)	hsa-miR-146a-5p	0.781574
UTR3	17:4997401-4997421	0.018422526	
UTR3	17:4998700-4998720	0.002202589	
UTR3	17:4998930-4998951	0.010916968	
ENST00000275493	ENSG00000146648 (EGFR)	hsa-miR-146a-5p	0.781509
UTR3	7:55274208-55274236	0.00205656	
UTR3	7:55275853-55275868	0.015314033	
UTR3	7:55276483-55276509	0.005169866	
UTR3	7:55276553-55276571	0.000579984	
UTR3	7:55277443-55277467	0.005169352	
UTR3	7:55278271-55278296	0.001655944	
UTR3	7:55278364-55278378	0.001590065	
ENST00000554401	ENSG00000080824 (HSP90AA1)	hsa-miR-146a-5p	0.781425
UTR3	14:102550143-102550158	0.031527834	
ENST00000354631	ENSG00000107614 (TRDMT1)	hsa-miR-146a-5p	0.78104
UTR3	10:17190319-17190338	0.001088942	
UTR3	10:17188624-17188641	0.001974097	
UTR3	10:17187998-17188025	0.004122753	
UTR3	10:17186587-17186598	0.016369666	
UTR3	10:17186407-17186428	0.002859177	
UTR3	10:17185776-17185803	0.001777648	
UTR3	10:17185140-17185158	0.003298698	
ENST00000290585	ENSG00000159409 (CELF3)	hsa-miR-146a-5p	0.780985
UTR3	1:151674963-151674989	0.031485763	
ENST00000528670	ENSG00000187240 (DYNC2H1)	hsa-miR-146a-5p	0.780782
UTR3	11:103194699-103194717	0.031466326	
ENST00000359486	ENSG00000100207 (TCF20)	hsa-miR-146a-5p	0.780637
UTR3	22:42557229-42557256	0.031452469	
ENST00000409284	ENSG00000163492 (CCDC141)	hsa-miR-146a-5p	0.780168
UTR3	2:179742591-179742610	0.012264997	
UTR3	2:179742576-179742589	0.008793196	
UTR3	2:179741848-179741870	0.001866353	
UTR3	2:179741261-179741284	0.002349312	
UTR3	2:179741030-179741049	0.001932964	
UTR3	2:179740787-179740806	0.001562389	
UTR3	2:179739377-179739398	0.002638566	

ENST00000400085	ENSG00000215343 (ZNF705D)	hsa-miR-146a-5p	0.779144
UTR3	8:11971586-11971612	0.002332187	
UTR3	8:11972070-11972092	0.002494284	
UTR3	8:11972464-11972487	0.024872516	
UTR3	8:11970378-11970405	0.008775052	
ENST00000503808	ENSG00000157426 (AASDH)	hsa-miR-146a-5p	0.779109
UTR3	4:57237647-57237671	0.004763025	
UTR3	4:57221466-57221485	0.012582883	
UTR3	4:57216071-57216091	0.002585563	
UTR3	4:57204944;57209001-57204957;57209014	0.011375552	
ENST00000374525	ENSG00000241978 (AKAP2)	hsa-miR-146a-5p	0.778869
UTR3	9:112932321-112932348	0.003723278	
UTR3	9:112932886-112932911	0.001645306	
UTR3	9:112932993-112933012	0.000760435	
UTR3	9:112934143-112934165	0.005426306	
UTR3	9:112934736-112934760	0.015458723	
UTR3	9:112899870-112899894	0.021564409	
ENST00000340652	ENSG00000188730 (VWC2)	hsa-miR-146a-5p	0.778436
UTR3	7:49952411-49952426	0.003035178	
UTR3	7:49953587-49953615	0.005558946	
UTR3	7:49955069-49955079	0.001605012	
UTR3	7:49955659-49955677	0.006608128	
UTR3	7:49957254-49957274	0.002251633	
UTR3	7:49957730-49957744	0.005225367	
UTR3	7:49958148-49958172	0.001610643	
UTR3	7:49960181-49960209	0.001545764	
UTR3	7:49960982-49960996	0.003802536	
ENST00000423275	ENSG00000132155 (RAF1)	hsa-miR-146a-5p	0.77825
UTR3	3:12645783;12647700-12645788;12647716	0.019419684	
UTR3	3:12627217-12627234	0.009805652	
UTR3	3:12626640-12626661	0.002000189	
ENST00000374530	ENSG00000157654 (PALM2-AKAP2)	hsa-miR-146a-5p	0.778177
UTR3	9:112932321-112932348	0.003769924	
UTR3	9:112932886-112932911	0.001635708	
UTR3	9:112932993-112933012	0.000759281	
UTR3	9:112934143-112934165	0.005497444	
UTR3	9:112934736-112934760	0.015377568	
UTR3	9:112899870-112899894	0.021117441	
ENST00000240922	ENSG00000121579 (NAA50)	hsa-miR-146a-5p	0.777265
UTR3	3:113440212-113440234	0.006491922	
UTR3	3:113439213-113439241	0.006504448	
UTR3	3:113436536-113436556	0.003578365	
UTR3	3:113436421-113436448	0.00124722	

UTR3	3:113436248-113436274	0.002170259	
UTR3	3:113436233-113436248	0.011140216	
ENST00000302103	ENSG00000172461 (FUT9)	hsa-miR-146a-5p	0.776879
UTR3	6:96652702-96652726	0.003394851	
UTR3	6:96652740-96652765	0.003006036	
UTR3	6:96653800-96653816	0.001264878	
UTR3	6:96656844-96656858	0.001345893	
UTR3	6:96657138-96657158	0.001983625	
UTR3	6:96657842-96657861	0.009713825	
UTR3	6:96658413-96658436	0.004118123	
UTR3	6:96659746-96659770	0.002962461	
UTR3	6:96663163-96663178	0.0033063	
ENST00000295851	ENSG00000138443 (ABI2)	hsa-miR-146a-5p	0.776831
UTR3	2:204292376-204292403	0.005363376	
UTR3	2:204295997-204296004	0.004177317	
UTR3	2:204297381-204297408	0.000959874	
UTR3	2:204299480-204299506	0.002076199	
UTR3	2:204301980-204302004	0.001881069	
UTR3	2:204303017-204303043	0.001005082	
UTR3	2:204303664-204303691	0.002065638	
UTR3	2:204307358-204307382	0.002656673	
UTR3	2:204307923-204307936	0.001737389	
UTR3	2:204310882-204310898	0.001032072	
UTR3	2:204311271-204311291	0.003130079	
UTR3	2:204311454-204311471	0.002610166	
UTR3	2:204312010-204312032	0.002396586	
ENST00000423760	ENSG00000106443 (PHF14)	hsa-miR-146a-5p	0.776225
UTR3	7:11209201-11209224	0.031034444	
ENST00000462833	ENSG00000173175 (ADCY5)	hsa-miR-146a-5p	0.775935
UTR3	3:123002783-123002789	0.005820464	
UTR3	3:123001748-123001772	0.005678369	
UTR3	3:123001455-123001471	0.014066476	
UTR3	3:123001357-123001380	0.002899947	
UTR3	3:123071410;123166259-123071428;123166260	0.013361539	
ENST00000313349	ENSG00000179833 (SERTAD2)	hsa-miR-146a-5p	0.775828
UTR3	2:64862995-64863010	0.017793496	
UTR3	2:64862070-64862095	0.003950069	
UTR3	2:64860915-64860939	0.003986255	
UTR3	2:64860483-64860499	0.002580131	
UTR3	2:64859848-64859871	0.002687118	
ENST00000380607	ENSG00000107295 (SH3GL2)	hsa-miR-146a-5p	0.775498
UTR3	9:17795989-17796011	0.030966068	
ENST00000233202	ENSG00000018280 (SLC11A1)	hsa-miR-146a-5p	0.775257
UTR3	2:219261589-219261613	0.030943462	

ENST00000529131	ENSG00000204711 (C9orf135)	hsa-miR-146a-5p	0.774777
UTR3	9:72520899-72520922	0.030898469	
ENST00000413659	ENSG00000128815 (WDFY4)	hsa-miR-146a-5p	0.774634
UTR3	10:50030518-50030534	0.001465835	
UTR3	10:50030578;50034716-50030582;50034738	0.001758598	
UTR3	10:50034816-50034842	0.000798382	
UTR3	10:50083137-50083165	0.002852473	
UTR3	10:50098626-50098640	0.003035366	
UTR3	10:50190713-50190740	0.020974431	
ENST00000306829	ENSG00000171914 (TLN2)	hsa-miR-146a-5p	0.77436
UTR3	15:63132929-63132949	0.007240556	
UTR3	15:63133097-63133119	0.016735288	
UTR3	15:63135024-63135046	0.004436258	
UTR3	15:63135388-63135403	0.00106223	
UTR3	15:63135803-63135829	0.001385116	
ENST00000503275	ENSG00000069018 (TRPC7)	hsa-miR-146a-5p	0.774283
UTR3	5:135601943-135601957	0.024018092	
UTR3	5:135567126-135567143	0.006834178	
ENST00000310903	ENSG00000174527 (MYO1H)	hsa-miR-146a-5p	0.773657
UTR3	12:109885343-109885362	0.02773355	
UTR3	12:109885417-109885440	0.003060225	
ENST00000360409	ENSG00000118257 (NRP2)	hsa-miR-146a-5p	0.773651
UTR3	2:206659997-206660023	0.00415494	
UTR3	2:206660728-206660756	0.00210191	
UTR3	2:206660787-206660804	0.000942388	
UTR3	2:206661760-206661783	0.006703829	
UTR3	2:206662129-206662147	0.016890144	
ENST00000447079	ENSG00000167258 (CDK12)	hsa-miR-146a-5p	0.773512
UTR3	17:37687606-37687628	0.012027335	
UTR3	17:37688511-37688537	0.008469247	
UTR3	17:37689062-37689082	0.001760164	
UTR3	17:37690884-37690898	0.005073362	
UTR3	17:37682424-37682448	0.017573589	
ENST00000360264	ENSG00000257923 (CUX1)	hsa-miR-146a-5p	0.773137
UTR3	7:101900512-101900534	0.002641667	
UTR3	7:101901365-101901386	0.027407677	
UTR3	7:101840024-101840047	0.003828768	
ENST00000334295	ENSG00000134779 (TPGS2)	hsa-miR-146a-5p	0.77267
UTR3	18:34374350-34374365	0.02477679	
UTR3	18:34374141-34374158	0.00592502	
ENST00000359660	ENSG00000145743 (FBXL17)	hsa-miR-146a-5p	0.772496
UTR3	5:107196335-107196354	0.013389599	
UTR3	5:107195034-107195061	0.017295973	
ENST00000339249	ENSG00000173275 (ZNF449)	hsa-miR-146a-5p	0.772179
UTR3	X:134496188-134496201	0.002674348	

UTR3	X:134496309-134496322	0.002778763	
UTR3	X:134496368-134496386	0.012707382	
UTR3	X:134496412-134496435	0.002153008	
UTR3	X:134496590-134496605	0.010342594	
ENST00000271411	ENSG00000143190 (POU2F1)	hsa-miR-146a-5p	0.771974
UTR3	1:167386118-167386136	0.003169494	
UTR3	1:167386426-167386450	0.001564091	
UTR3	1:167387788-167387809	0.001751984	
UTR3	1:167388800-167388820	0.002342305	
UTR3	1:167390092-167390113	0.001645663	
UTR3	1:167390388-167390404	0.001148715	
UTR3	1:167390656-167390682	0.001733045	
UTR3	1:167390875-167390889	0.001823769	
UTR3	1:167391641-167391660	0.001729229	
UTR3	1:167393062-167393082	0.013728812	
ENST00000510994	ENSG00000129055 (ANAPC13)	hsa-miR-146a-5p	0.771533
UTR3	3:134197398-134197417	0.003827852	
UTR3	3:134197257-134197277	0.00407199	
UTR3	3:134197032-134197058	0.005429311	
UTR3	3:134196864-134196881	0.004364537	
UTR3	3:134196787-134196814	0.003293384	
UTR3	3:134196645-134196665	0.003467471	
UTR3	3:134196555-134196582	0.006141597	
ENST00000260526	ENSG00000137962 (ARHGAP29)	hsa-miR-146a-5p	0.771282
UTR3	1:94637384-94637399	0.02868194	
UTR3	1:94697001-94697013	0.010000997	
ENST00000263102	ENSG00000108091 (CCDC6)	hsa-miR-146a-5p	0.771099
UTR3	10:61551788-61551813	0.002892418	
UTR3	10:61550926-61550942	0.017144803	
UTR3	10:61550748-61550765	0.010518693	
ENST00000559291	ENSG00000128891 (C15orf57)	hsa-miR-146a-5p	0.770473
UTR3	15:40829192-40829212	0.002180053	
UTR3	15:40829057-40829075	0.00107606	
UTR3	15:40826901-40826916	0.007526304	
UTR3	15:40826715-40826734	0.019715589	
ENST00000264434	ENSG00000115998 (C2orf42)	hsa-miR-146a-5p	0.770391
UTR3	2:70377356-70377375	0.005871769	
UTR3	2:70377046-70377070	0.024618661	
ENST00000367975	ENSG00000143252 (SDHC)	hsa-miR-146a-5p	0.770355
UTR3	1:161332297-161332317	0.025312837	
UTR3	1:161332903-161332927	0.00517426	

ENST00000426126	ENSG00000186908 (ZDHHC17)	hsa-miR-146a-5p	0.770253
UTR3	12:77244820-77244834	0.002807948	
UTR3	12:77245020-77245044	0.019001805	
UTR3	12:77245630-77245651	0.003611749	
UTR3	12:77245664-77245691	0.001341103	
UTR3	12:77247165-77247187	0.003715118	
ENST00000335397	ENSG00000186509 (OR9Q1)	hsa-miR-146a-5p	0.769706
UTR3	11:57948511-57948529	0.020071187	
UTR3	11:57948623-57948638	0.010356075	
ENST00000570137	ENSG00000166446 (CDYL2)	hsa-miR-146a-5p	0.769505
UTR3	16:80637360-80637382	0.005581081	
UTR3	16:80636505-80636523	0.005913208	
UTR3	16:80635053-80635071	0.001199073	
UTR3	16:80634868-80634884	0.002013479	
UTR3	16:80633965-80633978	0.002244725	
UTR3	16:80632358-80632384	0.008829571	
UTR3	16:80632031-80632042	0.004627559	
ENST00000392837	ENSG00000111785 (RIC8B)	hsa-miR-146a-5p	0.769218
UTR3	12:107280432-107280448	0.017400079	
UTR3	12:107236394-107236419	0.021152499	
UTR3	12:107237647-107237666	0.030868985	
ENST00000452722	ENSG00000182985 (CADM1)	hsa-miR-146a-5p	0.769147
UTR3	11:115046172-115046192	0.003246481	
UTR3	11:115045929-115045942	0.008070384	
UTR3	11:115045900-115045913	0.003804404	
UTR3	11:115044149-115044173	0.002383755	
UTR3	11:115043879-115043893	0.004582884	
UTR3	11:115043810-115043830	0.002685952	
UTR3	11:115040828-115040841	0.005601838	
ENST00000361905	ENSG00000187079 (TEAD1)	hsa-miR-146a-5p	0.768991
UTR3	11:12959157-12959175	0.022237818	
UTR3	11:12964780-12964807	0.001822843	
UTR3	11:12965610-12965627	0.00316793	
UTR3	11:12965790-12965805	0.003132832	
ENST00000403299	ENSG00000100065 (CARD10)	hsa-miR-146a-5p	0.768959
UTR3	22:37886884-37886902	0.004705371	
UTR3	22:37886850-37886876	0.018775482	
UTR3	22:37886636-37886658	0.006877565	
ENST00000205636	ENSG00000091317 (CMTM6)	hsa-miR-146a-5p	0.768325
UTR3	3:32523859-32523884	0.002210182	
UTR3	3:32523798-32523816	0.005038224	
UTR3	3:32523008-32523024	0.022052681	
UTR3	3:32544114-32544136	0.005378228	
ENST00000376447	ENSG00000165105 (RASEF)	hsa-miR-146a-5p	0.768037
UTR3	9:85597173-85597182	0.004214675	

UTR3	9:85596948-85596972	0.005809627	
UTR3	9:85595194-85595211	0.020249421	
ENST00000525508	ENSG00000171316 (CHD7)	hsa-miR-146a-5p	0.76792
UTR3	8:61736940-61736965	0.003791536	
UTR3	8:61737175-61737196	0.024979303	
UTR3	8:61654855-61654875	0.007911839	
ENST00000297354	ENSG00000164764 (SBSPON)	hsa-miR-146a-5p	0.767572
UTR3	8:73979524-73979539	0.004612662	
UTR3	8:73979420-73979439	0.025618414	
ENST00000400075	ENSG00000154727 (GABPA)	hsa-miR-146a-5p	0.767181
UTR3	21:27141970-27141991	0.002700943	
UTR3	21:27142879-27142903	0.002017218	
UTR3	21:27143164-27143173	0.00604562	
UTR3	21:27143610-27143634	0.002136727	
UTR3	21:27143700-27143715	0.001637223	
UTR3	21:27143712-27143739	0.007859008	
UTR3	21:27144531-27144551	0.007798559	
ENST00000466490	ENSG00000144867 (SRPRB)	hsa-miR-146a-5p	0.766872
UTR3	3:133538784-133538795	0.016196474	
UTR3	3:133539445-133539464	0.008554979	
UTR3	3:133539831-133539857	0.005415562	
ENST00000591604	ENSG00000167383 (ZNF229)	hsa-miR-146a-5p	0.766841
UTR3	19:44934554-44934577	0.01118548	
UTR3	19:44933356-44933371	0.001191958	
UTR3	19:44933023-44933038	0.001578209	
UTR3	19:44931957-44931973	0.003030857	
UTR3	19:44931274-44931288	0.005287492	
UTR3	19:44931258-44931277	0.007890238	
ENST00000474541	ENSG00000135747 (ZNF670)	hsa-miR-146a-5p	0.766534
UTR3	1:247132707-247132728	0.006414973	
UTR3	1:247131172-247131187	0.006195642	
UTR3	1:247131155-247131175	0.017525545	
ENST00000366862	ENSG00000143756 (FBXO28)	hsa-miR-146a-5p	0.765962
UTR3	1:224346067-224346082	0.017228917	
UTR3	1:224347277-224347299	0.000736263	
UTR3	1:224348852-224348871	0.010053088	
UTR3	1:224345149-224345176	0.010723444	
ENST00000479862	ENSG00000165078 (CPA6)	hsa-miR-146a-5p	0.76581
UTR3	8:68421795-68421819	0.008134097	
UTR3	8:68334368-68334391	0.021936041	
ENST00000351193	ENSG00000169251 (NMD3)	hsa-miR-146a-5p	0.765506
UTR3	3:160969991-160970008	0.030042383	
ENST00000309964	ENSG00000134852 (CLOCK)	hsa-miR-146a-5p	0.765352
UTR3	4:56298619-56298636	0.001790034	
UTR3	4:56296279-56296286	0.005757977	

UTR3	4:56296207-56296228	0.000719194	
UTR3	4:56294864-56294878	0.007946682	
UTR3	4:56294583-56294600	0.004444244	
UTR3	4:56345078-56345102	0.040373706	
ENST00000296755	ENSG00000131711 (MAP1B)	hsa-miR-146a-5p	0.765075
UTR3	5:71501200-71501228	0.006459613	
UTR3	5:71501564-71501591	0.003555071	
UTR3	5:71502309-71502333	0.005129667	
UTR3	5:71502492-71502516	0.001856105	
UTR3	5:71502884-71502899	0.001588414	
UTR3	5:71503898-71503911	0.005895966	
UTR3	5:71504300-71504322	0.002173042	
UTR3	5:71504331-71504351	0.001220931	
UTR3	5:71493627-71493642	0.010985216	
ENST00000383699	ENSG00000175161 (CADM2)	hsa-miR-146a-5p	0.764683
UTR3	3:86117484-86117493	0.022375851	
UTR3	3:86120669-86120680	0.00410935	
UTR3	3:86010761-86010788	0.017337678	
ENST00000318709	ENSG00000105778 (AVL9)	hsa-miR-146a-5p	0.764284
UTR3	7:32625069-32625087	0.001983241	
UTR3	7:32627371-32627391	0.002142464	
UTR3	7:32627737-32627764	0.007831366	
UTR3	7:32628146-32628162	0.01352906	
UTR3	7:32598552-32598574	0.009457523	
UTR3	7:32619864-32619882	0.012099581	
ENST00000298573	ENSG00000111266 (DUSP16)	hsa-miR-146a-5p	0.76421
UTR3	12:12630428-12630444	0.02992465	
ENST00000314099	ENSG00000236761 (CTAGE9)	hsa-miR-146a-5p	0.764028
UTR3	6:132029649-132029672	0.004437528	
UTR3	6:132029619-132029636	0.025470596	
ENST00000261486	ENSG00000129595 (EPB41L4A)	hsa-miR-146a-5p	0.763539
UTR3	5:111500009-111500030	0.010721261	
UTR3	5:111499642-111499657	0.006677655	
UTR3	5:111498489-111498512	0.00431172	
UTR3	5:111498361-111498379	0.006591148	
UTR3	5:111504703-111504727	0.008173174	
ENST00000265350	ENSG00000124641 (MED20)	hsa-miR-146a-5p	0.762941
UTR3	6:41874680-41874700	0.005563161	
UTR3	6:41874150-41874172	0.002774452	
UTR3	6:41873578-41873605	0.008642528	
UTR3	6:41873177-41873204	0.012829648	
ENST00000514215	ENSG00000198780 (FAM169A)	hsa-miR-146a-5p	0.762843

	5:74100421;74100981-		
UTR3	74100430;74100994	0.008306232	
UTR3	5:74096756-74096778	0.021494683	
ENST00000379261	ENSG00000048740 (CELF2)	hsa-miR-146a-5p	0.762791
UTR3	10:11371947-11371964	0.016522875	
UTR3	10:11372517-11372540	0.004760932	
UTR3	10:11372929-11372956	0.00319109	
UTR3	10:11374027-11374044	0.001819461	
UTR3	10:11376812-11376831	0.003501888	
ENST00000174653	ENSG00000070718 (AP3M2)	hsa-miR-146a-5p	0.762791
UTR3	8:42026774-42026795	0.027522974	
UTR3	8:42028297-42028311	0.002273209	
ENST00000216218	ENSG00000100380 (ST13)	hsa-miR-146a-5p	0.762736
UTR3	22:41222443-41222467	0.008692053	
UTR3	22:41221845-41221861	0.008197398	
UTR3	22:41220743-41220757	0.012901748	
ENST00000318445	ENSG00000176463 (SLCO3A1)	hsa-miR-146a-5p	0.762733
UTR3	15:92706501-92706524	0.004750478	
UTR3	15:92706967-92706993	0.007251796	
UTR3	15:92707355-92707381	0.008253502	
UTR3	15:92708521-92708541	0.003637618	
UTR3	15:92708930-92708940	0.005897565	
ENST00000582730	ENSG00000136450 (SRSF1)	hsa-miR-146a-5p	0.761149
UTR3	17:56082720-56082742	0.029648254	
ENST00000371130	ENSG00000009694 (TENM1)	hsa-miR-146a-5p	0.760123
UTR3	X:123514056-123514069	0.006382576	
UTR3	X:123513573-123513593	0.003763372	
UTR3	X:123513108-123513123	0.002056295	
UTR3	X:123511629-123511645	0.002181726	
UTR3	X:123510127-123510153	0.009697181	
UTR3	X:123509935-123509960	0.005474987	
ENST00000263707	ENSG00000115112 (TFCP2L1)	hsa-miR-146a-5p	0.760116
UTR3	2:121981394-121981421	0.003622276	
UTR3	2:121981199-121981222	0.001821588	
UTR3	2:121979406-121979421	0.001446493	
UTR3	2:121979205-121979222	0.015964871	
UTR3	2:121978599-121978622	0.002118843	
UTR3	2:121974472-121974496	0.004581412	
ENST00000298875	ENSG00000165934 (CPSF2)	hsa-miR-146a-5p	0.760062
UTR3	14:92628335-92628362	0.003329643	
UTR3	14:92629217-92629237	0.002273503	
UTR3	14:92630026-92630045	0.013586247	
UTR3	14:92630744-92630749	0.007492676	
UTR3	14:92627498-92627518	0.014357651	

ENST00000441159	ENSG00000170379 (FAM115C)	hsa-miR-146a-5p	0.759974
UTR3	7:143425075-143425102	0.003722528	
UTR3	7:143425885-143425910	0.011081892	
UTR3	7:143426895-143426915	0.012499141	
UTR3	7:143424599-143424621	0.011397659	
ENST00000540438	ENSG00000048028 (USP28)	hsa-miR-146a-5p	0.759736
UTR3	11:113705018-113705043	0.027787795	
UTR3	11:113679138-113679159	0.001733644	
ENST00000436063	ENSG00000067334 (DNTTIP2)	hsa-miR-146a-5p	0.759614
UTR3	1:94334358-94334371	0.006715639	
UTR3	1:94333924-94333950	0.008077036	
UTR3	1:94333391-94333406	0.005787435	
UTR3	1:94342535-94342555	0.019135182	
UTR3	1:94342155-94342176	0.019274125	
ENST00000398637	ENSG00000111371 (SLC38A1)	hsa-miR-146a-5p	0.759425
UTR3	12:46582079-46582096	0.007047058	
UTR3	12:46580082-46580109	0.002686922	
UTR3	12:46579055-46579082	0.004838594	
UTR3	12:46578503-46578522	0.00589066	
UTR3	12:46577625-46577643	0.009030371	
ENST00000295414	ENSG00000163249 (CCNYL1)	hsa-miR-146a-5p	0.759338
UTR3	2:208619125-208619145	0.005154687	
UTR3	2:208620530-208620553	0.011602643	
UTR3	2:208620557-208620585	0.007151993	
UTR3	2:208620835-208620858	0.005576515	
ENST00000260116	ENSG00000137561 (TTPA)	hsa-miR-146a-5p	0.758924
UTR3	8:63973471-63973494	0.009641321	
UTR3	8:63972855-63972878	0.005309769	
UTR3	8:63972369-63972382	0.014497751	
ENST00000526446	ENSG00000110717 (NDUFS8)	hsa-miR-146a-5p	0.75879
UTR3	11:67803849;67803929-67803848;67803948	0.029436872	
ENST00000373119	ENSG00000102271 (KLHL4)	hsa-miR-146a-5p	0.758157
UTR3	X:86922332-86922355	0.003776051	
UTR3	X:86923385-86923403	0.001790437	
UTR3	X:86923823-86923849	0.001176489	
UTR3	X:86924536-86924561	0.020868275	
UTR3	X:86869039;86869437-86869047;86869447	0.009081191	
ENST00000367139	ENSG00000117280 (RAB7L1)	hsa-miR-146a-5p	0.757968
UTR3	1:205739222-205739246	0.003194221	
UTR3	1:205739143-205739170	0.005060097	
UTR3	1:205737268-205737288	0.021109211	

ENST00000439211	ENSG00000228716 (DHFR)	hsa-miR-146a-5p	0.757713
UTR3	5:79924269-79924289	0.009728971	
UTR3	5:79922591-79922618	0.004227688	
UTR3	5:79945221-79945243	0.05740097	
ENST00000369872	ENSG00000143028 (SYPL2)	hsa-miR-146a-5p	0.757569
UTR3	1:110023279-110023306	0.00244687	
UTR3	1:110024141-110024159	0.011675509	
UTR3	1:110024274-110024295	0.002567821	
UTR3	1:110024284-110024305	0.002726834	
UTR3	1:110024302-110024323	0.004494673	
UTR3	1:110024429-110024450	0.002352395	
UTR3	1:110009723-110009749	0.015165148	
ENST00000522481	ENSG00000253896 (AC144568.2)	hsa-miR-146a-5p	0.757464
UTR3	8:29334-29354	0.013698895	
UTR3	8:29354-29373	0.015619784	
ENST00000325203	ENSG00000091879 (ANGPT2)	hsa-miR-146a-5p	0.7574
UTR3	8:6359662-6359679	0.002561476	
UTR3	8:6358925-6358944	0.002050293	
UTR3	8:6357701-6357719	0.004387123	
UTR3	8:6357623-6357642	0.008625816	
UTR3	8:6357611-6357626	0.002804885	
UTR3	8:6357246-6357274	0.008883437	
ENST00000453304	ENSG00000182168 (UNC5C)	hsa-miR-146a-5p	0.757177
UTR3	4:96089287-96089305	0.002649601	
UTR3	4:96089165-96089184	0.010211895	
UTR3	4:96087123-96087145	0.002763001	
UTR3	4:96086624-96086648	0.002838777	
UTR3	4:96084508-96084530	0.010829923	
ENST00000335144	ENSG00000186767 (SPIN4)	hsa-miR-146a-5p	0.756801
UTR3	X:62569888-62569910	0.005433099	
UTR3	X:62569487-62569504	0.004369167	
UTR3	X:62568189-62568205	0.003123306	
UTR3	X:62567237-62567259	0.016334205	
ENST00000380003	ENSG00000147894 (C9orf72)	hsa-miR-146a-5p	0.756728
UTR3	9:27548091-27548099	0.017175663	
UTR3	9:27547624-27547639	0.01207765	
ENST00000223129	ENSG00000106399 (RPA3)	hsa-miR-146a-5p	0.756577
UTR3	7:7676536-7676558	0.004849317	
UTR3	7:7676465-7676491	0.024390549	
ENST00000295797	ENSG00000163558 (PRKCI)	hsa-miR-146a-5p	0.756071
UTR3	3:170022965-170022985	0.029195047	
ENST00000381813	ENSG00000205795 (CYS1)	hsa-miR-146a-5p	0.756053
UTR3	2:10198494-10198518	0.003994981	
UTR3	2:10198127-10198151	0.005865746	

UTR3	2:10197049-10197068	0.019332654	
ENST00000597832	ENSG00000204514 (ZNF814)	hsa-miR-146a-5p	0.755801
UTR3	19:58363262-58363276	0.001903635	
UTR3	19:58362197-58362221	0.027267472	
ENST00000324472	ENSG00000177990 (DPY19L2)	hsa-miR-146a-5p	0.755702
UTR3	12:63953628-63953643	0.011652718	
UTR3	12:63953062-63953088	0.017509614	
ENST00000433351	ENSG00000116005 (PCYOX1)	hsa-miR-146a-5p	0.755523
UTR3	2:70504909-70504925	0.002300138	
UTR3	2:70505560-70505587	0.001907555	
UTR3	2:70505578-70505593	0.005297891	
UTR3	2:70507812-70507838	0.01964091	
ENST00000554557	ENSG00000072121 (ZFYVE26)	hsa-miR-146a-5p	0.755157
UTR3	14:68265239-68265261	0.005809706	
UTR3	14:68265229-68265257	0.003937823	
UTR3	14:68260386-68260399	0.005732104	
UTR3	14:68251801-68251818	0.007379659	
UTR3	14:68241750-68241766	0.004531334	
UTR3	14:68228088-68228116	0.001723477	
ENST00000371244	ENSG00000162409 (PRKAA2)	hsa-miR-146a-5p	0.754761
UTR3	1:57174170-57174179	0.003890047	
UTR3	1:57175233-57175254	0.001561101	
UTR3	1:57176616-57176642	0.001765876	
UTR3	1:57177251-57177277	0.001804601	
UTR3	1:57177313-57177336	0.009836895	
UTR3	1:57179625-57179638	0.008094142	
UTR3	1:57179796-57179815	0.002126449	
ENST00000530414	ENSG00000109832 (DDX25)	hsa-miR-146a-5p	0.754516
UTR3	11:125791921-125791946	0.023297503	
UTR3	11:125788641-125788661	0.026429259	
ENST00000370630	ENSG00000117151 (CTBS)	hsa-miR-146a-5p	0.754448
UTR3	1:85020137-85020161	0.00411006	
UTR3	1:85019638-85019651	0.007650105	
UTR3	1:85018719-85018742	0.002893908	
UTR3	1:85018396-85018410	0.004845695	
UTR3	1:85017717-85017743	0.001990105	
UTR3	1:85016901-85016922	0.007561616	
ENST00000318876	ENSG00000165861 (ZFYVE1)	hsa-miR-146a-5p	0.754165
UTR3	14:73437546-73437572	0.029026544	
ENST00000403491	ENSG00000162599 (NFIA)	hsa-miR-146a-5p	0.754079
UTR3	1:61921023-61921042	0.010774161	
UTR3	1:61921212-61921229	0.007812039	
UTR3	1:61927584-61927596	0.003296429	

UTR3	1:61928357-61928381	0.007136307	
ENST00000377252	ENSG00000120594 (PLXDC2)	hsa-miR-146a-5p	0.753652
UTR3	10:20569122-20569142	0.005014655	
UTR3	10:20570889-20570912	0.001684768	
UTR3	10:20573636-20573661	0.01104128	
UTR3	10:20575402-20575424	0.001111703	
UTR3	10:20578395-20578408	0.007044237	
UTR3	10:20578430-20578457	0.003084747	
ENST00000254442	ENSG00000091157 (WDR7)	hsa-miR-146a-5p	0.753567
UTR3	18:54694454-54694475	0.018427013	
UTR3	18:54694577-54694594	0.002844227	
UTR3	18:54696698-54696715	0.001362395	
UTR3	18:54697017-54697037	0.001856904	
UTR3	18:54348616;54349910-54348622;54349923	0.012922063	
UTR3	18:54424026-54424050	0.008271206	
ENST00000607453	ENSG00000272195 (RP11-156E8.1)	hsa-miR-146a-5p	0.753512
UTR3	1:245132902-245132920	0.028969012	
ENST00000317568	ENSG00000126391 (FRMD8)	hsa-miR-146a-5p	0.753354
UTR3	11:65180335-65180359	0.006687249	
UTR3	11:65180932-65180953	0.022267852	
ENST00000391736	ENSG00000186818 (LILRB4)	hsa-miR-146a-5p	0.753108
UTR3	19:55181109-55181128	0.016679578	
UTR3	19:55181678-55181691	0.01225395	
ENST00000476906	ENSG00000261210 (CLEC19A)	hsa-miR-146a-5p	0.753014
UTR3	16:19310099-19310121	0.028925222	
ENST00000295087	ENSG00000162980 (ARL5A)	hsa-miR-146a-5p	0.75291
UTR3	2:152659605-152659625	0.005994233	
UTR3	2:152659555-152659578	0.004655337	
UTR3	2:152659177-152659204	0.005748492	
UTR3	2:152658020-152658033	0.001619297	
UTR3	2:152658010-152658033	0.000665343	
UTR3	2:152657163-152657185	0.008088019	
UTR3	2:152656276-152656292	0.002145347	
ENST00000329122	ENSG00000184724 (KRTAP6-1)	hsa-miR-146a-5p	0.752671
UTR3	21:31985802-31985820	0.028895136	
ENST00000565367	ENSG00000069943 (PIGB)	hsa-miR-146a-5p	0.752641
UTR3	15:55632855-55632880	0.026474523	
UTR3	15:55621979-55622000	0.012034782	
ENST00000266673	ENSG00000139291 (TMEM19)	hsa-miR-146a-5p	0.75211
UTR3	12:72095134-72095160	0.024177891	
UTR3	12:72096185-72096206	0.001079554	
UTR3	12:72091254-72091278	0.017298997	

ENST00000280098	ENSG00000144228 (SPOPL)	hsa-miR-146a-5p	0.75186
UTR3	2:139327055-139327079	0.013899793	
UTR3	2:139327685-139327710	0.002606678	
UTR3	2:139330213-139330235	0.012317472	
ENST00000381118	ENSG00000049167 (ERCC8)	hsa-miR-146a-5p	0.751708
UTR3	5:60220199-60220221	0.017478999	
UTR3	5:60199486-60199511	0.011331708	
ENST00000539981	ENSG00000163539 (CLASP2)	hsa-miR-146a-5p	0.751648
UTR3	3:33540045-33540071	0.018968249	
UTR3	3:33538832-33538855	0.003264926	
UTR3	3:33538256-33538266	0.003902216	
UTR3	3:33614743-33614755	0.013170542	
ENST00000273221	ENSG00000144711 (IQSEC1)	hsa-miR-146a-5p	0.750946
UTR3	3:12940191-12940217	0.004006398	
UTR3	3:12939592-12939619	0.001567302	
UTR3	3:12939561-12939581	0.001128368	
UTR3	3:12939182-12939202	0.019361265	
UTR3	3:12978019-12978041	0.013197979	
ENST00000242848	ENSG00000123200 (ZC3H13)	hsa-miR-146a-5p	0.750857
UTR3	13:46529739-46529756	0.00234056	
UTR3	13:46529692-46529720	0.008479488	
UTR3	13:46529356-46529371	0.003392516	
UTR3	13:46559533-46559558	0.0108338	
UTR3	13:46554115-46554138	0.010199069	
UTR3	13:46542122-46542147	0.011913528	
UTR3	13:46541812-46541829	0.021522614	
ENST00000481579	ENSG00000077254 (USP33)	hsa-miR-146a-5p	0.750818
UTR3	1:78162315-78162331	0.02873281	
ENST00000397412	ENSG00000007545 (CRAMP1L)	hsa-miR-146a-5p	0.750812
UTR3	16:1724929-1724944	0.023778891	
UTR3	16:1725250-1725267	0.002015429	
UTR3	16:1726012-1726038	0.001665467	
UTR3	16:1726213-1726230	0.001272542	
ENST00000337743	ENSG00000089123 (TASP1)	hsa-miR-146a-5p	0.750551
UTR3	20:13370455-13370482	0.023028811	
UTR3	20:13370068-13370086	0.005680683	
ENST00000377122	ENSG00000078114 (NEBL)	hsa-miR-146a-5p	0.749784
UTR3	10:21074068-21074094	0.005675404	
UTR3	10:21073345-21073363	0.001292988	
UTR3	10:21071921-21071947	0.005169158	
UTR3	10:21071758-21071776	0.002637018	
UTR3	10:21070567-21070594	0.001441142	
UTR3	10:21069690-21069712	0.00986586	
UTR3	10:21069136-21069157	0.002561081	
ENST00000530758	ENSG00000254999 (BRK1)	hsa-miR-146a-5p	0.749575
UTR3	3:10168317-10168340	0.028624427	

ENST00000389063	ENSG00000172795 (DCP2)	hsa-miR-146a-5p	0.749272
UTR3	5:112349249-112349264	0.012024438	
UTR3	5:112352416-112352430	0.001247987	
UTR3	5:112353185-112353200	0.001393052	
UTR3	5:112353620-112353648	0.002378867	
UTR3	5:112354933-112354956	0.005470883	
UTR3	5:112355582-112355593	0.006082857	
ENST00000407627	ENSG00000066044 (ELAVL1)	hsa-miR-146a-5p	0.749232
UTR3	19:8027933-8027959	0.003996286	
UTR3	19:8027280-8027295	0.012477886	
UTR3	19:8024425-8024444	0.00239629	
UTR3	19:8023784-8023798	0.009724089	
ENST00000274140	ENSG00000145495 (MARCH6)	hsa-miR-146a-5p	0.749214
UTR3	5:10433902-10433924	0.009336636	
UTR3	5:10433938-10433962	0.003289346	
UTR3	5:10435168-10435187	0.015966982	
ENST00000308448	ENSG00000138138 (ATAD1)	hsa-miR-146a-5p	0.749206
UTR3	10:89512862-89512882	0.00202542	
UTR3	10:89511957-89511980	0.001796814	
UTR3	10:89511399-89511415	0.012310153	
UTR3	10:89511371-89511397	0.012459923	
ENST00000435504	ENSG00000143970 (ASXL2)	hsa-miR-146a-5p	0.749064
UTR3	2:25962355-25962380	0.003208564	
UTR3	2:25961005-25961030	0.002373536	
UTR3	2:25960072-25960088	0.003438629	
UTR3	2:25957508-25957520	0.00276348	
UTR3	2:25957054-25957076	0.01679573	
ENST00000503400	ENSG00000005700 (IBTK)	hsa-miR-146a-5p	0.748956
UTR3	6:82927824-82927845	0.014759492	
UTR3	6:82924148-82924168	0.001875469	
UTR3	6:82914619-82914643	0.002567036	
UTR3	6:82900901-82900922	0.003366874	
UTR3	6:82891614-82891630	0.00251115	
UTR3	6:82880965-82880987	0.003490522	
ENST00000359286	ENSG00000196946 (ZNF705A)	hsa-miR-146a-5p	0.748747
UTR3	12:8331099-8331125	0.002519264	
UTR3	12:8331583-8331605	0.002088762	
UTR3	12:8331977-8332000	0.022196226	
UTR3	12:8329890-8329917	0.008778421	
ENST00000261491	ENSG00000102780 (DGKH)	hsa-miR-146a-5p	0.748719
UTR3	13:42806651-42806671	0.001361377	
UTR3	13:42807511-42807535	0.002120909	
UTR3	13:42807695-42807717	0.001773981	
UTR3	13:42811880-42811905	0.001995711	
UTR3	13:42812755-42812781	0.001104906	

UTR3	13:42813647-42813667	0.001625703	
UTR3	13:42814236-42814252	0.001154295	
UTR3	13:42816157-42816174	0.005022002	
UTR3	13:42816987-42817007	0.010221331	
UTR3	13:42784783-42784803	0.010772268	
ENST00000332624	ENSG00000115993 (TRAK2)	hsa-miR-146a-5p	0.748679
UTR3	2:202244213-202244236	0.006015762	
UTR3	2:202243925-202243953	0.002129718	
UTR3	2:202243898-202243919	0.000793046	
UTR3	2:202242503-202242523	0.019608011	
ENST00000561965	ENSG00000167264 (DUS2)	hsa-miR-146a-5p	0.747854
UTR3	16:68112355-68112376	0.00305053	
UTR3	16:68113091-68113105	0.025424401	
ENST00000378116	ENSG00000148468 (FAM171A1)	hsa-miR-146a-5p	0.747709
UTR3	10:15254859-15254875	0.004907986	
UTR3	10:15254584-15254607	0.019008404	
UTR3	10:15254459-15254476	0.002728254	
UTR3	10:15254302-15254321	0.0018177	
ENST00000370695	ENSG00000198689 (SLC9A6)	hsa-miR-146a-5p	0.74765
UTR3	X:135127209-135127228	0.014531219	
UTR3	X:135128097-135128124	0.012809048	
UTR3	X:135128121-135128149	0.001116979	
ENST00000355100	ENSG00000140526 (ABHD2)	hsa-miR-146a-5p	0.747572
UTR3	15:89739363-89739377	0.003136079	
UTR3	15:89740034-89740052	0.002945874	
UTR3	15:89745443-89745465	0.022368537	
ENST00000388712	ENSG00000135052 (GOLM1)	hsa-miR-146a-5p	0.747551
UTR3	9:88642455-88642480	0.013701812	
UTR3	9:88642281-88642304	0.005617207	
UTR3	9:88641136-88641157	0.007153807	
UTR3	9:88661394-88661417	0.009833873	
ENST00000397512	ENSG00000177042 (TMEM80)	hsa-miR-146a-5p	0.747365
UTR3	11:703960-703981	0.028432536	
ENST00000327739	ENSG00000173124 (C10orf129)	hsa-miR-146a-5p	0.747338
UTR3	10:96985507-96985532	0.005640607	
UTR3	10:96985583-96985607	0.01875445	
UTR3	10:96985743-96985766	0.004035129	
ENST00000372037	ENSG00000107779 (BMPR1A)	hsa-miR-146a-5p	0.747063
UTR3	10:88683548-88683576	0.018189198	
UTR3	10:88684413-88684437	0.002973934	
UTR3	10:88691662-88691686	0.003047516	
UTR3	10:88692299-88692323	0.004195765	

ENST00000264711	ENSG00000115137 (DNAJC27)	hsa-miR-146a-5p	0.746784
UTR3	2:25170228-25170247	0.005536992	
UTR3	2:25167399-25167427	0.015557146	
UTR3	2:25167384-25167402	0.001691858	
UTR3	2:25166927-25166941	0.005596267	
ENST00000360135	ENSG00000168781 (PPIP5K1)	hsa-miR-146a-5p	0.746763
UTR3	15:43826717-43826743	0.008184816	
UTR3	15:43826616-43826636	0.01521916	
UTR3	15:43826338-43826363	0.003611942	
UTR3	15:43827014-43827025	0.006891396	
ENST00000394961	ENSG00000163104 (SMARCAD1)	hsa-miR-146a-5p	0.746744
UTR3	4:95174076-95174103	0.010604346	
UTR3	4:95195935;95196041-95195948;95196047	0.009659875	
UTR3	4:95199636-95199651	0.003520548	
UTR3	4:95199648;95199767-95199668;95199773	0.004594094	
ENST00000528609	ENSG00000165923 (AGBL2)	hsa-miR-146a-5p	0.746403
UTR3	11:47690437-47690458	0.008104619	
UTR3	11:47681462-47681484	0.009236071	
UTR3	11:47703643;47711628-47703647;47711649	0.044057271	
ENST00000223073	ENSG00000106344 (RBM28)	hsa-miR-146a-5p	0.745937
UTR3	7:127950554-127950578	0.006947414	
UTR3	7:127948550-127948572	0.001073343	
UTR3	7:127947447-127947465	0.001864247	
UTR3	7:127947337-127947356	0.001746621	
UTR3	7:127945486-127945505	0.001941085	
UTR3	7:127938730-127938743	0.002991324	
UTR3	7:127937990-127938003	0.009737153	
UTR3	7:127957767-127957785	0.009948234	
ENST00000265071	ENSG00000113361 (CDH6)	hsa-miR-146a-5p	0.745872
UTR3	5:31323667-31323694	0.007687153	
UTR3	5:31323979-31323999	0.003759869	
UTR3	5:31325743-31325755	0.003549541	
UTR3	5:31328951-31328970	0.00355894	
UTR3	5:31329145-31329169	0.009748056	
ENST00000558158	ENSG00000166069 (TMCO5A)	hsa-miR-146a-5p	0.745255
UTR3	15:38234505-38234531	0.024609799	
UTR3	15:38235767-38235795	0.003640621	
ENST00000380672	ENSG00000173068 (BNC2)	hsa-miR-146a-5p	0.745249
UTR3	9:16416380-16416405	0.001568865	
UTR3	9:16415378-16415396	0.001671604	

UTR3	9:16410755-16410770	0.002098057	
UTR3	9:16410172-16410192	0.0046937	
UTR3	9:16410165-16410190	0.001242199	
UTR3	9:16409908-16409934	0.015513601	
UTR3	9:16435730-16435746	0.007337621	
ENST00000323571	ENSG00000171475 (WIPF2)	hsa-miR-146a-5p	0.745192
UTR3	17:38434510-38434530	0.008767148	
UTR3	17:38435027-38435051	0.007787225	
UTR3	17:38435162-38435185	0.008310998	
UTR3	17:38438616-38438642	0.001169269	
UTR3	17:38420761-38420784	0.010874106	
ENST00000313806	ENSG00000159200 (RCAN1)	hsa-miR-146a-5p	0.74517
UTR3	21:35889881-35889894	0.008684016	
UTR3	21:35888949-35888967	0.01955906	
ENST00000563428	ENSG00000103168 (TAF1C)	hsa-miR-146a-5p	0.745046
UTR3	16:84213946;84214649-84213950;84214663	0.028232455	
ENST00000278379	ENSG00000110436 (SLC1A2)	hsa-miR-146a-5p	0.744918
UTR3	11:35282223-35282238	0.005383055	
UTR3	11:35282024-35282047	0.003581501	
UTR3	11:35281260-35281287	0.001228736	
UTR3	11:35279972-35279999	0.001067206	
UTR3	11:35279260-35279282	0.001818337	
UTR3	11:35278349-35278373	0.000914673	
UTR3	11:35278013-35278035	0.002164756	
UTR3	11:35277468-35277486	0.002099429	
UTR3	11:35276120-35276145	0.001534929	
UTR3	11:35274191-35274218	0.001268406	
UTR3	11:35273673-35273700	0.005217766	
UTR3	11:35273558-35273573	0.001942589	
ENST00000503129	ENSG00000152670 (DDX4)	hsa-miR-146a-5p	0.744186
UTR3	5:55094324-55094344	0.004741104	
UTR3	5:55094343-55094362	0.023417438	
ENST00000318522	ENSG00000143924 (EML4)	hsa-miR-146a-5p	0.744139
UTR3	2:42558814-42558832	0.006506355	
UTR3	2:42559080-42559097	0.016272795	
UTR3	2:42559310-42559330	0.002220503	
UTR3	2:42557145-42557159	0.015108047	
ENST00000383815	ENSG00000163701 (IL17RE)	hsa-miR-146a-5p	0.743442
UTR3	3:9952842;9952939-9952858;9952943	0.025513479	
UTR3	3:9948543;9948645-9948549;9948657	0.012526569	
ENST00000375859	ENSG00000106723 (SPIN1)	hsa-miR-146a-5p	0.743223
UTR3	9:91092699-91092727	0.00366862	
UTR3	9:91093239-91093256	0.016771442	
UTR3	9:91093470-91093496	0.007635913	

ENST00000425217	ENSG00000171435 (KSR2)	hsa-miR-146a-5p	0.742747
UTR3	12:117904253-117904280	0.001872919	
UTR3	12:117902773-117902796	0.001100254	
UTR3	12:117901663-117901686	0.001210662	
UTR3	12:117900606-117900621	0.00505229	
UTR3	12:117898334-117898359	0.006616551	
UTR3	12:117896986-117897007	0.001553948	
UTR3	12:117895323-117895346	0.000923132	
UTR3	12:117893705-117893728	0.001038116	
UTR3	12:117893278-117893299	0.001181664	
UTR3	12:117892109-117892137	0.001694999	
UTR3	12:117891929-117891950	0.002241032	
UTR3	12:117891131-117891155	0.003549633	
ENST00000281405	ENSG00000118965 (WDR35)	hsa-miR-146a-5p	0.74245
UTR3	2:20113010-20113030	0.00467745	
UTR3	2:20112431-20112446	0.004085916	
UTR3	2:20112418-20112446	0.001609817	
UTR3	2:20112278-20112298	0.002028621	
UTR3	2:20111991-20112009	0.002281082	
UTR3	2:20111572-20111599	0.001277346	
UTR3	2:20111351-20111375	0.001494322	
UTR3	2:20110904-20110926	0.00274985	
UTR3	2:20110784-20110802	0.002809308	
UTR3	2:20110357-20110374	0.00499608	
ENST00000499810	ENSG00000120705 (ETF1)	hsa-miR-146a-5p	0.742291
UTR3	5:137842384-137842410	0.014819097	
UTR3	5:137841992-137842006	0.013177077	
ENST00000395254	ENSG00000138311 (ZNF365)	hsa-miR-146a-5p	0.742203
UTR3	10:64160112-64160139	0.027988653	
ENST00000338316	ENSG00000078295 (ADCY2)	hsa-miR-146a-5p	0.742039
UTR3	5:7827088-7827116	0.003190431	
UTR3	5:7829004-7829031	0.020933708	
UTR3	5:7829258-7829282	0.001786855	
UTR3	5:7829340-7829365	0.00206366	
ENST00000334884	ENSG00000005483 (KMT2E)	hsa-miR-146a-5p	0.741986
UTR3	7:104748114-104748141	0.00876632	
UTR3	7:104750762-104750788	0.002840895	
UTR3	7:104751261-104751281	0.003113296	
UTR3	7:104752915-104752936	0.001794039	
UTR3	7:104753892-104753920	0.003339906	
UTR3	7:104754332-104754359	0.004125799	
UTR3	7:104747624-104747637	0.018619957	
ENST00000443676	ENSG00000215356 (ZNF705B)	hsa-miR-146a-5p	0.741201
UTR3	8:7810832-7810858	0.002197483	
UTR3	8:7811316-7811338	0.002155832	
UTR3	8:7811710-7811733	0.021605778	

UTR3	8:7809624-7809651	0.009545215	
ENST00000525557	ENSG00000140090 (SLC24A4)	hsa-miR-146a-5p	0.741017
UTR3	14:92959860-92959885	0.005757555	
UTR3	14:92960007-92960028	0.002090549	
UTR3	14:92960371-92960395	0.010720382	
UTR3	14:92960596-92960616	0.002742919	
UTR3	14:92961887-92961906	0.006576137	
ENST00000361085	ENSG00000075407 (ZNF37A)	hsa-miR-146a-5p	0.740919
UTR3	10:38407841-38407859	0.026008923	
UTR3	10:38409704-38409730	0.001870289	
ENST00000265843	ENSG00000110723 (EXPH5)	hsa-miR-146a-5p	0.740735
UTR3	11:108379335-108379352	0.003618741	
UTR3	11:108376247-108376270	0.014543727	
UTR3	11:108376236-108376250	0.003897475	
UTR3	11:108409804-108409824	0.00844923	
UTR3	11:108384421-108384440	0.009135927	
UTR3	11:108383011-108383032	0.008257432	
ENST00000524135	ENSG00000185728 (YTHDF3)	hsa-miR-146a-5p	0.740544
UTR3	8:64099029-64099053	0.022180219	
UTR3	8:64124147-64124160	0.002418696	
UTR3	8:64124438-64124463	0.003248372	
ENST00000425534	ENSG00000163412 (EIF4E3)	hsa-miR-146a-5p	0.740531
UTR3	3:71730992-71731005	0.00146791	
UTR3	3:71729548-71729567	0.001305347	
UTR3	3:71728199-71728211	0.008205902	
UTR3	3:71728088-71728110	0.001047364	
UTR3	3:71727420-71727441	0.001795691	
UTR3	3:71726641-71726665	0.001020106	
UTR3	3:71725157-71725173	0.005428295	
UTR3	3:71724601-71724626	0.007575527	
ENST00000534127	ENSG00000166444 (ST5)	hsa-miR-146a-5p	0.740509
UTR3	11:8714929-8714948	0.026295775	
UTR3	11:8752726-8752741	0.007670813	
ENST00000367290	ENSG00000120262 (CCDC170)	hsa-miR-146a-5p	0.739936
UTR3	6:151939597-151939613	0.027795574	
ENST00000377627	ENSG00000041988 (THAP3)	hsa-miR-146a-5p	0.739822
UTR3	1:6695167-6695182	0.027785908	
ENST00000231461	ENSG00000113532 (ST8SIA4)	hsa-miR-146a-5p	0.739547
UTR3	5:100145625-100145646	0.025588906	
UTR3	5:100144706-100144724	0.002173705	
ENST00000581161	ENSG00000176225 (RTTN)	hsa-miR-146a-5p	0.739282
UTR3	18:67812926-67812940	0.004070491	
UTR3	18:67741162-67741187	0.0061682	

UTR3	18:67721391-67721412	0.002225383	
UTR3	18:67715345-67715371	0.001703317	
UTR3	18:67688042;67691958-67688053;67691965	0.013572697	
ENST00000358154	ENSG00000197006 (METTL9)	hsa-miR-146a-5p	0.73916
UTR3	16:21667597-21667619	0.009778258	
UTR3	16:21668000-21668026	0.004550026	
UTR3	16:21668562-21668583	0.008736581	
UTR3	16:21666716-21666742	0.021282407	
ENST00000559724	ENSG00000197299 (BLM)	hsa-miR-146a-5p	0.739077
UTR3	15:91304163-91304179	0.017428668	
UTR3	15:91304443-91304463	0.003744245	
UTR3	15:91358388-91358415	0.006549794	
ENST00000264914	ENSG00000113273 (ARSB)	hsa-miR-146a-5p	0.738514
UTR3	5:78076162-78076182	0.002869956	
UTR3	5:78075930-78075951	0.01755465	
UTR3	5:78075030-78075050	0.002159202	
UTR3	5:78074431-78074452	0.005091263	
ENST00000340057	ENSG00000112115 (IL17A)	hsa-miR-146a-5p	0.7385
UTR3	6:52054827-52054843	0.01732916	
UTR3	6:52054949-52054969	0.010344696	
ENST00000295092	ENSG00000162981 (FAM84A)	hsa-miR-146a-5p	0.73808
UTR3	2:14775043-14775070	0.006701904	
UTR3	2:14775343-14775370	0.002947344	
UTR3	2:14776247-14776261	0.003487858	
UTR3	2:14778135-14778156	0.001793373	
UTR3	2:14778303-14778326	0.001566353	
UTR3	2:14779381-14779407	0.00434844	
UTR3	2:14779554-14779573	0.00269352	
UTR3	2:14779924-14779943	0.00409955	
ENST00000585477	ENSG00000228075 (BOD1L2)	hsa-miR-146a-5p	0.738066
UTR3	18:54816605-54816623	0.003136516	
UTR3	18:54817114-54817127	0.004386204	
UTR3	18:54817186-54817201	0.016575048	
UTR3	18:54814882-54814908	0.016566024	
ENST00000328439	ENSG00000196132 (MYT1)	hsa-miR-146a-5p	0.737797
UTR3	20:62872092-62872112	0.026420864	
UTR3	20:62848507-62848530	0.005931999	
ENST00000529122	ENSG00000254535 (PABPC4L)	hsa-miR-146a-5p	0.73778
UTR3	4:135119689-135119709	0.001685147	
UTR3	4:135118503-135118531	0.003278821	
UTR3	4:135117547-135117563	0.022648966	
ENST00000314011	ENSG00000125945 (ZNF436)	hsa-miR-146a-5p	0.737775
UTR3	1:23687881-23687897	0.002920859	

UTR3	1:23687169-23687196	0.002517414	
UTR3	1:23686512-23686533	0.00254246	
UTR3	1:23686473-23686499	0.009275488	
UTR3	1:23689450-23689472	0.027024564	
UTR3	1:23689252-23689272	0.014346948	
ENST00000373036	ENSG00000188786 (MTF1)	hsa-miR-146a-5p	0.737763
UTR3	1:38279179-38279205	0.001320727	
UTR3	1:38278992-38279019	0.002030099	
UTR3	1:38277993-38278011	0.005188797	
UTR3	1:38276658-38276685	0.001850365	
UTR3	1:38276127-38276145	0.003769872	
UTR3	1:38275969-38275996	0.005237584	
UTR3	1:38275818-38275840	0.005573125	
UTR3	1:38287813-38287827	0.012637803	
ENST00000357250	ENSG00000112280 (COL9A1)	hsa-miR-146a-5p	0.737745
UTR3	6:70926411-70926430	0.01221746	
UTR3	6:70926190-70926210	0.015392523	
ENST00000537676	ENSG00000165192 (ASB11)	hsa-miR-146a-5p	0.737737
UTR3	X:15301443-15301468	0.023827996	
UTR3	X:15300503-15300528	0.003781377	
ENST00000203001	ENSG00000089195 (TRMT6)	hsa-miR-146a-5p	0.737626
UTR3	20:5919148-5919166	0.023041822	
UTR3	20:5918769-5918788	0.004558149	
ENST00000296812	ENSG00000151876 (FBXO4)	hsa-miR-146a-5p	0.737235
UTR3	5:41934534-41934561	0.008842127	
UTR3	5:41935058-41935065	0.018724844	
ENST00000325455	ENSG00000082175 (PGR)	hsa-miR-146a-5p	0.737075
UTR3	11:100909018-100909034	0.002898197	
UTR3	11:100905128-100905148	0.001148552	
UTR3	11:100904014-100904040	0.001993331	
UTR3	11:100902310-100902315	0.001507442	
UTR3	11:100901858-100901875	0.001077059	
UTR3	11:100901540-100901562	0.010947185	
UTR3	11:100901169-100901192	0.005306827	
UTR3	11:100900920-100900938	0.002674858	
ENST00000564533	ENSG00000260240 (RP11-105C20.2)	hsa-miR-146a-5p	0.736848
UTR3	16:59771866-59771886	0.027534364	
ENST00000463144	ENSG00000204410 (MSH5)	hsa-miR-146a-5p	0.736639
UTR3	6:31721103-31721125	0.008810273	
UTR3	6:31721378-31721400	0.004935601	
UTR3	6:31726017-31726030	0.013770835	
ENST00000338056	ENSG00000105784 (RUNDC3B)	hsa-miR-146a-5p	0.736632
UTR3	7:87459838-87459853	0.012158351	
UTR3	7:87460908-87460928	0.012600136	

UTR3	7:87460914-87460936	0.00275762	
ENST00000356205	ENSG00000143570 (SLC39A1)	hsa-miR-146a-5p	0.736169
UTR3	1:153932522-153932539	0.002772303	
UTR3	1:153932452-153932472	0.017727469	
UTR3	1:153931970-153931989	0.006977401	
ENST00000452027	ENSG00000154548 (SRSF12)	hsa-miR-146a-5p	0.735976
UTR3	6:89806949-89806971	0.027460915	
ENST00000287957	ENSG00000157259 (GATAD1)	hsa-miR-146a-5p	0.735863
UTR3	7:92085994-92086021	0.021277475	
UTR3	7:92087047-92087070	0.006173935	
ENST00000554081	ENSG00000185246 (PRPF39)	hsa-miR-146a-5p	0.735199
UTR3	14:45578908-45578932	0.00557445	
UTR3	14:45581641-45581660	0.002759825	
UTR3	14:45584961-45584983	0.004866203	
UTR3	14:45585161-45585173	0.01419511	
ENST00000438447	ENSG00000133401 (PDZD2)	hsa-miR-146a-5p	0.735049
UTR3	5:32108255-32108271	0.024524222	
UTR3	5:32109990-32110014	0.001723403	
UTR3	5:32090222-32090244	0.005617387	
ENST00000593946	ENSG00000039650 (PNKP)	hsa-miR-146a-5p	0.734939
UTR3	19:50365669-50365688	0.003132535	
UTR3	19:50365342;50365442-50365362;50365449	0.00304718	
UTR3	19:50364515-50364539	0.021194076	
ENST00000313070	ENSG00000081803 (CADPS2)	hsa-miR-146a-5p	0.734715
UTR3	7:121959842-121959850	0.00478924	
UTR3	7:121959106-121959128	0.003667822	
UTR3	7:121959048-121959066	0.009716258	
UTR3	7:121958866-121958880	0.004219968	
UTR3	7:121958664-121958684	0.004961735	
ENST00000586178	ENSG00000011258 (MBTD1)	hsa-miR-146a-5p	0.734704
UTR3	17:49257277-49257302	0.006482296	
UTR3	17:49257099-49257126	0.007625253	
UTR3	17:49256140-49256157	0.001997146	
UTR3	17:49255957-49255983	0.002976594	
UTR3	17:49254967-49254995	0.008272781	
ENST00000376701	ENSG00000015285 (WAS)	hsa-miR-146a-5p	0.73465
UTR3	X:48549731-48549755	0.006914588	
UTR3	X:48549748-48549761	0.020434987	
ENST00000354452	ENSG00000196628 (TCF4)	hsa-miR-146a-5p	0.734453
UTR3	18:52894372-52894391	0.005397486	
UTR3	18:52893419-52893431	0.003296146	
UTR3	18:52892519-52892539	0.009591558	
UTR3	18:52891555-52891568	0.002930244	

UTR3	18:52890168-52890190	0.006117567	
ENST00000416691	ENSG00000133103 (COG6)	hsa-miR-146a-5p	0.73429
UTR3	13:40363169-40363195	0.009979994	
UTR3	13:40364358-40364378	0.002528366	
UTR3	13:40364999-40365015	0.002936137	
UTR3	13:40365012-40365035	0.001329693	
UTR3	13:40365720-40365729	0.007244967	
UTR3	13:40253707-40253731	0.015416381	
ENST00000394008	ENSG00000198083 (KRTAP9-9)	hsa-miR-146a-5p	0.733869
UTR3	17:39412242-39412252	0.010420289	
UTR3	17:39412359-39412380	0.016863805	
ENST00000532592	ENSG00000170743 (SYT9)	hsa-miR-146a-5p	0.733817
UTR3	11:7489041-7489066	0.001731113	
UTR3	11:7489498-7489514	0.016514964	
UTR3	11:7490193-7490219	0.009033736	
ENST00000209875	ENSG00000094916 (CBX5)	hsa-miR-146a-5p	0.733622
UTR3	12:54635386-54635402	0.011437923	
UTR3	12:54632906-54632931	0.002459292	
UTR3	12:54630985-54631006	0.001886556	
UTR3	12:54629577-54629594	0.002302396	
UTR3	12:54628661-54628680	0.002529992	
UTR3	12:54627871-54627890	0.002029345	
UTR3	12:54625272-54625296	0.004617961	
ENST00000361377	ENSG00000164172 (MOCS2)	hsa-miR-146a-5p	0.733536
UTR3	5:52393851-52393867	0.027256281	
ENST00000373218	ENSG00000148730 (EIF4EBP2)	hsa-miR-146a-5p	0.73297
UTR3	10:72184543-72184567	0.009499709	
UTR3	10:72184844-72184870	0.003111461	
UTR3	10:72185256-72185273	0.007410466	
UTR3	10:72188305-72188327	0.00718737	
ENST00000551632	ENSG00000178498 (DTX3)	hsa-miR-146a-5p	0.732615
UTR3	12:58003156-58003172	0.002243689	
UTR3	12:58003408-58003426	0.024935642	
ENST00000354449	ENSG00000197548 (ATG7)	hsa-miR-146a-5p	0.732446
UTR3	3:11597745-11597765	0.001179585	
UTR3	3:11598004-11598031	0.014601651	
UTR3	3:11598244-11598266	0.011384029	
ENST00000318238	ENSG00000177409 (SAMD9L)	hsa-miR-146a-5p	0.732425
UTR3	7:92760458-92760477	0.004957551	
UTR3	7:92759734-92759759	0.005318299	
UTR3	7:92759708-92759734	0.00280099	
UTR3	7:92759548-92759569	0.014086669	
ENST00000491614	ENSG00000165609 (NUDT5)	hsa-miR-146a-5p	0.732203
UTR3	10:12209360-12209373	0.024624672	

UTR3	10:12208683-12208697	0.001705083	
UTR3	10:12208671-12208697	0.000815194	
ENST00000320672	ENSG00000145687 (SSBP2)	hsa-miR-146a-5p	0.731934
UTR3	5:80715650-80715675	0.003898335	
UTR3	5:80715129-80715150	0.004271012	
UTR3	5:80712983-80713004	0.001502898	
UTR3	5:80712678-80712706	0.001893006	
UTR3	5:80712240-80712265	0.002516015	
UTR3	5:80711089-80711114	0.003981479	
UTR3	5:80710458-80710482	0.001349791	
UTR3	5:80709081-80709105	0.007710063	
ENST00000328747	ENSG00000146757 (ZNF92)	hsa-miR-146a-5p	0.73189
UTR3	7:64853807;64863254-64853814;64863266	0.015000645	
UTR3	7:64863903-64863921	0.01721057	
UTR3	7:64864071-64864089	0.016438553	
UTR3	7:64864659-64864677	0.030454349	
ENST00000597774	ENSG00000196961 (AP2A1)	hsa-miR-146a-5p	0.73143
UTR3	19:50302677-50302703	0.011382961	
UTR3	19:50304243;50304660-50304256;50304664	0.013607156	
UTR3	19:50306572-50306599	0.002090491	
ENST00000502346	ENSG00000174695 (TMEM167A)	hsa-miR-146a-5p	0.731404
UTR3	5:82352768-82352783	0.009677789	
UTR3	5:82350797-82350814	0.001643411	
UTR3	5:82350452-82350477	0.008203916	
UTR3	5:82349440-82349454	0.003947701	
UTR3	5:82349092-82349118	0.003605638	
ENST00000493594	ENSG00000117226 (GBP3)	hsa-miR-146a-5p	0.73119
UTR3	1:89473013-89473038	0.027060633	
ENST00000285208	ENSG00000154917 (RAB6B)	hsa-miR-146a-5p	0.731025
UTR3	3:133547225-133547244	0.004460754	
UTR3	3:133546876-133546889	0.002668254	
UTR3	3:133546036-133546063	0.002016221	
UTR3	3:133545202-133545219	0.001086443	
UTR3	3:133545055-133545079	0.000919666	
UTR3	3:133544689-133544708	0.006965892	
UTR3	3:133543086-133543097	0.008929674	
ENST00000341516	ENSG00000139990 (DCAF5)	hsa-miR-146a-5p	0.730972
UTR3	14:69520197-69520215	0.012257123	
UTR3	14:69519487-69519503	0.003348623	
UTR3	14:69518458-69518480	0.0022644	
UTR3	14:69517697-69517714	0.004831934	
UTR3	14:69584886-69584909	0.013321774	
UTR3	14:69558600;69583083-69558604;69583105	0.006306571	

ENST00000367635	ENSG00000116191 (RALGPS2)	hsa-miR-146a-5p	0.73045
UTR3	1:178885610-178885626	0.016649975	
UTR3	1:178885988-178886011	0.002462159	
UTR3	1:178886027-178886045	0.002334513	
UTR3	1:178887019-178887045	0.001806963	
UTR3	1:178863106-178863121	0.017167767	
ENST00000413432	ENSG00000006607 (FARP2)	hsa-miR-146a-5p	0.730415
UTR3	2:242383392-242383408	0.001536109	
UTR3	2:242384525-242384543	0.001346798	
UTR3	2:242385154-242385179	0.007124595	
UTR3	2:242386093-242386119	0.006161804	
UTR3	2:242386151-242386163	0.008902427	
UTR3	2:242381695-242381719	0.009233577	
ENST00000273375	ENSG00000144840 (RABL3)	hsa-miR-146a-5p	0.729535
UTR3	3:120408481-120408504	0.004271288	
UTR3	3:120407232-120407248	0.001541299	
UTR3	3:120405695-120405719	0.016837611	
UTR3	3:120405532-120405554	0.004273095	
ENST00000326407	ENSG00000135916 (ITM2C)	hsa-miR-146a-5p	0.728941
UTR3	2:231743433-231743456	0.003981531	
UTR3	2:231743767-231743780	0.006415481	
UTR3	2:231743809-231743825	0.006959177	
UTR3	2:231743890-231743909	0.00951798	
ENST00000220244	ENSG00000103888 (KIAA1199)	hsa-miR-146a-5p	0.728868
UTR3	15:81243603-81243621	0.024180375	
UTR3	15:81234367-81234390	0.012606234	
ENST00000477053	ENSG00000134490 (TMEM241)	hsa-miR-146a-5p	0.728842
UTR3	18:20932231;20936558-20932253;20936561	0.006520917	
UTR3	18:20877989-20878007	0.00494183	
UTR3	18:20877197-20877211	0.001625192	
UTR3	18:20876007-20876020	0.01377804	
ENST00000448849	ENSG00000134897 (BIVM)	hsa-miR-146a-5p	0.728793
UTR3	13:103492339-103492353	0.024493085	
UTR3	13:103493269-103493292	0.002368777	
ENST00000480058	ENSG00000112739 (PRPF4B)	hsa-miR-146a-5p	0.728702
UTR3	6:4062076-4062100	0.009701209	
UTR3	6:4062397-4062421	0.003332188	
UTR3	6:4064554-4064567	0.013820951	
ENST00000367588	ENSG00000135835 (KIAA1614)	hsa-miR-146a-5p	0.728611
UTR3	1:180915319-180915335	0.004844488	
UTR3	1:180915564-180915580	0.011100525	
UTR3	1:180915897-180915916	0.010221201	

UTR3	1:180905580-180905602	0.003359965	
ENST00000559192	ENSG00000140455 (USP3)	hsa-miR-146a-5p	0.728557
UTR3	15:63883306-63883327	0.019188363	
UTR3	15:63886075-63886099	0.002605	
UTR3	15:63886810-63886828	0.005049014	
ENST00000392438	ENSG00000198836 (OPA1)	hsa-miR-146a-5p	0.728522
UTR3	3:193412654-193412681	0.004347384	
UTR3	3:193413050-193413078	0.002105715	
UTR3	3:193415041-193415061	0.014528414	
UTR3	3:193332650-193332667	0.015852153	
UTR3	3:193360583-193360606	0.009578091	
ENST00000529689	ENSG00000137500 (CCDC90B)	hsa-miR-146a-5p	0.72852
UTR3	11:82970573-82970595	0.002783952	
UTR3	11:82970498-82970515	0.003473714	
UTR3	11:82970202-82970225	0.020581681	
ENST00000261438	ENSG00000109787 (KLF3)	hsa-miR-146a-5p	0.728448
UTR3	4:38699030-38699056	0.007705201	
UTR3	4:38699193-38699210	0.019128144	
ENST00000370125	ENSG00000079335 (CDC14A)	hsa-miR-146a-5p	0.728422
UTR3	1:100964495-100964516	0.020949811	
UTR3	1:100984811-100984829	0.005881424	
ENST00000376040	ENSG00000135040 (NAA35)	hsa-miR-146a-5p	0.72841
UTR3	9:88573457-88573481	0.078262116	
ENST00000375321	ENSG00000107968 (MAP3K8)	hsa-miR-146a-5p	0.728219
UTR3	10:30749949-30749970	0.023472576	
UTR3	10:30739370-30739397	0.015400193	
ENST00000298198	ENSG00000165434 (PGM2L1)	hsa-miR-146a-5p	0.727476
UTR3	11:74046996-74047021	0.002778655	
UTR3	11:74046985-74047011	0.002527521	
UTR3	11:74045883-74045898	0.011609185	
UTR3	11:74045551-74045566	0.001825589	
UTR3	11:74044365-74044380	0.001901888	
UTR3	11:74041496-74041520	0.006110285	
ENST00000273666	ENSG00000145087 (STXBP5L)	hsa-miR-146a-5p	0.727135
UTR3	3:121138547-121138574	0.003957856	
UTR3	3:121139695-121139714	0.007813951	
UTR3	3:121140171-121140196	0.002295955	
UTR3	3:121140667-121140695	0.001290831	
UTR3	3:121141557-121141583	0.001319065	
UTR3	3:121141584-121141605	0.001087527	
UTR3	3:121142413-121142432	0.002532684	
UTR3	3:121142920-121142936	0.0056564	

UTR3	3:120628538-120628564	0.003783782	
ENST00000607197	ENSG00000051620 (HEBP2)	hsa-miR-146a-5p	0.727103
UTR3	6:138740959-138740983	0.006811306	
UTR3	6:138741689-138741710	0.001589795	
UTR3	6:138742862-138742885	0.01832135	
ENST00000312431	ENSG00000151914 (DST)	hsa-miR-146a-5p	0.727026
UTR3	6:56417400-56417421	0.001895465	
UTR3	6:56357206-56357224	0.003104473	
UTR3	6:56352005-56352031	0.0020256	
UTR3	6:56323888-56323905	0.003168137	
UTR3	6:56322923-56322944	0.008240217	
UTR3	6:56473076-56473090	0.017161522	
UTR3	6:56438542-56438554	0.016780119	
ENST00000392054	ENSG00000153823 (PID1)	hsa-miR-146a-5p	0.72685
UTR3	2:229890085-229890103	0.004555012	
UTR3	2:229889168-229889181	0.022146538	
ENST00000316397	ENSG00000197414 (GOLGA6L1)	hsa-miR-146a-5p	0.726818
UTR3	15:22744730-22744745	0.009591	
UTR3	15:22744977-22744996	0.006945674	
UTR3	15:22745756-22745770	0.010162249	
ENST00000369681	ENSG00000065615 (CYB5R4)	hsa-miR-146a-5p	0.72614
UTR3	6:84671167-84671181	0.002569469	
UTR3	6:84671178-84671201	0.00252392	
UTR3	6:84671467-84671488	0.002347885	
UTR3	6:84672095-84672122	0.001861066	
UTR3	6:84672545-84672561	0.001644236	
UTR3	6:84673371-84673391	0.00522266	
UTR3	6:84673601-84673617	0.003295376	
UTR3	6:84675544-84675558	0.003676758	
UTR3	6:84665124-84665138	0.016006377	
ENST00000231721	ENSG00000010319 (SEMA3G)	hsa-miR-146a-5p	0.725983
UTR3	3:52469351-52469376	0.025347128	
UTR3	3:52467995-52468019	0.001283177	
ENST00000514176	ENSG00000109805 (NCAPG)	hsa-miR-146a-5p	0.725971
UTR3	4:17818994-17819012	0.011998186	
UTR3	4:17838817-17838834	0.006128265	
UTR3	4:17845019-17845031	0.008502861	
ENST00000388825	ENSG00000211445 (GPX3)	hsa-miR-146a-5p	0.725339
UTR3	5:150408013-150408033	0.018362035	
UTR3	5:150408253-150408269	0.008215348	
ENST00000465254	ENSG00000189367 (KIAA0408)	hsa-miR-146a-5p	0.725324
UTR3	6:127765166-127765182	0.00619066	
UTR3	6:127764154-127764173	0.002408908	
UTR3	6:127762530-127762552	0.017976614	

ENST00000371117	ENSG00000170927 (PKHD1)	hsa-miR-146a-5p	0.725256
UTR3	6:51483224-51483246	0.003063342	
UTR3	6:51480990-51481010	0.004959784	
UTR3	6:51927354-51927370	0.013182963	
UTR3	6:51875229-51875257	0.04253556	
UTR3	6:51695668-51695689	0.006143828	
ENST00000367996	ENSG00000158859 (ADAMTS4)	hsa-miR-146a-5p	0.725243
UTR3	1:161160210-161160230	0.002278636	
UTR3	1:161159616-161159643	0.001811067	
UTR3	1:161156829-161156846	0.00205079	
UTR3	1:161156298-161156318	0.002759304	
UTR3	1:161154101-161154123	0.016436043	
UTR3	1:161163537-161163563	0.005959314	
ENST00000411937	ENSG00000170390 (DCLK2)	hsa-miR-146a-5p	0.725224
UTR3	4:151178507-151178526	0.026567943	
ENST00000291825	ENSG00000105227 (PRX)	hsa-miR-146a-5p	0.724766
UTR3	19:40903783-40903806	0.010920553	
UTR3	19:40903768-40903794	0.008143914	
UTR3	19:40901464-40901482	0.007465992	
ENST00000373191	ENSG00000126070 (AGO3)	hsa-miR-146a-5p	0.72474
UTR3	1:36522106-36522129	0.016083051	
UTR3	1:36525752-36525770	0.001010397	
UTR3	1:36532580-36532603	0.001010097	
UTR3	1:36532963-36532978	0.002197626	
UTR3	1:36534002-36534024	0.001291508	
UTR3	1:36535016-36535041	0.004935648	
ENST00000497136	ENSG00000120008 (WDR11)	hsa-miR-146a-5p	0.724431
UTR3	10:122665424-122665451	0.002998541	
UTR3	10:122665467-122665489	0.00059876	
UTR3	10:122665568-122665594	0.000513586	
UTR3	10:122668128-122668144	0.007870633	
UTR3	10:122668527-122668554	0.005164918	
UTR3	10:122668597-122668617	0.002771677	
UTR3	10:122668910-122668930	0.006584875	
ENST00000371464	ENSG00000138207 (RBP4)	hsa-miR-146a-5p	0.723963
UTR3	10:95351581-95351607	0.025224947	
UTR3	10:95360481-95360493	0.005971435	
ENST00000338983	ENSG00000091436 (MLTK)	hsa-miR-146a-5p	0.723639
UTR3	2:174087078-174087096	0.012309652	
UTR3	2:174087552-174087568	0.001547266	
UTR3	2:174089347-174089373	0.002499978	
UTR3	2:174091323-174091337	0.003076922	
UTR3	2:174091608-174091613	0.007004375	
ENST00000396065	ENSG00000140297 (GCNT3)	hsa-miR-146a-5p	0.723307
UTR3	15:59911928-59911952	0.002807163	
UTR3	15:59911964-59911991	0.004228182	

UTR3	15:59912268-59912285	0.003646424	
UTR3	15:59912309-59912335	0.015729345	
ENST00000445930	ENSG00000100519 (PSMC6)	hsa-miR-146a-5p	0.723163
UTR3	14:53194687-53194707	0.026399359	
ENST00000374045	ENSG00000181634 (TNFSF15)	hsa-miR-146a-5p	0.723126
UTR3	9:117551105-117551126	0.001187995	
UTR3	9:117550749-117550761	0.001869002	
UTR3	9:117550664-117550690	0.001096389	
UTR3	9:117550480-117550507	0.002405811	
UTR3	9:117549598-117549618	0.001500167	
UTR3	9:117548772-117548793	0.014787199	
UTR3	9:117547890-117547908	0.001703009	
UTR3	9:117547701-117547719	0.001846717	
ENST00000522372	ENSG00000215262 (KCNU1)	hsa-miR-146a-5p	0.723029
UTR3	8:36793125-36793141	0.02638842	
ENST00000225719	ENSG00000108582 (CPD)	hsa-miR-146a-5p	0.722677
UTR3	17:28793275-28793294	0.001939602	
UTR3	17:28794048-28794073	0.001734219	
UTR3	17:28794769-28794788	0.002229838	
UTR3	17:28796863-28796886	0.006429324	
UTR3	17:28796955-28796979	0.01402673	
ENST00000327835	ENSG00000164684 (ZNF704)	hsa-miR-146a-5p	0.72258
UTR3	8:81552311-81552337	0.001598726	
UTR3	8:81550731-81550757	0.002707824	
UTR3	8:81549058-81549085	0.002154335	
UTR3	8:81548321-81548341	0.003371963	
UTR3	8:81545746-81545770	0.006002798	
UTR3	8:81542010-81542030	0.002140493	
UTR3	8:81541699-81541725	0.003310561	
UTR3	8:81541355-81541370	0.005065047	
ENST00000337190	ENSG00000123933 (MXD4)	hsa-miR-146a-5p	0.722415
UTR3	4:2252207-2252230	0.014780699	
UTR3	4:2251996-2252013	0.008523784	
UTR3	4:2251236-2251256	0.001775334	
UTR3	4:2250742-2250765	0.001258486	
ENST00000537989	ENSG00000112182 (BACH2)	hsa-miR-146a-5p	0.722211
UTR3	6:90641808-90641826	0.005504197	
UTR3	6:90641618-90641642	0.007590394	
UTR3	6:90641592-90641616	0.004668391	
UTR3	6:90639242-90639264	0.001268894	
UTR3	6:90638042-90638068	0.00158229	
UTR3	6:90636750-90636777	0.0045142	
UTR3	6:90660188-90660205	0.005733717	
ENST00000488729	ENSG00000134313 (KIDINS220)	hsa-miR-146a-5p	0.722204
UTR3	2:8953395-8953420	0.005051681	

UTR3	2:8931277-8931295	0.002071019	
UTR3	2:8930137-8930156	0.005304765	
UTR3	2:8871383-8871400	0.01153972	
UTR3	2:8871037-8871062	0.002353901	
ENST00000381254	ENSG00000178295 (GEN1)	hsa-miR-146a-5p	0.722141
UTR3	2:17963285-17963311	0.005798488	
UTR3	2:17964000-17964021	0.004782558	
UTR3	2:17965596-17965616	0.002272135	
UTR3	2:17962927-17962953	0.02968433	
UTR3	2:17963080-17963102	0.012267818	
UTR3	2:17963085-17963111	0.007195161	
ENST00000351217	ENSG00000156642 (NPTN)	hsa-miR-146a-5p	0.722017
UTR3	15:73853320-73853340	0.004761932	
UTR3	15:73852471-73852486	0.021543923	
ENST00000450363	ENSG00000072041 (SLC6A15)	hsa-miR-146a-5p	0.721984
UTR3	12:85277323-85277343	0.023612735	
UTR3	12:85275932-85275952	0.001509941	
UTR3	12:85275827-85275848	0.00118052	
ENST00000536534	ENSG00000180287 (PLD5)	hsa-miR-146a-5p	0.721641
UTR3	1:242252564-242252579	0.00366527	
UTR3	1:242250768-242250789	0.018114574	
UTR3	1:242249920-242249937	0.001503777	
UTR3	1:242248735-242248762	0.001708895	
UTR3	1:242247751-242247774	0.001282744	
ENST00000451905	ENSG00000233539 (AC011294.3)	hsa-miR-146a-5p	0.721522
UTR3	7:46727686-46727700	0.014462619	
UTR3	7:46727666-46727689	0.011803022	
ENST00000369498	ENSG00000165775 (FUNDC2)	hsa-miR-146a-5p	0.721463
UTR3	X:154286092-154286103	0.001252906	
UTR3	X:154286775-154286800	0.00154851	
UTR3	X:154286892-154286916	0.000626569	
UTR3	X:154288115-154288135	0.022832822	
ENST00000338700	ENSG00000109654 (TRIM2)	hsa-miR-146a-5p	0.721435
UTR3	4:154256161-154256179	0.005162217	
UTR3	4:154256702-154256728	0.002949663	
UTR3	4:154260045-154260065	0.018146628	
ENST00000396150	ENSG00000083828 (ZNF586)	hsa-miR-146a-5p	0.721137
UTR3	19:58291564-58291586	0.020584107	
UTR3	19:58291715-58291730	0.005650221	
ENST00000335056	ENSG00000104970 (KIR3DX1)	hsa-miR-146a-5p	0.720991
UTR3	19:55055482-55055495	0.010838469	
UTR3	19:55055855-55055882	0.009406111	
UTR3	19:55055995-55056008	0.002411176	

UTR3	19:55056091-55056115	0.00239749	
UTR3	19:55044953-55044969	0.005606962	
ENST00000392583	ENSG00000111335 (OAS2)	hsa-miR-146a-5p	0.720866
UTR3	12:113447712-113447731	0.004653492	
UTR3	12:113447755-113447766	0.002390687	
UTR3	12:113448216-113448241	0.011087402	
UTR3	12:113448613-113448639	0.008080673	
ENST00000263636	ENSG00000054219 (LY75)	hsa-miR-146a-5p	0.720497
UTR3	2:160660002-160660028	0.015852195	
UTR3	2:160673426-160673446	0.012881021	
UTR3	2:160661677-160661701	0.027181369	
ENST00000359202	ENSG00000132446 (FTHL17)	hsa-miR-146a-5p	0.720432
UTR3	X:31089432-31089450	0.004831089	
UTR3	X:31089377-31089387	0.021345935	
ENST00000315571	ENSG00000179241 (LDLRAD3)	hsa-miR-146a-5p	0.720249
UTR3	11:36251681-36251704	0.014549684	
UTR3	11:36253421-36253439	0.005270181	
UTR3	11:36119989-36120009	0.026808001	
ENST00000425538	ENSG00000141052 (MYOCD)	hsa-miR-146a-5p	0.720246
UTR3	17:12667175-12667201	0.006378475	
UTR3	17:12667259-12667276	0.004177591	
UTR3	17:12667294-12667317	0.003088802	
UTR3	17:12668069-12668095	0.001185617	
UTR3	17:12668982-12668995	0.002628447	
UTR3	17:12670426-12670442	0.001293842	
UTR3	17:12671385-12671408	0.001926563	
UTR3	17:12672060-12672076	0.005482622	
ENST00000341861	ENSG00000074603 (DPP8)	hsa-miR-146a-5p	0.719517
UTR3	15:65738580-65738600	0.003944539	
UTR3	15:65737613-65737628	0.002330516	
UTR3	15:65735775-65735801	0.008882991	
UTR3	15:65735743-65735769	0.00189357	
UTR3	15:65735679-65735698	0.002084898	
UTR3	15:65734904-65734921	0.006966326	
ENST00000376387	ENSG00000125304 (TM9SF2)	hsa-miR-146a-5p	0.719299
UTR3	13:100215232-100215243	0.026085263	
ENST00000529193	ENSG00000157613 (CREB3L1)	hsa-miR-146a-5p	0.719048
UTR3	11:46342461-46342486	0.020980148	
UTR3	11:46342504-46342521	0.00393429	
UTR3	11:46338953-46338973	0.005497625	
ENST00000439924	ENSG00000182149 (IST1)	hsa-miR-146a-5p	0.718969
UTR3	16:71956536-71956562	0.006790926	
UTR3	16:71961859-71961885	0.01079788	
UTR3	16:71962168-71962190	0.002560685	

UTR3	16:71962343-71962370	0.005909015	
ENST00000323037	ENSG00000180233 (ZNRF2)	hsa-miR-146a-5p	0.718846
UTR3	7:30405972-30405989	0.020747647	
UTR3	7:30406559-30406572	0.005300892	
ENST00000334082	ENSG00000152413 (HOMER1)	hsa-miR-146a-5p	0.718661
UTR3	5:78671360-78671377	0.006638862	
UTR3	5:78671185-78671208	0.003777381	
UTR3	5:78670634-78670660	0.002604742	
UTR3	5:78669262-78669288	0.002572374	
UTR3	5:78669082-78669091	0.005480701	
UTR3	5:78668889-78668912	0.004959565	
ENST00000396368	ENSG00000166783 (KIAA0430)	hsa-miR-146a-5p	0.718614
UTR3	16:15689067-15689088	0.020750935	
UTR3	16:15718747-15718768	0.01085051	
UTR3	16:15703543-15703558	0.011943225	
ENST00000436636	ENSG00000154310 (TNIK)	hsa-miR-146a-5p	0.718146
UTR3	3:170780592-170780612	0.00227554	
UTR3	3:170780533-170780543	0.001935208	
UTR3	3:170779648-170779671	0.018696733	
UTR3	3:170779604-170779630	0.003084497	
ENST00000607544	ENSG00000226763 (SRRM5)	hsa-miR-146a-5p	0.718118
UTR3	19:44118535-44118554	0.025989759	
ENST00000382177	ENSG00000159459 (UBR1)	hsa-miR-146a-5p	0.71782
UTR3	15:43317119-43317143	0.008144251	
UTR3	15:43262730-43262756	0.002677494	
UTR3	15:43244572-43244596	0.009041734	
UTR3	15:43236909-43236934	0.001179033	
UTR3	15:43236834-43236860	0.002406252	
UTR3	15:43235585-43235599	0.002516951	
ENST00000483141	ENSG00000101473 (ACOT8)	hsa-miR-146a-5p	0.717691
UTR3	20:44471689-44471715	0.022248996	
UTR3	20:44471463-44471484	0.003706303	
ENST00000427134	ENSG00000204308 (RNF5)	hsa-miR-146a-5p	0.717488
UTR3	6:32151186-32151213	0.025938944	
ENST00000472232	ENSG00000107262 (BAG1)	hsa-miR-146a-5p	0.717318
UTR3	9:33255074-33255092	0.023121762	
UTR3	9:33254419-33254439	0.002803437	
ENST00000399231	ENSG00000197535 (MYO5A)	hsa-miR-146a-5p	0.717068
UTR3	15:52604424-52604439	0.007706837	
UTR3	15:52603954-52603978	0.001359991	
UTR3	15:52603418-52603433	0.000979016	
UTR3	15:52602946-52602969	0.001978687	
UTR3	15:52601046-52601066	0.006379806	
UTR3	15:52600982-52601007	0.004503038	
UTR3	15:52600494-52600518	0.002997702	

ENST00000336216	ENSG00000140382 (HMG20A)	hsa-miR-146a-5p	0.717008
UTR3	15:77776309-77776324	0.002792753	
UTR3	15:77776866-77776884	0.001921316	
UTR3	15:77777052-77777067	0.00566704	
UTR3	15:77777662-77777681	0.015519138	
ENST00000486995	ENSG00000049883 (PTCD2)	hsa-miR-146a-5p	0.716906
UTR3	5:71627201;71630845-71627202;71630865	0.002703114	
UTR3	5:71638810-71638823	0.004133965	
UTR3	5:71648547-71648572	0.001513331	
UTR3	5:71654683-71654699	0.004715019	
UTR3	5:71654804-71654828	0.007122298	
UTR3	5:71654864-71654890	0.004833174	
UTR3	5:71616241-71616260	0.004174086	
ENST00000585916	ENSG00000078043 (PIAS2)	hsa-miR-146a-5p	0.716362
UTR3	18:44391578-44391604	0.004852735	
UTR3	18:44391402-44391422	0.002690861	
UTR3	18:44391302-44391325	0.003125522	
UTR3	18:44390821-44390842	0.003965566	
UTR3	18:44390690-44390701	0.001444115	
UTR3	18:44470561-44470588	0.020678038	
UTR3	18:44401028;44407922-44401035;44407935	0.017389033	
ENST00000304032	ENSG00000157985 (AGAP1)	hsa-miR-146a-5p	0.716136
UTR3	2:237036104-237036126	0.002784975	
UTR3	2:237036171-237036197	0.000876893	
UTR3	2:237036828-237036852	0.001173276	
UTR3	2:237039426-237039442	0.002920315	
UTR3	2:237039928-237039950	0.018074637	
ENST00000333012	ENSG00000123427 (METTL21B)	hsa-miR-146a-5p	0.716069
UTR3	12:58174434-58174452	0.00402399	
UTR3	12:58174656-58174683	0.009181485	
UTR3	12:58175824-58175836	0.005253402	
UTR3	12:58176022-58176042	0.003554498	
UTR3	12:58176022-58176050	0.003811267	
ENST00000503329	ENSG00000172901 (AQPEP)	hsa-miR-146a-5p	0.715943
UTR3	5:115341551-115341573	0.020732803	
UTR3	5:115361939-115361960	0.001944679	
UTR3	5:115362826-115362853	0.003137082	
ENST00000592623	ENSG00000115268 (RPS15)	hsa-miR-146a-5p	0.715613
UTR3	19:1440368-1440382	0.025788045	
ENST00000312377	ENSG00000161800 (RACGAP1)	hsa-miR-146a-5p	0.715538
UTR3	12:50383546-50383560	0.025782028	
ENST00000252252	ENSG00000185479 (KRT6B)	hsa-miR-146a-5p	0.715523

UTR3	12:52840830-52840848	0.004614934	
UTR3	12:52840584-52840598	0.021165887	
ENST00000561038	ENSG00000100938 (GMPR2)	hsa-miR-146a-5p	0.715041
UTR3	14:24703432;24704943-24703447;24704947	0.018359543	
UTR3	14:24707666-24707692	0.007382606	
ENST00000562435	ENSG00000171208 (NETO2)	hsa-miR-146a-5p	0.714807
UTR3	16:47116434-47116456	0.003135125	
UTR3	16:47115557-47115581	0.002761684	
UTR3	16:47115491-47115509	0.001229274	
UTR3	16:47113775-47113803	0.00271124	
UTR3	16:47111638-47111661	0.01588606	
ENST00000414099	ENSG00000183873 (SCN5A)	hsa-miR-146a-5p	0.714582
UTR3	3:38591357-38591385	0.010033587	
UTR3	3:38590608-38590627	0.004340165	
UTR3	3:38590048-38590075	0.011331559	
ENST00000399138	ENSG00000091542 (ALKBH5)	hsa-miR-146a-5p	0.714412
UTR3	17:18111886-18111905	0.019185963	
UTR3	17:18112176-18112199	0.003441018	
UTR3	17:18113109-18113126	0.00306473	
ENST00000417640	ENSG00000123119 (NECAB1)	hsa-miR-146a-5p	0.714071
UTR3	8:91967924-91967946	0.005064738	
UTR3	8:91969015-91969033	0.003099428	
UTR3	8:91969066-91969089	0.007496204	
UTR3	8:91971091-91971102	0.007945032	
UTR3	8:91836986-91837001	0.009519732	
ENST00000369443	ENSG00000196505 (GDAP2)	hsa-miR-146a-5p	0.713835
UTR3	1:118410920-118410934	0.001750168	
UTR3	1:118410232-118410251	0.001116296	
UTR3	1:118409233-118409250	0.001067352	
UTR3	1:118408982-118409003	0.001475156	
UTR3	1:118408893-118408921	0.00062145	
UTR3	1:118407395-118407415	0.001835208	
UTR3	1:118406934-118406957	0.003906327	
UTR3	1:118406757-118406778	0.004943286	
UTR3	1:118406267-118406280	0.008930254	
ENST00000334241	ENSG00000186300 (ZNF555)	hsa-miR-146a-5p	0.713464
UTR3	19:2854824-2854846	0.002183291	
UTR3	19:2856751-2856771	0.0029283	
UTR3	19:2856784-2856811	0.005779217	
UTR3	19:2857035-2857054	0.001350279	
UTR3	19:2858549-2858567	0.009083549	
UTR3	19:2859642-2859662	0.004291216	
ENST00000317605	ENSG00000181541 (MAB21L2)	hsa-miR-146a-5p	0.713391

UTR3	4:151505726-151505745	0.025609967	
ENST00000397762	ENSG00000129472 (RAB2B)	hsa-miR-146a-5p	0.713046
UTR3	14:21928652-21928671	0.005444332	
UTR3	14:21927494-21927516	0.016026036	
UTR3	14:21927271-21927289	0.004112104	
ENST00000368936	ENSG00000143363 (PRUNE)	hsa-miR-146a-5p	0.712834
UTR3	1:151006808-151006832	0.02556549	
ENST00000479198	ENSG00000163630 (SYNPR)	hsa-miR-146a-5p	0.712473
UTR3	3:63602188-63602214	0.005318345	
UTR3	3:63602292-63602308	0.020218365	
ENST00000532428	ENSG00000149089 (APIP)	hsa-miR-146a-5p	0.712459
UTR3	11:34877794-34877815	0.009787496	
UTR3	11:34876948-34876968	0.002163944	
UTR3	11:34874829-34874857	0.013584102	
ENST00000462898	ENSG00000051382 (PIK3CB)	hsa-miR-146a-5p	0.71242
UTR3	3:138452279;138453476-138452280;138453500	0.005770467	
UTR3	3:138409968;138413628-138409985;138413637	0.001935952	
UTR3	3:138474665-138474685	0.059121434	
ENST00000428443	ENSG00000187231 (SESTD1)	hsa-miR-146a-5p	0.71224
UTR3	2:179973763-179973781	0.003698019	
UTR3	2:179972630-179972652	0.00150294	
UTR3	2:179971399-179971413	0.0020344	
UTR3	2:179971078-179971086	0.001146653	
UTR3	2:179969095-179969114	0.001346571	
UTR3	2:179968898-179968923	0.001516781	
UTR3	2:179968839-179968864	0.000798582	
UTR3	2:179966867-179966892	0.013474133	
ENST00000602786	ENSG00000120694 (HSPH1)	hsa-miR-146a-5p	0.712226
UTR3	13:31722177-31722205	0.025516982	
ENST00000512701	ENSG00000155158 (TTC39B)	hsa-miR-146a-5p	0.711609
UTR3	9:15167380-15167406	0.001481228	
UTR3	9:15165746-15165760	0.001427863	
UTR3	9:15165726-15165749	0.002416139	
UTR3	9:15165591-15165606	0.000686969	
UTR3	9:15164880-15164904	0.00246548	
UTR3	9:15164804-15164832	0.002131546	
UTR3	9:15164547-15164565	0.002802147	
UTR3	9:15164236-15164252	0.00442806	
UTR3	9:15163967-15163982	0.002881567	
UTR3	9:15163703-15163728	0.004746804	
ENST00000369663	ENSG00000112837 (TBX18)	hsa-miR-146a-5p	0.711572
UTR3	6:85446114-85446134	0.021991993	
UTR3	6:85442757-85442770	0.003472913	
ENST00000608469	ENSG00000134365 (CFHR4)	hsa-miR-146a-5p	0.711106
UTR3	1:196887675-196887691	0.025427749	

ENST00000296257	ENSG00000163904 (SENP2)	hsa-miR-146a-5p	0.710996
UTR3	3:185347716-185347737	0.009809193	
UTR3	3:185349674-185349689	0.005097237	
UTR3	3:185350722-185350746	0.00335592	
UTR3	3:185327121;185329439-185327138;185329444	0.0292191	
ENST00000286428	ENSG00000155959 (VBP1)	hsa-miR-146a-5p	0.710455
UTR3	X:154467826-154467833	0.025375987	
ENST00000308488	ENSG00000048649 (RSF1)	hsa-miR-146a-5p	0.710327
UTR3	11:77375360-77375387	0.00115089	
UTR3	11:77373796-77373817	0.001433872	
UTR3	11:77372362-77372383	0.002382776	
UTR3	11:77371937-77371952	0.003826515	
UTR3	11:77371615-77371632	0.016571774	
ENST00000369686	ENSG00000134245 (WNT2B)	hsa-miR-146a-5p	0.710281
UTR3	1:113064590-113064614	0.001858088	
UTR3	1:113065673-113065696	0.002534488	
UTR3	1:113065785-113065806	0.003508181	
UTR3	1:113066795-113066819	0.00215	
UTR3	1:113067104-113067123	0.001973615	
UTR3	1:113067949-113067972	0.001017641	
UTR3	1:113069976-113070003	0.001404489	
UTR3	1:113071925-113071938	0.006273391	
UTR3	1:113072181-113072202	0.004642305	
ENST00000451899	ENSG00000164823 (OSGIN2)	hsa-miR-146a-5p	0.710149
UTR3	8:90938933-90938950	0.007978669	
UTR3	8:90936971-90936985	0.019805437	
UTR3	8:90937474-90937489	0.038093755	
ENST00000475381	ENSG00000144959 (NCEH1)	hsa-miR-146a-5p	0.710146
UTR3	3:172348390-172348411	0.003954758	
UTR3	3:172348081-172348100	0.021396739	
ENST00000296487	ENSG00000164088 (PPM1M)	hsa-miR-146a-5p	0.710134
UTR3	3:52283605-52283622	0.011529361	
UTR3	3:52283673-52283692	0.013821139	
ENST00000341287	ENSG00000188674 (C2orf80)	hsa-miR-146a-5p	0.709741
UTR3	2:209030119-209030136	0.025319346	
ENST00000249883	ENSG00000114019 (AMOTL2)	hsa-miR-146a-5p	0.709734
UTR3	3:134076092-134076106	0.004525076	
UTR3	3:134075960-134075986	0.002573719	
UTR3	3:134075177-134075191	0.01821999	
ENST00000423484	ENSG00000234444 (ZNF736)	hsa-miR-146a-5p	0.709731
UTR3	7:63810125-63810147	0.002558939	
UTR3	7:63810429-63810454	0.0022883	

UTR3	7:63810483-63810504	0.009482078	
UTR3	7:63811256-63811273	0.001420319	
UTR3	7:63811539-63811559	0.001192906	
UTR3	7:63808782-63808799	0.012779621	
UTR3	7:63809117-63809135	0.008810815	
UTR3	7:63809455-63809471	0.011646785	
ENST00000517608	ENSG00000104343 (UBE2W)	hsa-miR-146a-5p	0.709514
UTR3	8:74705004-74705027	0.005495796	
UTR3	8:74704469-74704493	0.002810388	
UTR3	8:74704262-74704279	0.001907198	
UTR3	8:74703409-74703427	0.001873212	
UTR3	8:74702447-74702466	0.001300952	
UTR3	8:74701754-74701778	0.001567831	
UTR3	8:74700977-74701004	0.001728822	
UTR3	8:74700944-74700971	0.000609613	
UTR3	8:74699261-74699285	0.004001668	
UTR3	8:74698591-74698605	0.004005871	
ENST00000356860	ENSG00000153956 (CACNA2D1)	hsa-miR-146a-5p	0.708986
UTR3	7:81578481-81578498	0.017401946	
UTR3	7:81577606-81577625	0.002181764	
UTR3	7:81575913-81575929	0.005675782	
ENST00000176763	ENSG00000072786 (STK10)	hsa-miR-146a-5p	0.708664
UTR3	5:171471344-171471366	0.002517595	
UTR3	5:171469919-171469935	0.002769577	
UTR3	5:171469803-171469827	0.009676107	
UTR3	5:171469136-171469155	0.00839473	
UTR3	5:171481694;171482592-171481698;171482614	0.008620403	
ENST00000469154	ENSG00000082014 (SMARCD3)	hsa-miR-146a-5p	0.708582
UTR3	7:150939293-150939318	0.019477971	
UTR3	7:150938530-150938554	0.003162988	
UTR3	7:150937970-150937998	0.002586525	
ENST00000473989	ENSG00000157741 (UBN2)	hsa-miR-146a-5p	0.708547
UTR3	7:138983882-138983908	0.002116829	
UTR3	7:138984635-138984654	0.001883594	
UTR3	7:138984934-138984960	0.001813076	
UTR3	7:138986561-138986575	0.007141499	
UTR3	7:138988747-138988766	0.002071541	
UTR3	7:138990070-138990081	0.001792762	
UTR3	7:138990226-138990246	0.001968507	
UTR3	7:138990610-138990630	0.002102894	
UTR3	7:138991151-138991174	0.001560305	
UTR3	7:138968607-138968632	0.012467289	
ENST00000283131	ENSG00000153147 (SMARCA5)	hsa-miR-146a-5p	0.70831

UTR3	4:144475433-144475460	0.023631278	
UTR3	4:144475539-144475562	0.001574659	
ENST00000334464	ENSG00000165650 (PDZD8)	hsa-miR-146a-5p	0.708023
UTR3	10:119041846-119041858	0.014480358	
UTR3	10:119041566-119041588	0.00657857	
UTR3	10:119043413-119043440	0.017936468	
ENST00000442697	ENSG00000164031 (DNAJB14)	hsa-miR-146a-5p	0.707968
UTR3	4:100819567-100819584	0.001181713	
UTR3	4:100817413-100817424	0.019725039	
UTR3	4:100851608-100851629	0.018513732	
ENST00000327946	ENSG00000182771 (GRID1)	hsa-miR-146a-5p	0.707789
UTR3	10:87361298-87361312	0.007235146	
UTR3	10:87359492-87359507	0.017929661	
ENST00000314400	ENSG00000163608 (C3orf17)	hsa-miR-146a-5p	0.70741
UTR3	3:112723769-112723796	0.009343446	
UTR3	3:112723256-112723281	0.002021838	
UTR3	3:112723233-112723245	0.00346225	
UTR3	3:112721725-112721740	0.002143604	
UTR3	3:112727249-112727275	0.021460736	
UTR3	3:112724669-112724686	0.010953925	
ENST00000366429	ENSG00000173421 (CCDC36)	hsa-miR-146a-5p	0.707403
UTR3	3:49283036-49283043	0.025134251	
ENST00000382968	ENSG00000178222 (RNF212)	hsa-miR-146a-5p	0.707339
UTR3	4:1066426-1066448	0.0176062	
UTR3	4:1065949-1065975	0.004561857	
UTR3	4:1065895-1065920	0.002961165	
ENST00000427594	ENSG00000176771 (NCKAP5)	hsa-miR-146a-5p	0.707303
UTR3	2:133879677-133879692	0.00633239	
UTR3	2:133879657-133879675	0.01401701	
UTR3	2:133887553-133887572	0.020436963	
ENST00000328945	ENSG00000184860 (SDR42E1)	hsa-miR-146a-5p	0.707181
UTR3	16:82032668-82032694	0.016082651	
UTR3	16:82031993-82032020	0.003112384	
UTR3	16:82032751-82032771	0.024680741	
ENST00000380750	ENSG00000205572 (SERF1B)	hsa-miR-146a-5p	0.707083
UTR3	5:69337839-69337859	0.014277308	
UTR3	5:69338532-69338550	0.006172483	
UTR3	5:69338645-69338671	0.004659243	
ENST00000394123	ENSG00000145907 (G3BP1)	hsa-miR-146a-5p	0.706981
UTR3	5:151184161-151184187	0.003261946	
UTR3	5:151184819-151184838	0.005113378	
UTR3	5:151186415-151186437	0.004335171	

UTR3	5:151186627-151186639	0.00477848	
UTR3	5:151187109-151187136	0.002131474	
UTR3	5:151188789-151188814	0.00172792	
UTR3	5:151189387-151189401	0.001274087	
UTR3	5:151190798-151190814	0.001455574	
UTR3	5:151190912-151190927	0.001022971	
ENST00000464950	ENSG00000058799 (YIPF1)	hsa-miR-146a-5p	0.706525
UTR3	1:54317939;54320674-54317943;54320687	0.025064992	
ENST00000475390	ENSG00000121871 (SLITRK3)	hsa-miR-146a-5p	0.706307
UTR3	3:164905123-164905137	0.025047824	
ENST00000219905	ENSG00000174197 (MGA)	hsa-miR-146a-5p	0.706246
UTR3	15:42060141-42060164	0.010581522	
UTR3	15:42060607-42060621	0.004584916	
UTR3	15:42061649-42061672	0.00987661	
ENST00000409444	ENSG00000155755 (TMEM237)	hsa-miR-146a-5p	0.705909
UTR3	2:202488712-202488723	0.005285775	
UTR3	2:202488368-202488396	0.0042626	
UTR3	2:202488343-202488363	0.005739256	
UTR3	2:202488142-202488155	0.002166953	
UTR3	2:202487688-202487703	0.007561873	
ENST00000396573	ENSG00000183454 (GRIN2A)	hsa-miR-146a-5p	0.705884
UTR3	16:9856625-9856646	0.004416685	
UTR3	16:9855462-9855482	0.001381727	
UTR3	16:9852639-9852664	0.015417175	
UTR3	16:10032108-10032129	0.008409629	
UTR3	16:10031891-10031910	0.008172272	
ENST00000503039	ENSG00000113761 (ZNF346)	hsa-miR-146a-5p	0.705555
UTR3	5:176508148-176508175	0.024988648	
ENST00000313234	ENSG00000167767 (KRT80)	hsa-miR-146a-5p	0.705507
UTR3	12:52564182-52564199	0.001235714	
UTR3	12:52564079-52564107	0.001150523	
UTR3	12:52563151-52563176	0.009162707	
UTR3	12:52562985-52563008	0.013435889	
ENST00000220597	ENSG00000076641 (PAG1)	hsa-miR-146a-5p	0.70537
UTR3	8:81888635-81888653	0.003713386	
UTR3	8:81887676-81887690	0.002204876	
UTR3	8:81885245-81885263	0.002006279	
UTR3	8:81881129-81881151	0.011722228	
UTR3	8:81880692-81880713	0.005327333	
ENST00000237201	ENSG00000118434 (SPACA1)	hsa-miR-146a-5p	0.705148
UTR3	6:88776237-88776254	0.02495663	
ENST00000370550	ENSG00000153936 (HS2ST1)	hsa-miR-146a-5p	0.705113

UTR3	1:87570418-87570442	0.008976504	
UTR3	1:87571013-87571032	0.004547892	
UTR3	1:87571431-87571454	0.0031361	
UTR3	1:87573698-87573722	0.002181782	
UTR3	1:87575597-87575615	0.006111626	
ENST00000225512	ENSG00000108379 (WNT3)	hsa-miR-146a-5p	0.705031
UTR3	17:44841739-44841759	0.015535959	
UTR3	17:44841468-44841494	0.003709932	
UTR3	17:44841324-44841341	0.002242488	
UTR3	17:44840699-44840715	0.003459043	
ENST00000360311	ENSG00000197385 (ZNF860)	hsa-miR-146a-5p	0.704803
UTR3	3:32032938-32032957	0.004455493	
UTR3	3:32032974-32033000	0.01367657	
UTR3	3:32030774-32030796	0.027673547	
ENST00000541428	ENSG00000198363 (ASPH)	hsa-miR-146a-5p	0.704397
UTR3	8:62415246-62415269	0.002123036	
UTR3	8:62414082-62414102	0.018977921	
UTR3	8:62413553-62413570	0.003796677	
ENST00000327470	ENSG00000183161 (FANCF)	hsa-miR-146a-5p	0.704277
UTR3	11:22646166-22646191	0.021722662	
UTR3	11:22644792-22644807	0.003165589	
ENST00000278302	ENSG00000121236 (TRIM6)	hsa-miR-146a-5p	0.704159
UTR3	11:5632641-5632665	0.01501939	
UTR3	11:5633366-5633391	0.009859529	
ENST00000296318	ENSG00000144730 (IL17RD)	hsa-miR-146a-5p	0.703997
UTR3	3:57130277-57130299	0.002988282	
UTR3	3:57129931-57129959	0.016469471	
UTR3	3:57129196-57129205	0.00155689	
UTR3	3:57129162-57129174	0.000714052	
UTR3	3:57128893-57128913	0.001405329	
UTR3	3:57127794-57127819	0.001732231	
ENST00000334513	ENSG00000186364 (NUDT17)	hsa-miR-146a-5p	0.703965
UTR3	1:145586129-145586147	0.023714463	
UTR3	1:145588403-145588418	0.005327905	
ENST00000395811	ENSG00000133030 (MPRIP)	hsa-miR-146a-5p	0.703889
UTR3	17:17089714-17089740	0.000947979	
UTR3	17:17090273-17090296	0.000970185	
UTR3	17:17093180-17093203	0.002700438	
UTR3	17:17093563-17093580	0.004022018	
UTR3	17:17095319-17095339	0.003270642	
UTR3	17:17095458-17095481	0.011168748	
UTR3	17:17030079-17030101	0.008113679	
ENST00000409719	ENSG00000125611 (CHCHD5)	hsa-miR-146a-5p	0.703074
UTR3	2:113344305-113344328	0.005714927	
UTR3	2:113344369-113344388	0.003334216	

UTR3	2:113344839-113344851	0.011646731	
UTR3	2:113345359-113345383	0.004098057	
ENST00000320238	ENSG00000177551 (NHLH2)	hsa-miR-146a-5p	0.702961
UTR3	1:116380433-116380460	0.003754205	
UTR3	1:116380251-116380275	0.012474385	
UTR3	1:116379225-116379248	0.008556497	
ENST00000480174	ENSG00000137941 (TTLL7)	hsa-miR-146a-5p	0.702712
UTR3	1:84372074-84372090	0.021906702	
UTR3	1:84335198-84335213	0.002858876	
ENST00000311381	ENSG00000130349 (C6orf203)	hsa-miR-146a-5p	0.702457
UTR3	6:107372505-107372531	0.024745631	
ENST00000261292	ENSG00000101670 (LIPG)	hsa-miR-146a-5p	0.702387
UTR3	18:47116976-47116998	0.002354678	
UTR3	18:47117368-47117390	0.006395358	
UTR3	18:47118588-47118609	0.012137497	
UTR3	18:47119051-47119077	0.003852674	
ENST00000357637	ENSG00000197563 (PIGN)	hsa-miR-146a-5p	0.702311
UTR3	18:59712514-59712536	0.002878617	
UTR3	18:59712504-59712526	0.001434815	
UTR3	18:59774108;59774219-59774114;59774239	0.029385373	
UTR3	18:59763151-59763169	0.008120807	
UTR3	18:59763143-59763169	0.006636351	
UTR3	18:59756056;59757709-59756075;59757710	0.019731675	
ENST00000367342	ENSG00000163362 (C1orf106)	hsa-miR-146a-5p	0.702223
UTR3	1:200883084-200883102	0.004597919	
UTR3	1:200884156-200884178	0.009785827	
UTR3	1:200884363-200884385	0.010343611	
ENST00000533482	ENSG00000113522 (RAD50)	hsa-miR-146a-5p	0.702121
UTR3	5:131915054-131915073	0.014726256	
UTR3	5:131923722-131923748	0.003606611	
UTR3	5:131978115-131978127	0.00638655	
ENST00000423058	ENSG00000144285 (SCN1A)	hsa-miR-146a-5p	0.701963
UTR3	2:166847536-166847562	0.021975956	
UTR3	2:166909393-166909420	0.012136633	
ENST00000367895	ENSG00000112319 (EYA4)	hsa-miR-146a-5p	0.701685
UTR3	6:133850301-133850323	0.005353135	
UTR3	6:133851189-133851211	0.00290307	
UTR3	6:133851334-133851344	0.00173238	
UTR3	6:133851344-133851371	0.00090888	
UTR3	6:133851496-133851523	0.001952821	
UTR3	6:133853215-133853224	0.011835037	
ENST00000233336	ENSG00000114999 (TTL)	hsa-miR-146a-5p	0.701679
UTR3	2:113287117-113287141	0.002889275	

UTR3	2:113289054-113289076	0.011267141	
UTR3	2:113290994-113291007	0.004181814	
UTR3	2:113295494-113295503	0.002063608	
UTR3	2:113295587-113295601	0.001113602	
UTR3	2:113296029-113296055	0.001494869	
UTR3	2:113298365-113298389	0.00167457	
ENST00000434618	ENSG00000231925 (TAPBP)	hsa-miR-146a-5p	0.701588
UTR3	6:33268996-33269017	0.02316588	
UTR3	6:33281009-33281032	0.006914784	
ENST00000318789	ENSG00000114861 (FOXP1)	hsa-miR-146a-5p	0.701546
UTR3	3:71008311-71008328	0.007921231	
UTR3	3:71006634-71006654	0.002684158	
UTR3	3:71005494-71005517	0.00208375	
UTR3	3:71005034-71005050	0.007664543	
UTR3	3:71004672-71004694	0.004320792	
ENST00000601302	ENSG00000268223 (ARL14EPL)	hsa-miR-146a-5p	0.701427
UTR3	5:115395495-115395516	0.024665163	
ENST00000370521	ENSG00000065243 (PKN2)	hsa-miR-146a-5p	0.700675
UTR3	1:89301045-89301064	0.020493695	
UTR3	1:89301886-89301906	0.004112851	
ENST00000206595	ENSG00000092140 (G2E3)	hsa-miR-146a-5p	0.700609
UTR3	14:31085932-31085956	0.004835105	
UTR3	14:31087192-31087215	0.001772556	
UTR3	14:31085700-31085724	0.058534159	
ENST00000349496	ENSG00000168036 (CTNNB1)	hsa-miR-146a-5p	0.70055
UTR3	3:41280996-41281013	0.024596796	
ENST00000380079	ENSG00000127954 (STEAP4)	hsa-miR-146a-5p	0.700486
UTR3	7:87907586-87907602	0.003676025	
UTR3	7:87907556-87907573	0.003279148	
UTR3	7:87906073-87906085	0.011629472	
UTR3	7:87913296-87913320	0.024700116	
ENST00000426077	ENSG00000226479 (TMEM185B)	hsa-miR-146a-5p	0.700466
UTR3	2:120979090-120979109	0.01334669	
UTR3	2:120978896-120978915	0.011243547	
ENST00000420627	ENSG00000196924 (FLNA)	hsa-miR-146a-5p	0.70028
UTR3	X:153590437-153590461	0.011915998	
UTR3	X:153590096-153590119	0.001218754	
UTR3	X:153576936-153576949	0.011441047	
ENST00000216877	ENSG00000132670 (PTPRA)	hsa-miR-146a-5p	0.700188
UTR3	20:3019131-3019138	0.022310316	
UTR3	20:3019379-3019404	0.002258305	
ENST00000546931	ENSG00000170627 (GTSF1)	hsa-miR-146a-5p	0.70018
UTR3	12:54849888-54849902	0.024567978	

ENST00000504771	ENSG00000145779 (TNFAIP8)	hsa-miR-146a-5p	0.700114
UTR3	5:118729700-118729721	0.009593539	
UTR3	5:118729731-118729753	0.001890359	
UTR3	5:118731034-118731057	0.001316546	
UTR3	5:118731973-118731989	0.001679385	
UTR3	5:118734761-118734784	0.010083051	

Supplementary table 4: targets for miRNAs hsa-miR-146a-5p
